# Responses of hyperthermophilic crenarchaea to UV irradiation

**DOI:** 10.1186/gb-2007-8-10-r220

**Published:** 2007-10-11

**Authors:** Dorothee Götz, Sonia Paytubi, Stacey Munro, Magnus Lundgren, Rolf Bernander, Malcolm F White

**Affiliations:** 1 Aquapharm Bio-Discoveries, European Centre for Marine Biotechnology, Dunbeg, Oban PA37 1QA, UK; 2 Centre for Biomolecular Sciences, University of St Andrews, North Haugh, St Andrews KY16 9ST, UK; 3 Department of Molecular Evolution, Uppsala University, Norbyvägen 18C, SE-752 36, Uppsala, Sweden

## Abstract

**Background:**

DNA damage leads to cellular responses that include the increased expression of DNA repair genes, repression of DNA replication and alterations in cellular metabolism. Archaeal information processing pathways resemble those in eukaryotes, but archaeal damage response pathways remain poorly understood.

**Results:**

We analyzed the transcriptional response to UV irradiation in two related crenarchaea, *Sulfolobus solfataricus *and *Sulfolobus acidocaldarius*. *Sulfolobus *species encounter high levels of DNA damage in nature, as they inhabit high temperature, aerobic environments and are exposed to sunlight. No increase in expression of DNA repair genes following UV irradiation was observed. There was, however, a clear transcriptional response, including repression of DNA replication and chromatin proteins. Differential effects on the expression of the three transcription factor B (*tfb*) genes hint at a mechanism for the modulation of transcriptional patterns in response to DNA damage. TFB3, which is strongly induced following UV irradiation, competes with TFB1 for binding to RNA polymerase *in vitro*, and may act as a repressor of transcription or an alternative transcription factor for certain promoters.

**Conclusion:**

A clear response to DNA damage was observed, with down-regulation of the DNA replication machinery, changes in transcriptional regulatory proteins, and up-regulation of the biosynthetic enzymes for beta-carotene, which has UV protective properties, and proteins that detoxify reactive oxygen species. However, unlike eukaryotes and bacteria, there was no induction of DNA repair proteins in response to DNA damage, probably because these are expressed constitutively to deal with increased damage arising due to high growth temperatures.

## Abstract 2

The transcriptional response to UV irradiation was analyzed in two related crenarchaea, Sulfolobus solfataricus and Sulfolobus acidocaldarius, showing a clear response to DNA damage but no increase in the expression of DNA repair genes.

## Background

The maintenance of genomic integrity is a crucial task for every living thing, and all organisms devote considerable resources to the repair of DNA damage. Many environmental factors contribute to the overall load of DNA damage suffered by a cell. The crenarchaeal *Sulfolobus *species probably suffer more than most, due to growth at 80°C, leading to more rapid reactions such as hydrolytic deamination of nucleotide bases, an aerobic lifestyle resulting in DNA damage by reactive oxygen species (ROS) and exposure to UV irradiation. Despite this high damage load, the rate of mutation in *Sulfolobus acidocaldarius *is no higher than for mesophilic organisms such as *Escherichia coli *[1], indicating that DNA damage is repaired efficiently.

Whilst archaea have eukaryal-type informational pathways such as DNA replication and transcription, archaeal DNA repair pathways are still poorly understood. In brief, archaeal DNA repair appears to be a mosaic of universal, bacterial and eukaryal-type repair proteins and pathways [2,3]. Base excision repair proteins, for example, are common to all three domains of life, whilst double-strand break repair appears to be at least partially conserved between eukarya and archaea. Some key proteins, such as mismatch repair and DNA damage recognition proteins, have not yet been identified in archaea, although the bacterial UvrABC system is present in some species, including halophiles, where it is involved in the repair of UV damage [4].

In addition to DNA repair proteins, bacteria and eukaryotes have damage response pathways whose function is to detect DNA damage and modulate cellular processes. These include control of the transcriptional repertoire of the cell, activation of repair enzymes and repression of DNA replication and cell division. In general, the aim is to ensure that DNA repair proteins are activated and that DNA replication is delayed until repair can be completed. Perhaps the best understood example is the bacterial SOS response first proposed by Miro Radman [5]. Under normal growth conditions, where levels of DNA damage are low, transcription of repair genes is repressed. When DNA damage occurs, a damage signal corresponding to the strand exchange protein RecA bound to single-stranded DNA causes the LexA repressor to be degraded, resulting in the induction of transcription of a large number of repair proteins.

There are no clear archaeal homologues of either bacterial LexA or of eukaryal proteins involved in the transcriptional response to DNA damage, such as p53. Experimental studies of the archaeal DNA damage response have been very limited. Analysis of the transcriptional response of the euryarchaeon *Halobacterium *NRC-1 to UV irradiation showed no evidence for a concerted up-regulation of genes encoding DNA repair proteins [6]. An investigation of the expression levels of a limited number of repair proteins in *Pyrococcus abyssi *showed little or no induction following ionizing irradiation, but found that DNA replication was inhibited in damaged cells, consistent with the need to repair damage before replication is resumed [7]. UV irradiation of *S. solfataricus *downregulated transcription of the gene encoding the chromatin protein Sso7 and upregulated transcription levels of the gene for the putative repair protein XPB1, with evidence for a change in transcriptional start site [8]. Thus, the preliminary evidence suggests that archaea do have a DNA damage response that includes both transcriptional repression and activation, and inhibition of other cellular processes whilst repair is carried out. However, it is still unknown how DNA damage is detected, how this signal results in changes in DNA replication and transcription, and how general these mechanisms are across the diverse archaeal lineage. For example, recent studies have demonstrated that *S. solfataricus *shows no bias for quicker repair of transcribed versus non-transcribed strands, whilst transcription-coupled repair is common in the eukaryal and bacterial domains [9,10].

To begin to address these questions, we undertook a comprehensive study of the cellular response of the related crenarchaea *S. solfataricus *and *S. acidocaldarius *to DNA damage caused by UV irradiation. Changes in global transcription levels were assessed by whole-genome microarray analyses, and changes in the expression levels of key genes were confirmed by quantitative RT-PCR and western blotting. Flow cytometry was used to examine the effect of DNA damage on the *Sulfolobus *cell cycle. We show that UV irradiation does not alter expression levels of DNA repair genes, suggesting constitutive expression of repair proteins in *Sulfolobus *species. We observed repression of genes encoding DNA replication and chromatin proteins, consistent with the inhibition of DNA replication to allow repair to take place. A few genes were highly induced following UV irradiation, including those encoding the Cdc6-2 protein, the DNA polymerase DpoII, the putative transcriptional repressor transcription factor (II) B (TFB)3, a group of proteins providing protection from the effects of ROS, and some proteins of unknown function. These observations suggest that hyperthermophilic archaea have a programmed cellular response to DNA damage caused by UV irradiation.

## Results

### Global transcriptional response to UV irradiation

**Figure 1:**
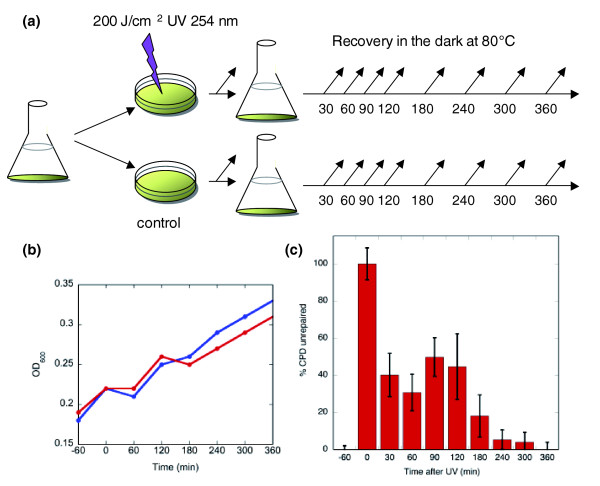
Experimental design and DNA damage. **(a) **Illustration of the experiment design. After growth to early log phase, cells were placed in Petri dishes and exposed to 200 J/m^2 ^UV irradiation, then allowed to recover in the dark at 80°C. Arrows indicate sampling time points. **(b) **Optical density measurement of *S. solfataricus *UV-treated (red) and control (blue) cultures. Cells were exposed to UV at time zero. **(c) **Generation and removal of CPDs in chromosomal DNA. DNA damage was measured by ELISA using a primary antibody specific for CPDs. Data were normalized so that the signal at time zero (immediately after UV irradiation) represented 100% unrepaired CPDs. Each data point represents the mean from six experimental replicates and error bars show the standard deviation of each data set.

To investigate the response of *S. solfataricus *and *S. acidocaldarius *to UV light induced DNA damage, cultures were exposed to 200 J/m^2 ^single wavelength (254 nm) UV light during early exponential growth (OD_600 _about 0.2, Figure 1a). At each time point indicated, samples were taken for mRNA and protein extraction, and for flow cytometry. Preliminary studies showed that higher UV fluency levels resulted in the death of the majority of the cells (data not shown). UV treatment was carried out at room temperature and the cultures were kept in the dark throughout the procedure to prevent photoreactivation. To compensate for effects related to temperature changes, control cultures were subjected to the same procedure except for the UV irradiation. The temperature shock and handling effects caused growth retardation in both the control and irradiated cultures, particularly in the first hour after treatment (Figure 1b). The appearance and clearance of DNA damage following UV irradiation was quantified using an antibody specific for cyclobutane pyrimidine dimers (CPDs). We observed biphasic kinetics where a majority of CPDs (50-60%) was repaired within 30 minutes and the rest were repaired at a slower pace, with 95% repair after 4 h (Figure 1c).

**Figure 2:**
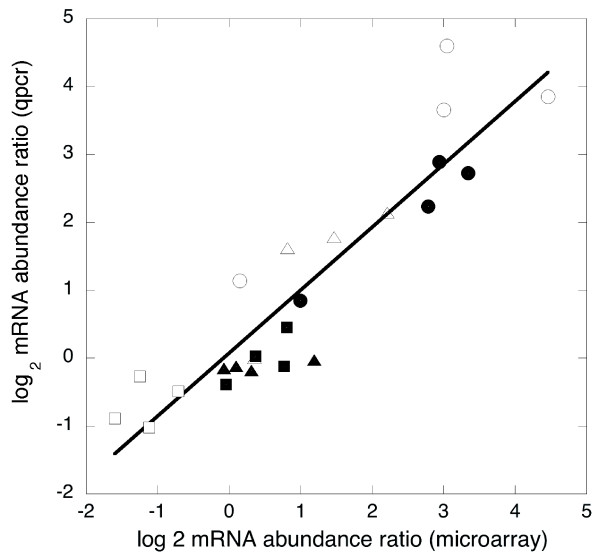
Comparison of transcript abundance quantified by microarray analysis and quantitative RT-PCR (qpcr). The changes in mRNA levels of six genes following UV irradiation were analyzed by quantitative RT-PCR to provide an independent confirmation of the microarray data. The log_2 _expression values for *ssb *(closed triangles), *cdc6-2 *(closed circles), *tfb1 *(closed squares), *tfb2 *(open squares), *tfb3 *(open circles) and *dpoII *(open triangles) obtained from microarray and RT-PCR analyses are plotted on the x and y axes, respectively. The data obtained from the two methods yield a linear fit with a slope of 0.93 and R-value of 0.91.

Overall, 163 *S. solfataricus *and 157 *S. acidocaldarius *genes showed a >2-fold change in transcript abundance (up or down) after UV treatment compared to controls. For both species more genes were up-regulated than down-regulated, although this difference was more pronounced in *S. acidocaldarius *than in *S. solfataricus*. This is contrary to findings for *Halobacterium *NRC-1, where most changes directly after UV treatment were in the form of down-regulation, mainly of metabolic genes [6]. The greatest changes in transcript levels occurred at 90 and 120 minutes. Expression ratios for selected genes following UV irradiation are summarized in Table 1, and Additional data files 1-6 provide comprehensive data for all *S. solfataricus *and *S. acidocaldarius *genes on the arrays. The transcriptional responses of selected genes were analyzed further by quantitative RT-PCR to provide an independent confirmation of the microarray data (Table 2). We observed a good correlation between the two methods (Figure 2), providing confidence that the microarray data reflected real changes in the relative abundance of mRNA levels in UV treated and control cells. In general, we observed a good agreement between the two species for both induced and repressed genes in all the gene categories analyzed, including genes encoding hypothetical proteins (Table 1). This reflects the shared evolutionary heritage and lifestyle of these two *Sulfolobus *species, which grow in very similar environments. It also suggests that the transcriptional effects observed are not artifactual. Where there are differences, for example, in the expression changes observed with Cdc6-2 and RadA described below, these may reflect real differences in the UV damage response of the two species.

**Table 1:** Microarray data for abundance of selected transcripts following UV irradiation

				UV/control mRNA abundance ratio							
				
				*S. solfataricus *(minutes after UV irradiation)				*S. acidocaldarius *(minutes after UV irradiation)			
					
Process	Sso no.	Sac no.	Description	30	60	90	120	30	60	90	120
DNA replication	0257	0722	Cdc6-1	0.99	0.84	0.37	0.53	1.11	0.74	0.69	0.65
	0771	0903	Cdc6-2	1.99	7.66	10.2	6.9	0.94	1.01	1.1	1.18
	2184	0001	Cdc6-3	0.93	0.9	0.91	0.68	0.75	1.09	0.83	0.59
	0772	0901	Gins23	1.01	0.57	0.67	0.55	1.08	0.79	0.71	0.78
	1049	1278	Gins15	1.19	0.88	-	-	0.97	0.93	0.70	0.64
	0774	0900	MCM	0.98	0.68	0.86	0.77	1.01	0.78	0.82	0.7
	1048	1279	Primase PriS	1.51	0.97	1.14	1.02	0.91	0.76	0.82	0.64
	0557	1542	Primase PriL	1.03	0.76	0.77	0.72	1.18	1.28	1.05	1.04
	0552	1537	DpoI	1.04	0.71	1.11	0.99	1.01	0.8	0.63	0.63
	1459	2156	DpoII	1.28	1.76	4.63	2.76	2.95	3.13	5.06	4.9
	8124		DpoII	1.52	2.14	2.86	1.95	-	-	-	-
	0081	0074	DpoIII	1.13	0.97	0.83	0.73	1.01	0.56	0.44	0.36
	2448	0554	DpoIV	1.28	1.01	-	-	1.01	1.08	0.88	0.89
	2498	1112	Rib red nrdB	1.14	1.73	2.62	2.47	1.47	1.03	1.10	0.94
	0929	1353	Rib red nrdJ	0.86	0.72	1.45	0.83	1.05	1.40	1.90	1.41
DNA repair (selected)	0729	0604	XPF	1.17	1.26	1.1	1.15	1.18	1.16	1.05	1.02
	0473	1657	XPB2	1.43	1.13	1.17	1.08	1.07	0.98	0.95	0.8
	0179	0775	Fen1	1.27	0.84	1.18	0.93	0.92	0.87	0.62	0.61
	0313	0192	XPD	1.44	1.18	1.28	1.43	0.98	0.73	0.95	0.93
	250	0715	RadA	1.03	0.97	1.14	0.87	0.87	1.71	1.05	2.29
	0575	1558	HJC	1.39	1.1	1.4	1.28	1.03	1.05	1.06	1.12
	2250	0052	Mre11	0.84	0.95	1.69	1.19	0.96	0.92	0.97	0.88
	2251	0053	herA	0.97	1.06	2.11	1.4	1.06	1.11	0.9	0.71
	0116	1497	EndoIII	1.08	1.17	1.28	1.45	-	-	-	-
	2156	0015	Endo IV	1.13	0.7	0.78	0.74	1.01	0.91	0.81	0.67
	2454	0544	EndoV	1.41	1.12	1.03	0.91	1.11	1.15	1.44	1.65
	0904	1367	OGG1	1.0	1.01	1.12	0.92	0.88	1.3	1.13	1.21
	2487	1260	OGT	1.31	1.19	1.35	1.6	1.17	1.01	1.15	0.95
	2275	0159	UDGaseIV	1.24	0.86	1.03	0.83	1.08	0.75	0.88	0.89
	2484	1747	UDGaseVI	1.15	0.85	0.94	1.07	1.11	1.4	1.66	1.55
Chromatin	0962	1321	Alba1	0.87	1.11	0.73	0.95	0.91	0.96	0.91	1.0
	6877	1322	Alba2	1.04	0.85	0.99	0.8	0.86	1.19	0.82	1.66
	9180	0064	SSO7d	0.49	0.57	0.35	0.6	-	-	-	-
	9535	0362	SSO7d	0.52	0.52	0.36	0.61	-	-	-	-
	0420	0839	Rev gyrase rgy1	1.43	1.09	1.0	0.8	0.99	0.91	0.66	0.55
	0963	*	Rev gyrase rgy2	0.95	0.81	0.98	0.76	1.37	0.99	0.58	0.53
	0969	1314	Topo6A	0.95	0.8	1.31	1.25	0.91	0.99	0.89	0.79
	0968	1315	Topo 6B	1.12	0.67	2.25	1.7	1.02	0.72	0.64	0.53
	0907	1371	Topo 1	1.16	1.0	1.37	1.09	1.04	1.48	3.56	4.1
	2478	0381	Sir2	1.13	1.2	1.34	1.77	1.16	1.37	1.62	1.52
	2813	2284	Pat	1.17	0.93	1.07	1.14	0.97	1.0	1.38	1.55
Transcription	0280	0665	TFB3	1.11	2.82	8.26	8.03	2.32	4.08	7.05	6.95
	0946	1341	TFB2	0.61	0.33	0.42	0.46	1.04	0.83	0.4	0.4
	0446	0866	TFB1	0.97	1.29	1.75	1.7	0.94	1.29	1.06	1.12
	0291	0171	TFS	1.08	1.45	1.01	1.16	-	-	-	-
Oxidative stress	2078	1820	Nramp	1.42	3.03	4.55	4.76	1.02	1.58	4.79	6.57
	2079	1821	Dps	0.95	1.69	4.67	5.6	1.03	1.6	4.53	6.47
	2080	1822	Rieske	0.96	1.08	1.68	1.69	1.17	1.32	4.63	5.46
	1503	1170	MsrA	0.82	1.22	1.28	1.46	2.02	1.97	2.27	1.96
Early induced	2364	0975	SSB	2.28	1.24	1.07	0.95	1.81	1.91	0.82	0.98
	2374	0965	RIO kinase	2.81	1.05	1.00	1.08	1.41	1.04	0.84	0.85
	1127	0334	HdrC-1	2.66	1.76	1.84	1.78	1.60	2.02	2.30	2.04
	1129	0329	HdrB-1	2.65	1.32	1.57	1.28	1.55	1.83	1.77	1.76
	1131	0328	HdrA	2.23	1.71	2.17	2.04	1.66	1.56	1.95	1.59
	1134	0326	HdrC-2	2.13	1.06	1.36	1.12	1.08	1.26	0.82	1.25
	1135	0325	HdrB-2	2.39	1.36	1.25	1.31	0.98	1.33	1.57	2.21
	3201	1202	Sulfite oxidase	2.27	1.10	0.80	0.84	1.36	1.21	1.02	0.76
	2261	0331	Sulfide oxidored	7.64	1.57	1.33	1.15	0.87	1.25	1.17	1.15
	2905	0821	CrtB	2.57	1.66	1.17	1.26	1.17	1.10	0.85	0.95
	2906	0822	CrtZ	2.31	1.57	1.13	0.96	-	-	-	-
Hypothetical	2395	0951		0.98	1.73	10.0	12.8	0.95	2.95	9.83	10.8
	0691	0568		1.28	3.02	9.76	12.5	-	-	-	-
	3146	0568		0.27	1.13	2.86	3.50	-	-	-	-
	0037	1302		1.30	1.37	6.94	6.99	0.94	2.53	1.48	7.48
	0283	0667		1.42	1.40	4.98	4.67	1.18	2.65	3.87	2.46

Data are given as ratio of mRNA abundance of UV-treated over control cultures. Sso and Sac numbers represent the gene numbers from the corresponding genomes. Dashes indicate missing gene on array, or instances where data were excluded for technical reasons. *The gene for Sac rgy2 has not been properly annotated in the genome but is covered by the array.

**Table 2:** Summary of data from quantitative RT-PCR on the *S. solfataricus *genes *tfb3*, *cdc6-2*, *xpb1*, *dpoII *and *ssb*

		CP*		^†^UV/control expression ratio			
			
Protein	Sso no.	UV (120 min)	Control (120 min)	30 min	60 min	90 min	120 min
TFB1	0446	14.7 ± 0.21	14.5 ± 0.06	0.76	1.02	1.37	0.92
TFB2	0946	21.4 ± 0.21	20.2 ± 0.15	0.71	0.54	0.83	0.49
TFB3	0280	12.5 ± 0.06	16.3 ± 0.06	2.2	14.4	24.2	12.6
Cdc6-2	0771	12.7 ± 0.06	15.8 ± 0	1.8	7.4	6.6	4.7
XPB1	0959	13.8 ± 0.1	13.6 ± 0.12	0.80	1.07	1.05	0.92
DpoII	1459	11.1 ± 0.12	13.7 ± 0.06	1.0	3.07	4.41	3.44
SSB	2364	9.9 ± 0.12	9.8 ± 0.23	0.98	0.88	0.92	0.90

*CP: the cycle number at which the fluorescence crossed the threshold during PCR. The lower the CP, the more abundant the transcript. CP values are means of triplicate experiments with standard deviations shown. ^†^The expression ratio was calculated according to the method of [50].

### Early responses to UV irradiation

The initial response to UV irradiation must involve the generation of signals within the cell, most obviously DNA damage due to photoproducts and strand breaks, and increased concentrations of ROS. Signal transduction by as-yet unknown mechanisms led to a transcriptional response. In these experiments our earliest time point was taken at 30 minutes post-UV irradiation. It is clear that a transcriptional response is already well underway at this early time point. A number of genes show high levels of induction at 30 minutes. Single-stranded DNA binding proteins (SSBs) in bacteria and eukarya are known to play an important role in the early detection of DNA damage, and the *S. solfataricus *SSB has been shown to detect UV photoproducts *in vitro *[11]. Our microarray data show two-fold up-regulation of the *ssb *gene (*sso2364*, *saci0975*) at 30 minutes after UV treatment for *S. solfataricus *and at 30 and 60 minutes for *S. acidocaldarius*, but this mild induction was not apparent from quantitative RT-PCR (Table 2) and not obviously reflected at the protein level (see later). Protein kinases play an essential role in the mediation of the DNA damage response in eukarya. The gene encoding the atypical Ser-/Thr-RIO protein kinase-1 is induced at early time points in both *S. solfataricus *(*sso*2374) and *S. acidocaldarius *(*saci*_0965). The biological targets of RIO kinases are not yet known, but they have been implicated in cell cycle progression and chromosomal maintenance in yeast [12]. It will be interesting to determine whether the archaeal RIO kinase mediates protein phosphorylation in response to DNA damage, analogous to the ATM/ATR damage response pathway in eukarya. The genes for beta carotene biosynthesis *crtB *and *crtZ *(*sso*2905 and *sso*2906; *saci_*1733 and *saci_*1734) are strongly induced at the 30 minutes time point. Production of beta carotene has previously been observed in response to UV irradiation in *S. acidocaldarius *[13], and the pigment has a protective effect against UV light. Thus, *Sulfolobus *cells apparently synthesize protective pigments in response to damaging levels of UV light.

There are also early transcriptional responses in genes encoding a variety of metabolic pathways. Five genes encoding subunits of a putative heterodisulfide reductase (Hdr), arranged in an operon in *both Sulfolobus species *(e.g. *sso*1127-35), are strongly induced following UV irradiation. The function of this multienzyme complex is important for methanogenesis in methane-producing archaea, but is not understood in organisms such as *Archaeoglobus fulgidus *and *Sulfolobus *species, which do not generate methane. One suggestion is that these enzymes function in an electron transport chain that oxidizes menaquinol and passes electrons to a sulfate reduction pathway [14]. Another induced enzyme with a related function is the flavin adenine dinucleotide (FAD)-dependent sulfide:quinone oxidoreductase (SQR), which oxidizes sulfide and passes electrons into the respiratory chain, and is essential for chemolithoautotrophic growth using hydrogen sulfide as an electron donor [15]. The gene encoding this protein is induced in both *S. solfataricus *(*sso*2261) and *S. acidocaldarius *(*saci*_0331), and it is notable that the latter is located on the genome next to the *S. acidocaldarius hdr *genes (*saci*_0334-0339). Two genes in the riboflavin biosynthetic pathway (*sso*0401, *sso*0402; *saci*_0821, *saci*_0822) are also induced at early time points. Riboflavin is a precursor of FAD and flavin mononucleotide (FMN) [16]. Given the observation that many FAD-dependent oxidoreductases are also induced, this is consistent with an increased requirement for the cofactor following UV irradiation.

### DNA replication proteins are repressed following UV irradiation

Both eukarya and bacteria have mechanisms to inhibit DNA replication and cell division following DNA damage to allow DNA repair to take place, and a similar response has been observed in the euryarchaeon *Pyrococcus abyssi *following ionizing irradiation [7]. Genes encoding DNA replication proteins were amongst the most highly repressed following UV irradiation in both *S. solfataricus *and *S. acidocaldarius*. We observed significant down-regulation of the genes for the replication initiation proteins Cdc6-1 and Cdc6-3 [17], MCM helicase, and Gins proteins [18] (Table 1).

**Figure 3:**
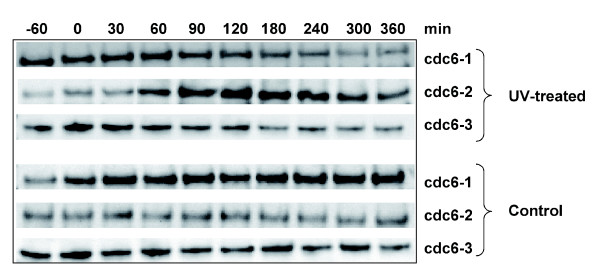
Cdc6-2 protein levels increase following UV irradiation. Western blot using antibodies against *S. solfataricus *Cdc6-1, Cdc6-2 and Cdc6-3. Cell-free extracts were prepared from samples taken from UV-treated and control cultures at the indicated time points. In agreement with the microarray data, the levels of Cdc6-2 protein show a clear increase after UV irradiation, whereas those of Cdc6-1 and Cdc6-3 are reduced.

In contrast with the repression of *cdc6-1 *and *cdc6-3*, strong up-regulation of *cdc6-2 *was evident in *S. solfataricus*. *cdc6-1 *and *cdc6-3 *expression levels were also reduced in *S. acidocaldarius*, whilst *cdc6-2 *levels were unchanged. Thus, in both organisms the levels of Cdc-6 protein are likely to be increased compared to Cdc6-1 and Cdc6-3 following UV irradiation. The increase in *S. solfataricus cdc6-2 *transcription was confirmed by quantitative RT-PCR (Table 2), and western blotting with antibodies against all three Cdc6 proteins showed that the transcriptional changes were reflected in the expression levels of each of the proteins (Figure 3). At 1 h after UV treatment there was a clear increase in the amount of Cdc6-2, which persisted until 4 h post-treatment before declining again. Cdc6 proteins are AAA+ type ATPases that bind to replication origins and interact with the replicative helicase MCM [19]. The role of Cdc6-2 in DNA replication is not completely clear, but elevated levels are found in stationary phase cultures and during the post-replicative cell cycle stage, that is, in non-replicating cells [17]. Also, Cdc6-2 can bind at origins at sites overlapping the binding sites for Cdc6-1 and Cdc6-3. These observations have prompted the suggestion that Cdc6-1 and Cdc6-3 act in a positive fashion to promote replication initiation, while Cdc6-2 may act as a repressor [17]. The differential transcriptional control of *cdc6-2 *from *cdc6-1 *and *cdc6-3 *following UV DNA damage therefore suggests a mechanism by which initiation of DNA replication could be repressed, giving cells time to carry out DNA repair.

*Sulfolobus *species encode two to three paralogues of the 7 kDa chromatin protein Sul7 (Sso7 or Sac7) [20]. Previous reports showed a considerable decrease of *sso7 *gene transcripts in UV-damaged *S. solfataricus *cultures [8]. In agreement with these studies, the two *sso7 *genes (*sso9180*, *sso9535*) on the microarrays showed substantial down-regulation in the microarray analyses, placing them amongst the most strongly repressed genes observed in this study. Sso7 makes up about 5% of the total soluble cell protein and is one of the 2 main chromatin proteins present in *Sulfolobus *species [20]. A reduction of *sso7 *transcript levels to one-third compared to those in control cells may reflect the repression of DNA replication, as chromatin proteins are needed in proportion to the amount of newly synthesized DNA. In contrast, the genes encoding the chromatin proteins Alba1 and Alba2 and Alba1 acetylase Pat (*sso2813*) [21] showed little change in expression following UV irradiation; however, the transcript abundance of the Alba1 deacetylase Sir2 (*sso2478*) did increase in both organisms, raising the possibility that Alba1 acetylation levels are reduced in response to DNA damage.

**Figure 4:**
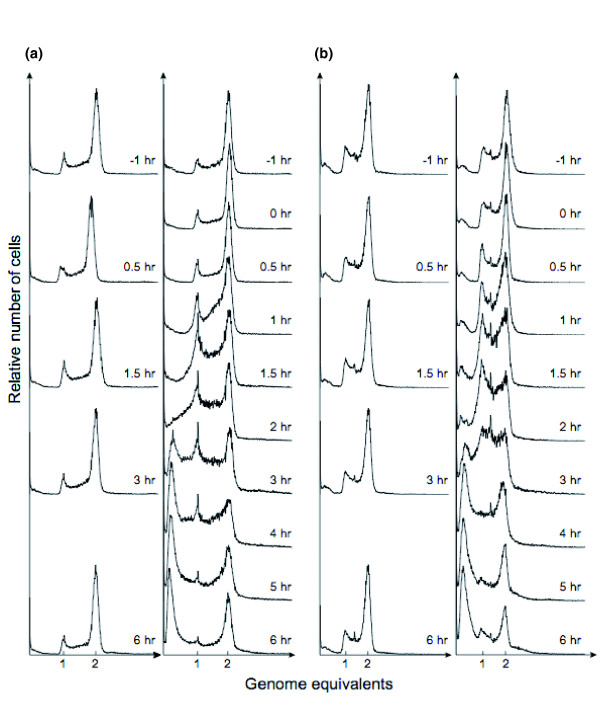
Flow cytometry DNA content distributions. Flow cytometry DNA content distributions for **(a) ***S. solfataricus *and **(b) ***S. acidocaldarius *from untreated control cultures (columns 1 and 3) and UV-irradiated cultures (columns 2 and 4) at different time points after treatment. The minor peak at a DNA content corresponding to 1.5 chromosome equivalents in the *S. acidocaldarius *distributions (columns 3 and 4) is a software artifact.

We also assessed DNA content and cell size of control and UV-treated cultures by flow cytometry. The *Sulfolobus *cell cycle is dominated by the post-replicative phase [22] and, during exponential growth, a majority of the population contains two chromosomes, while most of the remaining cells are in the replicative stage, and only a small proportion (≤5%) contain a single chromosome. The control cultures displayed typical DNA content distributions corresponding to this cell cycle organization at all time points, as did the UV-irradiated cultures at early time points after treatment (Figure 4). In the UV-treated cultures, the number of cells that contained a single chromosome equivalent gradually increased in relative abundance with time. In parallel, cells with a DNA content of more than one chromosome equivalent started to appear, eventually forming a continuous distribution that merged with the leftmost peak, which corresponds to DNA-less cells and background debris. Also, an increased proportion of cells with a DNA content between one and two chromosome equivalents became apparent between 1 h and 5 h after UV irradiation. The increased frequency of cells containing a single chromosome could be due to inhibition of replication initiation, in accordance with the changes in the expression patterns of the *cdc6 *genes (above), although the extensive DNA degradation indicates that chromosome breakdown also contributed to the relative increase in one-chromosome cells.

At 5-6 h after treatment, the DNA content distributions began to resemble those of the untreated control cultures, in addition to a large peak corresponding to DNA-less cells. Thus, the UV irradiation resulted in chromosome breakdown and generation of DNA-less cells in part of the cell cultures, while the remaining population recovered and eventually restored the typical DNA content distribution of an exponentially growing culture, in accordance with the continued increase in optical density (Figure 1b). No change in the flow cytometry light scatter distributions was observed in either culture during the time period analyzed (data not shown), indicating that any effects on cell size or cell integrity were below the detection limit.

In addition to the major replicative DNA polymerase DpoI [23], the *S. solfataricus *genome encodes three other DNA polymerases: DpoII, DpoIII and DpoIV [24,25]. The latter is a member of the DinB family of lesion bypass polymerases, and is specialized for the bypass of UV photoproducts [26], whilst DpoII and III have not been studied biochemically and their functions *in vivo *are unknown. Of the four polymerase genes, only *dpoII *was induced on the microarray following UV damage (Table 1), a result confirmed by quantitative RT-PCR (Table 2). The gene is annotated as two open reading frames (ORFs; *sso8124 *and *sso1459*) in the *S. solfataricus *genome due to a frameshift at position 75 in the gene, and both are up-regulated significantly. This may be a sequencing error, as the intact gene has been sequenced previously from the same strain of *S. solfataricus *[27]. The most closely related polymerase in the crenarchaeon *Pyrobaculum aerophilum*, PolB3, has been shown to possess DNA polymerase activity [28], but the two proteins share only 27% sequence identity and the *Sulfolobus *enzyme lacks the clear amino-terminal exonuclease domain of PolB3. The observation that the *Sulfolobus dpoII *gene is induced after UV irradiation whilst the replication machinery is repressed is suggestive of a role for the enzyme in the repair of DNA damage. Although most DNA repair enzymes are expressed constitutively in *Sulfolobus *(see below), it may be advantageous to control the expression of DpoII so that it does not interfere with DNA replication during undisturbed growth. Clearly the role of this enzyme deserves further study.

### Basal transcription apparatus

The archaeal basal transcription machinery, consisting in essence of a 12-subunit RNA polymerase holoenzyme and the transcription initiation proteins TBP (TATA binding protein) and TFB (transcription factor 2B homolog), represents a minimal version of the eukaryal transcription apparatus [29]. TBP and TFB bind at archaeal promoters, recruiting RNA polymerase to initiate transcription, and no other factors are required *in vitro *[30]. Following UV irradiation, we observed no significant changes in the transcript levels of TBP, nor any of the RNA polymerase subunits. Interestingly, however, there was a big change in transcription levels for the homologues of the basal transcription factor TFB in both species after UV-treatment. TFB comprises three domains; the carboxy-terminal core domain interacts with TBP and binds the BRE (the TFB responsive element) next to the TATA box using a helix-turn-helix (HTH) motif. The amino-terminal Zn ribbon domain, conserved in all eukaryal and archaeal TFB proteins, binds to RNA polymerase. A construct consisting of the Zn ribbon domain alone can inhibit transcription initiation *in vitro *by competing with TFB for recruitment of RNA polymerase. The B-finger domain, an extended region of the protein between the Zn ribbon and core domains, interacts with RNA polymerase and stimulates transcription initiation [31].

**Figure 5:**
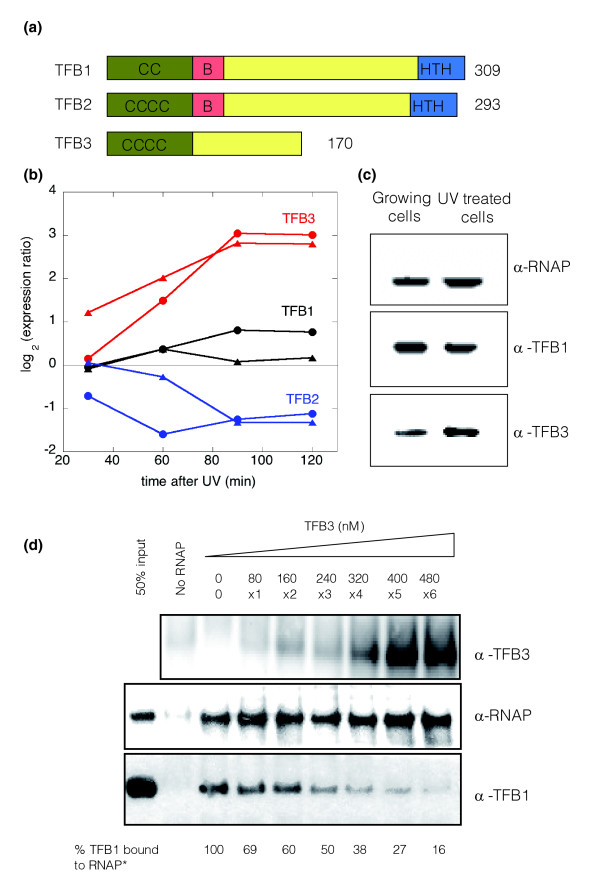
Differential effects of UV irradiation on expression of *Sulfolobus tfb *genes. **(a) **Both *S. solfataricus *and *S. acidocaldarius *encode three homologues of the basal transcription factor TFB. TFB1 and TFB2 are full-length orthologues, but TFB3 is severely truncated, lacking the B-finger domain (B) for transcription initiation and the HTH domain for binding to the BRE. C's represent the cysteines implicated in zinc binding **(b) **In both *S. solfataricus *and *S. acidocaldarius*, UV irradiation causes a dramatic increase in transcription of *tfb3 *and a significant reduction in expression of *tfb2*, whilst *tfb1 *levels are unaffected. Ratios of gene expression for the UV treated and control cultures are expressed in a log base 2 format, so that a value of 1 represents a two-fold increase in transcription. *S. acidocaldarius *and *S. solfataricus *data points are represented by triangles and circles, respectively. **(c) **Immunodetection of endogenous RNA polymerase (RNAP), TFB1 and TFB3 from cell lysates from control and UV-treated cells. Cells were harvested and lysed after 90 minutes of treatment. Levels of TFB3 protein increase after UV treatment in agreement with microarray data and quantitative RT-PCR (Table 2). **(d) **SDS-PAGE and western blot of immunoprecipitation of RNAP using specific antibodies (B' subunit) and co-immunoprecipitation of bound TFB1 and TFB3. Increasing concentrations of TFB3 (0-480 nM) were added to a mix with constant concentrations of RNAP and TFB1 (40 nM:80 nM). In the control lane where RNAP was not added, concentrations of TFB1 and TFB3 were 80 nM and 480 nM, respectively. The molar ratios of TFB3 and TFB1 are indicated below the TFB3 concentrations. Relative amounts of TFB1 bound to RNAP in each sample are indicated, showing a decrease of TFB1 bound to RNAP as TFB3 increases. All values are normalized with the total amount of RNAP immunoprecipitated in each sample.

The genomes of all three *Sulfolobus *species sequenced contain three homologues of the *tfb *gene (Figure 5a). Two of them (*tfb1 *and *tfb2*) encode full length TFB proteins, and TFB1 has been shown to support transcription initiation *in vitro *[30]. The third homologue (*sso*0280 in *S. solfataricus*) is significantly shorter than the other two. The sequence includes the Zn ribbon but lacks the B-finger domain and has an abbreviated core domain (Figure 5a). Under normal growth conditions in the absence of UV irradiation, the CP (crossing point: the cycle number at which the PCR product reaches a set threshold, a number that is lower for more abundant transcripts) values obtained from quantitative RT-PCR indicated that *tfb1 *is the most abundant transcript of the three, with *tfb2 *expressed at a low level (Table 2). Following UV irradiation, however, the relative abundances of these three transcripts changed significantly (Tables 1 and 2). Whilst there was a significant down-regulation of *tfb2 *(*sso0946*) and no significant change in the transcription levels of *tfb1 *(*sso0446*), *tfb3 *was one of the most highly up-regulated transcripts in response to UV irradiation in both *Sulfolobus *species (Figure 5b), and this was reflected in the increased concentrations of the TFB3 protein in UV-irradiated cells (Figure 5c). The radically altered organization of TFB3 suggests a role other than transcription initiation in *Sulfolobus *species. As it retains the Zn ribbon domain for interaction with RNA polymerase, TFB3 may act as a competitive inhibitor of transcription initiation, as has been noted for the Zn ribbon domain in isolation [32]. This was confirmed *in vitro *using the purified native RNA polymerase and recombinant TFB1 and TFB3 proteins from *S. solfataricus *(Figure 5d). In the absence of TFB3, TFB1 interacts with RNA polymerase, and can be pulled-down with antibodies raised against the B' subunit of RNA polymerase. However, increasing concentrations of TFB3 abolish the interaction between RNA polymerase and TFB1, consistent with the model proposed above. TFB3 is unlikely to interact with the BRE, as the conserved carboxy-terminal HTH motif that makes this contact is absent. The dramatic increase in *tfb3 *transcript abundance following DNA damage may provide a mechanism to modulate transcription from certain promoters.

### DNA repair proteins are not induced by UV irradiation

**Figure 6:**
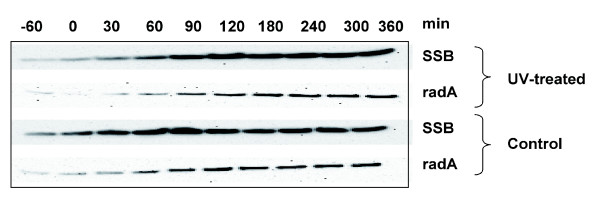
The repair proteins RadA and SSB are not strongly induced by UV irradiation. Western blot using antibodies against *S. solfataricus *RadA and SSB. Cell-free extracts were prepared from samples taken from UV-treated and control cultures at the indicated time points. In agreement with the microarray data, RadA levels do not increase significantly following UV irradiation. The levels of SSB protein are also largely unchanged, in agreement with quantitative RT-PCR (Table 2), though the microarray data showed a modest rise in SSB transcription at early time points after UV damage.

Transcription and DNA repair are linked intimately, both through transcriptional responses to DNA damage and by the fact that many DNA lesions are repaired following an encounter with a transcribing RNA polymerase molecule (transcription coupled repair). In most bacteria, many DNA repair proteins are under the control of the LexA repressor [33]. Archaea lack LexA homologues and, therefore, a classical SOS regulon, but an SOS-type response is still possible, even if not mediated by LexA. RadA (the archaeal RecA homolog) is up-regulated in response to UV damage in *Halobacterium *NRC-1; moderately in one study [6] and by up to seven-fold in another [34], and moderately up-regulated in response to ionizing radiation in the hyperthermophile *Pyrococcus furiosus *[35]. Our data showed no increase in *radA *transcript (Table 1) or protein levels (Figure 6) in *S. solfataricus*. In *S. acidocaldarius*, however, we observed a two-fold up-regulation of *radA *at 2 h.

UV irradiation causes a broad spectrum of DNA lesions, including photoproducts (predominantly CPDs and 6-4 photoproducts), oxidative damage to bases and single-strand DNA breaks. Despite the clear indications that *Sulfolobus *species have a transcriptional response to DNA damage, including induction of *cdc6-2 *and *tfb3*, we found no significant changes in transcription of known DNA repair proteins (Table 1). The experiments reported here were carried out in the absence of light, and no up-regulation of the photolyase gene was observed. Likewise, the lesion bypass DNA polymerase DpoIV, specialized for replication past UV photoproducts [26], was not induced.*S. acidocaldarius *possesses a gene encoding a UV damage endonuclease (*saci1096*) of the UVDE family [36], which is UV inducible in *Schizosaccharomyces pombe *following UV irradiation [37]. The *S. acidocaldarius *gene was only mildly induced after UV irradiation (Table 1).

In addition, none of the putative archaeal nucleotide excision repair proteins (XPB1, XPB2, XPD, XPF, Fen1, SSB) showed significant induction following UV irradiation. The data for *xpb1 *contrasts with an earlier study that reported a significant increase in *xpb1 *transcription following UV irradiation [8]. We therefore checked transcription of the *xpb1 *gene using quantitative PCR, which confirmed that no induction occurred (Table 2). However, the experimental conditions were not identical in our experiments and those of Salerno *et al*. [8], as in the latter a higher UV flux was used and transcript levels were monitored over a longer time period. Genes encoding enzymes involved in the pathway of base excision repair, which are required for the removal of bases damaged by UV light, were also not up-regulated significantly, and the same was true for the double-strand break repair proteins required for homologous recombination and the rescue of stalled replication forks (Table 1).

### Oxidative stress

In addition to the generation of photoproducts, UV irradiation results in the generation of ROS that cause a broad spectrum of DNA damage. We observed significant up-regulation of a cluster of ORFs in both species (*sso2078-2080*, *saci1820-1823*) with probable roles in the detoxification of ROS. One of the ORFs (*sso2079*, *saci1821*) codes for the *Sulfolobus *Dps protein. Dps is expressed under conditions of oxidative stress, when it forms a cage-like structure, binds to DNA and catalyzes the oxidation of reactive Fe(II) to Fe(III) ions, preventing the generation of damaging ROS via Fenton chemistry [38]. The *dps *gene is also up-regulated following UV irradiation in *Halobacterium *NRC1 [6] and gamma irradiation of *P. furiosus *[35]. The other members of this cluster are a homologue for a NRAMP protein [39] with a potential role in the transport of Fe^2+/3+ ^and Mn^2+ ^(*sso2078*, *saci1820*) and a ferritin homologue implicated in the storage of Fe^2+/3+ ^(*sso2080*, *saci1822*). The gene encoding peptide methionine sulfoxide reductase (MsrA) is also induced at early time points in both species (*sso1503*, *saci_1170*). MsrA reverses oxidative damage to methionine side chains in proteins and, therefore, protects the cell from oxidative stress and ROS. These gene products are thus likely to provide a front-line defense against DNA and protein damage caused by ROS generated as a consequence of UV irradiation.

## Discussion

**Figure 7:**
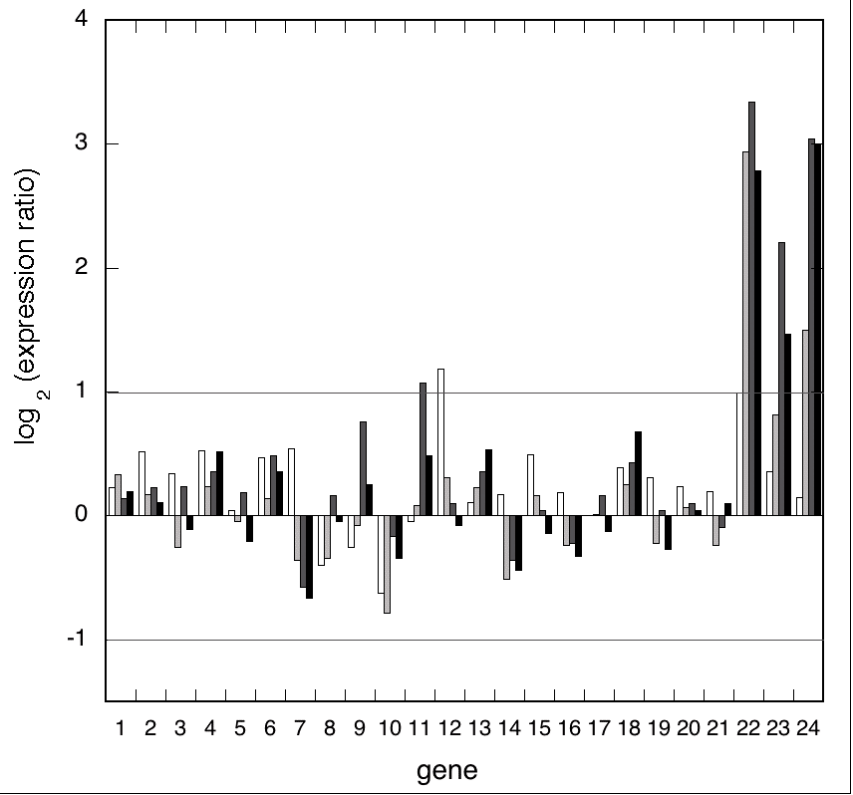
Transcription of DNA repair genes is not induced significantly by UV irradiation. Known DNA repair genes are grouped by function, with time points for 30 minutes (white), 60 minutes (light grey), 90 minutes (dark grey) and 120 minutes (black) after irradiation shown. Ratios of gene expression for the UV-treated and control cultures are expressed in a log base 2 format, so that a value of 1 represents a two-fold increase in transcription and a value of -1 represents a two-fold decrease (indicated by horizontal grey lines). Few data points show an induction greater than two-fold. By contrast, lanes 22-24 show the highly induced genes *cdc6-2*, *dpoII *and *tfb3*, respectively. Repair genes are all from *S. solfataricus *(Table 1): 1, *xpf*; 2, *xpb2*; 3, *fen1*; 4, *xpd*; 5, *radA*; 6, *hjc*; 7, *hje*; 8, *rad50*; 9, *mre11*; 10, *nurA*; 11, *herA*; 12, *ssb*; 13, *endoIII*; 14, *endoIV*; 15, *endoV*; 16, *mutT*; 17, *ogg1*; 18, *ogt*; 19, *udgaseIVa*; 20, *udgaseIVb*; 21, *udgaseVI*. Data for *S. acidocaldarius *repair genes are qualitatively similar (Table 1).

The data presented here allow a number of conclusions to be drawn and several hypotheses to be postulated. Firstly, it is striking that no DNA repair proteins in either *Sulfolobus *species were induced to a significant level following UV irradiation (Table 1, Figure 7). Further support comes from limited data from other hyperthermophilic archaea that, for example, the RadA protein is expressed constitutively and only moderately induced by DNA damage. Hyperthermophiles living at elevated temperatures inevitably suffer a high level of DNA damage under normal growth conditions, and repair proteins may be present in sufficient amounts during most, or all, of the cell cycle to enable the organisms to deal efficiently with frequent damage. Interestingly, RadA and some other DNA repair proteins do show a periodic pattern of expression during the cell cycle, suggesting that there is some level of transcriptional control at this level [23]. A coordinated SOS-response to DNA damage appears to be a phenomenon restricted to the bacteria, as DNA repair proteins in *S. cerevisiae *and higher eukaryotes are only mildly induced by DNA damage [40].

Although there may be no requirement to induce DNA repair genes following UV irradiation in *Sulfolobus *species, a clear transcriptional response was observed. Particularly striking was the repression of transcription of a number of DNA replication and chromatin proteins. When faced with elevated levels of DNA damage, bacterial and eukaryal organisms slow down, or completely inhibit, DNA replication and cell division. Many types of DNA lesion are exacerbated by DNA replication, for example uracil arising from cytosine deamination becomes fixed as a mutation only when replicated, and single-strand breaks are converted to more dangerous double-strand breaks by the passage of the replication fork. Since the Cdc6-2 protein is believed to act as a repressor of initiation of chromosome replication [17], the increase in the relative levels of the *cdc6-2 *transcript after UV irradiation is consistent with inhibition of DNA replication in response to DNA damage in *Sulfolobus *species. This is also in line with the observation that *cdc6-1 *and *cdc6-3 *transcription is induced at the G_1_/S transition during the *S. acidocaldarius *cell cycle, while *cdc6-2 *induction takes place just before entry into G_2_, when no further replication should occur [23].

Whilst the downstream effects of DNA damage on processes such as DNA replication have been demonstrated, there are important questions regarding the mechanisms by which the 'signal' of DNA damage is transduced to achieve these responses. The SOS system in bacteria provides a good example, as a RecA filament formed in response to DNA damage stimulates auto-proteolysis of the LexA repressor, leading to de-repression of a coordinately regulated set of genes. In eukaryotes, damage detection by RPA, the Mre11-Rad50-Nbs1 complex and other proteins initiate cascades of protein phosphorylation that activate DNA repair and cell cycle checkpoints (reviewed in [41,42]). We still do not know the primary DNA damage signal in archaea, though the single-stranded DNA binding protein SSB, which can bind specifically to damaged DNA and interacts with RNA polymerase [43] is a candidate. The transient early induction of transcription of the gene encoding SSB may, therefore, be significant for the DNA damage response. The early induction of the gene encoding a RIO-type protein kinase also warrants further investigation.

The striking differences in the expression of the three *Sulfolobus *TFB homologues after UV irradiation may provide a partial explanation for the transcriptional effects observed. It is possible that DNA replication genes are dependent on the TFB2 protein for transcription, and are thus repressed as levels of TFB2 fall after DNA damage. This is consistent with the observed induction of the *S. acidocaldarius tfb2 *transcript prior to the G_1_/S transition of the cell cycle [23]. In this scenario, the bulk of genes, which are not affected by UV irradiation, might be transcribed using TFB1, whose level remains constant. TFB3, which was highly induced following DNA damage, could conceivably be responsible for the increase in transcription levels observed for genes such as *cdc6-2 *and *dpoII*. However, the structure of TFB3 appears more consistent with a role in the repression of transcription, perhaps by competing with TFB1 and TFB2 by sequestering RNA polymerase molecules. We have confirmed that TFB1 and TFB3 compete for interaction with RNA polymerase *in vitro*. In both hypotheses, novel regulatory circuits, now open for experimental investigation, form an integrated part of the response mechanism.

Finally, a number of genes encoding proteins of unknown function are significantly up- or down-regulated following UV irradiation (Table 1 and Additional data files 1-6). Some of these may represent archaeal-specific DNA damage response proteins with potential roles in DNA replication, transcription or repair. For example, two of the mostly highly up-regulated genes in *S. solfataricus*, *sso0691 *and *sso3146*, are distant from one another on the chromosome but encode hypothetical proteins with 43% identity to one another at the amino acid level. This family is specific to crenarchaea and the protein is predicted to have seven transmembrane helices, suggesting a role in membrane transport or interaction with the environment. The operon *sso0117-0121 *is highly induced by UV radiation in *S. solfataricus*, as assessed by microarray and quantitative RT-PCR [10]. This operon is annotated as a pilus assembly system in related archaea [44], which may be consistent with the increase in intercell DNA transfer by conjugation observed on UV radiation of *S. acidocaldarius *cells [45]. The homologous pairs *sso2395*/*saci0951 *and *sso0037*/*saci1302 *are both highly induced following UV irradiation. These genes have no known function and are found only in *Sulfolobus *genomes. Induction in both species suggests a potential role in the cellular response to UV irradiation for these proteins.

## Conclusion

In conclusion, we have presented one of the first reported large-scale studies of the DNA damage response in hyperthermophilic crenarchaea. An independent study by Schleper and colleagues [46] reports similar results, despite many differences in experimental design. Confidence is also derived from the good correlation observed between the microarray data, quantitative RT-PCR and western blotting for selected targets. Finally, the comparison of two related species of *Sulfolobus *provides a further filter on the significance of the data reported here. For many genes, there is good qualitative agreement in expression changes observed in the two species following UV damage. Where there are differences, for example in the mild induction of RadA in *S. acidocaldarius *but not *S. solfataricus*, these may reflect genuine differences in the biology of the two organisms, which have significantly different genome sizes and content. *S. acidocaldarius *has about 1,000 less genes than *S. solfataricus *(2,292 versus 3,217), and about 300 unique genes [36].

The data allow the conclusion that the crenarchaea do have a programmed transcriptional response to DNA damage caused by UV irradiation. The expression of DNA replication proteins is strongly repressed, and the differential expression of the Cdc6-2 protein compared to Cdc6-1 and Cdc6-3 observed by others is also in evidence here. Changes in transcription patterns following DNA damage may be mediated partly by altered expression levels of the three TFB proteins encoded by *Sulfolobus *species, and we provide evidence that the highly induced TFB3 protein can compete with TFB1 for binding to RNA polymerase, thus providing a potential mechanistic basis for these effects. Several hypothetical proteins induced by UV in both studies hint at novel mechanisms for the DNA damage response in *Sulfolobus *species. Finally, we conclude that the genes encoding DNA repair proteins are not strongly induced by UV radiation, suggesting that the lifestyle of hyperthermophiles such as *Sulfolobus *leads to elevated levels of basal DNA damage and hence a capacity for constitutive DNA repair pathways.

## Materials and methods

### UV treatment and sampling

*S. solfataricus *P2 (DSMZ 1617) and *S. acidocaldarius *(DSMZ 639) were grown aerobically at 80°C to early exponential phase (OD_600 _approx. 0.18). Exposure to 200 J/m^2 ^UV light (254 nm) was carried out using a Stratalinker^® ^UV crosslinker 1800 (Stratagene, La Jolla, CA, USA). Cultures were irradiated in 25 ml volumes in standard pre-warmed Petri dishes to ensure UV light exposure of all cells, and returned to the incubator immediately afterwards. Total handling and UV exposure time totaled less than 5 minutes, and culture temperatures did not drop below 55°C. An earlier study showed that this dose of UV results in an average of 1 CPD per kilobase of DNA for *S. solfataricus *[8]. Control cultures were treated in the same way except for the UV irradiation. Samples for flow cytometry and western blots were taken at various times 1 h before and up to 6 h after UV exposure. Samples for RNA extraction were taken at 30, 60, 90 and 120 minutes after UV treatment, and RNA was extracted immediately.

### RNA extraction

For RNA extraction, samples were centrifuged at 4,000 rpm for 10 minutes at 4°C and the cell pellet was re-suspended in 1 ml of lysis buffer (4 M guanidine isothiocyanate, 25 mM sodium citrate, pH 7, 0.5% sarcosyl, 100 mM b-mercaptoethanol) and incubated on ice for 15 minutes. Na-acetate was added to a final concentration of 0.2 M. The samples were extracted once with an equal volume of water saturated phenol and once with 24:1 (v/v) chloroform:isoamylalcolhol. RNA was precipitated overnight with an equal volume of isopropanol and washed with 75% ethanol. The pellet was resuspended in 174 μl H_2_O and 20 μl of 10× DNase buffer (100 mM Tris, pH 7.5, 25 mM MgCl_2_, 5 mM CaCl_2_) and treated with 45 U DNase I for 30 minutes at 37°C. Sodium acetate (0.2 M final concentration) was added and RNA was extracted once with an equal volume of water saturated phenol and once with chloroform:isoamylalcohol. RNA was precipitated overnight with an equal volume of isopropanol and washed with 75% ice cold ethanol. Pellets were dried under vacuum and re-suspended in H_2_O. The quality of the RNA was checked on 2% agarose gels and the concentration determined by measuring the UV absorbance at 260 nm.

### cDNA synthesis and Cy-dye labeling

cDNA was prepared using random hexamer primers (GE Healthcare, Little Chalfont, Bucks, UK). Each cDNA reaction (20 μl) contained 15 μg of RNA, 5 μg primers, 0.5 mM dATP, 0.5 mM dGTP, 0.5 mM dCTP, 0.1 mM dTTP, 0.4 mM aminoallyl-dUTP (Sigma-Aldrich Co Ltd, Gillingham, UK), 42 mM DTT and 400 units Superscript II reverse transcriptase (Invitrogen, Carlsbad, CA, USA). The RNA was incubated with the random hexamer primers at 70°C for 10 minutes and cooled rapidly on ice. Nucleotides and reverse transcriptase were added to the primed RNA and cDNA synthesis was carried out at 42°C for 2 h. RNA was degraded by addition of 2 μl 200 mM EDTA and 3 μl 1 M NaOH and the reaction was incubated at 70°C for 15 minutes, after which 3 μl of 1 M HCl was added to neutralize the solution. The cDNA was purified using a MinElute PCR purification kit (Qiagen Ltd, Crawley, W. Sussex, UK). The cDNA was eluted in 20 μl 0.1 M NaHCO_3 _and labeled with either Cy3 or Cy5 monoreactive dye (Amersham) in the dark for 90 minutes. Samples to be co-hybridized were mixed and purified using Qiagen MinElute columns. The labeled cDNA was eluted in 20 μl elution buffer (10 mM Tris-HCl pH 8.5).

### Microarray design

DNA microarrays containing 1,914 and 2,488 gene-specific tags for *S. acidocaldarius *and *S. solfataricus*, respectively, were produced as described previously [47].

### Array hybridization

Microarray slides were pre-hybridized for 40 minutes at 42°C in 10 mg/ml BSA, 5× SSC and 0.1% SDS, washed three times in dH_2_O, followed by one wash in isopropanol and dried by centrifugation. The labeled cDNA was mixed with 57 μl hybridization mix (6.5× SSC, 0.16% SDS, 66% formamide) followed by addition of 10 μg tRNA and 10 μg herring sperm DNA. Samples were denatured for 2 minutes at 95°C and rapidly cooled on ice. The array slide was placed in a hybridization chamber (TeleChem International, Sunnyvale, CA, USA) and a cover slip (number 2 LifterSlip; Erie Scientific Company, Portsmouth, NH, USA) applied over the printed area. The hybridization mixture was added and the chamber was incubated at 42°C for 16 h. Slides were washed for 5 minutes in 2× SSC, 0.1% SDS at 42°C, then for 10 minutes in 0.1× SSC, 0.1%SDS at room temperature and 5 times in 0.1× SSC at room temperature and dried by centrifugation. Slides were scanned using a GenePix Personal 4100A microarray scanner (Molecular Devices Corp, Sunnyvale, CA, USA).

### Data analysis

All data analysis was carried out using GenePix Pro 5.1 (Molecular Devices) and the free web-based BioArray Software Environment (BASE) [48]. Normalization within and between arrays was performed to remove dye specific effects and to compensate for differences between arrays [49]. Data from duplicate spots were merged, as were data from dye-swap arrays and the triplicate experiments, and each value thus represented data from 12 hybridizations.

### Quantitative PCR

RNA from *S. solfataricus *P2 was extracted at time points 30, 60, 90 and 120 minutes following UV irradiation, and purified using an RNeasy Kit (Qiagen) according to the manufacturer's directions, with the substitution of Proteinase K in place of lysozyme, allowing more efficient digestion of the protein S layer surrounding *Sulfolobus *cells. Quantitative PCR was carried out using the Biorad iScript One-Step RT-PCR kit with SYBR Green kit according to the manufacturer's directions using a BioRad iQ5 RT-PCR system. The amplification efficiency for each primer set was determined by calibration with genomic DNA. Reactions for UV-irradiated and control samples were analyzed in triplicate by quantitative PCR. Crossing-points (CPs) for each amplification were measured, and the ratio of gene expression in the UV-treated versus control cultures at each time point was quantified using the method of [50]. The gene-specific forward and reverse oligos used for amplification were (5' to 3'): tfb1for, GGCCAGAACTTTGGATGAGA' tfb1rev, CCAGCAGTTAACCCAGAACC; tfb2for, CGCGTTGAAAAGAGTCCAAT; tfb2rev, GGAAGCTGCGCTCAAAGATA; tfb3for, TTAGATTCGCGTTAAATAATGG; tfb3rev, CAAATACGATCGCTTTCTTCG; cdc6-2for, CCACATAGAGAAGAGAAGATTAAGG; cdc6-2rev, GTAGCTGTTTTCCCAGTACC; xpb1for, TGAATGCAGGGGTTCTTGTT; xpb1rev, AGTTTTGCTTGCTTGCCATT; dpoIIfor, CCGCCTAGGGATAAAACCAT; dpoIIrev, CCTCAACTTCAGGCTTTTCG; ssbfor, AGTTTTGGAAGCAAGCGAAG; ssbrev, GTGGTCCACGCGTTTTCTAT.

### Flow cytometry

Samples (0.1 ml of cell culture) were fixed in 70% ethanol (final concentration) and stored at 4°C. The fixed cells were precipitated at 13,000 rpm for 30 minutes and re-suspended in 1 ml Tris-MgCl_2 _(10 mM Tris pH 7.4, 10 mM MgCl_2_) and precipitated again for 30 minutes, 13,000 rpm at 4°C. The pellets were re-suspended in 70 μl of Tris-MgCl_2 _and mixed with an equal volume of staining solution containing ethidium bromide (20 μg/ml) and mithramycin A (100 μg/ml). Sample analysis was performed as described previously [22], except that an A40 Analyzer instrument (Apogee Flow Systems, Hemel Hempstead, Herts, UK) was used.

### Western blots

For western blots, whole cell protein was separated on NuPage 4-12% Bis-Tris SDS gels (Invitrogen). Protein content was measured by Bradford assay and the protein content of each sample was normalized. Western blots were carried out following standard procedures. For the production of TFB3 antibodies, 1 mg of purified TFB3 protein was used to raise polyclonal antibodies in sheep (Scottish National Blood Transfusion Service).

### Detection of cyclobutane pyrimidine dimers in damaged DNA

DNA was extracted using the DNeasy Tissue kit (Qiagen) according to the manufacturer's protocol. DNA was quantified spectrophotometrically and DNA concentrations were adjusted to 10 ng/μl in H_2_O. DNA was added at 300 ng per well to microtitre plates (Greiner Bio-One Ltd, Stroudwater, Stonehouse, UK) and allowed to dry at 37°C for 48 h. Plates were washed 4 times with 100 μl washing buffer (PBS, 0.1% Tween 20) and 100 μl blocking buffer (PBS, 0.1% Tween 20, 5% (w/v) milk powder) were added to each well and left at room temperature for 90 minutes. Blocking buffer was removed and wells were washed 4 times with 100 μl washing buffer. The CPD-specific antibody (Kamiya Biomedical Company, Seattle, WA, USA) was added at a concentration of 1:250 (in washing buffer) and plates were incubated at room temperature for 90 minutes. Each well was washed five times with 100 μl washing buffer and 100 μl of a 1:5,000 diluted HRP-conjugated anti-mouse antibody (Pierce, Cramlington, Northumberland, UK) was added. Plates were incubated for 90 minutes at room temperature, after which each well was washed five times with 100 μl washing buffer. A 150 μl 1-Step™ Turbo TMB Elisa (Pierce) was added to each well and incubated for 5-30 minutes at room temperature. Reactions were stopped by the addition of 150 μl 2 M sulfuric acid and absorbance was quantified at 450 nm.

### Cloning and protein purification of TFB1 and TFB3

*tfb1 *and *tfb3 *genes from *S. solfataricus *genomic DNA were amplified by PCR and cloned into a modified Gateway vector for expression with a cleavable amino-terminal his-tag. Full details of this methodology will be published elsewhere, and are available from the corresponding author on request.

pDEST-TFB1 and pDEST-TFB3 were transformed to *Escherichia coli *Rosetta (DE3) or C43 (DE3) cells, respectively. Cells were grown in LB medium at 37°C to an OD_600 _of 0.6. At this point, induction of His_6_-tagged TFB1 and TFB3 was carried out by 200 μM isopropyl β-D-thiogalactopyranoside IPTG overnight at room temperature. Cells were harvested and re-suspended in lysis buffer (20 mM Tris-HCl (pH 8.0), 500 mM NaCl, 0.1% Triton X-100, 1 mM MgCl_2 _and Complete EDTA-free protease inhibitors (Roche Basel, Switzerland)), lysed by sonication and clarified by centrifugation. The supernatant was heated to 70°C for 10 minutes and re-centrifuged. The resultant supernatant was diluted two-fold in buffer A (20 mM Tris-HCl (pH 8.0), 500 mM NaCl, 30 mM NaH_2_PO_4_) plus 30 mM imidazole and was then applied to a column containing Ni-NTA-Agarose (HiTrap 5 ml chelating HP; GE Healthcare defined above) pre-equilibrated with buffer A + 30 mM imidazole. The proteins were eluted with a linear gradient of imidazole (buffer A + 500 mM imidazole). Fractions containing His-TFB1 and His-TFB3 were identified by SDS-PAGE and pooled. For His-TFB3 purification, all the buffers were supplemented with 2 mM DTT. His-TFB1 was dialyzed against Tev cleavage buffer (20 mM Tris-HCl (pH 7.0), 500 mM NaCl, 1 mM DTT, 30 mM NaH_2_PO_4 _and 10% glycerol) overnight at 4°C. The day after, the protein was Tev cleaved overnight at room temperature by adding a final concentration of 200 ng/μl of Tev protease. Cleaved TFB1 was re-purified by loading onto the same column pre-equilibrated with buffer A + 30 mM imidazole and collecting the flow through. Positive fractions were pooled and dialyzed extensively against freezing buffer (50 mM Tris-HCl (pH 7.5), 200 mM KCl, 1 mM DTT, 1 mM EDTA, 0.01% Triton X-100 and 50% glycerol). Further purification of His-TFB3 was achieved by loading the protein onto a HiLoad 26/60 Superdex 200 pgr size exclusion column (GE Healthcare) and following the protocol as shown below with buffer supplemented with 1 mM DTT.

### Purification of *S. solfataricus *RNA polymerase

Ten liters of *S. solfataricus *P2 cells were grown up to mid-log phase. At this point, cells were harvested and re-suspended in lysis buffer (50 mM MES (pH 6.0), 100 mM NaCl, 1 mM EDTA, 1 mM DTT and Complete protease inhibitors (Roche)), lysed by sonication and clarified by centrifugation. The resultant supernatant was two times diluted in buffer 1 (20 mM MES (pH 6.0), 1 mM EDTA and 0.5 mM DTT) plus 10 mM NaCl and was then applied to a pre-equilibrated Hi-Trap 5 ml Heparin column (Amersham). The proteins were eluted with a linear gradient of NaCl (buffer 1 + 1 M NaCl). Fractions containing the RNA polymerase were identified by dot-blot immunodetection with RNA polymerase subunit B' specific antibodies and pooled. RNA polymerase was purified to homogeneity by using a HiLoad 26/60 Superdex 200 pgr size exclusion column (GE Healthcare) equilibrated with gel filtration buffer (20 mM MES (pH 6.0), 200 mM NaCl, 1 mM EDTA and 0.5 mM DTT). Fractions containing the RNA polymerase were identified by SDS-PAGE and confirmed by mass spectrometry. Positive fractions were pooled and dialyzed extensively against freezing buffer.

### Immunoprecipitation

For anti-RNA polymerase immunoprecipitations, 2 μl of antibody was added to 20 μl of Dynabeads Protein G (Invitrogen) in antibody binding buffer (20 mM MES (pH 5.0), 0.1% Tween-20) at room temperature. After 40 minutes, beads were washed twice in the same buffer plus 0.1% Tween-20 continued by an extra wash in binding buffer 'BB' (PBS, 150 mM NaCl, 0.1% Tween-20). A mix of RNA polymerase, TFB1 and His-TFB3 in amounts indicated in the figure legends were mixed in 'BB' in a total volume of 100 μl. This mix was combined with antibody-coated beads for 2 h at room temperature. Then, the beads were washed twice with buffer 'BB' and PBS and re-suspended in 2× SDS-PAGE loading buffer.

### Data

The raw data have been submitted to the EBI ArrayExpress database with accession code E-MEXP-1252.

## Abbreviations

BRE, TFB responsive element; CP, crossing point; CPD, cyclobutane pyrimidine dimer; DTT, dithiothreitol; FAD, flavin adenine dinucleotide; HTH, helix-turn-helix; ORF, open reading frame; PBS, phosphate-buffered saline; ROS, reactive oxygen species; SSB, single-stranded DNA binding protein; TBP, TATA-binding protein; TFB, transcription factor (II) B.

## Authors' contributions

DG helped design the study, carried out the microarray, flow cytometry and western blotting experiments, participated in data analysis and drafted the experimental sections of the manuscript. SP carried out the experiments with transcription factors shown in Figure 5. SM carried out the quantitative RT-PCR studies. ML helped in the microarray and flow cytometry experiments, and in the microarray data analysis. RB participated in the design of the study and participated in the analysis of data and preparation of the draft manuscript. MFW supervised the study, participated in its design and coordination and wrote the manuscript. All authors read and approved the final manuscript.

## Additional data files

The following additional data are available with the online version of this paper. Additional data file 1 is a table listing the changes in transcript abundance for all *S. solfataricus *genes. Additional data file 2 is a table listing the changes in transcript abundance for all *S. acidocaldarius *genes. Additional data file 3 is a table listing the *S. solfataricus *genes induced by UV irradiation. Additional data file 4 is a table listing the *S. solfataricus *genes repressed by UV irradiation. Additional data file 5 is a table listing the *S. acidocaldarius *genes induced by UV irradiation. Additional data file 6 is a table listing the *S. acidocaldarius *genes repressed by UV irradiation.

## Supplementary Material

Additional data file 1 Changes in transcript abundance for all *S. solfataricus *genes. Click here for file

Additional data file 2 Changes in transcript abundance for all *S. acidocaldarius *genes. Click here for file

Additional data file 3 *S. solfataricus *genes induced by UV irradiation. Click here for file

Additional data file 4 *S. solfataricus *genes repressed by UV irradiation. Click here for file

Additional data file 5 *S. acidocaldarius *genes induced by UV irradiation. Click here for file

Additional data file 6 *S. acidocaldarius *genes repressed by UV irradiation. Click here for file
