# Supplementary material for: Responses of hyperthermophilic crenarchaea to UV irradiation
Source: Genome Biol. 2007 Oct 11;8(10):R220. doi: 10.1186/gb-2007-8-10-r220 (PMC2246294; doi:10.1186/gb-2007-8-10-r220)
Supplement: Additional data file 1 — Changes in transcript abundance for all S. solfataricus genes. [file gb-2007-8-10-r220-S1.doc]

Table S1. Ratio of expression of all *S. solfataricus* genes on the array, expressed as log_2_ (UV/ control). Positive and negative numbers indicate an increase and decrease in gene expression following UV irradiation, respectively.

| Gene | 30 min | 60 min | 90 min | 120 min |
| --- | --- | --- | --- | --- |
| SSO0001 | 0.296 | 0.043 | 1.453 | 1.616 |
| SSO0002 | 0.231 | -0.006 | 0.633 | 0.639 |
| SSO0004 | NA | NA | -0.072 | NA |
| SSO0006 | 0.227 | -0.325 | 0.303 | 0.012 |
| SSO0007 | 0.377 | 0.092 | 0.195 | 0.072 |
| SSO0008 | -0.029 | -0.497 | -0.068 | -0.268 |
| SSO0009 | 0.418 | 0.152 | 0.095 | 0.181 |
| SSO0010 | 0.093 | -0.204 | -0.235 | -0.198 |
| SSO0011 | 0.379 | 0.015 | 0.33 | 0.402 |
| SSO0012 | 0.272 | 0.311 | 0.003 | 0.298 |
| SSO0013 | 0.466 | 0.313 | -0.108 | -0.079 |
| SSO0014 | 0.264 | 0.139 | 0.187 | 0.178 |
| SSO0015 | 0.484 | -0.015 | 0.01 | 0.161 |
| SSO0016 | -0.117 | -0.09 | -0.064 | -0.283 |
| SSO0017 | 0.281 | -0.018 | 0.05 | -0.09 |
| SSO0018 | -0.358 | -0.797 | -0.432 | -0.785 |
| SSO0019 | 0.089 | -0.144 | -0.059 | -0.057 |
| SSO0020 | 0.172 | 0.098 | 0.138 | 0.115 |
| SSO0021 | 0.514 | 0.283 | 0.578 | 1.088 |
| SSO0022 | 0.043 | -0.037 | 0.032 | 0.139 |
| SSO0023 | -0.119 | -0.1 | 0.244 | 0.21 |
| SSO0024 | 0.545 | 0.383 | -0.068 | 0.299 |
| SSO0025 | 0.1 | -0.133 | -0.046 | -0.049 |
| SSO0026 | -0.124 | -0.367 | -0.32 | -0.309 |
| SSO0028 | 0.672 | 0.056 | 0.015 | 0.043 |
| SSO0029 | 0.036 | -0.721 | -0.42 | -0.595 |
| SSO0031 | 0.06 | -0.436 | -0.055 | -0.373 |
| SSO0032 | -0.577 | -0.232 | 0.08 | -0.079 |
| SSO0034 | -0.521 | -1.414 | -2.356 | -1.612 |
| SSO0035 | -0.067 | -0.842 | -0.822 | -0.478 |
| SSO0036 | NA | 0.477 | NA | NA |
| SSO0037 | 0.375 | 0.457 | 2.795 | 2.805 |
| SSO0038 | 0.023 | -0.015 | 0.092 | 0.269 |
| SSO0039 | 0.359 | 0.095 | -0.246 | -0.064 |
| SSO0044 | 0.757 | -0.223 | 0.02 | -0.105 |
| SSO0045 | 0.316 | -0.069 | 0.118 | 0.01 |
| SSO0046 | 0.172 | -0.285 | -0.263 | -0.413 |
| SSO0048 | -0.198 | -0.591 | -1.324 | -1.14 |
| SSO0049 | 0.317 | 0.085 | -0.264 | -0.146 |
| SSO0050 | -0.1 | -0.344 | -0.608 | -0.534 |
| SSO0051 | 0.076 | -0.327 | -0.194 | -0.366 |
| SSO0052 | 0.109 | -0.23 | -0.212 | -0.298 |
| SSO0053 | 0.257 | -0.306 | -0.3 | -0.231 |
| SSO0054 | 0.361 | 0.245 | 0.015 | 0.13 |
| SSO0055 | 0.896 | 0.374 | 0.127 | 0.279 |
| SSO0057 | NA | -0.09 | -0.219 | 0.168 |
| SSO0060 | 0.775 | 0.303 | -0.053 | 0.081 |
| SSO0061 | 0.316 | -0.064 | 0.092 | -0.196 |
| SSO0063 | 0.376 | -0.03 | 0.053 | -0.263 |
| SSO0064 | 0.641 | 0.127 | 0.217 | 0.205 |
| SSO0065 | 0.889 | 0.216 | 0.285 | 0.279 |
| SSO0066 | 0.32 | -0.08 | -0.098 | -0.229 |
| SSO0067 | -0.126 | -0.319 | -0.246 | -0.333 |
| SSO0068 | 0.026 | -0.839 | 0.032 | -0.636 |
| SSO0069 | 0.167 | -1.004 | -0.358 | -1.028 |
| SSO0070 | -0.054 | -0.462 | 0.22 | -0.441 |
| SSO0071 | -0.278 | -0.268 | -0.123 | -0.44 |
| SSO0072 | -0.233 | 0.039 | 0.025 | 0.019 |
| SSO0073 | -0.219 | -0.462 | -0.238 | -0.524 |
| SSO0074 | -0.19 | -0.143 | -0.11 | -0.241 |
| SSO0078 | 0.143 | -0.284 | -0.026 | -0.274 |
| SSO0079 | -0.323 | -0.357 | -0.034 | 0.043 |
| SSO0081 | 0.182 | -0.051 | -0.268 | -0.444 |
| SSO0082 | 0.286 | 0.4 | 0.068 | 0.145 |
| SSO0083 | 0.52 | -0.145 | -0.113 | -0.352 |
| SSO0084 | 0.307 | 0.389 | -0.183 | -0.095 |
| SSO0085 | 0.176 | -0.082 | 0.007 | -0.157 |
| SSO0086 | 0.344 | -0.085 | -0.134 | -0.179 |
| SSO0087 | 0.711 | 0.907 | 0.335 | 0.682 |
| SSO0090 | 0.094 | -0.343 | 0.052 | -0.389 |
| SSO0091 | -2.395 | -0.459 | -0.408 | -0.286 |
| SSO0093 | -0.197 | -0.668 | -0.22 | -0.633 |
| SSO0094 | 0.331 | -0.126 | -0.009 | -0.223 |
| SSO0095 | 0.201 | 0.354 | 0.687 | 0.522 |
| SSO0097 | 0.211 | 0.057 | 0.183 | -0.069 |
| SSO0098 | 0.103 | -0.091 | -0.356 | -0.362 |
| SSO0099 | -0.178 | -0.033 | 0.008 | 0.188 |
| SSO0100 | -0.275 | -0.663 | 0.047 | -0.484 |
| SSO0101 | NA | 0.803 | NA | NA |
| SSO0102 | -0.239 | -0.428 | -0.384 | -0.413 |
| SSO0103 | 0.267 | -0.089 | -0.015 | 0.005 |
| SSO0104 | -0.141 | 0.393 | 0.143 | 0.115 |
| SSO0106 | 0.019 | 0.031 | 0.276 | 0.112 |
| SSO0107 | 0.25 | -0.166 | -0.799 | -0.555 |
| SSO0108 | -0.345 | -0.579 | -1.326 | -1.061 |
| SSO0109 | -0.13 | NA | NA | NA |
| SSO0110 | -0.559 | -0.691 | -0.83 | -0.729 |
| SSO0111 | 0.34 | -0.44 | -0.055 | -0.38 |
| SSO0114 | 0.068 | 0.035 | -0.091 | -0.038 |
| SSO0115 | 0.2 | -0.068 | 0.18 | 0.101 |
| SSO0116 | 0.112 | 0.222 | 0.356 | 0.541 |
| SSO0117 | NA | 0.319 | 1.365 | 0.889 |
| SSO0118 | 0.256 | 0.94 | 2.395 | 2.953 |
| SSO0119 | 0.28 | 0.15 | 2.007 | 2.015 |
| SSO0120 | 0.072 | 0.768 | 2.774 | 2.796 |
| SSO0121 | 0.102 | 1.002 | 2.935 | 3.316 |
| SSO0122 | 0.192 | -0.003 | 0.149 | 0.264 |
| SSO0123 | 0.012 | NA | NA | 0.032 |
| SSO0124 | 0.343 | -0.022 | -0.022 | -0.126 |
| SSO0125 | -0.169 | 0.178 | 0.26 | 0.223 |
| SSO0127 | 0.211 | 0.155 | 0.111 | 0.083 |
| SSO0128 | 0.07 | -0.265 | 0.109 | -0.057 |
| SSO0129 | 0.321 | -0.159 | 0.103 | 0.092 |
| SSO0132 | 0.532 | 0.856 | 1.291 | 1.792 |
| SSO0138 | 0.127 | 0.282 | 0.125 | 0.095 |
| SSO0140 | 0.117 | 0.008 | 0.127 | 0.186 |
| SSO0142 | 0.141 | -0.063 | 0.144 | 0.146 |
| SSO0143 | 0.399 | 0.268 | 0.72 | 1.192 |
| SSO0144 | 0.633 | -0.018 | -0.082 | -0.08 |
| SSO0147 | -0.246 | -0.236 | -0.236 | -0.189 |
| SSO0149 | -0.029 | -0.679 | -0.905 | -0.95 |
| SSO0150 | 0.151 | -0.04 | -0.751 | -0.391 |
| SSO0151 | 0.062 | 0.63 | 0.029 | 0.198 |
| SSO0152 | 0.135 | NA | 0.835 | 0.95 |
| SSO0154 | 0.034 | -0.303 | -0.043 | -0.028 |
| SSO0155 | -0.089 | -0.29 | 0.239 | -0.269 |
| SSO0156 | 0.218 | -0.503 | 0.144 | -0.479 |
| SSO0157 | 0.22 | -0.369 | 0.233 | -0.242 |
| SSO0159 | 0.53 | 0.416 | 0.384 | 0.161 |
| SSO0160 | 0.115 | -0.158 | 0.251 | -0.243 |
| SSO0162 | -0.094 | -0.174 | 0.271 | -0.005 |
| SSO0163 | -0.021 | -0.383 | -0.265 | -0.434 |
| SSO0164 | -0.169 | 0.297 | -0.181 | 0.142 |
| SSO0165 | -0.108 | -0.039 | -0.065 | -0.236 |
| SSO0166 | 0.037 | -0.374 | 0.033 | -0.329 |
| SSO0167 | 0.274 | -0.001 | -0.11 | 0.018 |
| SSO0168 | 0.625 | -0.29 | 0.064 | -0.244 |
| SSO0169 | 0.276 | 0.051 | 0.168 | 0.018 |
| SSO0170 | 0.491 | 0.14 | 0.376 | 0.114 |
| SSO0171 | 0.062 | 0.357 | -0.024 | -0.104 |
| SSO0172 | -0.263 | -0.413 | -0.062 | -0.409 |
| SSO0173 | -0.108 | -0.14 | -0.013 | -0.317 |
| SSO0174 | 0.131 | -0.072 | -0.014 | 0.01 |
| SSO0175 | 0.32 | 0.289 | -0.139 | -0.112 |
| SSO0176 | 0.189 | -0.336 | 0.333 | -0.188 |
| SSO0179 | 0.344 | -0.252 | 0.237 | -0.102 |
| SSO0180 | -0.48 | -0.722 | -0.165 | -0.414 |
| SSO0181 | -0.056 | 0.267 | 0.291 | 0.599 |
| SSO0182 | 0.012 | -0.267 | 0.305 | 0.142 |
| SSO0184 | 0.293 | NA | NA | 0.375 |
| SSO0185 | 0.164 | -0.127 | 0.06 | -0.033 |
| SSO0186 | 0.021 | -0.46 | -0.04 | -0.376 |
| SSO0187 | -0.106 | -0.413 | -0.095 | -0.024 |
| SSO0189 | -0.004 | -0.483 | -0.009 | -0.438 |
| SSO0190 | -0.196 | -1.015 | -2.14 | -1.334 |
| SSO0191 | 0.636 | -0.097 | 0.07 | -0.053 |
| SSO0192 | 0.264 | 0.16 | -0.158 | -0.183 |
| SSO0193 | -0.071 | -0.193 | -0.358 | -0.323 |
| SSO0195 | -0.316 | -0.385 | -0.038 | -0.174 |
| SSO0197 | 0.228 | 0.113 | 0.397 | 0.558 |
| SSO0198 | -0.441 | -0.581 | 0.229 | -0.111 |
| SSO0199 | 0.295 | 0.05 | 0.072 | -0.09 |
| SSO0201 | 0.257 | -0.444 | -0.285 | -0.785 |
| SSO0202 | -0.114 | -0.399 | -0.597 | -0.416 |
| SSO0203 | -0.577 | -0.256 | 0.038 | -0.007 |
| SSO0207 | 0.17 | -0.238 | 0.231 | -0.204 |
| SSO0209 | 0.305 | -0.207 | -0.034 | -0.008 |
| SSO0210 | 0.363 | -0.289 | -0.034 | -0.26 |
| SSO0212 | 0.608 | 0.154 | 0.507 | 0.566 |
| SSO0213 | 0.454 | -0.005 | 0.402 | 0.388 |
| SSO0214 | NA | NA | NA | 0.05 |
| SSO0215 | 0.016 | -0.624 | 0.174 | -0.544 |
| SSO0216 | -0.054 | -0.032 | -0.014 | 0.009 |
| SSO0217 | 0.176 | -0.229 | 0.108 | -0.437 |
| SSO0218 | 0.264 | 0.148 | 0.113 | 0.299 |
| SSO0219 | -0.206 | -0.134 | -0.654 | -0.386 |
| SSO0220 | -0.222 | -0.354 | -0.132 | -0.34 |
| SSO0221 | -0.112 | -0.134 | -0.195 | -0.177 |
| SSO0223 | -0.388 | -0.629 | -0.179 | -0.499 |
| SSO0225 | -0.611 | -0.393 | -0.095 | -0.371 |
| SSO0227 | 0.028 | -0.506 | 0.237 | -0.449 |
| SSO0228 | 0.17 | -0.294 | 0.309 | 0.193 |
| SSO0230 | 0.316 | 0.308 | -0.347 | 0.033 |
| SSO0231 | 0.177 | -0.317 | -0.09 | -0.265 |
| SSO0232 | 0.102 | -0.629 | -0.145 | -0.705 |
| SSO0233 | -0.111 | -0.346 | -0.108 | -0.238 |
| SSO0234 | -0.003 | NA | NA | -0.337 |
| SSO0235 | -0.06 | 0.013 | -0.002 | -0.24 |
| SSO0237 | 0.519 | 0.514 | 0.115 | 0.447 |
| SSO0239 | -0.389 | -0.955 | -0.515 | -1.061 |
| SSO0240 | -0.2 | -0.44 | -0.247 | -0.555 |
| SSO0241 | -0.54 | -1.07 | -0.459 | -0.839 |
| SSO0242 | 0.243 | 0.021 | 0.248 | -0.096 |
| SSO0244 | -0.105 | -0.455 | 0.045 | -0.402 |
| SSO0245 | -0.238 | -0.549 | -0.032 | -0.277 |
| SSO0246 | 0.18 | 0.022 | 0.172 | -0.143 |
| SSO0248 | 0.442 | 0.178 | 0.349 | 0.151 |
| SSO0249 | 0.244 | 0.478 | 0.671 | 0.602 |
| SSO0250 | 0.037 | -0.05 | 0.184 | -0.202 |
| SSO0251 | 0.05 | 0.002 | -0.047 | -0.053 |
| SSO0252 | 0.471 | 0.345 | 0.351 | 0.332 |
| SSO0253 | 0.16 | -0.048 | -0.161 | -0.23 |
| SSO0254 | -0.117 | -0.205 | 0.49 | 0.197 |
| SSO0255 | -0.089 | 0.03 | 0.268 | 0.141 |
| SSO0256 | 0.322 | 0.049 | -0.046 | -0.07 |
| SSO0257 | -0.014 | -0.248 | -1.421 | -0.905 |
| SSO0259 | 0.734 | 0.439 | 0.297 | 0.46 |
| SSO0260 | 0.479 | 0.374 | -0.067 | NA |
| SSO0261 | 0.506 | -0.181 | -0.019 | 0.02 |
| SSO0264 | -0.012 | 0.076 | 0.124 | 0.236 |
| SSO0265 | 0.042 | -0.302 | 0.302 | 0.235 |
| SSO0266 | -0.048 | -0.355 | 0.001 | -0.252 |
| SSO0267 | 0.16 | -0.166 | -0.038 | -0.087 |
| SSO0269 | 0.348 | -0.193 | 0.412 | 0.017 |
| SSO0270 | 0.506 | 0.291 | 0.223 | 0.121 |
| SSO0271 | -0.002 | -0.248 | -1.116 | -0.874 |
| SSO0273 | -0.102 | -0.223 | -0.277 | -0.334 |
| SSO0274 | 0.045 | -0.515 | 0.002 | -0.3 |
| SSO0276 | -0.233 | -0.424 | -0.158 | -0.201 |
| SSO0277 | 0.257 | 0.208 | -0.134 | -0.197 |
| SSO0278 | -0.11 | -0.104 | 0.137 | -0.105 |
| SSO0279 | 0.043 | -0.147 | -0.146 | -0.315 |
| SSO0280 | 0.147 | 1.496 | 3.047 | 3.005 |
| SSO0281 | 0.119 | -0.074 | -0.113 | -0.194 |
| SSO0282 | -0.064 | -0.728 | -0.063 | -0.147 |
| SSO0283 | 0.511 | 0.487 | 2.315 | 2.224 |
| SSO0284 | 0.069 | -0.391 | -0.02 | -0.613 |
| SSO0286 | 0.132 | -0.146 | -0.094 | -0.352 |
| SSO0287 | -0.102 | -0.347 | 0.017 | -0.425 |
| SSO0290 | 1.103 | 0.941 | 0.204 | 0.251 |
| SSO0291 | 0.106 | 0.536 | -0.004 | 0.218 |
| SSO0293 | 0.721 | -0.244 | -0.191 | -0.549 |
| SSO0294 | -0.147 | 0.557 | -0.112 | 0.221 |
| SSO0296 | 0.182 | -0.129 | -0.149 | -0.207 |
| SSO0297 | 0.639 | 0.102 | 0.257 | 0.062 |
| SSO0298 | -0.32 | -0.012 | 0.162 | 0.046 |
| SSO0299 | 0.145 | -0.246 | 0.434 | -0.159 |
| SSO0302 | 0.063 | -0.452 | 0.089 | -0.382 |
| SSO0304 | -0.06 | -0.511 | -0.068 | -0.429 |
| SSO0305 | -0.151 | -0.346 | -0.038 | -0.451 |
| SSO0306 | 0.154 | -0.579 | 0.007 | -0.372 |
| SSO0307 | 0.123 | -0.212 | 0.106 | -0.281 |
| SSO0308 | 0.309 | -0.307 | 0.183 | -0.069 |
| SSO0309 | 0.358 | -0.1 | 0.027 | -0.008 |
| SSO0311 | 0.593 | 0.093 | -0.144 | 0.093 |
| SSO0312 | 0.289 | -0.017 | 0.052 | 0.041 |
| SSO0313 | 0.526 | 0.233 | 0.355 | 0.515 |
| SSO0314 | 0.142 | -0.133 | 0.089 | -0.145 |
| SSO0315 | -0.177 | -0.384 | -0.633 | -0.588 |
| SSO0316 | -0.138 | 0.039 | 0.127 | 0.234 |
| SSO0317 | 0.199 | -0.273 | 0.01 | -0.256 |
| SSO0318 | -0.185 | -0.252 | 0.127 | -0.171 |
| SSO0321 | 0.047 | -0.51 | -1.795 | -1.096 |
| SSO0322 | 0.093 | 0.23 | -0.138 | 0.081 |
| SSO0323 | 0.103 | -0.037 | 0.04 | -0.093 |
| SSO0324 | -0.169 | -0.413 | 0.15 | -0.125 |
| SSO0325 | -0.347 | -0.29 | -0.219 | -0.295 |
| SSO0326 | -0.278 | -0.095 | -0.001 | -0.105 |
| SSO0327 | -0.266 | -0.012 | -0.043 | -0.045 |
| SSO0328 | -0.333 | 0.074 | 0.202 | 0.299 |
| SSO0329 | -0.316 | -0.163 | -0.055 | -0.046 |
| SSO0330 | -0.601 | -0.352 | -0.162 | -0.152 |
| SSO0333 | 0.192 | -0.008 | 0.418 | 0.737 |
| SSO0334 | -0.036 | -0.628 | -0.028 | -0.711 |
| SSO0335 | -0.212 | -0.814 | -0.111 | -0.967 |
| SSO0337 | 0.395 | 0.131 | 0.417 | 0.303 |
| SSO0341 | 0.56 | -0.092 | 0.146 | -0.309 |
| SSO0342 | -0.44 | -0.232 | -0.381 | -0.435 |
| SSO0344 | -0.181 | -0.428 | -0.012 | -0.581 |
| SSO0345 | -0.129 | -0.71 | -0.609 | -1.207 |
| SSO0346 | 0.072 | -0.52 | -0.346 | -0.733 |
| SSO0347 | 0.459 | 0.012 | -0.163 | -0.183 |
| SSO0348 | 0.002 | -0.581 | -0.138 | -0.183 |
| SSO0349 | -0.463 | -0.447 | -0.245 | -0.367 |
| SSO0351 | -0.196 | -0.475 | -0.026 | -0.309 |
| SSO0352 | -0.8 | -1.044 | -0.533 | -0.846 |
| SSO0353 | -0.18 | -0.494 | -0.989 | -0.817 |
| SSO0354 | 0.152 | 0.257 | 0.196 | 0.239 |
| SSO0356 | -0.375 | -1.111 | -0.769 | -1.375 |
| SSO0358 | 0.466 | -0.149 | -0.1 | -0.305 |
| SSO0359 | 0.312 | 0.034 | 0.096 | -0.152 |
| SSO0362 | 0.315 | 0.16 | 0.181 | 0.069 |
| SSO0363 | 0.115 | 0.169 | 0.242 | 0.114 |
| SSO0364 | 0.474 | 0.534 | -0.172 | 0.047 |
| SSO0365 | 0.263 | NA | 0.015 | -0.094 |
| SSO0366 | 0.095 | 0.006 | -0.139 | -0.28 |
| SSO0367 | 0.316 | 0.396 | 0.31 | 0.207 |
| SSO0368 | 0.47 | 0.352 | 0.523 | 0.513 |
| SSO0369 | -0.064 | -0.276 | 0.515 | 0.522 |
| SSO0370 | 0.155 | 0.236 | -0.077 | 0.096 |
| SSO0371 | 0.37 | 0.012 | 0.068 | 0.061 |
| SSO0372 | 0.216 | 0.047 | -0.078 | -0.079 |
| SSO0373 | -0.018 | 0.016 | -0.079 | -0.227 |
| SSO0375 | -0.283 | -0.775 | -0.95 | -0.792 |
| SSO0376 | 0.152 | NA | NA | 0.205 |
| SSO0378 | 0.073 | -0.153 | -0.234 | -0.334 |
| SSO0379 | -0.017 | 0.27 | -0.151 | 0.587 |
| SSO0381 | 0.143 | -0.505 | 0.005 | -0.411 |
| SSO0382 | 0.073 | -0.188 | 0.105 | -0.143 |
| SSO0383 | 0.323 | -0.151 | -0.23 | -0.304 |
| SSO0386 | 0.121 | -0.16 | -0.265 | -0.326 |
| SSO0387 | 0.054 | 0.044 | 0.052 | 0.11 |
| SSO0388 | 0.205 | -0.14 | -0.361 | 0.134 |
| SSO0389 | 0.35 | 0.005 | 0.084 | -0.151 |
| SSO0390 | 0.262 | 0.067 | 0.173 | 0.012 |
| SSO0391 | 0.345 | 0.137 | 0.238 | 0.181 |
| SSO0392 | 0.291 | -0.176 | 0.137 | -0.063 |
| SSO0393 | -0.122 | -0.434 | -0.05 | -0.32 |
| SSO0394 | 0.7 | 0.803 | 0.833 | 1.25 |
| SSO0395 | 0.057 | NA | NA | 0.07 |
| SSO0396 | 0.14 | -0.189 | -0.284 | -0.185 |
| SSO0397 | 0.192 | -0.06 | -0.068 | -0.28 |
| SSO0399 | 0.579 | 0.099 | 0.064 | 0.06 |
| SSO0400 | 0.856 | 0.296 | 0.113 | -0.081 |
| SSO0401 | 1.096 | 0.503 | -0.079 | -0.19 |
| SSO0402 | 1.017 | 0.671 | 0.442 | 0.614 |
| SSO0405 | -0.098 | -0.116 | -0.752 | -0.239 |
| SSO0407 | -0.195 | -0.617 | -0.098 | -0.703 |
| SSO0408 | -0.218 | -0.05 | -0.207 | -0.082 |
| SSO0410 | 0.324 | 0.356 | 0.01 | 0.214 |
| SSO0411 | -0.244 | -0.511 | -0.256 | -0.537 |
| SSO0412 | -0.065 | -0.253 | 0.494 | -0.233 |
| SSO0414 | NA | NA | -0.011 | 0.155 |
| SSO0415 | -0.057 | -0.024 | -0.43 | -0.575 |
| SSO0416 | 0.13 | -0.071 | -0.028 | 0.034 |
| SSO0417 | -0.057 | -0.251 | 0.155 | -0.355 |
| SSO0420 | 0.515 | 0.12 | 0.003 | -0.33 |
| SSO0421 | -0.375 | -0.858 | 0.253 | -0.677 |
| SSO0422 | 0.626 | 0.142 | 0.01 | -0.025 |
| SSO0425 | -0.292 | 0.073 | 0.656 | 0.75 |
| SSO0426 | 0.342 | -0.063 | 0.068 | 0.083 |
| SSO0427 | 0.199 | -0.105 | -0.122 | -0.162 |
| SSO0428 | -0.45 | -0.261 | -0.181 | -0.316 |
| SSO0429 | -0.215 | -0.661 | -0.225 | -0.578 |
| SSO0430 | -0.106 | -0.043 | 0.168 | 0.152 |
| SSO0432 | 0.267 | -0.291 | NA | 0.054 |
| SSO0433 | 0.136 | -0.223 | 0.34 | -0.09 |
| SSO0434 | 0.048 | 0.176 | 0.499 | 0.306 |
| SSO0435 | -0.171 | -0.313 | -0.306 | -0.44 |
| SSO0436 | 0.302 | 0.06 | 0.662 | 0.566 |
| SSO0437 | 0.358 | 0.233 | 0.354 | 0.532 |
| SSO0438 | 0.011 | -0.068 | 0.049 | -0.126 |
| SSO0439 | 0.308 | -0.21 | -0.37 | -0.466 |
| SSO0440 | 0.053 | -0.254 | -0.11 | -0.277 |
| SSO0441 | -0.033 | -0.224 | -0.252 | -0.167 |
| SSO0442 | -0.198 | -0.253 | -0.368 | -0.221 |
| SSO0444 | 0.179 | -0.426 | -0.138 | -0.484 |
| SSO0445 | 0.615 | 0.228 | 0.023 | -0.001 |
| SSO0446 | -0.04 | 0.366 | 0.809 | 0.764 |
| SSO0447 | 0.111 | 0.137 | -0.242 | -0.119 |
| SSO0448 | -0.041 | 0.094 | 0.19 | 0.024 |
| SSO0449 | 0.258 | 0.411 | 0.472 | 0.349 |
| SSO0450 | -0.378 | -0.039 | -0.116 | -0.074 |
| SSO0451 | -0.787 | -0.813 | -2.237 | -1.823 |
| SSO0452 | -0.169 | -0.275 | -0.349 | -0.46 |
| SSO0453 | 0.055 | -0.043 | 0.209 | 0.217 |
| SSO0454 | 0.002 | -0.236 | -0.074 | -0.178 |
| SSO0455 | 0.01 | -0.094 | -0.04 | -0.071 |
| SSO0456 | 0.221 | -0.077 | 0.238 | 0.133 |
| SSO0458 | 0.376 | -0.131 | -0.202 | -0.257 |
| SSO0460 | 0.217 | 0.258 | 0.178 | 0.095 |
| SSO0461 | 0.166 | -0.135 | -0.531 | -0.261 |
| SSO0464 | 0.221 | 0.135 | 0.008 | -0.087 |
| SSO0465 | 0.25 | 0.345 | 0.258 | 0.178 |
| SSO0466 | 0.354 | -0.031 | 0.187 | 0.033 |
| SSO0467 | -0.031 | -0.353 | -0.586 | -0.412 |
| SSO0468 | -0.02 | -0.15 | -0.183 | -0.323 |
| SSO0469 | 0.055 | -0.471 | 0.084 | -0.353 |
| SSO0470 | -0.214 | -0.316 | -0.126 | -0.336 |
| SSO0472 | 0.63 | 0.31 | 0.496 | 0.599 |
| SSO0473 | 0.516 | 0.17 | 0.226 | 0.114 |
| SSO0475 | 0.077 | -0.315 | -0.02 | -0.167 |
| SSO0477 | 0.162 | 1.008 | 2.747 | 2.177 |
| SSO0478 | 0.4 | 0.179 | 0.117 | 0.306 |
| SSO0480 | 0.108 | -0.18 | 0.135 | 0.132 |
| SSO0481 | -0.107 | -0.234 | -0.222 | -0.418 |
| SSO0482 | 0.388 | 0.422 | 0.123 | 0.394 |
| SSO0483 | 0.241 | -0.234 | 0.019 | 0.03 |
| SSO0484 | 0.457 | -0.125 | 0.268 | 0.072 |
| SSO0485 | -0.062 | -0.29 | -0.078 | 0.052 |
| SSO0486 | 0.651 | 0.07 | 0.186 | 0.208 |
| SSO0488 | 0.411 | -0.184 | -0.484 | -0.424 |
| SSO0489 | 0.119 | -0.47 | -0.513 | -0.343 |
| SSO0490 | 0.452 | -0.021 | 0.052 | 0.012 |
| SSO0491 | 0.228 | NA | NA | 0.041 |
| SSO0493 | NA | NA | -0.029 | NA |
| SSO0495 | 0.104 | 0.149 | -0.135 | 0.012 |
| SSO0496 | 0.12 | -0.122 | -0.331 | -0.267 |
| SSO0497 | -0.249 | -0.274 | -0.277 | -0.475 |
| SSO0499 | 0.243 | -0.061 | -0.171 | -0.178 |
| SSO0500 | 0.257 | -0.125 | -0.007 | -0.108 |
| SSO0501 | 0.104 | -0.08 | -0.37 | -0.213 |
| SSO0502 | 0.413 | 0.225 | -0.027 | 0.017 |
| SSO0503 | 0.13 | 0.075 | 0.152 | 0.184 |
| SSO0504 | -0.076 | -0.587 | -0.092 | -0.476 |
| SSO0505 | -0.527 | -0.576 | -0.409 | -0.627 |
| SSO0506 | 0.195 | -0.471 | -0.055 | -0.094 |
| SSO0507 | -0.143 | -0.092 | -0.053 | 0.205 |
| SSO0515 | -0.483 | 0.593 | 0.3 | 0.571 |
| SSO0519 | 0.466 | 0.331 | 0.426 | 0.439 |
| SSO0520 | -0.029 | 1.118 | 0.341 | 0.707 |
| SSO0521 | 0.646 | 0.716 | 0.229 | 0.601 |
| SSO0522 | 0.341 | 0.467 | 1.23 | 1.477 |
| SSO0523 | 0.779 | 0.433 | 0.116 | 0.082 |
| SSO0526 | 0.264 | -0.007 | -0.207 | -0.142 |
| SSO0527 | 0.087 | -0.119 | 0.417 | -0.016 |
| SSO0528 | -0.018 | -0.158 | -0.173 | -0.729 |
| SSO0529 | 0.027 | -0.589 | 0.003 | -0.016 |
| SSO0530 | 0.566 | 0.146 | -0.176 | -0.168 |
| SSO0531 | 0.479 | -0.218 | -0.016 | -0.33 |
| SSO0532 | -0.072 | -0.157 | -0.419 | -0.644 |
| SSO0534 | 0.33 | -0.481 | -0.086 | -0.441 |
| SSO0535 | 0.53 | 0.381 | -0.247 | -0.457 |
| SSO0536 | 0.33 | 0.587 | -0.175 | -0.068 |
| SSO0537 | -0.075 | -0.561 | -0.722 | -0.707 |
| SSO0538 | 0.264 | 0.449 | 0.459 | 0.74 |
| SSO0539 | 0.348 | 0.547 | 0.749 | 0.73 |
| SSO0540 | 0.443 | 0.504 | 0.346 | 0.36 |
| SSO0544 | 0.755 | 0.013 | 0.069 | 0.189 |
| SSO0545 | 0.273 | -0.126 | -0.041 | -0.146 |
| SSO0546 | 0.015 | -0.244 | -0.087 | -0.275 |
| SSO0548 | 0.299 | 0.004 | -0.063 | 0.008 |
| SSO0550 | 0.4 | 0.149 | -0.243 | -0.138 |
| SSO0551 | 0.123 | -0.055 | -0.011 | -0.042 |
| SSO0552 | 0.058 | -0.488 | 0.147 | -0.021 |
| SSO0553 | 0.112 | -0.45 | -0.016 | -0.581 |
| SSO0554 | -0.074 | -0.483 | -0.102 | -0.411 |
| SSO0555 | 0.024 | 0.05 | -0.167 | -0.409 |
| SSO0556 | -0.051 | 0.041 | -0.262 | -0.257 |
| SSO0557 | 0.039 | -0.405 | -0.379 | -0.474 |
| SSO0558 | 0.055 | -0.374 | 0.209 | -0.437 |
| SSO0559 | 0.112 | -0.227 | 0.5 | -0.106 |
| SSO0560 | 0.071 | -0.141 | -0.14 | -0.389 |
| SSO0561 | -0.509 | -0.541 | -0.345 | -0.604 |
| SSO0563 | 0.038 | -0.521 | 0.123 | -0.455 |
| SSO0564 | -0.067 | -0.412 | 0.181 | -0.526 |
| SSO0566 | -0.137 | -0.569 | -0.042 | -0.55 |
| SSO0567 | -0.52 | 0.148 | -0.303 | -0.226 |
| SSO0569 | 0.088 | -0.126 | -0.264 | -0.177 |
| SSO0570 | 0.892 | -0.081 | 0.578 | -0.023 |
| SSO0571 | 0.242 | -0.251 | 0.412 | 0.013 |
| SSO0572 | 0.434 | -0.194 | -0.2 | -0.26 |
| SSO0575 | 0.479 | 0.131 | 0.49 | 0.352 |
| SSO0576 | 0.01 | -0.186 | 0.321 | -0.057 |
| SSO0577 | -0.061 | -0.751 | -0.196 | -0.693 |
| SSO0579 | -0.1 | -0.356 | -0.431 | -0.341 |
| SSO0580 | 0.171 | 0.256 | 0.243 | 0.391 |
| SSO0581 | 0.034 | 0.098 | 0.062 | 0.164 |
| SSO0582 | 0.02 | -0.424 | -0.199 | -0.504 |
| SSO0583 | 0.07 | -0.023 | -0.18 | -0.143 |
| SSO0584 | 0.159 | 0.032 | 0.123 | 0.05 |
| SSO0585 | 0.023 | 0.174 | -0.219 | -0.191 |
| SSO0586 | 0.43 | -0.089 | -0.002 | -0.039 |
| SSO0588 | -0.234 | -0.129 | 0.261 | 0.16 |
| SSO0589 | 0.15 | -0.062 | 0.158 | -0.248 |
| SSO0591 | 0.148 | -0.072 | -0.089 | -0.036 |
| SSO0592 | 0.185 | 0.553 | 0.504 | 0.396 |
| SSO0593 | 0.294 | -0.24 | 0.387 | -0.125 |
| SSO0594 | 0.208 | -0.303 | -0.04 | -0.419 |
| SSO0596 | 0.229 | -0.144 | 0.197 | -0.048 |
| SSO0597 | 0.044 | -0.293 | 0.077 | -0.24 |
| SSO0599 | 0.071 | -0.424 | 0.115 | -0.21 |
| SSO0600 | -0.058 | -0.09 | 0.289 | 0.239 |
| SSO0601 | -1.303 | NA | -0.581 | NA |
| SSO0602 | 0.057 | -0.148 | 0.104 | -0.137 |
| SSO0604 | -0.128 | -0.222 | 0.179 | 0.136 |
| SSO0605 | 0.574 | 0.152 | -0.01 | -0.091 |
| SSO0606 | -0.004 | 0.114 | 0.19 | 0.465 |
| SSO0607 | 0.531 | 0.122 | 0.153 | 0.108 |
| SSO0608 | 0.003 | NA | -0.817 | NA |
| SSO0609 | 0.087 | -0.641 | -0.64 | -0.606 |
| SSO0610 | 0.335 | -0.053 | 0.59 | 0.521 |
| SSO0612 | 0.432 | -0.168 | 0.007 | -0.181 |
| SSO0613 | -0.124 | -0.313 | -0.244 | -0.602 |
| SSO0614 | 0.558 | 0.121 | -0.161 | -0.203 |
| SSO0616 | 0.463 | -0.057 | -0.099 | -0.181 |
| SSO0617 | 1.049 | 0.372 | 0.12 | 0.227 |
| SSO0618 | -0.646 | -0.942 | 0.504 | 0.349 |
| SSO0619 | -0.293 | -0.89 | -1.008 | -0.956 |
| SSO0624 | 0.339 | 0.154 | 0.084 | -0.05 |
| SSO0625 | 0.361 | -0.392 | -0.096 | -0.363 |
| SSO0626 | 0.136 | -0.294 | 0.155 | -0.019 |
| SSO0627 | -0.236 | -0.205 | -0.522 | -0.352 |
| SSO0628 | 0.282 | -0.451 | 0.061 | -0.272 |
| SSO0629 | -0.037 | -0.409 | 0.06 | -0.128 |
| SSO0632 | -0.082 | -0.598 | -0.047 | -0.1 |
| SSO0633 | -0.348 | -0.672 | 0.06 | -0.057 |
| SSO0635 | 0.156 | -0.247 | -0.042 | -0.193 |
| SSO0636 | -0.053 | -0.267 | -0.093 | -0.3 |
| SSO0637 | -0.199 | 0.028 | -0.791 | -0.476 |
| SSO0638 | 0.149 | 0.472 | 0.159 | -0.236 |
| SSO0639 | 0.063 | -0.098 | 0.35 | -0.208 |
| SSO0640 | -0.015 | -0.197 | 0.297 | -0.184 |
| SSO0641 | -0.343 | -0.379 | 0.215 | -0.292 |
| SSO0645 | -0.231 | -0.102 | 0.152 | 0.107 |
| SSO0646 | 0.301 | -0.234 | 0.08 | 0.092 |
| SSO0647 | 0.363 | -0.042 | 0.322 | 0.459 |
| SSO0648 | -0.084 | 0.377 | -0.415 | 0.071 |
| SSO0649 | 0.572 | 0.522 | 0.041 | 0.201 |
| SSO0650 | 0.42 | 0.574 | -0.258 | 0.182 |
| SSO0651 | 0.256 | 0.115 | -0.019 | 0.014 |
| SSO0652 | 0.072 | 0.167 | 0.073 | 0.151 |
| SSO0653 | 0.249 | 0.078 | 0.219 | 0.201 |
| SSO0654 | -0.362 | 0.022 | -0.02 | -0.03 |
| SSO0655 | -0.015 | 0.071 | -0.027 | -0.108 |
| SSO0656 | 0.574 | 0.157 | -0.209 | 0.006 |
| SSO0657 | -0.348 | -0.182 | -0.153 | -0.198 |
| SSO0658 | 0.064 | -0.268 | 0.081 | -0.238 |
| SSO0659 | 0.176 | -0.01 | -0.06 | -0.073 |
| SSO0660 | 0.024 | -0.328 | -0.139 | -0.343 |
| SSO0661 | -0.214 | -0.559 | -0.086 | -0.405 |
| SSO0662 | 0.162 | -0.204 | 0.249 | -0.098 |
| SSO0664 | 0.072 | -0.128 | 0.032 | -0.254 |
| SSO0665 | 0.105 | 0.775 | 0.632 | 1.127 |
| SSO0666 | 0.57 | 0.645 | 0.849 | 1.121 |
| SSO0668 | 0.032 | -0.275 | -0.04 | -0.234 |
| SSO0669 | -0.066 | -0.314 | 0.076 | -0.107 |
| SSO0670 | 0.204 | 0.017 | -0.592 | -0.266 |
| SSO0671 | 0.235 | 0.311 | 0.022 | 0.187 |
| SSO0673 | 0.428 | 0.193 | 0.166 | 0.19 |
| SSO0674 | 0.563 | 0.045 | 0.001 | 0.073 |
| SSO0675 | 0.367 | 0.279 | 0.084 | 0.195 |
| SSO0676 | 0.026 | -0.319 | 0.087 | -0.076 |
| SSO0678 | 0.673 | 0.287 | 0.563 | 0.38 |
| SSO0679 | -0.032 | -0.174 | -0.677 | NA |
| SSO0680 | 0.444 | 0.187 | -0.245 | 0.04 |
| SSO0681 | 0.511 | -0.005 | -0.253 | -0.076 |
| SSO0683 | 0.692 | -0.02 | -0.106 | -0.021 |
| SSO0684 | 0.982 | -0.135 | 0.355 | 0.324 |
| SSO0686 | 0.417 | 0.021 | 0.501 | 0.439 |
| SSO0687 | -0.071 | 0.014 | 0.482 | 0.01 |
| SSO0688 | 0.135 | 0.137 | 0.282 | 0.067 |
| SSO0689 | 0.112 | 0.143 | -0.046 | -0.118 |
| SSO0690 | 0.025 | 0.091 | 0.185 | 0.186 |
| SSO0691 | 0.359 | 1.594 | 3.286 | 3.643 |
| SSO0692 | 0.067 | -0.056 | -0.187 | -0.261 |
| SSO0693 | 0.692 | -0.013 | 0.248 | 0.155 |
| SSO0694 | 0.159 | 0.113 | 0.67 | 0.524 |
| SSO0695 | 0.333 | 0.177 | 0.746 | 0.58 |
| SSO0696 | -0.431 | -0.276 | -0.249 | -0.335 |
| SSO0697 | -0.113 | -0.472 | 0.103 | -0.606 |
| SSO0698 | -0.291 | -0.492 | -0.135 | -0.739 |
| SSO0699 | -0.123 | -0.202 | -0.218 | -0.276 |
| SSO0700 | -0.129 | -0.579 | -0.113 | -0.506 |
| SSO0701 | -0.133 | -0.463 | -0.267 | -0.335 |
| SSO0702 | -0.111 | -0.451 | -0.168 | -0.444 |
| SSO0703 | 0.104 | -0.361 | -0.289 | -0.511 |
| SSO0704 | 0.298 | -0.272 | 0.031 | -0.467 |
| SSO0705 | 0.098 | -0.462 | 0.071 | -0.492 |
| SSO0707 | 0.321 | -0.242 | -0.153 | -0.483 |
| SSO0708 | 0.444 | -0.269 | -0.098 | -0.45 |
| SSO0709 | 0.332 | -0.074 | -0.209 | -0.362 |
| SSO0712 | 0.091 | -0.9 | -0.16 | -0.743 |
| SSO0713 | 0.293 | -0.399 | -0.203 | -0.328 |
| SSO0715 | 0.227 | NA | 0.166 | NA |
| SSO0716 | 0.196 | -0.056 | 0.218 | -0.095 |
| SSO0718 | -0.042 | -0.043 | -0.03 | -0.008 |
| SSO0719 | 0.193 | 0.104 | 0.619 | 0.25 |
| SSO0720 | 0.265 | 0.037 | 0.24 | 0.238 |
| SSO0722 | -0.231 | -0.303 | 0.278 | -0.287 |
| SSO0723 | -0.153 | 0.079 | 0.067 | -0.172 |
| SSO0724 | 0.207 | 0.01 | -0.075 | -0.19 |
| SSO0726 | 0.518 | -0.131 | 0.037 | -0.254 |
| SSO0728 | -0.235 | -0.14 | 0.181 | 0.076 |
| SSO0729 | 0.232 | 0.332 | 0.136 | 0.204 |
| SSO0730 | -0.273 | -0.057 | -0.244 | -0.139 |
| SSO0731 | 0.246 | 0.58 | 0.142 | 0.476 |
| SSO0732 | -0.013 | -0.269 | 0.216 | -0.109 |
| SSO0735 | 0.342 | -0.168 | 0.364 | 0.024 |
| SSO0736 | 0.466 | -0.274 | 0.389 | -0.24 |
| SSO0738 | 0.023 | 0.556 | 0.169 | 0.668 |
| SSO0739 | 0.414 | 0.187 | 0.203 | 0.328 |
| SSO0740 | 0.496 | 0.118 | 0.236 | 0.293 |
| SSO0741 | 0.492 | 0.107 | NA | -0.19 |
| SSO0742 | 0.228 | -0.092 | -0.5 | -0.434 |
| SSO0743 | -0.306 | -0.365 | -0.073 | -0.302 |
| SSO0745 | 0.334 | -0.008 | 0.48 | 0.259 |
| SSO0746 | -0.207 | -0.111 | -0.161 | -0.062 |
| SSO0747 | 0.186 | -0.137 | 0.242 | -0.021 |
| SSO0748 | 0.364 | NA | 0.381 | NA |
| SSO0749 | 0.708 | 0.473 | 0.395 | 0.572 |
| SSO0751 | 0.211 | -0.204 | -0.163 | -0.307 |
| SSO0752 | -0.357 | -0.001 | -0.415 | -0.356 |
| SSO0753 | 0.455 | 0.366 | 0.745 | 0.885 |
| SSO0754 | 0.372 | 0.338 | 0.504 | 0.486 |
| SSO0755 | -0.122 | -0.469 | -0.162 | -0.633 |
| SSO0757 | -0.131 | -0.004 | 0.005 | -0.121 |
| SSO0758 | -0.334 | -0.017 | -0.164 | -0.019 |
| SSO0760 | 0.486 | -0.24 | 0.094 | -0.179 |
| SSO0761 | -0.526 | -0.42 | 0.798 | 0.116 |
| SSO0762 | NA | 0.537 | NA | 0.233 |
| SSO0764 | -0.176 | -0.444 | -0.141 | -0.552 |
| SSO0765 | -0.013 | 0.049 | 0.043 | -0.056 |
| SSO0766 | -0.12 | 0.275 | -0.083 | 0.155 |
| SSO0767 | 0.378 | -0.102 | 0.353 | -0.002 |
| SSO0768 | 0.205 | -0.421 | 0.042 | -0.359 |
| SSO0769 | 0.171 | -0.389 | -0.05 | -0.308 |
| SSO0770 | 0.212 | 1.889 | 2.399 | 1.783 |
| SSO0772 | 0.013 | -0.811 | -0.577 | -0.869 |
| SSO0773 | 0.055 | 0.096 | -0.168 | 0.228 |
| SSO0774 | -0.028 | -0.53 | -0.225 | -0.37 |
| SSO0775 | 0.377 | 0.382 | 0.254 | 0.162 |
| SSO0776 | 0.115 | 0.058 | 0.018 | 0.076 |
| SSO0777 | 0.198 | NA | -0.166 | -0.185 |
| SSO0779 | -0.159 | -0.021 | 0.175 | -0.027 |
| SSO0780 | 0.062 | -0.07 | 0.172 | 0.298 |
| SSO0781 | -0.072 | 0.526 | 0.149 | 0.515 |
| SSO0785 | 0.091 | -0.041 | -0.17 | -0.198 |
| SSO0788 | 0.174 | -0.191 | -0.015 | 0.034 |
| SSO0790 | NA | 0.567 | NA | NA |
| SSO0792 | 0.555 | 0.31 | 0.978 | 1.212 |
| SSO0793 | 0.433 | 0.475 | 1.372 | 1.799 |
| SSO0795 | 0.266 | -0.08 | 0.337 | 0.133 |
| SSO0796 | -0.112 | 0.476 | 0.171 | 0.355 |
| SSO0798 | 0.393 | 0.365 | 0.173 | 0.348 |
| SSO0800 | NA | 0.328 | NA | NA |
| SSO0804 | -0.017 | -0.135 | -0.257 | -0.087 |
| SSO0805 | 0.25 | 0.075 | 0.058 | 0.21 |
| SSO0806 | 0.141 | 0.344 | 0.671 | 0.99 |
| SSO0807 | -0.023 | 0.011 | 0.459 | 0.972 |
| SSO0808 | 0.047 | 0.233 | -0.209 | -0.157 |
| SSO0809 | -0.39 | -0.384 | -0.185 | -0.495 |
| SSO0810 | -0.319 | -0.798 | -0.109 | -0.746 |
| SSO0813 | -0.141 | -0.242 | -0.037 | -0.307 |
| SSO0814 | -0.633 | -0.08 | -0.126 | 0.265 |
| SSO0816 | 0.024 | 0.1 | 0.319 | 0.214 |
| SSO0817 | -0.208 | 0.174 | -0.507 | -0.396 |
| SSO0818 | 0.023 | -0.076 | -0.342 | -0.385 |
| SSO0819 | -0.286 | -0.546 | -0.019 | -0.282 |
| SSO0820 | 0.026 | 0.218 | -0.034 | 0.196 |
| SSO0821 | -0.41 | -0.122 | -0.029 | -0.112 |
| SSO0823 | 0.301 | 0.158 | 1.508 | 2.002 |
| SSO0825 | 0.133 | 0.599 | 1.809 | 2.566 |
| SSO0827 | NA | 0.198 | NA | -0.068 |
| SSO0828 | -0.086 | 0.263 | -0.193 | NA |
| SSO0830 | 0.008 | -0.228 | 0.132 | 0.396 |
| SSO0833 | -0.738 | 0.068 | -0.157 | 0.124 |
| SSO0838 | -0.125 | -0.008 | -0.023 | 0.244 |
| SSO0840 | -0.098 | 0.287 | -0.045 | 0.168 |
| SSO0842 | 0.034 | -0.384 | 0.172 | 0.032 |
| SSO0845 | 0.242 | 0.469 | 0.375 | 0.501 |
| SSO0846 | 0.234 | NA | NA | 0.136 |
| SSO0847 | -0.068 | -0.045 | 0.026 | -0.061 |
| SSO0848 | 0.42 | 0.078 | 0.219 | 0.084 |
| SSO0849 | 0.326 | 0.133 | 0.146 | 0.187 |
| SSO0852 | 0.513 | 0.285 | 0.161 | 0.626 |
| SSO0857 | -0.48 | -0.818 | -0.26 | -0.583 |
| SSO0858 | -0.298 | -0.657 | -1.691 | -1.157 |
| SSO0860 | 0.281 | 0.118 | 0.231 | 0.183 |
| SSO0862 | -0.444 | -0.717 | -0.23 | -0.548 |
| SSO0863 | 0.241 | 0.193 | 0.237 | -0.048 |
| SSO0866 | -0.017 | -0.423 | -0.348 | -0.372 |
| SSO0867 | -0.439 | -0.342 | -0.17 | 0.026 |
| SSO0870 | 0.233 | -0.058 | 0.26 | 0.051 |
| SSO0871 | 0.071 | 0.115 | -0.184 | -0.07 |
| SSO0872 | -0.075 | -0.215 | 0.812 | 0.545 |
| SSO0873 | -0.047 | NA | 0.074 | -0.096 |
| SSO0874 | -0.458 | -0.141 | -0.043 | 0.001 |
| SSO0876 | 0.16 | -0.486 | 0.619 | -0.192 |
| SSO0878 | 0.45 | -0.055 | 0.135 | -0.172 |
| SSO0879 | 0.179 | -0.222 | 0.211 | 0.048 |
| SSO0881 | -0.037 | -0.693 | -1.043 | -0.922 |
| SSO0883 | -0.12 | -0.112 | -0.041 | -0.063 |
| SSO0886 | NA | 0.017 | NA | NA |
| SSO0887 | -0.165 | 0.04 | -0.148 | -0.097 |
| SSO0888 | 0.583 | 0.508 | 0.204 | 0.18 |
| SSO0889 | 0.308 | -0.474 | 0.302 | -0.346 |
| SSO0890 | 0.095 | -0.061 | 0.24 | -0.096 |
| SSO0892 | 0.099 | -0.327 | 0.235 | -0.035 |
| SSO0893 | 0.317 | 0.129 | 0.366 | 0.163 |
| SSO0895 | -0.139 | -0.154 | 0.252 | 0.231 |
| SSO0897 | 0.11 | 0.242 | 0.247 | 0.017 |
| SSO0898 | 0.283 | -0.013 | 0.048 | -0.097 |
| SSO0899 | -0.386 | -0.994 | -0.142 | -0.461 |
| SSO0900 | 0.32 | -0.147 | 0.096 | -0.207 |
| SSO0901 | 0.267 | 0.337 | 0.122 | 0.225 |
| SSO0902 | NA | NA | 0.243 | NA |
| SSO0903 | -0.086 | 0.241 | 0.279 | -0.012 |
| SSO0904 | 0 | 0.01 | 0.158 | -0.125 |
| SSO0905 | 0.387 | -0.38 | -0.073 | -0.564 |
| SSO0906 | 0.481 | -0.129 | 0.166 | -0.133 |
| SSO0907 | 0.22 | -0.006 | 0.45 | 0.128 |
| SSO0909 | 0.002 | -0.808 | -1.009 | -1.031 |
| SSO0911 | -0.356 | -1.638 | -2.079 | -1.773 |
| SSO0913 | -0.101 | -0.33 | -0.138 | -0.363 |
| SSO0914 | -0.053 | NA | -0.189 | -0.29 |
| SSO0915 | 0.686 | 0.477 | -0.552 | -0.233 |
| SSO0916 | 0.354 | 0.058 | -0.047 | -0.073 |
| SSO0917 | 0.152 | -0.226 | 0.265 | -0.156 |
| SSO0918 | 0.35 | -0.107 | 0.309 | -0.132 |
| SSO0919 | 0.286 | -0.168 | -0.027 | -0.299 |
| SSO0920 | 0.171 | -0.223 | -0.126 | -0.084 |
| SSO0921 | -0.051 | -0.227 | 0.187 | -0.048 |
| SSO0922 | -0.019 | -0.249 | -0.036 | -0.064 |
| SSO0923 | -0.163 | -0.432 | 0.102 | -0.451 |
| SSO0925 | 0.752 | 0.299 | -0.162 | -0.3 |
| SSO0927 | 1.002 | -0.284 | 0.262 | -0.497 |
| SSO0928 | 0.458 | 0.227 | 0.184 | 0.037 |
| SSO0929 | -0.218 | -0.473 | 0.534 | -0.262 |
| SSO0931 | 0.005 | 0.507 | 0.506 | 0.466 |
| SSO0934 | 0.026 | -0.025 | 0.179 | 0.294 |
| SSO0936 | -0.241 | -0.852 | -0.104 | -0.62 |
| SSO0938 | 0.022 | -0.587 | -0.029 | -0.317 |
| SSO0939 | NA | 0.787 | NA | NA |
| SSO0940 | -0.002 | -0.19 | -0.14 | -0.137 |
| SSO0941 | 0.405 | -0.196 | -0.048 | -0.302 |
| SSO0942 | 0.445 | -0.269 | -0.675 | -0.743 |
| SSO0943 | 0.455 | -0.263 | 0.11 | 0.049 |
| SSO0944 | -0.031 | -0.139 | 0.148 | 0.047 |
| SSO0946 | -0.708 | -1.608 | -1.264 | -1.13 |
| SSO0947 | 0.049 | 0.159 | 0.112 | NA |
| SSO0949 | 0.352 | 0.127 | -0.075 | 0.223 |
| SSO0950 | 0.061 | 0.315 | 0.252 | 0.444 |
| SSO0951 | -0.139 | 0.196 | -0.055 | -0.073 |
| SSO0952 | 0.224 | -0.068 | 0.122 | -0.036 |
| SSO0953 | 0.256 | 0.076 | 0.14 | 0.227 |
| SSO0954 | 0.219 | 0.08 | -0.148 | -0.029 |
| SSO0955 | 0.212 | 0.037 | 0.163 | 0.08 |
| SSO0956 | 0.376 | -0.337 | -0.125 | -0.434 |
| SSO0957 | 0.407 | -0.312 | 0.12 | -0.159 |
| SSO0959 | NA | NA | NA | -0.078 |
| SSO0960 | 0.721 | 0.574 | 0.603 | 0.928 |
| SSO0961 | 0.025 | 0.114 | 0.236 | 0.324 |
| SSO0962 | -0.199 | 0.145 | -0.446 | -0.071 |
| SSO0963 | -0.08 | -0.313 | -0.023 | -0.391 |
| SSO0965 | -0.344 | -1.183 | -0.441 | -1.158 |
| SSO0966 | 0.361 | 0.099 | 0.541 | 0.104 |
| SSO0967 | 0.409 | -0.132 | 0.378 | 0.036 |
| SSO0968 | 0.162 | -0.572 | 1.168 | 0.765 |
| SSO0969 | -0.08 | -0.319 | 0.384 | 0.316 |
| SSO0970 | -0.233 | -0.028 | -0.058 | 0.089 |
| SSO0971 | 0.271 | -0.347 | 0.208 | -0.393 |
| SSO0972 | 0.368 | -0.244 | 0.174 | -0.076 |
| SSO0974 | 0.246 | 0.089 | 0.118 | 0.078 |
| SSO0975 | 0.255 | -0.114 | 0.08 | 0.012 |
| SSO0976 | -0.094 | 0.194 | 0.072 | -0.121 |
| SSO0977 | -0.079 | -0.419 | -0.02 | -0.381 |
| SSO0978 | 0.196 | -0.039 | -0.01 | -0.133 |
| SSO0981 | 0.092 | -0.161 | 0.218 | 0.116 |
| SSO0983 | -0.076 | -0.471 | -0.221 | -0.521 |
| SSO0985 | 0.532 | 0.038 | 0.13 | 0.203 |
| SSO0986 | -0.083 | -0.302 | 0.446 | 0.589 |
| SSO0987 | 0.415 | -0.216 | -0.131 | -0.21 |
| SSO0988 | -0.078 | -0.462 | -0.256 | -0.512 |
| SSO0989 | 0.333 | -0.329 | -0.391 | -0.6 |
| SSO0990 | 0.771 | 0.031 | -0.011 | -0.151 |
| SSO0991 | 0.025 | -0.151 | 0.182 | 0.005 |
| SSO0992 | 0.058 | 0.003 | -0.04 | 0.229 |
| SSO0994 | 0.202 | -0.202 | 0.193 | 0.129 |
| SSO0995 | 0.462 | 0.151 | 0.182 | -0.032 |
| SSO0996 | 0.307 | -0.017 | 0.46 | 0.44 |
| SSO0997 | 0.273 | 0.192 | 0.529 | 0.985 |
| SSO0998 | 0.012 | 0.058 | 0.172 | 0.273 |
| SSO0999 | -0.269 | -0.553 | 0.117 | -0.191 |
| SSO1001 | 0.171 | 0.042 | 0.328 | 0.406 |
| SSO1003 | -0.374 | -1.052 | -0.129 | -0.543 |
| SSO1004 | -0.097 | -0.42 | 0.169 | -0.556 |
| SSO1005 | 0.19 | -0.082 | 0.099 | -0.165 |
| SSO10051 | -0.076 | -0.561 | -0.425 | -0.614 |
| SSO1008 | 0.015 | -0.071 | 0.109 | NA |
| SSO1009 | -0.165 | 0.023 | 0.059 | 0.246 |
| SSO1010 | 0.073 | -0.222 | -0.145 | -0.289 |
| SSO1012 | 0.319 | -0.076 | 0.076 | -0.06 |
| SSO1018 | -0.462 | -0.507 | -0.386 | -0.28 |
| SSO1019 | -1.318 | -0.492 | -0.614 | -0.638 |
| SSO1020 | -0.193 | NA | 0.082 | 0.111 |
| SSO1022 | -0.506 | -0.623 | -0.2 | -0.389 |
| SSO10224 | -0.095 | NA | NA | NA |
| SSO1023 | -0.089 | -0.227 | NA | -0.331 |
| SSO10237 | -0.156 | NA | NA | -0.073 |
| SSO1024 | -0.505 | -0.669 | -0.268 | -0.555 |
| SSO1025 | -0.35 | NA | -0.142 | 0.023 |
| SSO10258 | 0.251 | NA | NA | 0.435 |
| SSO1026 | -0.853 | -0.181 | -0.09 | -0.01 |
| SSO10269 | NA | -0.081 | NA | NA |
| SSO1027 | -0.201 | 0.153 | -1.526 | -1.197 |
| SSO1028 | -0.271 | -0.132 | 0.018 | -0.142 |
| SSO10285 | 0.244 | -0.088 | -0.254 | -0.246 |
| SSO1029 | 0.347 | -0.212 | 0.103 | -0.179 |
| SSO1030 | 0.285 | 0.017 | -0.155 | -0.092 |
| SSO1031 | 0.158 | 0.089 | 0.428 | 0.342 |
| SSO1032 | 0.113 | -0.026 | -0.019 | 0.054 |
| SSO1034 | 0.169 | -0.157 | NA | 0.289 |
| SSO10340 | -0.253 | -0.127 | -0.307 | -0.235 |
| SSO10342 | 0.15 | NA | -0.534 | NA |
| SSO10348 | NA | NA | NA | 0.173 |
| SSO1035 | 0.406 | -0.064 | 0.163 | -0.129 |
| SSO1036 | 0.414 | 0.873 | 0.454 | 0.87 |
| SSO1038 | 0.132 | -0.41 | -0.307 | -0.287 |
| SSO1039 | -0.078 | -0.303 | 0.114 | 0.244 |
| SSO1040 | -0.093 | -0.138 | -0.075 | -0.258 |
| SSO1041 | 0.281 | 0.321 | 0.166 | 0.243 |
| SSO1042 | 0.21 | 0.041 | 0.615 | -0.045 |
| SSO1043 | 0.237 | -0.083 | 0.015 | 0.012 |
| SSO1044 | 0.426 | -0.129 | 0.049 | -0.195 |
| SSO10449 | 0.039 | 0.122 | -0.403 | -0.294 |
| SSO1045 | -0.117 | -0.477 | -0.525 | -0.65 |
| SSO1046 | 0.155 | 0.617 | 0.453 | 0.593 |
| SSO1047 | 0.367 | 0.27 | 0.067 | 0.481 |
| SSO1048 | 0.591 | -0.043 | 0.186 | 0.022 |
| SSO1049 | 0.25 | -0.178 | NA | NA |
| SSO1050 | -0.026 | -0.391 | 0.07 | -0.247 |
| SSO1052 | 0.503 | -0.157 | 0.409 | -0.108 |
| SSO1053 | 0.519 | 0.643 | 2.647 | 2.748 |
| SSO1054 | 0.657 | 0.406 | 0.24 | 0.664 |
| SSO1055 | 0.102 | -0.162 | -0.215 | -0.23 |
| SSO1056 | 0.17 | -0.172 | 0.072 | -0.208 |
| SSO1057 | -0.406 | -0.148 | -0.026 | 0.168 |
| SSO1060 | 0.373 | -0.232 | -0.176 | -0.498 |
| SSO1061 | 0.396 | -0.179 | -0.115 | 0.045 |
| SSO1062 | 0.404 | 0.187 | 0.282 | 0.204 |
| SSO1063 | -0.34 | -0.725 | -0.526 | -0.538 |
| SSO1064 | 0.254 | 0.373 | -0.142 | -0.182 |
| SSO1065 | 0.292 | -0.012 | -0.103 | -0.331 |
| SSO1066 | 0.176 | -0.051 | -0.096 | -0.109 |
| SSO1067 | 0.158 | 0.097 | 0.012 | 0.165 |
| SSO1068 | 0.059 | NA | 0.256 | 0.427 |
| SSO1069 | 0.194 | -0.029 | -0.01 | -0.123 |
| SSO10704 | 0.048 | 0.073 | -1.247 | -1.011 |
| SSO1074 | -0.209 | -0.034 | -0.305 | -0.435 |
| SSO1075 | 0.104 | -0.117 | -0.231 | -0.161 |
| SSO1076 | -0.112 | 0.112 | 0.488 | 0.299 |
| SSO1077 | -0.153 | 0.111 | -0.08 | 0.023 |
| SSO1078 | 0.107 | -0.204 | -0.041 | -0.357 |
| SSO10784 | -0.18 | NA | -0.164 | -0.15 |
| SSO10788 | 0.322 | -0.017 | 0.059 | -0.072 |
| SSO1079 | 0.297 | -0.075 | 0.005 | 0.018 |
| SSO10802 | 0.179 | -0.067 | 0.1 | -0.044 |
| SSO1082 | -0.376 | -0.537 | -0.232 | -0.367 |
| SSO10828 | 0.067 | 0.071 | 0.163 | -0.003 |
| SSO1083 | -0.485 | -0.12 | -0.127 | -0.034 |
| SSO1085 | 0.002 | 0.071 | -0.093 | -0.027 |
| SSO1086 | -0.451 | 0.006 | -0.15 | 0.267 |
| SSO1089 | 0.058 | -0.696 | -1.176 | -1.053 |
| SSO1090 | 0.438 | 0.192 | 0.123 | 0.355 |
| SSO1091 | -0.354 | -0.43 | 0.03 | -0.311 |
| SSO1092 | -0.611 | -0.462 | -0.053 | -0.141 |
| SSO1093 | 0.123 | -0.05 | 0.027 | -0.309 |
| SSO1095 | -0.193 | -0.354 | 0.187 | -0.35 |
| SSO1096 | 0.14 | 0.072 | 0.015 | 0.012 |
| SSO1098 | -0.429 | -0.48 | -0.899 | -0.583 |
| SSO1100 | -0.45 | -0.351 | -0.564 | -0.345 |
| SSO1101 | -0.575 | -0.969 | -0.702 | -0.678 |
| SSO1102 | 0.304 | 0.089 | 0.108 | -0.05 |
| SSO11020 | 0.128 | 0.092 | -0.318 | -0.147 |
| SSO1104 | 0.061 | 0.124 | 0.202 | NA |
| SSO1105 | 0.306 | -0.008 | -0.122 | -0.181 |
| SSO1106 | -0.016 | -0.07 | -0.137 | -0.154 |
| SSO1107 | 0.558 | -0.11 | -0.16 | -0.158 |
| SSO11071 | 0.615 | 0.127 | 0.765 | 0.356 |
| SSO1108 | -0.441 | -0.345 | -0.585 | -0.697 |
| SSO1109 | 0.137 | 0.681 | 0.293 | 0.579 |
| SSO1110 | 0.16 | -0.22 | -0.152 | -0.233 |
| SSO1111 | 0.367 | 0.123 | -0.15 | -0.145 |
| SSO11114 | 0.046 | -0.115 | -0.118 | -0.465 |
| SSO1112 | 0.382 | 0.039 | 0.518 | -0.012 |
| SSO11133 | -0.239 | -0.325 | -0.001 | -0.033 |
| SSO1116 | -0.143 | NA | NA | NA |
| SSO1117 | 0.523 | 0.043 | 0.066 | 0.064 |
| SSO1118 | NA | NA | 0.039 | NA |
| SSO1119 | -0.603 | -0.819 | -0.31 | -0.534 |
| SSO11196 | 0.419 | -0.073 | 0.077 | -0.156 |
| SSO1120 | 0.67 | 0.269 | 0.322 | 0.267 |
| SSO1121 | 0.318 | NA | -0.098 | NA |
| SSO1123 | 1.042 | 0.131 | 0.222 | -0.134 |
| SSO11231 | 0.21 | 0.06 | 0.059 | -0.089 |
| SSO1125 | 0.818 | 0.655 | 0.117 | 0.42 |
| SSO1126 | 0.391 | 0.368 | -0.178 | 0.235 |
| SSO1127 | 1.414 | 0.817 | 0.882 | 0.83 |
| SSO1129 | 1.404 | 0.401 | 0.648 | 0.351 |
| SSO1131 | 1.154 | 0.773 | 1.117 | 1.031 |
| SSO1133 | 0.598 | 0 | 0.354 | 0.161 |
| SSO1134 | 1.09 | 0.087 | 0.439 | 0.165 |
| SSO1135 | 1.258 | 0.441 | 0.327 | 0.389 |
| SSO1137 | -0.077 | -0.201 | -0.042 | -0.124 |
| SSO1138 | 0.134 | -0.127 | 0.138 | 0.035 |
| SSO11387 | 0.264 | 0.085 | -0.233 | -0.067 |
| SSO1140 | 0.624 | 0.219 | 0.213 | 0.204 |
| SSO1141 | 0.21 | -0.359 | -0.232 | -0.232 |
| SSO11412 | -0.174 | -0.047 | 0.074 | 0.147 |
| SSO1144 | 0.381 | 0.175 | -0.027 | 0.105 |
| SSO1145 | 0.223 | 0.071 | 0.446 | 0.353 |
| SSO1147 | 0.412 | 0.09 | 0.236 | 0.328 |
| SSO1148 | 0.182 | -0.046 | -0.33 | -0.063 |
| SSO1149 | 0.862 | 0.566 | 0.427 | 0.652 |
| SSO1150 | 0.355 | 0.342 | -0.058 | 0.184 |
| SSO1151 | 0.713 | -0.16 | 0.032 | -0.209 |
| SSO1152 | 0.029 | -0.094 | 0.307 | 0.329 |
| SSO1153 | 0.207 | -0.155 | -0.031 | -0.27 |
| SSO1154 | 0.04 | 0.339 | -0.233 | 0.135 |
| SSO1155 | -0.144 | -0.104 | 0.388 | 0.175 |
| SSO1157 | 0.318 | 0.103 | 0.18 | 0.419 |
| SSO11571 | NA | 0.401 | NA | 0 |
| SSO11575 | NA | 0.365 | NA | NA |
| SSO1160 | -0.372 | -0.304 | -0.022 | -0.344 |
| SSO1161 | 0.105 | 0.202 | 1.299 | 1.862 |
| SSO11614 | 0.077 | -0.122 | -0.349 | -0.28 |
| SSO1162 | 0.774 | 0.447 | 0.626 | 0.706 |
| SSO1163 | 0.412 | 0.341 | 0.692 | 1.081 |
| SSO11637 | 0.465 | -0.048 | 0.068 | 0.078 |
| SSO1167 | NA | NA | NA | 0.645 |
| SSO1168 | 0.04 | -0.301 | -0.095 | -0.456 |
| SSO1169 | 0.197 | -0.5 | -0.087 | -0.36 |
| SSO1170 | 0.043 | NA | -0.062 | 0.096 |
| SSO1171 | -0.125 | -0.372 | -0.398 | -0.498 |
| SSO1172 | -0.041 | -0.391 | 0.278 | 0.373 |
| SSO1173 | 0.226 | 0.147 | -0.182 | -0.16 |
| SSO1174 | 0.216 | 0.113 | -0.195 | 0.005 |
| SSO1175 | -0.261 | -0.823 | -0.075 | -0.416 |
| SSO1176 | 0.542 | -0.354 | -0.586 | -0.656 |
| SSO1177 | 0.098 | -0.094 | 0.331 | 0.023 |
| SSO1178 | -0.3 | -0.095 | -0.293 | -0.35 |
| SSO1183 | NA | NA | 0.443 | 0.514 |
| SSO1185 | -0.196 | -0.019 | -0.233 | -0.077 |
| SSO1186 | 0.076 | 0.037 | 0.181 | 0.287 |
| SSO1188 | 0.035 | -0.123 | 0.389 | 0.126 |
| SSO1189 | 0.148 | 0.009 | 0.04 | -0.07 |
| SSO1191 | -0.079 | -0.136 | 0.259 | 0.466 |
| SSO11914 | 0.193 | -0.281 | -0.835 | -0.721 |
| SSO1192 | 0.035 | -0.137 | 0.211 | 0.158 |
| SSO11920 | 0.579 | -0.05 | 0.199 | -0.454 |
| SSO1193 | NA | NA | NA | 0.53 |
| SSO11934 | -0.495 | -0.378 | -0.087 | -0.343 |
| SSO11939 | -0.549 | -0.483 | -0.255 | -0.5 |
| SSO1195 | 0.069 | -0.209 | -0.099 | -0.227 |
| SSO1196 | -0.243 | NA | NA | NA |
| SSO11972 | -0.287 | -0.17 | -0.346 | -0.184 |
| SSO11979 | 0.263 | NA | 0.068 | NA |
| SSO12018 | 0.016 | 0.137 | -0.258 | -0.213 |
| SSO1206 | 0.005 | -0.165 | 0.693 | 0.659 |
| SSO1207 | 0.188 | -0.039 | 0.444 | 0.613 |
| SSO1208 | 0.105 | 0.058 | 0.401 | 0.486 |
| SSO12083 | -0.111 | 0.003 | -0.054 | 0.011 |
| SSO1209 | 0.088 | 0.267 | 0.727 | 0.278 |
| SSO12093 | 0.212 | NA | NA | 0.624 |
| SSO1210 | 0.053 | -0.05 | 0.133 | -0.021 |
| SSO12127 | -0.298 | -0.062 | 0.354 | -0.537 |
| SSO1213 | -0.288 | -0.105 | -0.039 | 0.243 |
| SSO1214 | 0.105 | 0.171 | 0.465 | 0.296 |
| SSO12142 | -0.066 | 0.087 | -0.085 | -0.041 |
| SSO1215 | NA | NA | NA | -0.171 |
| SSO1218 | 0.409 | -0.032 | 0.459 | 0.73 |
| SSO1219 | 0.313 | 0.049 | 0.268 | -0.007 |
| SSO12199 | 0.243 | -0.025 | -0.118 | -0.378 |
| SSO1220 | NA | NA | NA | 0.258 |
| SSO1221 | 0.2 | 0.163 | 0.26 | 0.108 |
| SSO1223 | 0.197 | 0.271 | 0.596 | 0.402 |
| SSO1224 | NA | NA | -0.189 | 0.272 |
| SSO1225 | 0.407 | -0.015 | 0.407 | 0.118 |
| SSO12252 | -0.12 | -0.209 | -0.75 | -0.429 |
| SSO12256 | 0.039 | -0.157 | -0.005 | 0.021 |
| SSO1227 | 0.026 | 1.14 | 0.731 | 0.851 |
| SSO1229 | NA | -0.019 | NA | -0.067 |
| SSO1231 | 0.253 | NA | NA | NA |
| SSO1233 | 0.291 | -0.158 | 0.118 | 0.127 |
| SSO1234 | -0.071 | NA | NA | NA |
| SSO1251 | -0.445 | -0.491 | -0.227 | -0.257 |
| SSO1252 | 0.006 | -0.142 | -0.216 | -0.334 |
| SSO1253 | -0.184 | -0.418 | -0.574 | -0.588 |
| SSO1254 | -0.239 | -0.399 | -0.164 | -0.389 |
| SSO1255 | -0.36 | -0.485 | -0.486 | -0.52 |
| SSO1256 | -0.725 | -1.495 | -1.483 | -1.7 |
| SSO1258 | -0.149 | -0.218 | -0.275 | -0.186 |
| SSO1260 | -0.465 | -0.515 | -0.394 | -0.648 |
| SSO1261 | 0.031 | -0.149 | -0.224 | -0.217 |
| SSO1262 | 0.255 | -0.174 | NA | -0.134 |
| SSO1265 | 0.177 | -0.158 | -0.08 | 0.056 |
| SSO1266 | -0.044 | -0.295 | -0.319 | -0.312 |
| SSO1268 | 0.012 | -0.448 | -0.073 | -0.219 |
| SSO1270 | 0.098 | -0.29 | -0.217 | -0.314 |
| SSO1271 | -0.103 | -0.206 | -0.431 | -0.263 |
| SSO1272 | 0.212 | -0.304 | -0.347 | -0.408 |
| SSO1273 | -0.09 | -0.912 | 0.157 | -0.712 |
| SSO1274 | -0.035 | -0.319 | -0.227 | -0.544 |
| SSO1275 | 0.119 | -0.243 | -0.314 | -0.547 |
| SSO1276 | -0.256 | -0.466 | -0.293 | -0.691 |
| SSO1277 | -0.132 | -0.472 | -0.275 | -0.441 |
| SSO1279 | NA | NA | NA | 0.187 |
| SSO1281 | -0.311 | -0.399 | -0.527 | -0.39 |
| SSO1282 | -0.301 | -0.858 | -0.77 | -0.888 |
| SSO1283 | -0.057 | -0.715 | -0.419 | -0.62 |
| SSO1284 | -0.035 | -0.321 | -0.242 | -0.449 |
| SSO1287 | -0.488 | -0.906 | -1.001 | -1.185 |
| SSO1288 | -0.042 | -0.296 | -1.364 | -1.101 |
| SSO1289 | -0.019 | -0.124 | 0.28 | 0.179 |
| SSO1290 | -0.349 | -0.472 | -0.864 | -0.831 |
| SSO1291 | -0.133 | -0.437 | -0.261 | -0.258 |
| SSO1292 | 0.516 | 0.152 | -0.065 | -0.091 |
| SSO1294 | -0.085 | -0.263 | -0.189 | -0.107 |
| SSO1296 | NA | 0.39 | -0.056 | NA |
| SSO1297 | 0.416 | -0.266 | 0.051 | -0.009 |
| SSO1299 | -0.081 | -0.341 | NA | 0.035 |
| SSO1300 | 0.156 | -0.404 | -0.143 | -0.503 |
| SSO1303 | -0.014 | 0.319 | 0.144 | 0.094 |
| SSO1305 | -0.01 | NA | NA | NA |
| SSO1306 | 0.16 | -0.253 | -0.204 | -0.284 |
| SSO1307 | -0.111 | 0.005 | -0.085 | -0.768 |
| SSO1310 | -0.184 | NA | NA | 0.16 |
| SSO1311 | -0.325 | -0.218 | 0.774 | 0.338 |
| SSO1312 | -0.505 | 0.584 | 1.192 | 0.951 |
| SSO1313 | 0.047 | 0.042 | 0.55 | 0.637 |
| SSO1314 | 0.299 | -0.024 | 0.628 | 0.463 |
| SSO1318 | NA | NA | NA | -0.401 |
| SSO1319 | 0.28 | -0.127 | -0.176 | -0.258 |
| SSO1320 | 0.124 | 0.427 | 0.147 | 0.332 |
| SSO1321 | -0.068 | -0.281 | -0.025 | 0.011 |
| SSO1322 | -0.728 | -0.057 | -0.652 | -0.304 |
| SSO1325 | -0.335 | -0.471 | -0.101 | -0.277 |
| SSO1327 | -0.017 | -0.159 | 0.09 | 0.1 |
| SSO1329 | -0.037 | -0.272 | 0.203 | -0.045 |
| SSO1330 | -0.146 | 0.211 | -0.195 | -0.091 |
| SSO1331 | 0.14 | -0.297 | -0.254 | -0.267 |
| SSO1333 | -0.113 | 0.072 | 0.54 | 0.273 |
| SSO1334 | -0.723 | -0.519 | 0.068 | -0.316 |
| SSO1338 | -0.064 | NA | 0.332 | 0.446 |
| SSO1340 | -0.149 | -0.259 | 0.05 | 0.076 |
| SSO1342 | NA | 0.365 | 0.172 | 0.239 |
| SSO1344 | -0.635 | -0.283 | 0.003 | -0.081 |
| SSO1345 | 0.081 | -0.047 | -0.088 | -0.105 |
| SSO1346 | -0.331 | -0.091 | -0.147 | -0.203 |
| SSO1351 | 0.86 | 1.255 | 2.121 | 1.997 |
| SSO1352 | 0.366 | 0.278 | 1.49 | 1.084 |
| SSO1353 | -0.008 | -0.679 | -0.184 | -0.408 |
| SSO1354 | NA | 0.406 | -0.21 | NA |
| SSO1356 | -0.231 | -0.322 | -0.018 | -0.148 |
| SSO1357 | -0.489 | -0.306 | -0.727 | -0.462 |
| SSO1359 | NA | NA | NA | 0.297 |
| SSO1360 | -0.016 | -0.075 | -0.223 | -0.028 |
| SSO1365 | 0.257 | NA | NA | 0.163 |
| SSO1369 | NA | -0.156 | 0.347 | 0.249 |
| SSO1373 | -0.551 | 0.208 | 0.267 | 0.465 |
| SSO1375 | 0.451 | 0.174 | 0.507 | 0.659 |
| SSO1378 | NA | NA | -2.372 | NA |
| SSO1380 | NA | 0.168 | 0.362 | 0.057 |
| SSO1383 | -0.098 | -0.152 | 0.051 | 0.304 |
| SSO1388 | -1.033 | -1.73 | -0.724 | -1.217 |
| SSO1389 | 0.231 | 0.078 | 0.052 | 0.002 |
| SSO1391 | -0.065 | -0.067 | 0.381 | 0.176 |
| SSO1392 | -0.113 | -0.147 | 0.213 | 0.124 |
| SSO1393 | -0.208 | -0.227 | -0.067 | -0.184 |
| SSO1395 | -0.321 | -0.128 | 0.068 | -0.085 |
| SSO1397 | -0.122 | 0.297 | -0.005 | -0.111 |
| SSO1398 | -0.165 | -0.307 | -0.347 | -0.504 |
| SSO1399 | -0.891 | -0.769 | -0.602 | -1.096 |
| SSO1400 | 0.043 | -0.374 | -0.229 | -0.455 |
| SSO1401 | -0.133 | -0.367 | -0.239 | -0.431 |
| SSO1403 | 0.11 | -0.152 | -0.237 | -0.186 |
| SSO1404 | 0.006 | -0.33 | -0.213 | -0.192 |
| SSO1405 | -0.134 | -0.741 | -0.245 | -0.106 |
| SSO1406 | -0.02 | -0.03 | 0.178 | 0.215 |
| SSO1409 | NA | NA | NA | -0.038 |
| SSO1412 | 0.057 | -0.04 | -0.158 | -0.037 |
| SSO1418 | 0.154 | -0.041 | 0.604 | 0.304 |
| SSO1419 | -0.551 | -0.963 | -0.319 | -0.547 |
| SSO1421 | -0.845 | -0.365 | -0.013 | 0.079 |
| SSO1422 | -0.385 | NA | -0.027 | 0.136 |
| SSO1423 | -0.671 | -0.612 | -0.17 | -0.339 |
| SSO1424 | -0.642 | -0.397 | -0.063 | -0.124 |
| SSO1426 | -0.372 | -0.44 | -0.031 | -0.082 |
| SSO1427 | -0.1 | -0.429 | -0.033 | -0.332 |
| SSO1428 | -0.163 | -0.711 | -0.005 | -0.31 |
| SSO1429 | -0.084 | -0.453 | -0.114 | -0.23 |
| SSO1431 | -0.285 | -0.428 | 0.475 | 0.503 |
| SSO1432 | -0.282 | -0.603 | 0.227 | -0.039 |
| SSO1433 | -0.663 | 0.139 | 0.101 | 0.241 |
| SSO1435 | -0.76 | -0.704 | -0.482 | -0.406 |
| SSO1437 | 0.046 | -0.421 | 0.241 | -0.008 |
| SSO1438 | -0.48 | -0.248 | 0.249 | 0.21 |
| SSO1439 | -0.278 | -0.286 | 0.142 | -0.124 |
| SSO1440 | 0.145 | NA | 0.111 | NA |
| SSO1441 | -0.402 | -0.299 | 0.194 | -0.069 |
| SSO1444 | -0.296 | -0.13 | -0.138 | -0.239 |
| SSO1445 | -0.157 | -0.413 | 0.055 | -0.016 |
| SSO1449 | 0.087 | 0.089 | 0.362 | 0.317 |
| SSO1450 | NA | NA | 0.785 | 0.404 |
| SSO1451 | 0.04 | 0.274 | 0.867 | 1.21 |
| SSO1456 | NA | 0 | NA | NA |
| SSO1458 | 0.334 | 1.217 | 2.366 | 1.979 |
| SSO1459 | 0.359 | 0.819 | 2.212 | 1.467 |
| SSO1460 | 0.163 | 0.133 | 0.573 | 0.577 |
| SSO1462 | NA | NA | NA | 0.064 |
| SSO1463 | 0.242 | 0.146 | 0.354 | 0.327 |
| SSO1464 | -0.123 | 0.153 | 0.277 | 0.597 |
| SSO1465 | -0.262 | -0.2 | -0.015 | 0.12 |
| SSO1468 | -0.426 | -0.321 | 0.315 | 0.039 |
| SSO1469 | -0.49 | 0.069 | 0.121 | -0.061 |
| SSO1470 | -0.152 | 0.005 | -0.043 | -0.15 |
| SSO1471 | -0.63 | -0.236 | 0.031 | -0.107 |
| SSO1475 | NA | NA | 0.703 | 0.158 |
| SSO1483 | -0.553 | 0.169 | 0.605 | 0.513 |
| SSO1489 | -0.858 | -0.284 | -0.689 | -0.502 |
| SSO1491 | -0.15 | -0.044 | 0.209 | 0.015 |
| SSO1493 | -0.017 | -0.26 | 0.41 | -0.361 |
| SSO1494 | -0.087 | -0.189 | -0.169 | -0.144 |
| SSO1495 | -1.095 | 0.18 | -0.033 | 0.176 |
| SSO1500 | -0.735 | -0.627 | 0.019 | -0.527 |
| SSO1501 | -0.579 | 0.664 | 1.284 | 1.098 |
| SSO1503 | -0.293 | 0.291 | 0.358 | 0.543 |
| SSO1505 | -0.017 | -0.116 | 0.018 | 0.113 |
| SSO1510 | -0.503 | -0.669 | -0.024 | -0.36 |
| SSO1512 | -0.883 | -0.929 | 0.179 | -0.45 |
| SSO1513 | -0.277 | -0.225 | -0.047 | -0.111 |
| SSO1514 | -1.24 | -0.357 | 0.073 | -0.137 |
| SSO1515 | -0.977 | -0.405 | -0.523 | -0.546 |
| SSO1519 | -0.214 | -0.241 | -0.36 | -0.537 |
| SSO1520 | NA | NA | 0.012 | NA |
| SSO1522 | -0.448 | -0.112 | -0.168 | -0.005 |
| SSO1523 | -0.036 | -0.049 | 0.27 | 0.095 |
| SSO1524 | -0.712 | -0.385 | -0.198 | -0.52 |
| SSO1526 | -0.417 | -0.411 | 0.336 | 0.003 |
| SSO1527 | -0.065 | -0.418 | 0.107 | -0.096 |
| SSO1529 | -0.368 | -0.597 | -0.333 | -0.485 |
| SSO1530 | -0.531 | -0.764 | -0.125 | -0.381 |
| SSO1531 | -0.824 | -0.921 | -0.172 | -0.431 |
| SSO1532 | -0.866 | -0.188 | 0.128 | 0.145 |
| SSO1533 | -0.811 | -0.334 | 0.127 | 0.351 |
| SSO1535 | -0.069 | 0.11 | 1.993 | 2.73 |
| SSO1536 | -0.035 | 0.149 | 0.346 | 0.13 |
| SSO1537 | -0.586 | -0.273 | -0.034 | -0.134 |
| SSO1538 | -0.008 | -0.148 | 0.309 | 0.313 |
| SSO1539 | -0.172 | -0.129 | -0.03 | 0.106 |
| SSO1540 | -0.666 | -0.963 | 0.079 | -0.011 |
| SSO1542 | -0.239 | -0.59 | -0.227 | -0.121 |
| SSO1545 | -0.464 | -0.223 | 0.192 | 0.024 |
| SSO1550 | -0.125 | NA | 0.021 | 0.396 |
| SSO1552 | -0.212 | -0.503 | -0.128 | -0.256 |
| SSO1555 | -0.389 | 0.176 | 0.123 | 0.32 |
| SSO1560 | -0.248 | -0.351 | -0.212 | -0.222 |
| SSO1562 | -0.183 | 0.1 | -0.126 | 0.037 |
| SSO1563 | NA | NA | 0.187 | NA |
| SSO1565 | 0.641 | -0.045 | 0.482 | 0.647 |
| SSO1566 | 0.236 | -0.236 | -0.127 | -0.218 |
| SSO1568 | 0.02 | 0.046 | -0.02 | -0.149 |
| SSO1569 | -0.006 | NA | NA | NA |
| SSO1570 | -0.517 | 0.104 | -0.382 | -0.251 |
| SSO1571 | 0.129 | -0.044 | 0.383 | 0.109 |
| SSO1574 | NA | NA | -1.047 | -0.325 |
| SSO1577 | 0.336 | NA | 0.446 | 0.229 |
| SSO1578 | 0.178 | 0.205 | 0.737 | 1.155 |
| SSO1579 | 0.305 | -0.01 | 0.044 | 0.303 |
| SSO1580 | 1.634 | 0.261 | 1.192 | 1.994 |
| SSO1581 | NA | 0.179 | NA | NA |
| SSO1583 | -0.914 | -0.51 | -0.595 | -0.308 |
| SSO1587 | 0.037 | 0.086 | 0.101 | 0.15 |
| SSO1588 | 0.313 | 0.178 | 0.188 | 0.658 |
| SSO1589 | -0.04 | -0.042 | -0.114 | 0.015 |
| SSO1590 | 0.184 | -0.035 | 0.101 | 0.204 |
| SSO1591 | 0.139 | -0.101 | 0.12 | 0.18 |
| SSO1593 | 0.083 | NA | 0.025 | 0.078 |
| SSO1594 | 0.048 | -0.175 | -0.124 | -0.227 |
| SSO1595 | 0.175 | -0.015 | 0.341 | 0.409 |
| SSO1597 | -0.036 | -0.517 | 0.073 | -0.375 |
| SSO1598 | -0.279 | -0.37 | 0.029 | -0.412 |
| SSO1600 | 0.244 | -0.345 | 0.292 | -0.087 |
| SSO1601 | 0.315 | 0.268 | 0.565 | 0.845 |
| SSO1608 | NA | 0.286 | NA | 0.559 |
| SSO1610 | 0.241 | 0.251 | 0.432 | 0.269 |
| SSO1611 | -0.112 | -0.025 | -0.148 | -0.136 |
| SSO1613 | 0.333 | NA | NA | NA |
| SSO1615 | NA | 0.003 | NA | NA |
| SSO1617 | NA | NA | NA | -0.714 |
| SSO1618 | 0.188 | -0.022 | -0.023 | -0.264 |
| SSO1620 | 0.203 | 0.035 | 0.102 | -0.146 |
| SSO1621 | -0.074 | -0.206 | 0.211 | 0.251 |
| SSO1622 | 0.122 | -0.156 | NA | NA |
| SSO1623 | -0.209 | -0.268 | -0.003 | -0.151 |
| SSO1624 | 0.225 | 0.155 | 0.353 | 0.33 |
| SSO1625 | -0.052 | -0.176 | 0.233 | 0.139 |
| SSO1626 | -0.934 | -0.196 | -0.022 | -0.082 |
| SSO1635 | 0.097 | -0.204 | 0.043 | -0.072 |
| SSO1636 | 0.119 | -0.272 | -0.091 | -0.134 |
| SSO1637 | NA | 0.122 | NA | NA |
| SSO1640 | -0.232 | -0.052 | NA | -0.211 |
| SSO1641 | -0.311 | -0.419 | -0.081 | -0.303 |
| SSO1643 | NA | NA | 0.21 | NA |
| SSO1645 | -0.133 | NA | NA | 0.621 |
| SSO1646 | -0.772 | -0.41 | 0.241 | -0.205 |
| SSO1651 | -0.157 | -0.138 | 0.188 | -0.177 |
| SSO1653 | -0.782 | -0.886 | -0.148 | -0.773 |
| SSO1655 | -0.096 | -0.262 | -0.071 | -0.084 |
| SSO1657 | -0.074 | -0.39 | 0.076 | -0.39 |
| SSO1662 | 0.102 | -0.409 | 0.248 | 0.066 |
| SSO1663 | -0.183 | -0.441 | -0.046 | -0.306 |
| SSO1664 | 0.054 | 0.147 | 0.222 | 0.23 |
| SSO1665 | -0.749 | -0.68 | -0.346 | -0.121 |
| SSO1666 | NA | -0.161 | NA | NA |
| SSO1667 | -0.111 | -0.45 | 0.06 | -0.124 |
| SSO1668 | 0.103 | -0.487 | 0.004 | 0.134 |
| SSO1671 | NA | NA | 0.255 | 0.127 |
| SSO1673 | -0.201 | -0.179 | 0.45 | 0.043 |
| SSO1674 | NA | NA | NA | 0.058 |
| SSO1675 | NA | -0.163 | 0.14 | -0.32 |
| SSO1676 | -0.646 | -1.066 | -0.332 | -0.88 |
| SSO1686 | -0.573 | 0.068 | 0.355 | 0.458 |
| SSO1689 | NA | NA | -0.087 | -0.125 |
| SSO1690 | -0.28 | NA | NA | NA |
| SSO1691 | -1.355 | -0.483 | -0.078 | -0.328 |
| SSO1692 | -0.043 | 0.204 | 1.453 | 1.765 |
| SSO1693 | 0.188 | 0.005 | 0.181 | 0.311 |
| SSO1701 | -1.221 | -0.926 | -0.34 | -0.293 |
| SSO1711 | -0.122 | -0.058 | -0.116 | 0.057 |
| SSO1714 | NA | NA | 0.711 | NA |
| SSO1727 | 0.099 | NA | 0.553 | NA |
| SSO1730 | 0.312 | NA | 0.178 | NA |
| SSO1739 | -0.495 | -0.246 | -0.039 | 0.117 |
| SSO1740 | -1.068 | -0.364 | -0.079 | -0.109 |
| SSO1741 | 0.237 | 0.056 | 0.589 | 0.495 |
| SSO1742 | 0.455 | 0.074 | 0.575 | 0.764 |
| SSO1746 | -0.081 | 0.232 | 0.343 | 0.498 |
| SSO1752 | -0.649 | -0.035 | 0.131 | 0.18 |
| SSO1755 | 0.542 | 0.055 | -0.084 | -0.288 |
| SSO1757 | 0.131 | 0.225 | 0.004 | 0.102 |
| SSO1759 | 0.188 | -0.065 | 0.218 | 0.232 |
| SSO1762 | 0.233 | 0.003 | 0.416 | 0.519 |
| SSO1764 | 0.01 | -0.049 | 0.072 | 0.075 |
| SSO1765 | 0 | -0.092 | NA | NA |
| SSO1767 | NA | NA | 0.659 | 0.99 |
| SSO1768 | NA | NA | NA | -0.067 |
| SSO1773 | NA | NA | NA | 0.24 |
| SSO1774 | -1.034 | 0.039 | 1.389 | 1.135 |
| SSO1777 | NA | NA | -0.054 | NA |
| SSO1780 | -0.111 | -0.026 | NA | -0.005 |
| SSO1782 | 0.162 | -0.251 | 0.111 | -0.216 |
| SSO1784 | -0.026 | -0.014 | 0.193 | -0.046 |
| SSO1785 | -0.357 | -0.092 | -0.081 | -0.183 |
| SSO1786 | -0.486 | -0.437 | -0.191 | -0.077 |
| SSO1787 | -0.734 | -0.243 | -0.214 | -0.299 |
| SSO1789 | -0.473 | -0.242 | 0.089 | 0.116 |
| SSO1790 | -0.28 | -0.289 | 0.099 | -0.162 |
| SSO1802 | -0.145 | 0.373 | 0.433 | 0.453 |
| SSO1805 | 0.201 | 0.216 | 0.259 | 0.174 |
| SSO1806 | -0.179 | 0.157 | 0.493 | 0.285 |
| SSO1807 | 0.508 | -0.52 | -0.908 | -0.487 |
| SSO1808 | -0.141 | -0.214 | -0.018 | -0.229 |
| SSO1810 | 0.428 | 0.229 | 0.531 | 0.693 |
| SSO1811 | -0.155 | -0.313 | 0.109 | 0.078 |
| SSO1812 | 0.039 | -0.08 | 0.085 | 0.108 |
| SSO1813 | 0.395 | -0.199 | -0.103 | 0.205 |
| SSO1816 | 0.065 | NA | NA | NA |
| SSO1817 | -1.376 | -1.848 | -1.49 | -1.574 |
| SSO1818 | -0.76 | -0.977 | -0.271 | -0.65 |
| SSO1823 | -0.193 | 0.982 | 1.516 | 1.568 |
| SSO1824 | -0.081 | -0.08 | 0.421 | 0.308 |
| SSO1827 | 0.185 | 0.109 | 0.096 | 0.1 |
| SSO1831 | -0.208 | -0.234 | 0.433 | 0.34 |
| SSO1834 | -0.192 | 0.371 | 0.639 | 1.06 |
| SSO1835 | 0.032 | -0.066 | 0.547 | 0.642 |
| SSO1838 | -1.285 | -1.085 | 0.278 | 0.136 |
| SSO1839 | -0.034 | -0.352 | 0 | -0.137 |
| SSO1840 | -0.052 | -0.244 | 0.228 | 0.143 |
| SSO1842 | -0.539 | -0.504 | -0.443 | -0.36 |
| SSO1857 | 0.316 | 0.829 | 1.463 | 1.623 |
| SSO1858 | 0.057 | -0.247 | 0.036 | -0.142 |
| SSO1859 | -0.061 | 0.066 | -0.164 | -0.068 |
| SSO1860 | -0.035 | -0.356 | -0.209 | -0.152 |
| SSO1861 | -0.253 | -0.262 | -0.064 | -0.059 |
| SSO1863 | 0.049 | 0.219 | 0.346 | 0.394 |
| SSO1864 | -0.461 | -0.483 | -0.212 | -0.405 |
| SSO1865 | -0.249 | -0.129 | -0.466 | -0.112 |
| SSO1867 | -0.14 | -0.5 | -0.497 | -0.626 |
| SSO1868 | 0.018 | -0.072 | -0.219 | -0.157 |
| SSO1870 | 0.722 | -0.038 | -0.486 | -0.12 |
| SSO1872 | 0.782 | 0.535 | 0.566 | 0.535 |
| SSO1873 | NA | 0.436 | 0.177 | NA |
| SSO1874 | 0.251 | 0.237 | NA | 0.363 |
| SSO1875 | 0.326 | 0.144 | 0.243 | -0.434 |
| SSO1876 | 0.146 | NA | NA | NA |
| SSO1877 | 0.511 | 0.685 | 0.481 | 0.451 |
| SSO1878 | 0.349 | 0.338 | 0.414 | 0.224 |
| SSO1880 | 0.267 | 0.186 | 0.64 | 0.501 |
| SSO1881 | 0.253 | 0.199 | NA | NA |
| SSO1882 | 0.73 | 0 | 0.258 | 0.185 |
| SSO1886 | NA | 0.069 | NA | NA |
| SSO1891 | 0.546 | 0.812 | 0.133 | 0.132 |
| SSO1892 | 0.118 | 0.286 | 0.458 | 0.714 |
| SSO1898 | 0.102 | 0.015 | -0.103 | -0.092 |
| SSO1899 | -0.154 | 0.224 | 0.268 | 0.556 |
| SSO1901 | -0.599 | 0.028 | -0.032 | -0.194 |
| SSO1903 | 0.162 | 0.111 | -0.09 | -0.14 |
| SSO1905 | -0.611 | -0.306 | -0.546 | -0.23 |
| SSO1909 | -0.289 | 0.317 | 0.401 | 0.77 |
| SSO1910 | 0.055 | 0.107 | 0.067 | -0.101 |
| SSO1912 | 0.203 | 0.056 | NA | -0.232 |
| SSO1913 | -0.327 | -0.07 | -0.033 | -0.256 |
| SSO1914 | -0.089 | -0.022 | -0.039 | -0.098 |
| SSO1917 | 0.161 | 0.416 | 0.394 | -0.108 |
| SSO1918 | 0.052 | 0.151 | 0.449 | 0.195 |
| SSO1921 | 0.135 | NA | NA | NA |
| SSO1922 | -0.448 | 0.126 | -0.028 | 0.032 |
| SSO1931 | -0.433 | -0.233 | -0.121 | 0.014 |
| SSO1932 | -0.467 | -0.319 | 0.297 | -0.084 |
| SSO1939 | -0.532 | -0.601 | -0.252 | -0.352 |
| SSO1941 | -0.341 | -0.462 | -0.118 | -0.285 |
| SSO1945 | -0.211 | -0.087 | -0.247 | -0.158 |
| SSO1947 | 0.056 | -0.208 | -0.089 | -0.287 |
| SSO1949 | 0.175 | 0.32 | -0.518 | NA |
| SSO1952 | 0.349 | NA | NA | NA |
| SSO1958 | 0.167 | -0.021 | -0.029 | -0.047 |
| SSO1959 | -0.507 | -0.142 | 0.113 | -0.215 |
| SSO1960 | -0.22 | -0.355 | -0.492 | -0.474 |
| SSO1962 | 0.118 | NA | NA | 0.237 |
| SSO1968 | 0.271 | 0.001 | 0.228 | 0.25 |
| SSO1969 | NA | NA | NA | 0.066 |
| SSO1974 | -0.155 | 0.061 | -0.251 | -0.143 |
| SSO1975 | -0.332 | -0.214 | -0.083 | -0.297 |
| SSO1976 | -0.31 | -0.082 | -0.095 | 0.029 |
| SSO1980 | 0.242 | -0.203 | -0.067 | -0.059 |
| SSO1981 | NA | NA | 0.238 | NA |
| SSO1983 | 0.233 | 0.251 | 0.427 | 0.44 |
| SSO1984 | 0.159 | 1.369 | NA | 0.308 |
| SSO1986 | -0.605 | -0.299 | -0.1 | -0.226 |
| SSO1987 | -0.381 | -0.543 | -0.244 | -0.503 |
| SSO1988 | -0.021 | -0.403 | -0.22 | -0.291 |
| SSO1990 | -0.07 | -0.371 | -0.105 | -0.242 |
| SSO1991 | -0.693 | -0.838 | -0.544 | -0.639 |
| SSO1992 | -0.593 | -0.563 | -0.365 | -0.462 |
| SSO1993 | NA | NA | NA | 0.192 |
| SSO1996 | 0.035 | -0.43 | 0.105 | -0.003 |
| SSO1997 | -0.303 | -0.448 | 0.473 | -0.152 |
| SSO2005 | NA | NA | NA | -0.16 |
| SSO2010 | -0.146 | -0.368 | -0.023 | -0.241 |
| SSO2012 | 0.168 | 0.248 | 0.29 | 0.513 |
| SSO2014 | 0.121 | 0.089 | 0.28 | 0.509 |
| SSO2020 | 0.053 | 0.256 | 0.592 | 0.614 |
| SSO2022 | -0.246 | -0.227 | -0.03 | -0.053 |
| SSO2025 | 0.038 | 0.223 | 0.311 | 0.052 |
| SSO2028 | -0.355 | -0.05 | -0.144 | 0.072 |
| SSO2029 | 0.074 | -0.305 | -0.374 | -0.131 |
| SSO2030 | 0.135 | -0.209 | -0.258 | -0.37 |
| SSO2032 | 0.124 | 0.084 | NA | -0.192 |
| SSO2035 | 0.973 | 0.555 | 0.07 | 0.322 |
| SSO2039 | 0.38 | 0.023 | 0.238 | 0.159 |
| SSO2043 | 0.338 | -0.254 | -0.266 | -0.491 |
| SSO2044 | -0.105 | -0.278 | -0.059 | -0.165 |
| SSO2045 | 0.022 | -0.373 | -0.108 | -0.238 |
| SSO2049 | 0.029 | 0.215 | 0.057 | NA |
| SSO2050 | 0.225 | 0.03 | 0.203 | 0.065 |
| SSO2053 | 0.254 | 0.065 | 0.047 | 0.082 |
| SSO2054 | 0.687 | 0 | 0.104 | 0.009 |
| SSO2057 | 0.325 | 0.196 | 0.304 | 0.254 |
| SSO2058 | 0.29 | NA | NA | NA |
| SSO2059 | 0.016 | -0.395 | -0.257 | -0.245 |
| SSO2060 | -0.121 | -0.416 | -0.054 | -0.372 |
| SSO2061 | -0.375 | -0.215 | 0.346 | -0.04 |
| SSO2062 | -0.705 | -0.738 | 0.23 | -0.262 |
| SSO2063 | -0.51 | -0.793 | -0.008 | -0.317 |
| SSO2064 | -0.264 | -0.484 | 0.046 | -0.303 |
| SSO2066 | -0.035 | -0.176 | -0.048 | -0.155 |
| SSO2067 | -0.042 | -0.402 | -0.005 | -0.109 |
| SSO2069 | NA | NA | 0.355 | NA |
| SSO2070 | 0.085 | -0.622 | -0.397 | -0.371 |
| SSO2071 | -0.629 | -0.195 | -0.134 | 0.153 |
| SSO2072 | 0.321 | 0.333 | -0.204 | 0.066 |
| SSO2073 | -0.026 | 0.148 | 0.875 | -0.381 |
| SSO2076 | 0.048 | 0.112 | 0.234 | NA |
| SSO2078 | 0.507 | 1.598 | 2.187 | 2.249 |
| SSO2079 | -0.069 | 0.758 | 2.222 | 2.485 |
| SSO2080 | -0.055 | 0.106 | 0.751 | 0.754 |
| SSO2081 | 0.036 | -0.351 | -0.432 | -0.25 |
| SSO2083 | 0.104 | 0.156 | -0.827 | -0.335 |
| SSO2085 | NA | NA | -0.072 | NA |
| SSO2087 | -0.214 | 0.854 | 0.606 | 0.965 |
| SSO2088 | -0.044 | -0.714 | 0.285 | -0.038 |
| SSO2089 | 0.201 | 0.091 | 0.602 | 0.793 |
| SSO2091 | 0.021 | -0.334 | 0.075 | -0.718 |
| SSO2092 | 0.518 | -0.175 | -0.554 | -0.531 |
| SSO2093 | 0.283 | -0.386 | -0.35 | -0.383 |
| SSO2094 | NA | -0.326 | 0.248 | -0.14 |
| SSO2095 | -0.773 | -1.107 | -0.604 | -1.06 |
| SSO2096 | NA | -0.031 | NA | NA |
| SSO2097 | -0.209 | 0.048 | -0.173 | -0.02 |
| SSO2098 | -0.582 | -0.482 | 0.025 | -0.364 |
| SSO2101 | -0.541 | NA | 0.138 | -0.098 |
| SSO2105 | 0.037 | 0.155 | 0.91 | 1.027 |
| SSO2106 | -0.087 | -0.108 | 0.435 | 0.308 |
| SSO2107 | 0.247 | 0.075 | 0.07 | 0.042 |
| SSO2109 | 0.527 | NA | -0.11 | NA |
| SSO2110 | -0.164 | -0.265 | -0.374 | -0.429 |
| SSO2111 | -0.156 | 0.111 | 0.404 | 0.672 |
| SSO2112 | -0.169 | -0.112 | 0.174 | 0.053 |
| SSO2113 | 0.389 | 0.084 | 0.485 | 0.619 |
| SSO2114 | NA | -0.234 | NA | -0.234 |
| SSO2115 | 0.151 | -0.184 | 0.06 | 0.103 |
| SSO2116 | -0.155 | -0.24 | 0.052 | -0.026 |
| SSO2117 | 0.026 | 0.044 | -0.059 | -0.08 |
| SSO2118 | -0.054 | -0.16 | -0.073 | -0.161 |
| SSO2119 | -0.049 | -0.079 | 0.125 | -0.008 |
| SSO2121 | 0.102 | 0.595 | 0.653 | 0.888 |
| SSO2122 | -0.046 | -0.089 | 0.257 | 0.094 |
| SSO2125 | 0.622 | 0.131 | 0.082 | 0.117 |
| SSO2126 | -0.657 | -0.129 | 0.159 | 0.293 |
| SSO2127 | 0.196 | 0.04 | 0.304 | 0.546 |
| SSO2128 | -0.015 | -0.311 | 0.209 | 0.225 |
| SSO2129 | 0.162 | -0.013 | 0.597 | 0.543 |
| SSO2130 | 0.012 | -0.004 | 0.054 | 0.204 |
| SSO2131 | -0.343 | -0.182 | -0.221 | -0.121 |
| SSO2133 | -0.328 | 0.593 | 0.504 | 0.56 |
| SSO2134 | -0.031 | -0.066 | 0.08 | -0.04 |
| SSO2135 | 0.307 | -0.269 | 0.209 | -0.336 |
| SSO2137 | 0.33 | -0.737 | 0.222 | -0.641 |
| SSO2138 | 0.358 | -0.222 | 0.157 | -0.271 |
| SSO2139 | -0.146 | -0.163 | 0.321 | 0.202 |
| SSO2140 | 0.004 | 0.577 | 0.251 | 0.659 |
| SSO2141 | -0.075 | -0.586 | 0.5 | 0.113 |
| SSO2142 | 0.37 | 0.176 | 0.09 | 0.088 |
| SSO2146 | -0.187 | 0.21 | -0.16 | 0.244 |
| SSO2148 | 0.918 | 0.554 | 0.551 | 0.624 |
| SSO2150 | 0.059 | -0.49 | -0.074 | -0.678 |
| SSO2151 | 0.305 | 0.223 | 0.273 | 0.192 |
| SSO2152 | 0.407 | 0.318 | 0.125 | 0.214 |
| SSO2154 | 0.011 | -0.14 | -0.466 | -0.388 |
| SSO2155 | 0.154 | NA | NA | NA |
| SSO2156 | 0.176 | -0.516 | -0.35 | -0.431 |
| SSO2157 | 0.319 | -0.271 | -0.077 | -0.214 |
| SSO2158 | 0.535 | -0.215 | -0.274 | -0.269 |
| SSO2159 | 0.027 | -0.555 | -0.734 | -0.827 |
| SSO2160 | 0.223 | NA | 0.122 | 0.474 |
| SSO2161 | 0.689 | 0.533 | 0.647 | 0.781 |
| SSO2162 | -0.128 | -0.164 | 0.101 | -0.45 |
| SSO2163 | 0.195 | -0.041 | -0.023 | -0.203 |
| SSO2164 | NA | NA | NA | 0.098 |
| SSO2165 | 0.342 | 0.227 | 0.402 | 0.347 |
| SSO2166 | 0.462 | NA | 0.189 | NA |
| SSO2167 | 0.191 | -0.231 | -0.22 | -0.326 |
| SSO2169 | 0.387 | 0.129 | 0.382 | 0.606 |
| SSO2170 | 0.09 | 0.015 | -0.359 | -0.161 |
| SSO2171 | -0.097 | -0.067 | -0.135 | -0.166 |
| SSO2172 | 0.145 | -0.326 | 0.082 | -0.117 |
| SSO2174 | 0.053 | -0.218 | 0.075 | -0.193 |
| SSO2175 | 0.013 | 0.356 | 0.202 | 0.144 |
| SSO2176 | 0.117 | 0.316 | NA | 0.218 |
| SSO2178 | 0.404 | 0.586 | 0.858 | 1.242 |
| SSO2179 | 0.077 | -0.352 | -0.313 | -0.525 |
| SSO2181 | 0.001 | -0.332 | 0.611 | 0.391 |
| SSO2182 | -0.03 | -0.514 | -0.107 | -0.282 |
| SSO2183 | 0.273 | -0.237 | -0.246 | 0.005 |
| SSO2184 | -0.107 | -0.149 | -0.135 | -0.565 |
| SSO2186 | 0.204 | -0.075 | -0.228 | -0.192 |
| SSO2187 | 0.303 | 0.239 | 0.232 | 0.017 |
| SSO2188 | 0.122 | -0.284 | 0.17 | -0.007 |
| SSO2190 | 0.161 | -0.104 | -0.151 | -0.115 |
| SSO2191 | 0.148 | -0.117 | -0.195 | -0.238 |
| SSO2192 | -0.041 | -0.047 | 0.009 | 0.079 |
| SSO2193 | 0.347 | -0.026 | 0.129 | -0.064 |
| SSO2194 | 0.416 | 0.551 | 0.932 | 1.086 |
| SSO2195 | 0.256 | 0.308 | 0.203 | 0.025 |
| SSO2197 | -0.251 | -0.401 | 0.507 | 0.861 |
| SSO2199 | -0.13 | -0.472 | 1.169 | 1.434 |
| SSO2201 | 0.166 | -0.445 | -0.153 | -0.414 |
| SSO2202 | -0.331 | -0.911 | -0.903 | -0.772 |
| SSO2204 | 0.141 | -0.251 | 0.619 | 0.783 |
| SSO2205 | 0.442 | -0.201 | -0.016 | -0.143 |
| SSO2206 | 0.633 | -0.251 | 0.342 | NA |
| SSO2208 | -0.22 | -0.397 | -0.083 | 0.14 |
| SSO2209 | 0.276 | -0.527 | -0.833 | -0.672 |
| SSO2210 | 0.55 | 0.186 | 0.004 | 0.041 |
| SSO2211 | 0.013 | -0.211 | 0.235 | -0.146 |
| SSO2212 | 0.12 | 0.418 | 0.109 | 0.295 |
| SSO2213 | 0.014 | -0.432 | -0.466 | -0.708 |
| SSO2214 | 0.12 | NA | NA | NA |
| SSO2215 | -0.137 | -0.377 | -0.064 | -0.172 |
| SSO2216 | 0.166 | -0.016 | 0.338 | 0.118 |
| SSO2218 | 0.194 | -0.032 | -0.062 | -0.125 |
| SSO2219 | -0.025 | -0.282 | -0.442 | -0.351 |
| SSO2220 | 0.286 | -0.07 | 0.172 | 0.069 |
| SSO2221 | 0.073 | 0.076 | 0.032 | -0.099 |
| SSO2222 | -0.156 | -0.626 | -0.316 | -0.692 |
| SSO2223 | 0.257 | -0.049 | 0.063 | 0.035 |
| SSO2224 | 0.073 | -0.097 | 0.077 | -0.14 |
| SSO2225 | -0.05 | -0.198 | -0.014 | -0.059 |
| SSO2226 | -0.154 | -0.162 | 0.03 | -0.379 |
| SSO2227 | 0.11 | 0.193 | 0.218 | 0.356 |
| SSO2229 | -0.139 | -0.455 | -0.254 | -0.516 |
| SSO2230 | 0.031 | 0.084 | 0.104 | 0.188 |
| SSO2231 | -0.173 | -0.592 | -0.039 | -0.571 |
| SSO2232 | -0.337 | -0.865 | -0.501 | -0.995 |
| SSO2233 | 0.045 | -0.521 | -0.576 | -0.658 |
| SSO2234 | 0.013 | -0.595 | -0.185 | -0.427 |
| SSO2236 | -0.198 | -0.143 | -0.041 | -0.064 |
| SSO2237 | -0.014 | -0.099 | -0.094 | -0.013 |
| SSO2238 | -0.345 | -0.543 | -0.39 | -0.052 |
| SSO2241 | -2.024 | -2.036 | -1.221 | -1.478 |
| SSO2243 | -0.591 | -0.233 | -0.173 | -0.156 |
| SSO2244 | -0.539 | -0.215 | -0.117 | -0.174 |
| SSO2245 | 0.064 | -0.11 | -0.013 | -0.051 |
| SSO2246 | -0.08 | -0.244 | 0.05 | -0.037 |
| SSO2247 | -0.47 | -0.188 | -0.428 | -0.275 |
| SSO2248 | -0.626 | -0.792 | -0.164 | -0.338 |
| SSO2249 | -0.39 | -0.343 | 0.165 | -0.037 |
| SSO2250 | -0.259 | -0.075 | 0.756 | 0.246 |
| SSO2251 | -0.038 | 0.082 | 1.074 | 0.485 |
| SSO2253 | -0.146 | -0.19 | -0.455 | -0.424 |
| SSO2254 | -0.096 | -0.202 | -0.043 | -0.222 |
| SSO2255 | -0.201 | -0.42 | -0.503 | -0.502 |
| SSO2256 | 0.197 | -0.162 | 0.22 | 0.187 |
| SSO2257 | -0.047 | -0.356 | 0.218 | -0.025 |
| SSO2261 | 2.934 | 0.65 | 0.407 | 0.204 |
| SSO2262 | 0.563 | 0.002 | -0.09 | -0.286 |
| SSO2263 | 0.35 | -0.327 | -0.106 | -0.266 |
| SSO2265 | -0.247 | -0.603 | -0.311 | -0.607 |
| SSO2266 | -0.137 | -0.499 | -0.163 | -0.349 |
| SSO2268 | 0.245 | 0.062 | 0.044 | -0.075 |
| SSO2269 | 0.341 | 0.103 | -0.141 | -0.115 |
| SSO2273 | -0.249 | 0.006 | -0.185 | 0.064 |
| SSO2275 | 0.307 | -0.225 | 0.038 | -0.265 |
| SSO2276 | -0.101 | -0.245 | -0.379 | -0.312 |
| SSO2277 | -0.391 | -1.367 | -1.22 | -1.169 |
| SSO2278 | -0.239 | 0.012 | -0.261 | -0.058 |
| SSO2279 | -0.103 | -0.608 | -0.243 | -0.492 |
| SSO2281 | -0.412 | -1.121 | -0.313 | -1.133 |
| SSO2282 | 0.007 | -0.55 | -0.113 | -0.512 |
| SSO2284 | 0.622 | 0.344 | 0.949 | 0.877 |
| SSO2285 | 0.597 | 0.364 | 0.478 | 0.549 |
| SSO2286 | 0.162 | 0.33 | 0.19 | 0.317 |
| SSO2287 | 0.32 | 0.045 | 0.105 | 0.064 |
| SSO2288 | 0.268 | -0.316 | -0.394 | -0.173 |
| SSO2289 | -0.106 | -0.598 | -0.293 | -0.621 |
| SSO2290 | 0.19 | NA | NA | 0.175 |
| SSO2291 | -0.042 | -0.42 | -0.284 | -0.265 |
| SSO2292 | 0.114 | -0.344 | -0.429 | -0.401 |
| SSO2294 | 0.455 | -0.039 | 0.188 | 0.161 |
| SSO2295 | 0.168 | 0.191 | -0.076 | 0.024 |
| SSO2299 | 0.199 | -0.114 | 0.342 | 0.345 |
| SSO2301 | 0.031 | -0.136 | 0.102 | -0.13 |
| SSO2303 | 0.18 | -0.13 | 0.173 | -0.072 |
| SSO2305 | 0.241 | 0.011 | 0.339 | 0.092 |
| SSO2306 | 0.176 | -0.146 | 0.268 | -0.027 |
| SSO2308 | -0.002 | -0.205 | 0.297 | 0.16 |
| SSO2309 | 0.049 | -0.367 | 0.075 | -0.21 |
| SSO2310 | 0.188 | 0.028 | -0.145 | -0.253 |
| SSO2316 | 0.653 | NA | NA | 0.489 |
| SSO2318 | NA | NA | -0.766 | 0.312 |
| SSO2319 | 0.163 | -0.094 | 0.322 | 0.221 |
| SSO2323 | 0.177 | -0.08 | 0.178 | -0.071 |
| SSO2324 | NA | -0.328 | NA | -0.066 |
| SSO2327 | -0.65 | 0.43 | 0.558 | 0.351 |
| SSO2328 | 0.035 | 0.207 | 0.191 | 0.189 |
| SSO2332 | -0.066 | 0.442 | 0.798 | 0.557 |
| SSO2334 | -0.081 | -0.146 | 0.067 | -0.097 |
| SSO2336 | 0.271 | -0.008 | -0.211 | -0.131 |
| SSO2337 | -0.083 | -0.256 | -0.258 | -0.108 |
| SSO2338 | 0.59 | 1.17 | 2.703 | 3.053 |
| SSO2339 | 0.74 | 0.137 | 0.135 | 0.024 |
| SSO2342 | 0.016 | 0.406 | 0.078 | 0.207 |
| SSO2343 | -0.032 | -0.107 | -0.513 | -0.417 |
| SSO2344 | 0.424 | 0.015 | -0.065 | -0.306 |
| SSO2345 | 0.195 | 0.032 | -0.014 | -0.185 |
| SSO2346 | -0.074 | -0.251 | -0.234 | -0.355 |
| SSO2347 | -0.117 | -0.222 | 0.092 | -0.154 |
| SSO2348 | 0.785 | 0.102 | -0.1 | -0.126 |
| SSO2349 | 0.351 | 0.401 | -0.032 | 0.009 |
| SSO2350 | 0.356 | -0.026 | 0.351 | 0.092 |
| SSO2351 | 0.327 | 0.069 | 0.315 | 0.215 |
| SSO2352 | -0.064 | -0.101 | -0.2 | -0.221 |
| SSO2353 | 0.151 | 0.147 | 0.223 | 0.043 |
| SSO2354 | -0.069 | -0.223 | -0.247 | -0.179 |
| SSO2355 | -0.248 | -0.321 | -0.253 | -0.497 |
| SSO2356 | 0.252 | NA | 0.222 | NA |
| SSO2357 | 0.308 | -0.049 | 0.513 | 0.071 |
| SSO2358 | 0.2 | 0.099 | 0.466 | 0.386 |
| SSO2359 | 0.016 | -0.242 | 0.473 | 0.315 |
| SSO2360 | 0.317 | -0.125 | 0.31 | -0.027 |
| SSO2363 | 0.256 | -0.417 | -0.079 | -0.502 |
| SSO2364 | 1.189 | 0.315 | 0.1 | -0.08 |
| SSO2365 | 0.745 | 0 | 0.225 | -0.2 |
| SSO2367 | 0.34 | -0.054 | 0.066 | 0.014 |
| SSO2368 | 0.529 | 0.345 | 0.107 | 0.251 |
| SSO2370 | -0.646 | -0.558 | -0.623 | -0.144 |
| SSO2371 | 0.511 | 0.671 | 0.078 | -0.185 |
| SSO2372 | 0.076 | -0.015 | -0.138 | -0.039 |
| SSO2373 | 0.049 | -0.068 | 0.207 | 0.223 |
| SSO2374 | 1.491 | 0.068 | 0.001 | 0.105 |
| SSO2375 | 0.635 | -0.032 | -0.29 | -0.259 |
| SSO2377 | -0.069 | -0.39 | -0.115 | -0.176 |
| SSO2380 | 0.064 | -0.066 | 0 | -0.249 |
| SSO2381 | 0.295 | -0.291 | -0.42 | -0.434 |
| SSO2382 | 0.497 | -0.224 | -0.281 | -0.465 |
| SSO2383 | 0.777 | -0.319 | -0.242 | -0.261 |
| SSO2384 | 0.974 | -0.127 | 0.156 | 0.101 |
| SSO2385 | 0.505 | -0.141 | 0.285 | 0.189 |
| SSO2386 | -0.173 | -0.511 | 0.319 | 0.032 |
| SSO2387 | 0.021 | -0.284 | 0.114 | -0.167 |
| SSO2389 | -0.112 | -0.114 | NA | NA |
| SSO2390 | 0.017 | -0.263 | -0.312 | -0.367 |
| SSO2391 | 0.015 | -0.324 | -0.016 | -0.346 |
| SSO2393 | -0.211 | -0.525 | -0.073 | -0.051 |
| SSO2394 | -0.276 | -0.758 | -0.468 | -0.974 |
| SSO2395 | -0.026 | 0.792 | 3.33 | 3.684 |
| SSO2397 | -0.157 | -0.136 | -0.275 | -0.269 |
| SSO2398 | -0.276 | -0.594 | -0.43 | -0.695 |
| SSO2399 | 0.185 | -0.311 | -0.122 | -0.202 |
| SSO2400 | 0.724 | -0.069 | -0.017 | -0.288 |
| SSO2401 | -0.164 | -0.104 | -0.004 | -0.024 |
| SSO2402 | 0.677 | NA | NA | NA |
| SSO2404 | -1.101 | 0.45 | 0.893 | 1.047 |
| SSO2405 | 0.143 | 0.389 | 0.578 | 0.977 |
| SSO2406 | -0.176 | -0.069 | 0.102 | -0.06 |
| SSO2407 | -0.39 | -0.519 | -0.472 | -0.568 |
| SSO2408 | 0.313 | -0.044 | 0.576 | 0.533 |
| SSO2409 | 0.098 | 0.35 | 0.189 | 0.22 |
| SSO2410 | 0.16 | 0.262 | 0.201 | 0.116 |
| SSO2411 | 0.196 | 0.072 | 0.005 | -0.069 |
| SSO2412 | -0.044 | 0.1 | 0.084 | 0.068 |
| SSO2413 | 0.354 | 0.3 | 0.273 | 0.188 |
| SSO2416 | 1.147 | -0.055 | -0.033 | -0.076 |
| SSO2418 | 0.514 | -0.072 | 0.248 | 0.127 |
| SSO2420 | 0.2 | -0.423 | -0.101 | -0.668 |
| SSO2422 | 0.351 | 0.036 | 0.509 | 0.104 |
| SSO2424 | -0.17 | -0.018 | -0.257 | -0.1 |
| SSO2425 | 0.575 | 0.029 | 0.299 | 0.179 |
| SSO2426 | 0.035 | -0.13 | -0.085 | 0.01 |
| SSO2427 | -0.452 | 0.154 | -0.464 | 0.027 |
| SSO2428 | 0.174 | 0.114 | 0.04 | -0.125 |
| SSO2429 | 0.645 | 0.055 | 0.256 | 0.119 |
| SSO2431 | -0.114 | 0.044 | -0.116 | -0.129 |
| SSO2432 | -0.059 | -0.096 | 0.115 | 0.112 |
| SSO2433 | 0.023 | 0.135 | 0.027 | 0.028 |
| SSO2434 | -0.162 | -0.512 | 0.254 | -0.423 |
| SSO2435 | 0.299 | -0.097 | 0.035 | -0.021 |
| SSO2436 | 0.508 | 0.363 | 0.309 | NA |
| SSO2438 | 0.588 | 0.184 | -0.02 | 0.085 |
| SSO2439 | 0.193 | 0.009 | 0.03 | -0.028 |
| SSO2440 | 0.075 | -0.399 | -0.489 | -0.603 |
| SSO2441 | 0.266 | -0.003 | 0.115 | 0.003 |
| SSO2442 | -0.606 | -0.213 | 0.163 | 0.202 |
| SSO2445 | 0.326 | 0.112 | 0.227 | 0.505 |
| SSO2446 | 0.049 | 0.045 | -0.034 | 0.14 |
| SSO2448 | 0.354 | 0.009 | NA | NA |
| SSO2449 | 0.718 | -0.021 | -0.324 | -0.464 |
| SSO2450 | -0.039 | -0.712 | -0.13 | -0.505 |
| SSO2451 | 0.055 | 0.1 | 0.041 | 0.057 |
| SSO2452 | 0.281 | -0.178 | -0.011 | -0.264 |
| SSO2453 | -0.068 | 0.031 | -0.318 | -0.19 |
| SSO2454 | 0.501 | 0.159 | 0.04 | -0.142 |
| SSO2455 | 0.117 | 0.237 | -0.256 | 0.015 |
| SSO2457 | 0.44 | -0.165 | -0.481 | -0.67 |
| SSO2458 | 0.323 | 0.031 | 0.113 | 0.113 |
| SSO2459 | 0.343 | 0.336 | 0.315 | 0.09 |
| SSO2460 | 0.436 | 0.131 | 0.078 | 0.019 |
| SSO2461 | 0.509 | 0.181 | 0.28 | 0.281 |
| SSO2462 | 0.048 | -0.109 | 0.024 | -0.378 |
| SSO2463 | 0.059 | -0.336 | -0.212 | -0.479 |
| SSO2464 | 0.193 | -0.101 | -0.056 | -0.259 |
| SSO2466 | 0.424 | 0.036 | 0.229 | 0.078 |
| SSO2468 | 0.012 | -0.431 | 0.154 | 0.125 |
| SSO2469 | -0.12 | -0.353 | -0.495 | -0.653 |
| SSO2470 | 0.012 | -0.045 | 0.11 | 0.042 |
| SSO2471 | -0.07 | -0.591 | 0.243 | -0.448 |
| SSO2472 | 0.124 | -0.011 | NA | -0.389 |
| SSO2473 | NA | NA | 0.292 | NA |
| SSO2474 | -0.259 | -0.168 | -0.226 | -0.345 |
| SSO2475 | 0.176 | NA | 0.132 | 0.275 |
| SSO2476 | 0.46 | 0.307 | 1.577 | 2.161 |
| SSO2478 | 0.181 | 0.261 | 0.425 | 0.828 |
| SSO2479 | -0.138 | 0.322 | 0.567 | 0.589 |
| SSO2481 | 0.289 | -0.078 | -0.28 | -0.027 |
| SSO2482 | -0.013 | -0.768 | -0.292 | -0.687 |
| SSO2483 | -0.239 | -0.954 | -0.327 | -0.921 |
| SSO2484 | 0.2 | -0.238 | -0.097 | 0.095 |
| SSO2485 | -0.056 | -0.03 | -0.217 | -0.039 |
| SSO2486 | -0.334 | -0.505 | -0.172 | -0.513 |
| SSO2487 | 0.389 | 0.255 | 0.437 | 0.679 |
| SSO2488 | 0.34 | 0.308 | 0.239 | 0.303 |
| SSO2489 | 0.235 | 0.208 | 0.093 | 0.096 |
| SSO2490 | 0.27 | 0.236 | 0.403 | 0.46 |
| SSO2491 | 0.043 | 0.086 | 0.091 | -0.015 |
| SSO2492 | 0.078 | 0.251 | -0.033 | 0.203 |
| SSO2493 | 0.158 | 0.119 | 0.272 | 0.265 |
| SSO2494 | 0.304 | 0.21 | 0.517 | 0.634 |
| SSO2496 | 0.352 | 0.165 | 0.941 | 0.751 |
| SSO2497 | -0.06 | 0.039 | 0.811 | 0.613 |
| SSO2498 | 0.188 | 0.79 | 1.39 | 1.303 |
| SSO2500 | -0.109 | 0.139 | 0.327 | 0.121 |
| SSO2501 | 0.334 | 0.118 | 0.343 | 0.192 |
| SSO2503 | -0.07 | -0.027 | 0.712 | 0.278 |
| SSO2505 | 0.065 | 0.103 | 0.606 | 0.537 |
| SSO2506 | -0.034 | 0.044 | 0.224 | 0.032 |
| SSO2507 | -0.531 | 0.301 | -0.016 | 0.013 |
| SSO2508 | -0.078 | -0.254 | 0.044 | -0.351 |
| SSO2509 | 0.083 | 0.133 | -0.059 | -0.17 |
| SSO2510 | -0.104 | -0.365 | 0.144 | -0.172 |
| SSO2511 | -0.167 | -0.232 | 0.1 | -0.202 |
| SSO2514 | -0.221 | -1.061 | 0.255 | -0.816 |
| SSO2515 | -0.345 | 0.389 | -0.223 | -0.113 |
| SSO2516 | NA | NA | 0.191 | 0.24 |
| SSO2517 | -0.179 | -0.36 | 0.146 | -0.152 |
| SSO2518 | -0.253 | -0.279 | 0.05 | -0.058 |
| SSO2519 | -0.337 | -0.082 | 0.142 | 0.034 |
| SSO2520 | -0.661 | -0.328 | 0.116 | -0.02 |
| SSO2521 | -0.335 | -0.329 | -0.189 | -0.094 |
| SSO2522 | -0.205 | -0.176 | 0.122 | 0.042 |
| SSO2523 | -0.137 | -0.343 | 0.691 | 0.485 |
| SSO2524 | -0.096 | -0.504 | 0.04 | -0.338 |
| SSO2526 | 0.136 | -0.24 | 0.039 | -0.307 |
| SSO2527 | 0.242 | 0.018 | -0.198 | -0.302 |
| SSO2531 | 0.356 | 0.054 | 0.259 | 0.234 |
| SSO2532 | 0.085 | 0.485 | 0.043 | 0.263 |
| SSO2534 | -0.095 | 0.155 | 0.168 | 0.029 |
| SSO2535 | -0.125 | -0.169 | 0.093 | -0.095 |
| SSO2536 | 0.003 | 0.035 | 0.657 | 0.671 |
| SSO2537 | -0.404 | -0.101 | 0.179 | -0.085 |
| SSO2538 | -0.425 | -0.623 | -0.38 | -0.646 |
| SSO2539 | -0.146 | -0.376 | -1.182 | -0.872 |
| SSO2541 | -0.307 | -0.248 | -0.322 | -0.442 |
| SSO2544 | 0.129 | -0.1 | -0.06 | 0.029 |
| SSO2546 | 0.257 | 0.065 | 0.539 | 0.699 |
| SSO2549 | -0.39 | -0.175 | -0.043 | -0.146 |
| SSO2550 | -0.416 | -0.392 | -0.192 | -0.375 |
| SSO2551 | 0.059 | -0.438 | -0.356 | -0.534 |
| SSO2552 | -0.335 | -0.371 | -0.279 | -0.26 |
| SSO2553 | NA | 0.189 | NA | -0.397 |
| SSO2554 | 0.246 | -0.249 | 0.041 | -0.289 |
| SSO2555 | 0.04 | -0.064 | -0.065 | -0.058 |
| SSO2556 | 0.188 | -0.052 | -0.506 | -0.39 |
| SSO2558 | 0.287 | 0.297 | -0.09 | 0.042 |
| SSO2559 | -0.008 | -0.123 | -0.217 | -0.367 |
| SSO2560 | -0.306 | -0.025 | 0.079 | 0.248 |
| SSO2561 | 0.43 | 0.254 | 0.446 | NA |
| SSO2568 | -0.168 | -0.178 | 0.603 | 0.285 |
| SSO2569 | 0.057 | 0.344 | 0.382 | 1.093 |
| SSO2570 | 0.022 | 0.023 | -0.129 | 0.058 |
| SSO2571 | 0.468 | 0 | 0.062 | 0.096 |
| SSO2572 | -0.027 | -0.369 | -0.045 | -0.3 |
| SSO2573 | -0.198 | -0.06 | -0.575 | -0.105 |
| SSO2574 | -0.247 | 0.13 | -0.277 | -0.19 |
| SSO2575 | 0.391 | 0.121 | 0.004 | 0.027 |
| SSO2579 | 0.143 | -0.035 | 0.202 | 0.007 |
| SSO2581 | 0.049 | -0.272 | -0.059 | -0.31 |
| SSO2582 | -0.047 | -0.065 | -0.098 | -0.193 |
| SSO2583 | -0.127 | -0.142 | -0.334 | -0.485 |
| SSO2585 | 0.173 | -0.252 | -0.518 | -0.545 |
| SSO2586 | 0.159 | -0.334 | -0.043 | -0.208 |
| SSO2587 | 0.076 | 0.388 | 0.012 | 0.081 |
| SSO2588 | -0.065 | -0.533 | -0.166 | -0.534 |
| SSO2589 | -0.288 | -0.585 | -0.2 | -0.496 |
| SSO2590 | 0.142 | -0.074 | 0.109 | -0.004 |
| SSO2594 | -0.075 | -0.139 | -0.086 | -0.329 |
| SSO2595 | -0.07 | -0.306 | -0.564 | -0.475 |
| SSO2597 | 0.496 | -0.014 | 0.076 | -0.113 |
| SSO2598 | 0.063 | -0.077 | -0.248 | -0.404 |
| SSO2599 | 0.037 | -0.241 | -0.026 | -0.192 |
| SSO2600 | 0.827 | 0.521 | 0.432 | 0.352 |
| SSO2601 | 0.491 | -0.202 | 0.18 | NA |
| SSO2602 | -0.356 | -1.072 | -0.358 | -0.78 |
| SSO2603 | -0.393 | 0.095 | -0.095 | 0.173 |
| SSO2605 | 0.317 | -0.108 | 0.07 | -0.096 |
| SSO2606 | 0.2 | -0.215 | -0.034 | -0.167 |
| SSO2607 | -0.277 | -0.293 | -0.282 | -0.259 |
| SSO2608 | 0.431 | -0.322 | 0.051 | 0.13 |
| SSO2609 | -0.054 | -0.296 | 0.005 | 0.335 |
| SSO2610 | 0.054 | -0.334 | -0.201 | -0.377 |
| SSO2611 | 0.024 | -0.155 | -0.377 | -0.298 |
| SSO2612 | -0.045 | 0.222 | -0.303 | 0 |
| SSO2613 | -0.223 | 0.083 | -0.359 | 0.169 |
| SSO2615 | -0.548 | -0.286 | 0.043 | -0.102 |
| SSO2616 | 0.241 | NA | -0.011 | -0.158 |
| SSO2617 | 0.046 | -0.086 | 0.587 | 0.101 |
| SSO2618 | -0.296 | 0.084 | 0.108 | 0.091 |
| SSO2619 | 0.259 | -0.567 | -0.394 | -0.575 |
| SSO2620 | 0.014 | -0.262 | -0.153 | -0.254 |
| SSO2621 | 0.283 | -0.013 | 0.282 | -0.044 |
| SSO2622 | 0.254 | -0.237 | 0.087 | -0.026 |
| SSO2623 | -0.153 | -0.373 | -0.302 | -0.474 |
| SSO2624 | -0.21 | -0.7 | -0.197 | -0.457 |
| SSO2625 | -0.478 | -0.3 | -0.296 | -0.6 |
| SSO2626 | -0.345 | -0.501 | -0.273 | -0.397 |
| SSO2627 | -0.266 | -0.582 | -0.585 | -0.441 |
| SSO2628 | 0.216 | 0.387 | 0.305 | -0.046 |
| SSO2629 | -0.177 | 0.028 | 0.268 | 0.1 |
| SSO2631 | -0.33 | -0.756 | 0.056 | -0.458 |
| SSO2632 | 0.311 | 0.045 | 0.038 | -0.293 |
| SSO2633 | 0.319 | 0.279 | -0.011 | 0.038 |
| SSO2634 | -0.637 | -0.155 | -0.245 | -0.391 |
| SSO2635 | -0.163 | -0.294 | 0.051 | -0.102 |
| SSO2636 | -0.686 | -0.144 | -0.065 | -0.198 |
| SSO2637 | 0.115 | -0.22 | 0.005 | -0.421 |
| SSO2639 | -0.142 | -0.221 | 0.086 | 0.03 |
| SSO2640 | -0.273 | -0.039 | 0.108 | -0.096 |
| SSO2641 | -0.265 | -0.158 | 0.095 | -0.1 |
| SSO2642 | -0.074 | 0.352 | 0.189 | 0.77 |
| SSO2643 | 0.616 | 0.325 | 1.504 | 0.762 |
| SSO2644 | 0.182 | 0.154 | 1.259 | 0.855 |
| SSO2645 | -0.107 | -0.118 | 0.132 | 0.093 |
| SSO2646 | 0.386 | 0.163 | 0.081 | -0.006 |
| SSO2647 | 0.259 | -0.201 | 0.088 | -0.268 |
| SSO2648 | 0.301 | 0.039 | -0.045 | 0.154 |
| SSO2651 | -0.014 | -0.664 | 0.167 | -0.295 |
| SSO2652 | 0.284 | -0.037 | -0.236 | -0.192 |
| SSO2653 | 0.295 | -0.117 | -0.292 | -0.485 |
| SSO2656 | -0.05 | -0.283 | -0.026 | 0.005 |
| SSO2657 | 0.151 | -0.044 | 0.358 | 0.121 |
| SSO2658 | -0.041 | -0.149 | 0.202 | -0.031 |
| SSO2660 | 0.271 | -0.125 | 0.038 | 0.151 |
| SSO2661 | 0.074 | -0.086 | 0.41 | 0.26 |
| SSO2662 | 0.222 | -0.057 | 0.179 | 0.007 |
| SSO2663 | 0.046 | -0.085 | 0.015 | 0.138 |
| SSO2664 | -0.007 | -0.011 | -0.185 | -0.067 |
| SSO2665 | -0.319 | -0.421 | -0.248 | -0.272 |
| SSO2666 | 0.427 | 0.144 | 0.113 | -0.128 |
| SSO2667 | 0.251 | 0.174 | 0.206 | 0.099 |
| SSO2668 | -0.033 | -0.403 | -0.176 | -0.08 |
| SSO2669 | 0.108 | -0.402 | 0.351 | -0.057 |
| SSO2670 | 0.514 | 0.177 | 0.178 | 0.281 |
| SSO2671 | 0.293 | -0.159 | 0.32 | 0.089 |
| SSO2672 | 0.074 | -0.08 | -0.064 | -0.115 |
| SSO2673 | 0.301 | -0.082 | 0.185 | 0.171 |
| SSO2675 | 0.366 | -0.19 | 0.082 | -0.025 |
| SSO2676 | -0.051 | -0.173 | -0.397 | -0.359 |
| SSO2677 | -0.179 | 0.001 | -0.57 | -0.317 |
| SSO2678 | 0.011 | -0.331 | -0.315 | -0.206 |
| SSO2679 | 0.302 | 0.078 | 0.05 | -0.086 |
| SSO2680 | -0.046 | -0.246 | -0.179 | -0.057 |
| SSO2681 | -0.156 | 0.152 | 0.001 | 0.085 |
| SSO2683 | 0.15 | -0.035 | 0.273 | -0.046 |
| SSO2684 | 0.066 | -0.264 | 0.026 | -0.159 |
| SSO2686 | 0.155 | -0.008 | 0.291 | 0.264 |
| SSO2687 | NA | NA | NA | 0.548 |
| SSO2688 | 0.335 | 0.524 | 0.694 | 0.846 |
| SSO2689 | 0.214 | 0.1 | 0.61 | 0.539 |
| SSO2690 | 0.07 | 0.208 | -0.18 | 0.252 |
| SSO2691 | 0.176 | -0.08 | -0.197 | -0.01 |
| SSO2693 | -0.069 | -0.447 | 0.084 | -0.162 |
| SSO2694 | -0.044 | 0.075 | NA | NA |
| SSO2697 | -0.166 | 0.255 | -0.137 | -0.199 |
| SSO2699 | 0.078 | 0.074 | 0.08 | -0.046 |
| SSO2700 | 0.103 | 0.177 | 0.602 | 0.194 |
| SSO2702 | NA | NA | 0.215 | 0.314 |
| SSO2703 | 0.237 | 0.288 | 0.131 | 0.168 |
| SSO2704 | 0.394 | 0.868 | 1.001 | 0.916 |
| SSO2705 | -0.147 | 0.046 | -0.109 | -0.094 |
| SSO2707 | -0.138 | 0.233 | -0.032 | 0.051 |
| SSO2708 | -0.06 | 0.039 | -0.381 | -0.343 |
| SSO2710 | -0.008 | -0.024 | -0.156 | -0.313 |
| SSO2711 | 0.266 | 0.339 | -0.112 | -0.024 |
| SSO2712 | -0.24 | -0.712 | 0.008 | -0.512 |
| SSO2713 | -0.053 | -0.483 | 0.049 | -0.602 |
| SSO2714 | 0.164 | -0.048 | 0.388 | 0.592 |
| SSO2716 | 0.573 | 0.33 | 0.073 | 0.044 |
| SSO2717 | -0.136 | -0.272 | 0.536 | 0.529 |
| SSO2718 | 0.095 | 0.022 | -0.006 | -0.13 |
| SSO2719 | -0.013 | -0.333 | 0.022 | -0.26 |
| SSO2720 | 0.109 | 0.094 | 0.144 | 0.394 |
| SSO2725 | -0.31 | -0.295 | -0.222 | -0.211 |
| SSO2726 | 0.001 | -0.199 | -0.344 | -0.37 |
| SSO2727 | 0.07 | -0.248 | 0.057 | -0.228 |
| SSO2728 | -0.529 | -0.017 | 0.257 | 0.306 |
| SSO2729 | 0.026 | -0.174 | 0.029 | 0.169 |
| SSO2730 | 0.033 | -0.362 | -0.042 | -0.39 |
| SSO2732 | -0.149 | 0.194 | -0.171 | -0.089 |
| SSO2733 | 0.238 | 0.067 | 0.094 | 0.038 |
| SSO2734 | 0.171 | 0.036 | 0.148 | -0.113 |
| SSO2735 | -0.627 | -0.353 | -0.287 | -0.161 |
| SSO2736 | -0.092 | -0.262 | -0.084 | -0.197 |
| SSO2737 | -0.091 | -0.41 | -0.097 | -0.488 |
| SSO2738 | NA | NA | NA | -0.011 |
| SSO2740 | -0.107 | -0.141 | -0.092 | -0.049 |
| SSO2741 | 0.188 | 0.003 | -0.039 | -0.159 |
| SSO2742 | 0.31 | -0.118 | 0.225 | -0.074 |
| SSO2743 | 0.461 | 0.157 | 0.394 | 0.368 |
| SSO2744 | 0.08 | -0.113 | 0.214 | 0.012 |
| SSO2745 | 0.172 | 0.537 | 0.143 | 0.517 |
| SSO2746 | 0.499 | -0.082 | -0.111 | -0.218 |
| SSO2747 | -0.137 | -0.249 | -0.277 | -0.436 |
| SSO2748 | -0.295 | -0.383 | -0.314 | -0.434 |
| SSO2749 | -0.762 | 0.022 | 0.139 | 0.016 |
| SSO2750 | -0.629 | -1.592 | -2.03 | -1.597 |
| SSO2751 | -0.655 | -1.007 | -1.722 | -1.226 |
| SSO2752 | 0.29 | 0.481 | 0.41 | 0.299 |
| SSO2753 | 0.1 | -0.324 | 0.014 | -0.217 |
| SSO2754 | -0.226 | -0.265 | -0.14 | -0.419 |
| SSO2755 | 0.222 | -0.138 | 0.181 | 0.139 |
| SSO2756 | 0.053 | -0.094 | 0.772 | 0.479 |
| SSO2757 | 0.538 | 0.424 | 1.197 | 0.492 |
| SSO2758 | 0.653 | 1.29 | -0.044 | 0.398 |
| SSO2759 | 0.46 | 0.098 | 0.262 | 0.019 |
| SSO2761 | 0.28 | -0.146 | 0.204 | -0.092 |
| SSO2762 | -0.026 | -0.288 | -0.061 | -0.592 |
| SSO2763 | -0.199 | -0.142 | -0.136 | -0.643 |
| SSO2764 | 0.002 | -0.177 | -0.002 | 0.027 |
| SSO2765 | 1.089 | 0.311 | 1.154 | 1.612 |
| SSO2766 | -0.378 | -0.57 | -0.364 | -0.561 |
| SSO2768 | 0.071 | -0.047 | 0.074 | -0.159 |
| SSO2769 | 0.728 | 0.234 | -0.086 | -0.084 |
| SSO2770 | -0.112 | -0.148 | -0.469 | -0.595 |
| SSO2773 | -0.193 | -0.004 | -0.169 | -0.329 |
| SSO2774 | -0.006 | -0.181 | 0.005 | -0.205 |
| SSO2775 | 0.281 | -0.348 | -0.032 | -0.213 |
| SSO2776 | -0.028 | -0.25 | -0.304 | -0.373 |
| SSO2777 | 0.022 | -0.015 | -0.084 | -0.002 |
| SSO2778 | -0.02 | -0.126 | -0.791 | -0.717 |
| SSO2779 | 0.036 | 0.481 | -0.05 | -0.02 |
| SSO2780 | 0.159 | NA | NA | -0.507 |
| SSO2781 | -0.149 | -0.203 | -0.483 | -0.429 |
| SSO2783 | -0.259 | -0.216 | 0.135 | 0.038 |
| SSO2786 | -0.174 | 0.332 | -0.306 | -0.024 |
| SSO2787 | 0.006 | NA | NA | NA |
| SSO2788 | -0.034 | NA | -0.47 | 0.243 |
| SSO2789 | 0.17 | -0.375 | -0.436 | -0.537 |
| SSO2790 | 0.061 | -0.257 | -0.007 | -0.093 |
| SSO2791 | 0.254 | 0.002 | -0.034 | -0.103 |
| SSO2792 | 0.151 | -0.089 | 0.311 | 0.488 |
| SSO2793 | 0.199 | -0.008 | 0.804 | 0.993 |
| SSO2794 | 0.059 | -0.058 | 0.433 | 0.592 |
| SSO2795 | 0.092 | -0.214 | 0.025 | 0.009 |
| SSO2796 | -0.052 | -0.422 | -0.22 | -0.355 |
| SSO2797 | -0.135 | 0.372 | 0.2 | 0.305 |
| SSO2798 | 0.71 | 0.338 | 0.376 | 0.304 |
| SSO2799 | 0.535 | -0.052 | 0.331 | 0.256 |
| SSO2800 | -0.1 | -0.202 | 0.173 | -0.093 |
| SSO2801 | 0.23 | -0.029 | -0.082 | 0.337 |
| SSO2802 | 0.129 | 0.017 | 0.107 | -0.057 |
| SSO2803 | -0.034 | -0.295 | -0.136 | -0.226 |
| SSO2805 | 0.096 | -0.442 | -0.059 | -0.113 |
| SSO2807 | -0.108 | -0.184 | -0.373 | -0.062 |
| SSO2808 | 0.097 | -0.372 | 0.155 | -0.124 |
| SSO2810 | 0.042 | -0.107 | -0.006 | -0.141 |
| SSO2813 | 0.224 | -0.1 | 0.095 | 0.186 |
| SSO2814 | 0.564 | -0.215 | NA | NA |
| SSO2815 | 0.329 | -0.089 | 0.516 | 0.09 |
| SSO2816 | 0.028 | -0.125 | 0.487 | 0.048 |
| SSO2817 | 0.347 | 0.079 | -0.061 | -0.294 |
| SSO2819 | -0.033 | -0.397 | 0.069 | -0.11 |
| SSO2821 | 0.362 | -0.139 | 0.187 | -0.269 |
| SSO2822 | 0.324 | -0.236 | 0.211 | -0.292 |
| SSO2824 | 0.441 | 0.393 | 0.238 | -0.119 |
| SSO2825 | 0.207 | -0.033 | 0.098 | 0.047 |
| SSO2827 | -0.325 | -0.299 | 0.017 | 0.126 |
| SSO2828 | 0.406 | 0.294 | 0.128 | 0.312 |
| SSO2829 | 0.268 | -0.058 | 0.318 | -0.147 |
| SSO2830 | -0.004 | -0.144 | -0.027 | -0.043 |
| SSO2831 | 0.012 | -0.066 | 0.088 | 0.128 |
| SSO2832 | 0.046 | -0.223 | -0.116 | -0.385 |
| SSO2833 | 0.311 | -0.366 | NA | 0.702 |
| SSO2835 | 0.379 | 0.175 | 0.341 | 0.445 |
| SSO2836 | 0.398 | -0.05 | 0.268 | 0.152 |
| SSO2838 | 0.256 | -0.152 | 0.051 | -0.144 |
| SSO2839 | 0.165 | -0.241 | 0.162 | 0.223 |
| SSO2841 | 0.257 | -0.035 | 0.348 | 0.449 |
| SSO2846 | 0.084 | 0.288 | 0.128 | 0.126 |
| SSO2847 | -0.267 | -0.272 | -0.12 | -0.042 |
| SSO2848 | -0.218 | 0.016 | 0.007 | -0.138 |
| SSO2849 | -0.104 | -0.36 | 0.23 | -0.297 |
| SSO2850 | -0.196 | -0.229 | 0.065 | -0.255 |
| SSO2852 | -0.206 | -0.271 | 0.043 | -0.296 |
| SSO2853 | 0.409 | 0.373 | -0.275 | -0.241 |
| SSO2856 | 0.266 | 0.247 | 0.918 | 1.026 |
| SSO2857 | -0.491 | NA | -0.477 | 0.415 |
| SSO2858 | NA | 0.109 | 0.174 | 0.206 |
| SSO2860 | -0.165 | -0.047 | 0.52 | 0.295 |
| SSO2861 | 0.105 | NA | NA | NA |
| SSO2863 | -0.144 | -0.39 | 0.183 | -0.173 |
| SSO2864 | 0.033 | 0.453 | 0.359 | 0.804 |
| SSO2865 | 0.047 | 0.046 | 0.154 | 0.047 |
| SSO2866 | 0.166 | NA | NA | 0.044 |
| SSO2867 | -0.668 | 0.183 | 0.128 | 0.171 |
| SSO2868 | 0.343 | 0.135 | 0.046 | -0.028 |
| SSO2869 | 0.347 | 0.113 | 0.208 | -0.133 |
| SSO2870 | -0.07 | -0.233 | -0.092 | -0.173 |
| SSO2871 | -0.064 | -0.296 | -0.078 | -0.231 |
| SSO2872 | 0.087 | -0.472 | 0.064 | -0.402 |
| SSO2874 | -0.117 | -0.408 | 0.001 | -0.475 |
| SSO2875 | -0.137 | -0.387 | 0.033 | -0.333 |
| SSO2877 | -0.64 | 0.027 | 0.169 | 0.221 |
| SSO2878 | -0.357 | -0.037 | 0.214 | 0.024 |
| SSO2879 | -0.1 | 0.512 | 0.13 | 0.312 |
| SSO2880 | 0.599 | -0.085 | -0.239 | -0.376 |
| SSO2881 | 0.775 | 1.699 | 1.897 | 1.874 |
| SSO2884 | 0.04 | -0.181 | -0.04 | -0.226 |
| SSO2885 | -0.263 | 0.146 | 0.492 | 0.194 |
| SSO2886 | 0.325 | 0.342 | 0.094 | 0.216 |
| SSO2890 | -0.123 | -0.259 | -0.116 | -0.175 |
| SSO2891 | 0.002 | 0.185 | -0.299 | -0.084 |
| SSO2892 | 0.208 | 0.331 | 0.033 | 0.037 |
| SSO2896 | -0.101 | -0.285 | -0.018 | -0.049 |
| SSO2897 | -0.267 | 0.35 | 0.247 | 0.323 |
| SSO2899 | -0.107 | 0.019 | -0.207 | -0.153 |
| SSO2901 | 0.17 | 0.008 | -0.017 | 0.017 |
| SSO2902 | NA | NA | NA | 0.17 |
| SSO2905 | 1.363 | 0.734 | 0.226 | 0.338 |
| SSO2906 | 1.206 | 0.651 | 0.181 | -0.053 |
| SSO2907 | 0.742 | 0.042 | 0.909 | 1.29 |
| SSO2908 | -0.098 | -0.856 | -0.772 | -0.884 |
| SSO2909 | -1.004 | -1.713 | -1.442 | -1.713 |
| SSO2911 | -1.364 | -1.734 | -1.863 | -1.674 |
| SSO2912 | -1.489 | -1.846 | -1.588 | -1.986 |
| SSO2913 | -0.326 | -0.75 | -0.425 | -0.733 |
| SSO2914 | -0.171 | -0.735 | NA | NA |
| SSO2915 | 0.171 | -0.054 | 0.274 | 0.209 |
| SSO2921 | -0.05 | -0.498 | -0.329 | -0.662 |
| SSO2934 | 0.005 | -0.338 | -0.064 | -0.382 |
| SSO2936 | -0.142 | -0.38 | -0.071 | -0.154 |
| SSO2937 | 0.122 | -0.103 | NA | -0.126 |
| SSO2939 | -0.645 | -0.818 | -0.722 | -0.775 |
| SSO2940 | -0.441 | -0.405 | -0.455 | -0.456 |
| SSO2941 | 0.069 | 0.124 | 0.129 | -0.005 |
| SSO2942 | -0.367 | -0.292 | 0.07 | -0.228 |
| SSO2946 | -0.751 | -0.677 | -0.596 | -0.653 |
| SSO2952 | -0.039 | 0.174 | -0.386 | -0.618 |
| SSO2953 | -0.102 | -0.208 | -0.061 | -0.208 |
| SSO2954 | -0.097 | -0.233 | NA | -0.056 |
| SSO2955 | 0.051 | 0.025 | -0.018 | -0.01 |
| SSO2960 | 0.108 | -0.081 | -0.092 | -0.112 |
| SSO2962 | -0.346 | -0.483 | -0.249 | -0.533 |
| SSO2963 | -0.149 | -0.399 | -0.386 | -0.417 |
| SSO2964 | -0.707 | -0.316 | -0.515 | -0.377 |
| SSO2965 | 0.279 | 0.113 | 0.009 | 0.162 |
| SSO2966 | 0.013 | 0.433 | 0.552 | 0.672 |
| SSO2967 | 0.004 | 0.766 | 1.198 | 1.234 |
| SSO2968 | -0.185 | -0.118 | 0.3 | 0.026 |
| SSO2969 | -0.14 | -0.128 | 0.197 | -0.062 |
| SSO2970 | -0.289 | -0.209 | 0.449 | -0.087 |
| SSO2971 | -0.47 | 0.001 | 0.355 | 0.079 |
| SSO2972 | -0.853 | 0.663 | 0.556 | 0.723 |
| SSO2973 | -0.198 | 0.29 | 0.387 | 0.067 |
| SSO2975 | 0.388 | 0.322 | 0.729 | 0.816 |
| SSO2976 | NA | 0.636 | NA | 0.467 |
| SSO2977 | 0.778 | -0.027 | -0.042 | -0.051 |
| SSO2978 | -0.147 | -0.153 | -0.144 | -0.222 |
| SSO2979 | -0.18 | -0.092 | -0.473 | -0.319 |
| SSO2980 | 0.61 | -0.104 | -0.038 | -0.162 |
| SSO2981 | NA | 0.069 | NA | 0.002 |
| SSO2983 | 0.236 | -0.017 | -0.126 | -0.239 |
| SSO2986 | 0.114 | 0.449 | 0.09 | 0.433 |
| SSO2987 | 0.133 | 0.064 | -0.43 | -0.163 |
| SSO2988 | 0.311 | -0.182 | -0.089 | -0.186 |
| SSO2989 | 0.029 | -0.288 | -0.211 | -0.408 |
| SSO2990 | -0.034 | -0.026 | 0.028 | -0.133 |
| SSO2991 | 0.464 | 0.029 | 0.008 | -0.178 |
| SSO2994 | NA | NA | NA | 0.058 |
| SSO2995 | 0.716 | 0.059 | 0.171 | -0.024 |
| SSO2996 | 0.158 | -0.122 | 0.04 | -0.233 |
| SSO3000 | 0.289 | 0.04 | -0.107 | -0.432 |
| SSO3002 | 0.2 | 0.41 | -0.144 | 0.13 |
| SSO3003 | -0.559 | -0.128 | -0.155 | -0.191 |
| SSO3004 | -0.131 | -0.273 | -0.133 | -0.308 |
| SSO3006 | -0.143 | -0.452 | 0.087 | -0.581 |
| SSO3007 | -0.254 | -0.255 | 0.062 | -0.177 |
| SSO3008 | -0.115 | -0.04 | -0.11 | -0.151 |
| SSO3009 | -0.065 | -0.206 | 0.008 | -0.318 |
| SSO3010 | 0.299 | -0.144 | -0.204 | -0.237 |
| SSO3011 | -0.231 | -0.144 | -0.456 | -0.438 |
| SSO3012 | 0.308 | -0.487 | -0.132 | -0.611 |
| SSO3014 | 0.32 | 0.337 | -0.157 | -0.046 |
| SSO3015 | 0.036 | -0.329 | -0.081 | -0.111 |
| SSO3017 | 0.276 | 0.301 | 0.018 | 0.261 |
| SSO3019 | -0.383 | -0.133 | 0.143 | 0.09 |
| SSO3022 | 0.276 | 0.112 | 1.233 | 1.636 |
| SSO3029 | 0.174 | -0.023 | -0.034 | -0.073 |
| SSO3032 | 0.08 | -0.367 | 0.201 | -0.215 |
| SSO3034 | -0.283 | NA | 0.276 | NA |
| SSO3035 | 0.036 | -0.045 | 0.173 | 0.009 |
| SSO3036 | 0.408 | 0.286 | 0.349 | 0.356 |
| SSO3038 | NA | NA | 0.295 | 0.599 |
| SSO3039 | 0.044 | -0.432 | 0.214 | 0.057 |
| SSO3041 | -0.021 | -0.157 | NA | 0.155 |
| SSO3042 | 0.092 | -0.198 | -0.035 | 0.019 |
| SSO3043 | -0.085 | -0.481 | -0.041 | -0.062 |
| SSO3045 | -0.033 | -0.04 | 0.268 | NA |
| SSO3046 | 0.234 | -0.609 | 0.1 | 0.316 |
| SSO3048 | NA | NA | 0.375 | 0.287 |
| SSO3049 | 0.059 | -0.653 | NA | NA |
| SSO3050 | -0.058 | -0.374 | -0.184 | -0.336 |
| SSO3051 | -0.494 | -0.964 | -0.458 | -0.986 |
| SSO3052 | 0.058 | -0.429 | -0.351 | -0.137 |
| SSO3053 | -0.29 | -0.64 | -0.032 | -0.326 |
| SSO3054 | -0.32 | -0.43 | -0.118 | -0.001 |
| SSO3055 | -0.343 | -0.402 | -0.039 | -0.145 |
| SSO3058 | -0.524 | -0.388 | 0.189 | -0.033 |
| SSO3059 | NA | -0.865 | NA | NA |
| SSO3060 | 0.239 | NA | NA | -0.304 |
| SSO3061 | -0.296 | -0.443 | -0.407 | -0.455 |
| SSO3062 | NA | NA | 0.115 | 0.088 |
| SSO3064 | -0.203 | -0.342 | -0.1 | -0.3 |
| SSO3066 | -0.085 | -0.877 | -0.398 | -0.856 |
| SSO3067 | -0.053 | -0.461 | 0.102 | -0.063 |
| SSO3068 | -0.124 | -0.344 | 0.098 | -0.138 |
| SSO3069 | -0.674 | -0.966 | -0.418 | -0.725 |
| SSO3071 | -0.667 | -0.441 | -0.401 | -0.349 |
| SSO3072 | 0.375 | 0.1 | 0.318 | 0.269 |
| SSO3073 | 0.735 | 0.809 | 0.313 | 0.501 |
| SSO3074 | 0.309 | 0.015 | 0.148 | -0.093 |
| SSO3078 | 0.223 | -0.193 | -0.429 | -0.267 |
| SSO3080 | -0.04 | -0.109 | -0.351 | -0.199 |
| SSO3081 | 0.127 | -0.055 | 0.131 | 0.104 |
| SSO3082 | 0.291 | -0.163 | 0.187 | 0.035 |
| SSO3083 | -0.099 | -0.322 | -0.402 | -0.395 |
| SSO3084 | -0.54 | -0.715 | -0.059 | -0.535 |
| SSO3085 | -0.821 | -0.629 | -0.19 | -0.743 |
| SSO3087 | -0.959 | -0.44 | -0.639 | -0.67 |
| SSO3088 | -0.374 | -0.337 | -0.212 | -0.484 |
| SSO3089 | -1.381 | -0.706 | -0.464 | -0.619 |
| SSO3090 | -0.516 | -0.698 | -0.21 | -0.598 |
| SSO3091 | -0.213 | 0.14 | 1.659 | 2.032 |
| SSO3092 | 0.1 | 0.021 | 1.038 | 1.646 |
| SSO3093 | 0.057 | -0.282 | -0.138 | -0.308 |
| SSO3095 | 0.294 | -0.199 | -0.017 | -0.196 |
| SSO3097 | NA | -0.076 | NA | NA |
| SSO3098 | 0.493 | 0.04 | 0.034 | -0.064 |
| SSO3099 | -0.085 | -0.144 | 0.069 | -0.149 |
| SSO3100 | -0.216 | -0.395 | -0.66 | -0.547 |
| SSO3103 | -0.071 | -0.261 | -0.28 | -0.41 |
| SSO3104 | -0.107 | -0.47 | -0.443 | -0.56 |
| SSO3105 | 0.051 | -0.095 | 0.042 | -0.134 |
| SSO3107 | -0.086 | -0.17 | 0.18 | -0.112 |
| SSO3108 | NA | NA | -0.075 | NA |
| SSO3110 | -0.267 | 0.309 | 0.129 | 0.068 |
| SSO3111 | -0.45 | 0.332 | -0.047 | 0.133 |
| SSO3112 | 0.177 | -0.091 | -0.052 | -0.176 |
| SSO3113 | 0.18 | -0.101 | 0.245 | -0.206 |
| SSO3114 | 0.11 | 0.107 | 0.29 | -0.082 |
| SSO3115 | -0.211 | -0.184 | 0.091 | -0.185 |
| SSO3117 | 0.074 | -0.348 | 0.168 | -0.338 |
| SSO3118 | -0.678 | 0.004 | -0.135 | -0.199 |
| SSO3120 | -0.082 | -0.085 | -0.158 | -0.17 |
| SSO3121 | 0.279 | -0.124 | 0.035 | -0.239 |
| SSO3124 | -0.466 | -0.693 | -0.433 | -0.722 |
| SSO3125 | 0.171 | 0.043 | 0.074 | -0.104 |
| SSO3127 | 0.046 | -0.408 | -0.326 | -0.527 |
| SSO3128 | 0.163 | -0.154 | NA | NA |
| SSO3129 | 0.229 | -0.195 | -0.268 | -0.377 |
| SSO3130 | -0.059 | NA | NA | 0.374 |
| SSO3131 | 0.053 | 0.103 | 0.174 | 0.147 |
| SSO3132 | -0.061 | -0.069 | -0.108 | -0.021 |
| SSO3136 | -0.131 | -0.202 | -0.132 | -0.245 |
| SSO3138 | -0.102 | -0.291 | -0.096 | -0.104 |
| SSO3139 | -0.18 | -0.503 | -0.107 | -0.246 |
| SSO3141 | 0.112 | 0.052 | -0.138 | -0.037 |
| SSO3142 | 0.162 | -0.172 | 0.117 | -0.091 |
| SSO3143 | 0.088 | 0.062 | NA | NA |
| SSO3144 | -0.143 | -0.001 | -0.346 | -0.228 |
| SSO3145 | -0.108 | -0.29 | 0.263 | 0.212 |
| SSO3146 | 0.272 | 1.131 | 2.857 | 3.496 |
| SSO3147 | 0.056 | -0.228 | 0.225 | 0.303 |
| SSO3148 | -0.105 | -0.391 | -0.053 | -0.439 |
| SSO3149 | 0.323 | 0.124 | 0.43 | 0.486 |
| SSO3150 | 0.782 | -0.071 | 0.038 | 0.02 |
| SSO3155 | -0.035 | 0.055 | -0.079 | -0.231 |
| SSO3157 | 0.187 | 0.407 | -0.004 | 0.068 |
| SSO3158 | 0.044 | 0.017 | 0.203 | 0.014 |
| SSO3159 | 0.262 | -0.206 | 0.186 | -0.184 |
| SSO3161 | 0.166 | 0.181 | 0.014 | 0.249 |
| SSO3163 | 0.158 | -0.461 | 0.01 | -0.309 |
| SSO3164 | 0.354 | -0.068 | -0.18 | NA |
| SSO3165 | 0.38 | -0.165 | 0 | -0.187 |
| SSO3166 | 0.005 | -0.208 | -0.154 | -0.2 |
| SSO3167 | 0.448 | 0.212 | 0.177 | 0.257 |
| SSO3168 | 0.165 | 0 | 0.321 | 0.392 |
| SSO3169 | 0.128 | -0.016 | 0.076 | -0.066 |
| SSO3170 | 0.001 | -0.1 | -0.042 | 0.008 |
| SSO3174 | 0.442 | 0.27 | -0.016 | 0.197 |
| SSO3175 | 0.222 | -0.18 | 0.127 | -0.052 |
| SSO3176 | 0.189 | -0.006 | 0.011 | 0.005 |
| SSO3177 | -0.005 | 0.057 | -0.027 | 0.576 |
| SSO3178 | -0.303 | -0.024 | -0.235 | -0.135 |
| SSO3180 | -0.467 | -0.93 | 0.232 | 0.309 |
| SSO3181 | 0.245 | -0.066 | -0.007 | -0.091 |
| SSO3182 | -0.199 | -0.982 | -0.688 | -0.88 |
| SSO3183 | NA | NA | 0.191 | NA |
| SSO3184 | 0.083 | 0.049 | -0.094 | 0.049 |
| SSO3185 | 0.094 | 0.069 | 0.072 | 0.087 |
| SSO3186 | -0.203 | -0.072 | -0.209 | -0.078 |
| SSO3188 | -0.258 | -0.218 | -0.048 | -0.267 |
| SSO3189 | 0.276 | 0.371 | 0.784 | 1.111 |
| SSO3191 | 0.19 | -0.123 | 0.011 | 0.009 |
| SSO3192 | 0.344 | 0.563 | 0.808 | 0.914 |
| SSO3194 | 0.499 | -0.393 | 0.005 | -0.694 |
| SSO3195 | 0.327 | 0.049 | -0.05 | -0.059 |
| SSO3197 | 0.03 | -0.44 | 0.246 | -0.2 |
| SSO3198 | 0.343 | -0.388 | 0.157 | -0.327 |
| SSO3199 | 0.573 | 0.051 | -0.054 | NA |
| SSO3200 | 0.758 | -0.131 | -0.064 | -0.254 |
| SSO3201 | 1.182 | 0.137 | -0.326 | -0.255 |
| SSO3202 | 0.106 | 0.24 | 0.41 | 0.486 |
| SSO3203 | 0.259 | 0.175 | -0.144 | -0.087 |
| SSO3204 | 0.231 | -0.156 | 0.069 | 0.024 |
| SSO3205 | 0.262 | -0.2 | -0.235 | -0.153 |
| SSO3206 | -0.086 | -0.176 | -0.059 | -0.175 |
| SSO3207 | -0.151 | -0.726 | -0.813 | -0.662 |
| SSO3209 | -0.141 | -0.167 | 0.046 | -0.142 |
| SSO3210 | 0.032 | -0.064 | 0.414 | -0.096 |
| SSO3211 | -0.285 | -0.317 | 0.093 | -0.363 |
| SSO3212 | 0.202 | -0.062 | 0.367 | -0.019 |
| SSO3213 | 0.298 | 0.184 | 0.247 | 0.281 |
| SSO3214 | 0.321 | -0.253 | 0.002 | -0.216 |
| SSO3215 | 0.658 | 0.118 | -0.298 | -0.248 |
| SSO3216 | 0.645 | -0.005 | -0.339 | -0.532 |
| SSO3218 | 0.327 | 0.243 | 0.383 | 0.185 |
| SSO3219 | 0.547 | 0.176 | 0.138 | 0.133 |
| SSO3224 | 0.299 | 0.789 | -0.186 | 0.325 |
| SSO3225 | 0.13 | 0.144 | -0.105 | 0.222 |
| SSO3226 | 0.004 | -0.02 | 0.057 | 0.126 |
| SSO3227 | 0.349 | 0.142 | 0.277 | 0.161 |
| SSO3228 | 0.091 | -0.017 | -0.044 | -0.067 |
| SSO3230 | -0.073 | NA | -0.166 | NA |
| SSO3231 | 0.661 | 0.384 | 0.372 | 0.097 |
| SSO3232 | -0.339 | -0.062 | 0.111 | -0.055 |
| SSO3233 | 0.136 | 0.156 | 0.14 | 0.16 |
| SSO3234 | 0.04 | -0.066 | 0.053 | 0.072 |
| SSO3235 | 0.146 | 0.001 | 0.135 | 0.096 |
| SSO3237 | -0.251 | -1.177 | -1.189 | -1.035 |
| SSO3238 | 0.073 | 0.148 | 1.353 | 2.017 |
| SSO3239 | -0.006 | -0.627 | -0.078 | -0.295 |
| SSO3240 | 0.036 | -0.298 | -0.029 | -0.244 |
| SSO3241 | -0.026 | -0.363 | -0.215 | -0.166 |
| SSO3242 | -0.356 | -0.652 | -1.585 | -0.915 |
| SSO3243 | 0.25 | -0.234 | -0.024 | -0.244 |
| SSO3245 | 0.044 | 0.009 | -0.262 | -0.035 |
| SSO3246 | -0.357 | 0.261 | -0.955 | -1.095 |
| SSO3247 | 0.179 | 0.022 | 0.313 | 0.397 |
| SSO3248 | 0.214 | 0.012 | 0.133 | 0.073 |
| SSO3250 | 0.135 | -0.053 | -0.058 | -0.05 |
| SSO3251 | -0.073 | -0.097 | -0.507 | -0.201 |
| SSO3254 | 0.137 | -0.13 | 0.069 | -0.14 |
| SSO3257 | -0.83 | NA | NA | NA |
| SSO5027 | 0.292 | 0.065 | -0.09 | -0.032 |
| SSO5098 | 0.554 | 0.071 | 0.391 | 0.198 |
| SSO5140 | 0.145 | -0.471 | 0.073 | -0.312 |
| SSO5209 | 0.16 | -0.138 | 0.27 | 0.608 |
| SSO5317 | 0.141 | 0.574 | 0.352 | 0.237 |
| SSO5336 | -0.033 | 0.146 | 0.152 | 0.106 |
| SSO5343 | -0.077 | 0.272 | -0.235 | -0.208 |
| SSO5345 | 0.081 | 0.239 | -0.149 | -0.118 |
| SSO5410 | 0.302 | -0.064 | -0.148 | -0.297 |
| SSO5468 | 0.176 | 0.138 | 0.099 | 0.01 |
| SSO5478 | 0.257 | 0.162 | 0.002 | 0.111 |
| SSO5479 | 0.133 | 0.25 | -0.1 | -0.276 |
| SSO5522 | 0.25 | 0.284 | 0.08 | 0.407 |
| SSO5542 | 0.066 | -0.477 | -0.736 | -0.595 |
| SSO5544 | 0.238 | 0.044 | -0.375 | -0.26 |
| SSO5559 | -0.442 | -0.656 | -0.264 | -0.753 |
| SSO5561 | NA | NA | -0.01 | 0 |
| SSO5576 | 0.337 | 0.183 | NA | -0.109 |
| SSO5577 | 0.264 | NA | -0.11 | NA |
| SSO5663 | 0.345 | 0.005 | -0.419 | -0.062 |
| SSO5668 | -0.381 | -0.379 | -0.062 | -0.426 |
| SSO5671 | -0.319 | -0.922 | -0.253 | -0.541 |
| SSO5672 | 0.124 | -0.452 | -0.132 | -0.092 |
| SSO5761 | -0.275 | 0.032 | -0.178 | 0.059 |
| SSO5763 | -0.312 | -0.232 | -0.581 | -0.313 |
| SSO5798 | -0.167 | -0.094 | -0.354 | -0.293 |
| SSO5826 | -0.088 | -0.377 | -0.806 | -0.548 |
| SSO5844 | 0.079 | 0.104 | -0.218 | -0.137 |
| SSO5865 | 0.231 | 0.168 | 0.04 | -0.095 |
| SSO5866 | 0.212 | -0.006 | 0.111 | 0.239 |
| SSO5909 | 0.235 | 0.186 | -0.031 | -0.068 |
| SSO5983 | 0.226 | NA | NA | 0.116 |
| SSO6024 | NA | NA | -0.975 | NA |
| SSO6175 | -0.009 | -0.048 | -0.135 | -0.194 |
| SSO6179 | 0.275 | 0.149 | 0.156 | -0.018 |
| SSO6206 | 0.256 | -0.043 | -0.136 | -0.235 |
| SSO6223 | 0.13 | -0.241 | 0.211 | 0.133 |
| SSO6227 | NA | NA | NA | 0.486 |
| SSO6264 | 0.132 | -0.562 | -0.171 | -0.319 |
| SSO6374 | -0.478 | -0.339 | -0.149 | -0.221 |
| SSO6391 | 0.391 | NA | NA | NA |
| SSO6397 | -0.096 | -0.295 | -0.046 | -0.37 |
| SSO6401 | -0.011 | -0.486 | 0.201 | -0.477 |
| SSO6418 | -0.261 | 0.143 | -0.141 | -0.056 |
| SSO6453 | -0.108 | 0.065 | 0.04 | -0.117 |
| SSO6454 | 0.117 | -0.08 | -0.333 | -0.172 |
| SSO6503 | -0.056 | 0.415 | 0.69 | 0.697 |
| SSO6661 | 0.372 | 0.081 | 0.434 | 0.514 |
| SSO6663 | 0.111 | -0.24 | 0.175 | -0.045 |
| SSO6687 | 0.041 | -0.269 | -0.886 | -0.047 |
| SSO6716 | 0.164 | -0.092 | 0.115 | 0.029 |
| SSO6759 | -0.193 | 0.178 | -0.04 | 0.119 |
| SSO6768 | 0.112 | 0.04 | -0.047 | -0.021 |
| SSO6778 | -0.01 | 0.221 | -1 | -0.954 |
| SSO6817 | -0.464 | -0.169 | 0.201 | 0.301 |
| SSO6855 | 0.5 | -0.23 | -0.194 | -0.445 |
| SSO6877 | 0.059 | -0.228 | -0.016 | -0.324 |
| SSO6901 | -0.807 | -1.248 | -1.268 | -1.047 |
| SSO6904 | NA | 0.289 | -0.285 | 0.475 |
| SSO7111 | -0.015 | -0.217 | 0.169 | NA |
| SSO7114 | -0.109 | -0.231 | -0.153 | -0.449 |
| SSO7115 | 0.279 | -0.159 | 0.132 | -0.064 |
| SSO7348 | NA | 0.54 | 0.821 | 1.31 |
| SSO7412 | 0.007 | 0.261 | 0.332 | 0.584 |
| SSO7710 | 0.187 | 0.059 | 0.135 | 0.164 |
| SSO8090 | 0.097 | 0.048 | 0.274 | 0.216 |
| SSO8124 | 0.601 | 1.099 | 1.518 | 0.967 |
| SSO8462 | 0.024 | NA | NA | 0.241 |
| SSO8469 | 0.432 | 0.205 | 0.009 | 0.081 |
| SSO8472 | 0.195 | 0.307 | 0.288 | 0.19 |
| SSO8475 | 0.146 | 0.192 | 0.493 | 0.429 |
| SSO8478 | 0.03 | 0.005 | -0.145 | -0.266 |
| SSO8549 | 0.054 | -0.162 | -0.014 | -0.179 |
| SSO8620 | -0.206 | NA | NA | 0.05 |
| SSO8813 | NA | 0.546 | NA | NA |
| SSO8892 | -0.788 | 0.062 | 0.81 | 1.145 |
| SSO8906 | NA | 0.166 | NA | NA |
| SSO8910 | -0.812 | -0.156 | -0.181 | -0.127 |
| SSO8938 | NA | 0.11 | 0.351 | 0.585 |
| SSO8948 | -0.222 | 0.249 | 0.032 | NA |
| SSO8998 | -0.103 | -0.304 | 0.073 | -0.274 |
| SSO9092 | 0.386 | 0.124 | 0.042 | 0.192 |
| SSO9115 | 0.248 | 0.158 | -0.02 | 0.025 |
| SSO9136 | 0.241 | NA | NA | NA |
| SSO9180 | -1.019 | -0.82 | -1.518 | -0.747 |
| SSO9378 | -0.209 | 0.045 | 0.093 | 0.218 |
| SSO9500 | -0.273 | 0.131 | -0.22 | -0.037 |
| SSO9535 | -0.931 | -0.946 | -1.468 | -0.707 |
| SSO9953 | 0.305 | 0.004 | 0.144 | 0.202 |
