# Supplementary material for: Responses of hyperthermophilic crenarchaea to UV irradiation
Source: Genome Biol. 2007 Oct 11;8(10):R220. doi: 10.1186/gb-2007-8-10-r220 (PMC2246294; doi:10.1186/gb-2007-8-10-r220)
Supplement: Additional data file 4 — S. solfataricus genes repressed by UV irradiation. [file gb-2007-8-10-r220-S4.doc]

Table S4. Ratio of expression of all *S. solfataricus* genes repressed by UV irradiation, expressed as log_2_ (UV/ control), and ranked by degree of repression at 120 min.

| Gene | 30 min | 60 min | 90 min | 120 min |
| --- | --- | --- | --- | --- |
| SSO2912 | -1.489 | -1.846 | -1.588 | -1.986 |
| SSO0451 | -0.787 | -0.813 | -2.237 | -1.823 |
| SSO0911 | -0.356 | -1.638 | -2.079 | -1.773 |
| SSO2909 | -1.004 | -1.713 | -1.442 | -1.713 |
| SSO1256 | -0.725 | -1.495 | -1.483 | -1.7 |
| SSO2911 | -1.364 | -1.734 | -1.863 | -1.674 |
| SSO0034 | -0.521 | -1.414 | -2.356 | -1.612 |
| SSO2750 | -0.629 | -1.592 | -2.03 | -1.597 |
| SSO1817 | -1.376 | -1.848 | -1.49 | -1.574 |
| SSO2241 | -2.024 | -2.036 | -1.221 | -1.478 |
| SSO0356 | -0.375 | -1.111 | -0.769 | -1.375 |
| SSO0190 | -0.196 | -1.015 | -2.14 | -1.334 |
| SSO2751 | -0.655 | -1.007 | -1.722 | -1.226 |
| SSO1388 | -1.033 | -1.73 | -0.724 | -1.217 |
| SSO0345 | -0.129 | -0.71 | -0.609 | -1.207 |
| SSO1027 | -0.201 | 0.153 | -1.526 | -1.197 |
| SSO1287 | -0.488 | -0.906 | -1.001 | -1.185 |
| SSO2277 | -0.391 | -1.367 | -1.22 | -1.169 |
| SSO0965 | -0.344 | -1.183 | -0.441 | -1.158 |
| SSO0858 | -0.298 | -0.657 | -1.691 | -1.157 |
| SSO0048 | -0.198 | -0.591 | -1.324 | -1.14 |
| SSO2281 | -0.412 | -1.121 | -0.313 | -1.133 |
| SSO0946 | -0.708 | -1.608 | -1.264 | -1.13 |
| SSO1288 | -0.042 | -0.296 | -1.364 | -1.101 |
| SSO1399 | -0.891 | -0.769 | -0.602 | -1.096 |
| SSO0321 | 0.047 | -0.51 | -1.795 | -1.096 |
| SSO3246 | -0.357 | 0.261 | -0.955 | -1.095 |
| SSO0108 | -0.345 | -0.579 | -1.326 | -1.061 |
| SSO0239 | -0.389 | -0.955 | -0.515 | -1.061 |
| SSO2095 | -0.773 | -1.107 | -0.604 | -1.06 |
| SSO1089 | 0.058 | -0.696 | -1.176 | -1.053 |
| SSO6901 | -0.807 | -1.248 | -1.268 | -1.047 |
| SSO3237 | -0.251 | -1.177 | -1.189 | -1.035 |
| SSO0909 | 0.002 | -0.808 | -1.009 | -1.031 |
| SSO0069 | 0.167 | -1.004 | -0.358 | -1.028 |
| SSO10704 | 0.048 | 0.073 | -1.247 | -1.011 |
| SSO2232 | -0.337 | -0.865 | -0.501 | -0.995 |
| SSO3051 | -0.494 | -0.964 | -0.458 | -0.986 |
| SSO2394 | -0.276 | -0.758 | -0.468 | -0.974 |
| SSO0335 | -0.212 | -0.814 | -0.111 | -0.967 |
| SSO0619 | -0.293 | -0.89 | -1.008 | -0.956 |
| SSO6778 | -0.01 | 0.221 | -1 | -0.954 |
| SSO0149 | -0.029 | -0.679 | -0.905 | -0.95 |
| SSO0881 | -0.037 | -0.693 | -1.043 | -0.922 |
| SSO2483 | -0.239 | -0.954 | -0.327 | -0.921 |
| SSO3242 | -0.356 | -0.652 | -1.585 | -0.915 |
| SSO0257 | -0.014 | -0.248 | -1.421 | -0.905 |
| SSO1282 | -0.301 | -0.858 | -0.77 | -0.888 |
| SSO2908 | -0.098 | -0.856 | -0.772 | -0.884 |
| SSO1676 | -0.646 | -1.066 | -0.332 | -0.88 |
| SSO3182 | -0.199 | -0.982 | -0.688 | -0.88 |
| SSO0271 | -0.002 | -0.248 | -1.116 | -0.874 |
| SSO2539 | -0.146 | -0.376 | -1.182 | -0.872 |
| SSO0772 | 0.013 | -0.811 | -0.577 | -0.869 |
| SSO3066 | -0.085 | -0.877 | -0.398 | -0.856 |
| SSO0352 | -0.8 | -1.044 | -0.533 | -0.846 |
| SSO0241 | -0.54 | -1.07 | -0.459 | -0.839 |
| SSO1290 | -0.349 | -0.472 | -0.864 | -0.831 |
| SSO2159 | 0.027 | -0.555 | -0.734 | -0.827 |
| SSO0353 | -0.18 | -0.494 | -0.989 | -0.817 |
| SSO2514 | -0.221 | -1.061 | 0.255 | -0.816 |
| SSO0375 | -0.283 | -0.775 | -0.95 | -0.792 |
| SSO0018 | -0.358 | -0.797 | -0.432 | -0.785 |
| SSO0201 | 0.257 | -0.444 | -0.285 | -0.785 |
| SSO2602 | -0.356 | -1.072 | -0.358 | -0.78 |
| SSO2939 | -0.645 | -0.818 | -0.722 | -0.775 |
| SSO1653 | -0.782 | -0.886 | -0.148 | -0.773 |
| SSO2202 | -0.331 | -0.911 | -0.903 | -0.772 |
| SSO1307 | -0.111 | 0.005 | -0.085 | -0.768 |
| SSO5559 | -0.442 | -0.656 | -0.264 | -0.753 |
| SSO9180 | -1.019 | -0.82 | -1.518 | -0.747 |
| SSO0810 | -0.319 | -0.798 | -0.109 | -0.746 |
| SSO3085 | -0.821 | -0.629 | -0.19 | -0.743 |
| SSO0712 | 0.091 | -0.9 | -0.16 | -0.743 |
| SSO0942 | 0.445 | -0.269 | -0.675 | -0.743 |
| SSO0698 | -0.291 | -0.492 | -0.135 | -0.739 |
| SSO2913 | -0.326 | -0.75 | -0.425 | -0.733 |
| SSO0346 | 0.072 | -0.52 | -0.346 | -0.733 |
| SSO0110 | -0.559 | -0.691 | -0.83 | -0.729 |
| SSO0528 | -0.018 | -0.158 | -0.173 | -0.729 |
| SSO3069 | -0.674 | -0.966 | -0.418 | -0.725 |
| SSO3124 | -0.466 | -0.693 | -0.433 | -0.722 |
| SSO11914 | 0.193 | -0.281 | -0.835 | -0.721 |
| SSO2091 | 0.021 | -0.334 | 0.075 | -0.718 |
| SSO2778 | -0.02 | -0.126 | -0.791 | -0.717 |
| SSO1617 | NA | NA | NA | -0.714 |
| SSO1273 | -0.09 | -0.912 | 0.157 | -0.712 |
| SSO0334 | -0.036 | -0.628 | -0.028 | -0.711 |
| SSO2213 | 0.014 | -0.432 | -0.466 | -0.708 |
| SSO9535 | -0.931 | -0.946 | -1.468 | -0.707 |
| SSO0537 | -0.075 | -0.561 | -0.722 | -0.707 |
| SSO0232 | 0.102 | -0.629 | -0.145 | -0.705 |
| SSO0407 | -0.195 | -0.617 | -0.098 | -0.703 |
| SSO1108 | -0.441 | -0.345 | -0.585 | -0.697 |
| SSO2398 | -0.276 | -0.594 | -0.43 | -0.695 |
| SSO3194 | 0.499 | -0.393 | 0.005 | -0.694 |
| SSO0577 | -0.061 | -0.751 | -0.196 | -0.693 |
| SSO2222 | -0.156 | -0.626 | -0.316 | -0.692 |
| SSO1276 | -0.256 | -0.466 | -0.293 | -0.691 |
| SSO2482 | -0.013 | -0.768 | -0.292 | -0.687 |
| SSO1101 | -0.575 | -0.969 | -0.702 | -0.678 |
| SSO2150 | 0.059 | -0.49 | -0.074 | -0.678 |
| SSO0421 | -0.375 | -0.858 | 0.253 | -0.677 |
| SSO2209 | 0.276 | -0.527 | -0.833 | -0.672 |
| SSO3087 | -0.959 | -0.44 | -0.639 | -0.67 |
| SSO2457 | 0.44 | -0.165 | -0.481 | -0.67 |
| SSO2420 | 0.2 | -0.423 | -0.101 | -0.668 |
| SSO3207 | -0.151 | -0.726 | -0.813 | -0.662 |
| SSO2921 | -0.05 | -0.498 | -0.329 | -0.662 |
| SSO2233 | 0.045 | -0.521 | -0.576 | -0.658 |
| SSO1176 | 0.542 | -0.354 | -0.586 | -0.656 |
| SSO2946 | -0.751 | -0.677 | -0.596 | -0.653 |
| SSO2469 | -0.12 | -0.353 | -0.495 | -0.653 |
| SSO1818 | -0.76 | -0.977 | -0.271 | -0.65 |
| SSO1045 | -0.117 | -0.477 | -0.525 | -0.65 |
| SSO1260 | -0.465 | -0.515 | -0.394 | -0.648 |
| SSO2538 | -0.425 | -0.623 | -0.38 | -0.646 |
| SSO0532 | -0.072 | -0.157 | -0.419 | -0.644 |
| SSO2763 | -0.199 | -0.142 | -0.136 | -0.643 |
| SSO2137 | 0.33 | -0.737 | 0.222 | -0.641 |
| SSO1991 | -0.693 | -0.838 | -0.544 | -0.639 |
| SSO1019 | -1.318 | -0.492 | -0.614 | -0.638 |
| SSO0068 | 0.026 | -0.839 | 0.032 | -0.636 |
| SSO0093 | -0.197 | -0.668 | -0.22 | -0.633 |
| SSO0755 | -0.122 | -0.469 | -0.162 | -0.633 |
| SSO0505 | -0.527 | -0.576 | -0.409 | -0.627 |
| SSO1867 | -0.14 | -0.5 | -0.497 | -0.626 |
| SSO2289 | -0.106 | -0.598 | -0.293 | -0.621 |
| SSO0936 | -0.241 | -0.852 | -0.104 | -0.62 |
| SSO1283 | -0.057 | -0.715 | -0.419 | -0.62 |
| SSO3089 | -1.381 | -0.706 | -0.464 | -0.619 |
| SSO2952 | -0.039 | 0.174 | -0.386 | -0.618 |
| SSO10051 | -0.076 | -0.561 | -0.425 | -0.614 |
| SSO0284 | 0.069 | -0.391 | -0.02 | -0.613 |
| SSO3012 | 0.308 | -0.487 | -0.132 | -0.611 |
| SSO2265 | -0.247 | -0.603 | -0.311 | -0.607 |
| SSO0609 | 0.087 | -0.641 | -0.64 | -0.606 |
| SSO0697 | -0.113 | -0.472 | 0.103 | -0.606 |
| SSO0561 | -0.509 | -0.541 | -0.345 | -0.604 |
| SSO2440 | 0.075 | -0.399 | -0.489 | -0.603 |
| SSO0613 | -0.124 | -0.313 | -0.244 | -0.602 |
| SSO2713 | -0.053 | -0.483 | 0.049 | -0.602 |
| SSO2625 | -0.478 | -0.3 | -0.296 | -0.6 |
| SSO0989 | 0.333 | -0.329 | -0.391 | -0.6 |
| SSO3090 | -0.516 | -0.698 | -0.21 | -0.598 |
| SSO5542 | 0.066 | -0.477 | -0.736 | -0.595 |
| SSO0029 | 0.036 | -0.721 | -0.42 | -0.595 |
| SSO2770 | -0.112 | -0.148 | -0.469 | -0.595 |
| SSO2762 | -0.026 | -0.288 | -0.061 | -0.592 |
| SSO0315 | -0.177 | -0.384 | -0.633 | -0.588 |
| SSO1253 | -0.184 | -0.418 | -0.574 | -0.588 |
| SSO1098 | -0.429 | -0.48 | -0.899 | -0.583 |
| SSO0857 | -0.48 | -0.818 | -0.26 | -0.583 |
| SSO0344 | -0.181 | -0.428 | -0.012 | -0.581 |
| SSO3006 | -0.143 | -0.452 | 0.087 | -0.581 |
| SSO0553 | 0.112 | -0.45 | -0.016 | -0.581 |
| SSO0429 | -0.215 | -0.661 | -0.225 | -0.578 |
| SSO2619 | 0.259 | -0.567 | -0.394 | -0.575 |
| SSO0415 | -0.057 | -0.024 | -0.43 | -0.575 |
| SSO2231 | -0.173 | -0.592 | -0.039 | -0.571 |
| SSO2407 | -0.39 | -0.519 | -0.472 | -0.568 |
| SSO2184 | -0.107 | -0.149 | -0.135 | -0.565 |
| SSO0905 | 0.387 | -0.38 | -0.073 | -0.564 |
| SSO2766 | -0.378 | -0.57 | -0.364 | -0.561 |
| SSO3104 | -0.107 | -0.47 | -0.443 | -0.56 |
| SSO1004 | -0.097 | -0.42 | 0.169 | -0.556 |
| SSO1024 | -0.505 | -0.669 | -0.268 | -0.555 |
| SSO0240 | -0.2 | -0.44 | -0.247 | -0.555 |
| SSO0107 | 0.25 | -0.166 | -0.799 | -0.555 |
| SSO0764 | -0.176 | -0.444 | -0.141 | -0.552 |
| SSO0566 | -0.137 | -0.569 | -0.042 | -0.55 |
| SSO0293 | 0.721 | -0.244 | -0.191 | -0.549 |
| SSO0862 | -0.444 | -0.717 | -0.23 | -0.548 |
| SSO5826 | -0.088 | -0.377 | -0.806 | -0.548 |
| SSO1419 | -0.551 | -0.963 | -0.319 | -0.547 |
| SSO3100 | -0.216 | -0.395 | -0.66 | -0.547 |
| SSO1275 | 0.119 | -0.243 | -0.314 | -0.547 |
| SSO1515 | -0.977 | -0.405 | -0.523 | -0.546 |
| SSO2585 | 0.173 | -0.252 | -0.518 | -0.545 |
| SSO1274 | -0.035 | -0.319 | -0.227 | -0.544 |
| SSO0215 | 0.016 | -0.624 | 0.174 | -0.544 |
| SSO1003 | -0.374 | -1.052 | -0.129 | -0.543 |
| SSO5671 | -0.319 | -0.922 | -0.253 | -0.541 |
| SSO1063 | -0.34 | -0.725 | -0.526 | -0.538 |
| SSO0411 | -0.244 | -0.511 | -0.256 | -0.537 |
| SSO1519 | -0.214 | -0.241 | -0.36 | -0.537 |
| SSO2789 | 0.17 | -0.375 | -0.436 | -0.537 |
| SSO12127 | -0.298 | -0.062 | 0.354 | -0.537 |
| SSO3084 | -0.54 | -0.715 | -0.059 | -0.535 |
| SSO1119 | -0.603 | -0.819 | -0.31 | -0.534 |
| SSO0050 | -0.1 | -0.344 | -0.608 | -0.534 |
| SSO2588 | -0.065 | -0.533 | -0.166 | -0.534 |
| SSO2551 | 0.059 | -0.438 | -0.356 | -0.534 |
| SSO2962 | -0.346 | -0.483 | -0.249 | -0.533 |
| SSO3216 | 0.645 | -0.005 | -0.339 | -0.532 |
| SSO2092 | 0.518 | -0.175 | -0.554 | -0.531 |
| SSO1500 | -0.735 | -0.627 | 0.019 | -0.527 |
| SSO3127 | 0.046 | -0.408 | -0.326 | -0.527 |
| SSO0564 | -0.067 | -0.412 | 0.181 | -0.526 |
| SSO2179 | 0.077 | -0.352 | -0.313 | -0.525 |
| SSO0073 | -0.219 | -0.462 | -0.238 | -0.524 |
| SSO0983 | -0.076 | -0.471 | -0.221 | -0.521 |
| SSO1255 | -0.36 | -0.485 | -0.486 | -0.52 |
| SSO1524 | -0.712 | -0.385 | -0.198 | -0.52 |
| SSO2229 | -0.139 | -0.455 | -0.254 | -0.516 |
| SSO2486 | -0.334 | -0.505 | -0.172 | -0.513 |
| SSO2712 | -0.24 | -0.712 | 0.008 | -0.512 |
| SSO0988 | -0.078 | -0.462 | -0.256 | -0.512 |
| SSO2282 | 0.007 | -0.55 | -0.113 | -0.512 |
| SSO0703 | 0.104 | -0.361 | -0.289 | -0.511 |
| SSO2780 | 0.159 | NA | NA | -0.507 |
| SSO0700 | -0.129 | -0.579 | -0.113 | -0.506 |
| SSO2450 | -0.039 | -0.712 | -0.13 | -0.505 |
| SSO1398 | -0.165 | -0.307 | -0.347 | -0.504 |
| SSO0582 | 0.02 | -0.424 | -0.199 | -0.504 |
| SSO1987 | -0.381 | -0.543 | -0.244 | -0.503 |
| SSO1300 | 0.156 | -0.404 | -0.143 | -0.503 |
| SSO1489 | -0.858 | -0.284 | -0.689 | -0.502 |
| SSO2255 | -0.201 | -0.42 | -0.503 | -0.502 |
| SSO2363 | 0.256 | -0.417 | -0.079 | -0.502 |
| SSO11939 | -0.549 | -0.483 | -0.255 | -0.5 |
| SSO0223 | -0.388 | -0.629 | -0.179 | -0.499 |
| SSO1171 | -0.125 | -0.372 | -0.398 | -0.498 |
| SSO1060 | 0.373 | -0.232 | -0.176 | -0.498 |
| SSO2355 | -0.248 | -0.321 | -0.253 | -0.497 |
| SSO2589 | -0.288 | -0.585 | -0.2 | -0.496 |
| SSO0809 | -0.39 | -0.384 | -0.185 | -0.495 |
| SSO2279 | -0.103 | -0.608 | -0.243 | -0.492 |
| SSO0705 | 0.098 | -0.462 | 0.071 | -0.492 |
| SSO2043 | 0.338 | -0.254 | -0.266 | -0.491 |
| SSO2737 | -0.091 | -0.41 | -0.097 | -0.488 |
| SSO1807 | 0.508 | -0.52 | -0.908 | -0.487 |
| SSO1529 | -0.368 | -0.597 | -0.333 | -0.485 |
| SSO2583 | -0.127 | -0.142 | -0.334 | -0.485 |
| SSO2653 | 0.295 | -0.117 | -0.292 | -0.485 |
| SSO3088 | -0.374 | -0.337 | -0.212 | -0.484 |
| SSO0100 | -0.275 | -0.663 | 0.047 | -0.484 |
| SSO0444 | 0.179 | -0.426 | -0.138 | -0.484 |
| SSO0707 | 0.321 | -0.242 | -0.153 | -0.483 |
| SSO2463 | 0.059 | -0.336 | -0.212 | -0.479 |
| SSO0156 | 0.218 | -0.503 | 0.144 | -0.479 |
| SSO0035 | -0.067 | -0.842 | -0.822 | -0.478 |
| SSO6401 | -0.011 | -0.486 | 0.201 | -0.477 |
| SSO0637 | -0.199 | 0.028 | -0.791 | -0.476 |
| SSO0504 | -0.076 | -0.587 | -0.092 | -0.476 |
| SSO2595 | -0.07 | -0.306 | -0.564 | -0.475 |
| SSO0497 | -0.249 | -0.274 | -0.277 | -0.475 |
| SSO2874 | -0.117 | -0.408 | 0.001 | -0.475 |
| SSO1960 | -0.22 | -0.355 | -0.492 | -0.474 |
| SSO2623 | -0.153 | -0.373 | -0.302 | -0.474 |
| SSO0557 | 0.039 | -0.405 | -0.379 | -0.474 |
| SSO0704 | 0.298 | -0.272 | 0.031 | -0.467 |
| SSO0439 | 0.308 | -0.21 | -0.37 | -0.466 |
| SSO11114 | 0.046 | -0.115 | -0.118 | -0.465 |
| SSO2382 | 0.497 | -0.224 | -0.281 | -0.465 |
| SSO2449 | 0.718 | -0.021 | -0.324 | -0.464 |
| SSO1357 | -0.489 | -0.306 | -0.727 | -0.462 |
| SSO1992 | -0.593 | -0.563 | -0.365 | -0.462 |
| SSO0899 | -0.386 | -0.994 | -0.142 | -0.461 |
| SSO0452 | -0.169 | -0.275 | -0.349 | -0.46 |
| SSO2631 | -0.33 | -0.756 | 0.056 | -0.458 |
| SSO2624 | -0.21 | -0.7 | -0.197 | -0.457 |
| SSO2940 | -0.441 | -0.405 | -0.455 | -0.456 |
| SSO1168 | 0.04 | -0.301 | -0.095 | -0.456 |
| SSO3061 | -0.296 | -0.443 | -0.407 | -0.455 |
| SSO1400 | 0.043 | -0.374 | -0.229 | -0.455 |
| SSO0563 | 0.038 | -0.521 | 0.123 | -0.455 |
| SSO0305 | -0.151 | -0.346 | -0.038 | -0.451 |
| SSO0923 | -0.163 | -0.432 | 0.102 | -0.451 |
| SSO1512 | -0.883 | -0.929 | 0.179 | -0.45 |
| SSO2162 | -0.128 | -0.164 | 0.101 | -0.45 |
| SSO0708 | 0.444 | -0.269 | -0.098 | -0.45 |
| SSO1284 | -0.035 | -0.321 | -0.242 | -0.449 |
| SSO7114 | -0.109 | -0.231 | -0.153 | -0.449 |
| SSO0227 | 0.028 | -0.506 | 0.237 | -0.449 |
| SSO2471 | -0.07 | -0.591 | 0.243 | -0.448 |
| SSO6855 | 0.5 | -0.23 | -0.194 | -0.445 |
| SSO0702 | -0.111 | -0.451 | -0.168 | -0.444 |
| SSO0081 | 0.182 | -0.051 | -0.268 | -0.444 |
| SSO2541 | -0.307 | -0.248 | -0.322 | -0.442 |
| SSO2627 | -0.266 | -0.582 | -0.585 | -0.441 |
| SSO1277 | -0.132 | -0.472 | -0.275 | -0.441 |
| SSO0070 | -0.054 | -0.462 | 0.22 | -0.441 |
| SSO0534 | 0.33 | -0.481 | -0.086 | -0.441 |
| SSO0435 | -0.171 | -0.313 | -0.306 | -0.44 |
| SSO0071 | -0.278 | -0.268 | -0.123 | -0.44 |
| SSO3148 | -0.105 | -0.391 | -0.053 | -0.439 |
| SSO3011 | -0.231 | -0.144 | -0.456 | -0.438 |
| SSO0189 | -0.004 | -0.483 | -0.009 | -0.438 |
| SSO0558 | 0.055 | -0.374 | 0.209 | -0.437 |
| SSO0217 | 0.176 | -0.229 | 0.108 | -0.437 |
| SSO2747 | -0.137 | -0.249 | -0.277 | -0.436 |
| SSO0342 | -0.44 | -0.232 | -0.381 | -0.435 |
| SSO1074 | -0.209 | -0.034 | -0.305 | -0.435 |
| SSO2748 | -0.295 | -0.383 | -0.314 | -0.434 |
| SSO0163 | -0.021 | -0.383 | -0.265 | -0.434 |
| SSO2381 | 0.295 | -0.291 | -0.42 | -0.434 |
| SSO0742 | 0.228 | -0.092 | -0.5 | -0.434 |
| SSO0956 | 0.376 | -0.337 | -0.125 | -0.434 |
| SSO3000 | 0.289 | 0.04 | -0.107 | -0.432 |
| SSO1531 | -0.824 | -0.921 | -0.172 | -0.431 |
| SSO1401 | -0.133 | -0.367 | -0.239 | -0.431 |
| SSO2156 | 0.176 | -0.516 | -0.35 | -0.431 |
| SSO12252 | -0.12 | -0.209 | -0.75 | -0.429 |
| SSO2781 | -0.149 | -0.203 | -0.483 | -0.429 |
| SSO2110 | -0.164 | -0.265 | -0.374 | -0.429 |
| SSO0304 | -0.06 | -0.511 | -0.068 | -0.429 |
| SSO2234 | 0.013 | -0.595 | -0.185 | -0.427 |
| SSO5668 | -0.381 | -0.379 | -0.062 | -0.426 |
| SSO0287 | -0.102 | -0.347 | 0.017 | -0.425 |
| SSO2253 | -0.146 | -0.19 | -0.455 | -0.424 |
| SSO0488 | 0.411 | -0.184 | -0.484 | -0.424 |
| SSO2434 | -0.162 | -0.512 | 0.254 | -0.423 |
| SSO2637 | 0.115 | -0.22 | 0.005 | -0.421 |
| SSO2754 | -0.226 | -0.265 | -0.14 | -0.419 |
| SSO0594 | 0.208 | -0.303 | -0.04 | -0.419 |
| SSO0481 | -0.107 | -0.234 | -0.222 | -0.418 |
| SSO2963 | -0.149 | -0.399 | -0.386 | -0.417 |
| SSO2343 | -0.032 | -0.107 | -0.513 | -0.417 |
| SSO1175 | -0.261 | -0.823 | -0.075 | -0.416 |
| SSO0202 | -0.114 | -0.399 | -0.597 | -0.416 |
| SSO0180 | -0.48 | -0.722 | -0.165 | -0.414 |
| SSO2201 | 0.166 | -0.445 | -0.153 | -0.414 |
| SSO0102 | -0.239 | -0.428 | -0.384 | -0.413 |
| SSO0046 | 0.172 | -0.285 | -0.263 | -0.413 |
| SSO0467 | -0.031 | -0.353 | -0.586 | -0.412 |
| SSO1598 | -0.279 | -0.37 | 0.029 | -0.412 |
| SSO0554 | -0.074 | -0.483 | -0.102 | -0.411 |
| SSO0381 | 0.143 | -0.505 | 0.005 | -0.411 |
| SSO3103 | -0.071 | -0.261 | -0.28 | -0.41 |
| SSO0172 | -0.263 | -0.413 | -0.062 | -0.409 |
| SSO0555 | 0.024 | 0.05 | -0.167 | -0.409 |
| SSO1353 | -0.008 | -0.679 | -0.184 | -0.408 |
| SSO2989 | 0.029 | -0.288 | -0.211 | -0.408 |
| SSO1272 | 0.212 | -0.304 | -0.347 | -0.408 |
| SSO1435 | -0.76 | -0.704 | -0.482 | -0.406 |
| SSO1864 | -0.461 | -0.483 | -0.212 | -0.405 |
| SSO0661 | -0.214 | -0.559 | -0.086 | -0.405 |
| SSO2598 | 0.063 | -0.077 | -0.248 | -0.404 |
| SSO0244 | -0.105 | -0.455 | 0.045 | -0.402 |
| SSO2872 | 0.087 | -0.472 | 0.064 | -0.402 |
| SSO1318 | NA | NA | NA | -0.401 |
| SSO2292 | 0.114 | -0.344 | -0.429 | -0.401 |
| SSO2626 | -0.345 | -0.501 | -0.273 | -0.397 |
| SSO2553 | NA | 0.189 | NA | -0.397 |
| SSO0817 | -0.208 | 0.174 | -0.507 | -0.396 |
| SSO3083 | -0.099 | -0.322 | -0.402 | -0.395 |
| SSO0971 | 0.271 | -0.347 | 0.208 | -0.393 |
| SSO2634 | -0.637 | -0.155 | -0.245 | -0.391 |
| SSO0150 | 0.151 | -0.04 | -0.751 | -0.391 |
| SSO0963 | -0.08 | -0.313 | -0.023 | -0.391 |
| SSO1281 | -0.311 | -0.399 | -0.527 | -0.39 |
| SSO1657 | -0.074 | -0.39 | 0.076 | -0.39 |
| SSO2730 | 0.033 | -0.362 | -0.042 | -0.39 |
| SSO2556 | 0.188 | -0.052 | -0.506 | -0.39 |
| SSO1022 | -0.506 | -0.623 | -0.2 | -0.389 |
| SSO1254 | -0.239 | -0.399 | -0.164 | -0.389 |
| SSO0560 | 0.071 | -0.141 | -0.14 | -0.389 |
| SSO0090 | 0.094 | -0.343 | 0.052 | -0.389 |
| SSO2472 | 0.124 | -0.011 | NA | -0.389 |
| SSO2154 | 0.011 | -0.14 | -0.466 | -0.388 |
| SSO0219 | -0.206 | -0.134 | -0.654 | -0.386 |
| SSO0818 | 0.023 | -0.076 | -0.342 | -0.385 |
| SSO2832 | 0.046 | -0.223 | -0.116 | -0.385 |
| SSO2093 | 0.283 | -0.386 | -0.35 | -0.383 |
| SSO2934 | 0.005 | -0.338 | -0.064 | -0.382 |
| SSO0302 | 0.063 | -0.452 | 0.089 | -0.382 |
| SSO1530 | -0.531 | -0.764 | -0.125 | -0.381 |
| SSO0977 | -0.079 | -0.419 | -0.02 | -0.381 |
| SSO0111 | 0.34 | -0.44 | -0.055 | -0.38 |
| SSO2226 | -0.154 | -0.162 | 0.03 | -0.379 |
| SSO2462 | 0.048 | -0.109 | 0.024 | -0.378 |
| SSO12199 | 0.243 | -0.025 | -0.118 | -0.378 |
| SSO2964 | -0.707 | -0.316 | -0.515 | -0.377 |
| SSO2610 | 0.054 | -0.334 | -0.201 | -0.377 |
| SSO3129 | 0.229 | -0.195 | -0.268 | -0.377 |
| SSO0186 | 0.021 | -0.46 | -0.04 | -0.376 |
| SSO2880 | 0.599 | -0.085 | -0.239 | -0.376 |
| SSO2550 | -0.416 | -0.392 | -0.192 | -0.375 |
| SSO1597 | -0.036 | -0.517 | 0.073 | -0.375 |
| SSO2776 | -0.028 | -0.25 | -0.304 | -0.373 |
| SSO0031 | 0.06 | -0.436 | -0.055 | -0.373 |
| SSO0866 | -0.017 | -0.423 | -0.348 | -0.372 |
| SSO2060 | -0.121 | -0.416 | -0.054 | -0.372 |
| SSO0306 | 0.154 | -0.579 | 0.007 | -0.372 |
| SSO0225 | -0.611 | -0.393 | -0.095 | -0.371 |
| SSO2070 | 0.085 | -0.622 | -0.397 | -0.371 |
| SSO0774 | -0.028 | -0.53 | -0.225 | -0.37 |
| SSO2726 | 0.001 | -0.199 | -0.344 | -0.37 |
| SSO6397 | -0.096 | -0.295 | -0.046 | -0.37 |
| SSO2030 | 0.135 | -0.209 | -0.258 | -0.37 |
| SSO0349 | -0.463 | -0.447 | -0.245 | -0.367 |
| SSO1082 | -0.376 | -0.537 | -0.232 | -0.367 |
| SSO2390 | 0.017 | -0.263 | -0.312 | -0.367 |
| SSO2559 | -0.008 | -0.123 | -0.217 | -0.367 |
| SSO0051 | 0.076 | -0.327 | -0.194 | -0.366 |
| SSO2098 | -0.582 | -0.482 | 0.025 | -0.364 |
| SSO0913 | -0.101 | -0.33 | -0.138 | -0.363 |
| SSO3211 | -0.285 | -0.317 | 0.093 | -0.363 |
| SSO0625 | 0.361 | -0.392 | -0.096 | -0.363 |
| SSO0098 | 0.103 | -0.091 | -0.356 | -0.362 |
| SSO0709 | 0.332 | -0.074 | -0.209 | -0.362 |
| SSO1493 | -0.017 | -0.26 | 0.41 | -0.361 |
| SSO1842 | -0.539 | -0.504 | -0.443 | -0.36 |
| SSO1510 | -0.503 | -0.669 | -0.024 | -0.36 |
| SSO1169 | 0.197 | -0.5 | -0.087 | -0.36 |
| SSO2676 | -0.051 | -0.173 | -0.397 | -0.359 |
| SSO0768 | 0.205 | -0.421 | 0.042 | -0.359 |
| SSO1078 | 0.107 | -0.204 | -0.041 | -0.357 |
| SSO0752 | -0.357 | -0.001 | -0.415 | -0.356 |
| SSO2796 | -0.052 | -0.422 | -0.22 | -0.355 |
| SSO2346 | -0.074 | -0.251 | -0.234 | -0.355 |
| SSO0417 | -0.057 | -0.251 | 0.155 | -0.355 |
| SSO0469 | 0.055 | -0.471 | 0.084 | -0.353 |
| SSO1939 | -0.532 | -0.601 | -0.252 | -0.352 |
| SSO0627 | -0.236 | -0.205 | -0.522 | -0.352 |
| SSO0286 | 0.132 | -0.146 | -0.094 | -0.352 |
| SSO0083 | 0.52 | -0.145 | -0.113 | -0.352 |
| SSO2219 | -0.025 | -0.282 | -0.442 | -0.351 |
| SSO2508 | -0.078 | -0.254 | 0.044 | -0.351 |
| SSO1178 | -0.3 | -0.095 | -0.293 | -0.35 |
| SSO1095 | -0.193 | -0.354 | 0.187 | -0.35 |
| SSO3071 | -0.667 | -0.441 | -0.401 | -0.349 |
| SSO2266 | -0.137 | -0.499 | -0.163 | -0.349 |
| SSO2391 | 0.015 | -0.324 | -0.016 | -0.346 |
| SSO0889 | 0.308 | -0.474 | 0.302 | -0.346 |
| SSO1100 | -0.45 | -0.351 | -0.564 | -0.345 |
| SSO2474 | -0.259 | -0.168 | -0.226 | -0.345 |
| SSO1160 | -0.372 | -0.304 | -0.022 | -0.344 |
| SSO11934 | -0.495 | -0.378 | -0.087 | -0.343 |
| SSO0489 | 0.119 | -0.47 | -0.513 | -0.343 |
| SSO0660 | 0.024 | -0.328 | -0.139 | -0.343 |
| SSO2708 | -0.06 | 0.039 | -0.381 | -0.343 |
| SSO0579 | -0.1 | -0.356 | -0.431 | -0.341 |
| SSO0220 | -0.222 | -0.354 | -0.132 | -0.34 |
| SSO1423 | -0.671 | -0.612 | -0.17 | -0.339 |
| SSO2248 | -0.626 | -0.792 | -0.164 | -0.338 |
| SSO2524 | -0.096 | -0.504 | 0.04 | -0.338 |
| SSO3117 | 0.074 | -0.348 | 0.168 | -0.338 |
| SSO0234 | -0.003 | NA | NA | -0.337 |
| SSO0470 | -0.214 | -0.316 | -0.126 | -0.336 |
| SSO3050 | -0.058 | -0.374 | -0.184 | -0.336 |
| SSO2135 | 0.307 | -0.269 | 0.209 | -0.336 |
| SSO0696 | -0.431 | -0.276 | -0.249 | -0.335 |
| SSO0701 | -0.133 | -0.463 | -0.267 | -0.335 |
| SSO2083 | 0.104 | 0.156 | -0.827 | -0.335 |
| SSO0273 | -0.102 | -0.223 | -0.277 | -0.334 |
| SSO1252 | 0.006 | -0.142 | -0.216 | -0.334 |
| SSO0378 | 0.073 | -0.153 | -0.234 | -0.334 |
| SSO0067 | -0.126 | -0.319 | -0.246 | -0.333 |
| SSO2875 | -0.137 | -0.387 | 0.033 | -0.333 |
| SSO1427 | -0.1 | -0.429 | -0.033 | -0.332 |
| SSO1023 | -0.089 | -0.227 | NA | -0.331 |
| SSO1065 | 0.292 | -0.012 | -0.103 | -0.331 |
| SSO0531 | 0.479 | -0.218 | -0.016 | -0.33 |
| SSO2773 | -0.193 | -0.004 | -0.169 | -0.329 |
| SSO0166 | 0.037 | -0.374 | 0.033 | -0.329 |
| SSO2594 | -0.075 | -0.139 | -0.086 | -0.329 |
| SSO1691 | -1.355 | -0.483 | -0.078 | -0.328 |
| SSO0713 | 0.293 | -0.399 | -0.203 | -0.328 |
| SSO3198 | 0.343 | -0.388 | 0.157 | -0.327 |
| SSO3053 | -0.29 | -0.64 | -0.032 | -0.326 |
| SSO0386 | 0.121 | -0.16 | -0.265 | -0.326 |
| SSO2167 | 0.191 | -0.231 | -0.22 | -0.326 |
| SSO1574 | NA | NA | -1.047 | -0.325 |
| SSO6877 | 0.059 | -0.228 | -0.016 | -0.324 |
| SSO0193 | -0.071 | -0.193 | -0.358 | -0.323 |
| SSO0468 | -0.02 | -0.15 | -0.183 | -0.323 |
| SSO0393 | -0.122 | -0.434 | -0.05 | -0.32 |
| SSO1675 | NA | -0.163 | 0.14 | -0.32 |
| SSO2979 | -0.18 | -0.092 | -0.473 | -0.319 |
| SSO6264 | 0.132 | -0.562 | -0.171 | -0.319 |
| SSO3009 | -0.065 | -0.206 | 0.008 | -0.318 |
| SSO2063 | -0.51 | -0.793 | -0.008 | -0.317 |
| SSO2677 | -0.179 | 0.001 | -0.57 | -0.317 |
| SSO0938 | 0.022 | -0.587 | -0.029 | -0.317 |
| SSO0173 | -0.108 | -0.14 | -0.013 | -0.317 |
| SSO1334 | -0.723 | -0.519 | 0.068 | -0.316 |
| SSO0428 | -0.45 | -0.261 | -0.181 | -0.316 |
| SSO0279 | 0.043 | -0.147 | -0.146 | -0.315 |
| SSO1270 | 0.098 | -0.29 | -0.217 | -0.314 |
| SSO5763 | -0.312 | -0.232 | -0.581 | -0.313 |
| SSO2710 | -0.008 | -0.024 | -0.156 | -0.313 |
| SSO2276 | -0.101 | -0.245 | -0.379 | -0.312 |
| SSO1266 | -0.044 | -0.295 | -0.319 | -0.312 |
| SSO5140 | 0.145 | -0.471 | 0.073 | -0.312 |
| SSO1091 | -0.354 | -0.43 | 0.03 | -0.311 |
| SSO1428 | -0.163 | -0.711 | -0.005 | -0.31 |
| SSO2581 | 0.049 | -0.272 | -0.059 | -0.31 |
| SSO0026 | -0.124 | -0.367 | -0.32 | -0.309 |
| SSO0351 | -0.196 | -0.475 | -0.026 | -0.309 |
| SSO3163 | 0.158 | -0.461 | 0.01 | -0.309 |
| SSO1093 | 0.123 | -0.05 | 0.027 | -0.309 |
| SSO1583 | -0.914 | -0.51 | -0.595 | -0.308 |
| SSO3004 | -0.131 | -0.273 | -0.133 | -0.308 |
| SSO3093 | 0.057 | -0.282 | -0.138 | -0.308 |
| SSO0769 | 0.171 | -0.389 | -0.05 | -0.308 |
| SSO0813 | -0.141 | -0.242 | -0.037 | -0.307 |
| SSO0751 | 0.211 | -0.204 | -0.163 | -0.307 |
| SSO2526 | 0.136 | -0.24 | 0.039 | -0.307 |
| SSO1663 | -0.183 | -0.441 | -0.046 | -0.306 |
| SSO0358 | 0.466 | -0.149 | -0.1 | -0.305 |
| SSO1322 | -0.728 | -0.057 | -0.652 | -0.304 |
| SSO0383 | 0.323 | -0.151 | -0.23 | -0.304 |
| SSO3060 | 0.239 | NA | NA | -0.304 |
| SSO1641 | -0.311 | -0.419 | -0.081 | -0.303 |
| SSO2064 | -0.264 | -0.484 | 0.046 | -0.303 |
| SSO0743 | -0.306 | -0.365 | -0.073 | -0.302 |
| SSO2527 | 0.242 | 0.018 | -0.198 | -0.302 |
| SSO0941 | 0.405 | -0.196 | -0.048 | -0.302 |
| SSO3064 | -0.203 | -0.342 | -0.1 | -0.3 |
| SSO0274 | 0.045 | -0.515 | 0.002 | -0.3 |
| SSO2572 | -0.027 | -0.369 | -0.045 | -0.3 |
| SSO0636 | -0.053 | -0.267 | -0.093 | -0.3 |
| SSO1787 | -0.734 | -0.243 | -0.214 | -0.299 |
| SSO0919 | 0.286 | -0.168 | -0.027 | -0.299 |
| SSO2611 | 0.024 | -0.155 | -0.377 | -0.298 |
| SSO0052 | 0.109 | -0.23 | -0.212 | -0.298 |
| SSO1975 | -0.332 | -0.214 | -0.083 | -0.297 |
| SSO2849 | -0.104 | -0.36 | 0.23 | -0.297 |
| SSO5410 | 0.302 | -0.064 | -0.148 | -0.297 |
| SSO2852 | -0.206 | -0.271 | 0.043 | -0.296 |
| SSO0325 | -0.347 | -0.29 | -0.219 | -0.295 |
| SSO3239 | -0.006 | -0.627 | -0.078 | -0.295 |
| SSO2651 | -0.014 | -0.664 | 0.167 | -0.295 |
| SSO10449 | 0.039 | 0.122 | -0.403 | -0.294 |
| SSO1701 | -1.221 | -0.926 | -0.34 | -0.293 |
| SSO5798 | -0.167 | -0.094 | -0.354 | -0.293 |
| SSO0641 | -0.343 | -0.379 | 0.215 | -0.292 |
| SSO1988 | -0.021 | -0.403 | -0.22 | -0.291 |
| SSO0914 | -0.053 | NA | -0.189 | -0.29 |
| SSO1010 | 0.073 | -0.222 | -0.145 | -0.289 |
| SSO2554 | 0.246 | -0.249 | 0.041 | -0.289 |
| SSO1038 | 0.132 | -0.41 | -0.307 | -0.287 |
| SSO0722 | -0.231 | -0.303 | 0.278 | -0.287 |
| SSO1947 | 0.056 | -0.208 | -0.089 | -0.287 |
| SSO0091 | -2.395 | -0.459 | -0.408 | -0.286 |
| SSO1941 | -0.341 | -0.462 | -0.118 | -0.285 |
| SSO1306 | 0.16 | -0.253 | -0.204 | -0.284 |
| SSO0016 | -0.117 | -0.09 | -0.064 | -0.283 |
| SSO0819 | -0.286 | -0.546 | -0.019 | -0.282 |
| SSO2182 | -0.03 | -0.514 | -0.107 | -0.282 |
| SSO0307 | 0.123 | -0.212 | 0.106 | -0.281 |
| SSO1018 | -0.462 | -0.507 | -0.386 | -0.28 |
| SSO11614 | 0.077 | -0.122 | -0.349 | -0.28 |
| SSO0366 | 0.095 | 0.006 | -0.139 | -0.28 |
| SSO0397 | 0.192 | -0.06 | -0.068 | -0.28 |
| SSO1325 | -0.335 | -0.471 | -0.101 | -0.277 |
| SSO0245 | -0.238 | -0.549 | -0.032 | -0.277 |
| SSO0440 | 0.053 | -0.254 | -0.11 | -0.277 |
| SSO0699 | -0.123 | -0.202 | -0.218 | -0.276 |
| SSO2247 | -0.47 | -0.188 | -0.428 | -0.275 |
| SSO0546 | 0.015 | -0.244 | -0.087 | -0.275 |
| SSO8998 | -0.103 | -0.304 | 0.073 | -0.274 |
| SSO0078 | 0.143 | -0.284 | -0.026 | -0.274 |
| SSO2665 | -0.319 | -0.421 | -0.248 | -0.272 |
| SSO0628 | 0.282 | -0.451 | 0.061 | -0.272 |
| SSO1153 | 0.207 | -0.155 | -0.031 | -0.27 |
| SSO2397 | -0.157 | -0.136 | -0.275 | -0.269 |
| SSO0155 | -0.089 | -0.29 | 0.239 | -0.269 |
| SSO2158 | 0.535 | -0.215 | -0.274 | -0.269 |
| SSO0008 | -0.029 | -0.497 | -0.068 | -0.268 |
| SSO2647 | 0.259 | -0.201 | 0.088 | -0.268 |
| SSO3188 | -0.258 | -0.218 | -0.048 | -0.267 |
| SSO1331 | 0.14 | -0.297 | -0.254 | -0.267 |
| SSO3078 | 0.223 | -0.193 | -0.429 | -0.267 |
| SSO0496 | 0.12 | -0.122 | -0.331 | -0.267 |
| SSO0670 | 0.204 | 0.017 | -0.592 | -0.266 |
| SSO8478 | 0.03 | 0.005 | -0.145 | -0.266 |
| SSO2263 | 0.35 | -0.327 | -0.106 | -0.266 |
| SSO2291 | -0.042 | -0.42 | -0.284 | -0.265 |
| SSO0231 | 0.177 | -0.317 | -0.09 | -0.265 |
| SSO2275 | 0.307 | -0.225 | 0.038 | -0.265 |
| SSO2452 | 0.281 | -0.178 | -0.011 | -0.264 |
| SSO1618 | 0.188 | -0.022 | -0.023 | -0.264 |
| SSO1271 | -0.103 | -0.206 | -0.431 | -0.263 |
| SSO2062 | -0.705 | -0.738 | 0.23 | -0.262 |
| SSO0929 | -0.218 | -0.473 | 0.534 | -0.262 |
| SSO0461 | 0.166 | -0.135 | -0.531 | -0.261 |
| SSO0692 | 0.067 | -0.056 | -0.187 | -0.261 |
| SSO2383 | 0.777 | -0.319 | -0.242 | -0.261 |
| SSO2552 | -0.335 | -0.371 | -0.279 | -0.26 |
| SSO2719 | -0.013 | -0.333 | 0.022 | -0.26 |
| SSO5544 | 0.238 | 0.044 | -0.375 | -0.26 |
| SSO0572 | 0.434 | -0.194 | -0.2 | -0.26 |
| SSO0210 | 0.363 | -0.289 | -0.034 | -0.26 |
| SSO2607 | -0.277 | -0.293 | -0.282 | -0.259 |
| SSO2464 | 0.193 | -0.101 | -0.056 | -0.259 |
| SSO1291 | -0.133 | -0.437 | -0.261 | -0.258 |
| SSO1040 | -0.093 | -0.138 | -0.075 | -0.258 |
| SSO1319 | 0.28 | -0.127 | -0.176 | -0.258 |
| SSO1251 | -0.445 | -0.491 | -0.227 | -0.257 |
| SSO0556 | -0.051 | 0.041 | -0.262 | -0.257 |
| SSO0458 | 0.376 | -0.131 | -0.202 | -0.257 |
| SSO1552 | -0.212 | -0.503 | -0.128 | -0.256 |
| SSO1913 | -0.327 | -0.07 | -0.033 | -0.256 |
| SSO0317 | 0.199 | -0.273 | 0.01 | -0.256 |
| SSO2850 | -0.196 | -0.229 | 0.065 | -0.255 |
| SSO2620 | 0.014 | -0.262 | -0.153 | -0.254 |
| SSO0664 | 0.072 | -0.128 | 0.032 | -0.254 |
| SSO2310 | 0.188 | 0.028 | -0.145 | -0.253 |
| SSO0266 | -0.048 | -0.355 | 0.001 | -0.252 |
| SSO1570 | -0.517 | 0.104 | -0.382 | -0.251 |
| SSO2081 | 0.036 | -0.351 | -0.432 | -0.25 |
| SSO2380 | 0.064 | -0.066 | 0 | -0.249 |
| SSO0589 | 0.15 | -0.062 | 0.158 | -0.248 |
| SSO1050 | -0.026 | -0.391 | 0.07 | -0.247 |
| SSO3139 | -0.18 | -0.503 | -0.107 | -0.246 |
| SSO10285 | 0.244 | -0.088 | -0.254 | -0.246 |
| SSO2059 | 0.016 | -0.395 | -0.257 | -0.245 |
| SSO3136 | -0.131 | -0.202 | -0.132 | -0.245 |
| SSO3240 | 0.036 | -0.298 | -0.029 | -0.244 |
| SSO3243 | 0.25 | -0.234 | -0.024 | -0.244 |
| SSO0160 | 0.115 | -0.158 | 0.251 | -0.243 |
| SSO1990 | -0.07 | -0.371 | -0.105 | -0.242 |
| SSO0157 | 0.22 | -0.369 | 0.233 | -0.242 |
| SSO2010 | -0.146 | -0.368 | -0.023 | -0.241 |
| SSO0074 | -0.19 | -0.143 | -0.11 | -0.241 |
| SSO0597 | 0.044 | -0.293 | 0.077 | -0.24 |
| SSO0235 | -0.06 | 0.013 | -0.002 | -0.24 |
| SSO0405 | -0.098 | -0.116 | -0.752 | -0.239 |
| SSO1444 | -0.296 | -0.13 | -0.138 | -0.239 |
| SSO2983 | 0.236 | -0.017 | -0.126 | -0.239 |
| SSO3121 | 0.279 | -0.124 | 0.035 | -0.239 |
| SSO0233 | -0.111 | -0.346 | -0.108 | -0.238 |
| SSO2045 | 0.022 | -0.373 | -0.108 | -0.238 |
| SSO2191 | 0.148 | -0.117 | -0.195 | -0.238 |
| SSO0658 | 0.064 | -0.268 | 0.081 | -0.238 |
| SSO3010 | 0.299 | -0.144 | -0.204 | -0.237 |
| SSO0165 | -0.108 | -0.039 | -0.065 | -0.236 |
| SSO10340 | -0.253 | -0.127 | -0.307 | -0.235 |
| SSO6206 | 0.256 | -0.043 | -0.136 | -0.235 |
| SSO2114 | NA | -0.234 | NA | -0.234 |
| SSO0668 | 0.032 | -0.275 | -0.04 | -0.234 |
| SSO1110 | 0.16 | -0.22 | -0.152 | -0.233 |
| SSO2996 | 0.158 | -0.122 | 0.04 | -0.233 |
| SSO0412 | -0.065 | -0.253 | 0.494 | -0.233 |
| SSO1141 | 0.21 | -0.359 | -0.232 | -0.232 |
| SSO2871 | -0.064 | -0.296 | -0.078 | -0.231 |
| SSO0053 | 0.257 | -0.306 | -0.3 | -0.231 |
| SSO3155 | -0.035 | 0.055 | -0.079 | -0.231 |
| SSO1905 | -0.611 | -0.306 | -0.546 | -0.23 |
| SSO1429 | -0.084 | -0.453 | -0.114 | -0.23 |
| SSO1055 | 0.102 | -0.162 | -0.215 | -0.23 |
| SSO0253 | 0.16 | -0.048 | -0.161 | -0.23 |
| SSO1808 | -0.141 | -0.214 | -0.018 | -0.229 |
| SSO0066 | 0.32 | -0.08 | -0.098 | -0.229 |
| SSO2942 | -0.367 | -0.292 | 0.07 | -0.228 |
| SSO3144 | -0.143 | -0.001 | -0.346 | -0.228 |
| SSO2727 | 0.07 | -0.248 | 0.057 | -0.228 |
| SSO1594 | 0.048 | -0.175 | -0.124 | -0.227 |
| SSO1195 | 0.069 | -0.209 | -0.099 | -0.227 |
| SSO0373 | -0.018 | 0.016 | -0.079 | -0.227 |
| SSO1986 | -0.605 | -0.299 | -0.1 | -0.226 |
| SSO0567 | -0.52 | 0.148 | -0.303 | -0.226 |
| SSO2803 | -0.034 | -0.295 | -0.136 | -0.226 |
| SSO2884 | 0.04 | -0.181 | -0.04 | -0.226 |
| SSO0094 | 0.331 | -0.126 | -0.009 | -0.223 |
| SSO1560 | -0.248 | -0.351 | -0.212 | -0.222 |
| SSO2978 | -0.147 | -0.153 | -0.144 | -0.222 |
| SSO2254 | -0.096 | -0.202 | -0.043 | -0.222 |
| SSO6374 | -0.478 | -0.339 | -0.149 | -0.221 |
| SSO0442 | -0.198 | -0.253 | -0.368 | -0.221 |
| SSO2352 | -0.064 | -0.101 | -0.2 | -0.221 |
| SSO1268 | 0.012 | -0.448 | -0.073 | -0.219 |
| SSO1566 | 0.236 | -0.236 | -0.127 | -0.218 |
| SSO1261 | 0.031 | -0.149 | -0.224 | -0.217 |
| SSO2753 | 0.1 | -0.324 | 0.014 | -0.217 |
| SSO1782 | 0.162 | -0.251 | 0.111 | -0.216 |
| SSO3214 | 0.321 | -0.253 | 0.002 | -0.216 |
| SSO1959 | -0.507 | -0.142 | 0.113 | -0.215 |
| SSO3032 | 0.08 | -0.367 | 0.201 | -0.215 |
| SSO2157 | 0.319 | -0.271 | -0.077 | -0.214 |
| SSO0501 | 0.104 | -0.08 | -0.37 | -0.213 |
| SSO12018 | 0.016 | 0.137 | -0.258 | -0.213 |
| SSO2775 | 0.281 | -0.348 | -0.032 | -0.213 |
| SSO2725 | -0.31 | -0.295 | -0.222 | -0.211 |
| SSO1640 | -0.232 | -0.052 | NA | -0.211 |
| SSO2309 | 0.049 | -0.367 | 0.075 | -0.21 |
| SSO0599 | 0.071 | -0.424 | 0.115 | -0.21 |
| SSO0987 | 0.415 | -0.216 | -0.131 | -0.21 |
| SSO2953 | -0.102 | -0.208 | -0.061 | -0.208 |
| SSO2586 | 0.159 | -0.334 | -0.043 | -0.208 |
| SSO5343 | -0.077 | 0.272 | -0.235 | -0.208 |
| SSO1056 | 0.17 | -0.172 | 0.072 | -0.208 |
| SSO0296 | 0.182 | -0.129 | -0.149 | -0.207 |
| SSO2678 | 0.011 | -0.331 | -0.315 | -0.206 |
| SSO1646 | -0.772 | -0.41 | 0.241 | -0.205 |
| SSO2774 | -0.006 | -0.181 | 0.005 | -0.205 |
| SSO0207 | 0.17 | -0.238 | 0.231 | -0.204 |
| SSO1346 | -0.331 | -0.091 | -0.147 | -0.203 |
| SSO2163 | 0.195 | -0.041 | -0.023 | -0.203 |
| SSO2511 | -0.167 | -0.232 | 0.1 | -0.202 |
| SSO2399 | 0.185 | -0.311 | -0.122 | -0.202 |
| SSO0250 | 0.037 | -0.05 | 0.184 | -0.202 |
| SSO0276 | -0.233 | -0.424 | -0.158 | -0.201 |
| SSO3251 | -0.073 | -0.097 | -0.507 | -0.201 |
| SSO3166 | 0.005 | -0.208 | -0.154 | -0.2 |
| SSO3197 | 0.03 | -0.44 | 0.246 | -0.2 |
| SSO3118 | -0.678 | 0.004 | -0.135 | -0.199 |
| SSO3080 | -0.04 | -0.109 | -0.351 | -0.199 |
| SSO2697 | -0.166 | 0.255 | -0.137 | -0.199 |
| SSO2636 | -0.686 | -0.144 | -0.065 | -0.198 |
| SSO0657 | -0.348 | -0.182 | -0.153 | -0.198 |
| SSO0010 | 0.093 | -0.204 | -0.235 | -0.198 |
| SSO0785 | 0.091 | -0.041 | -0.17 | -0.198 |
| SSO2736 | -0.092 | -0.262 | -0.084 | -0.197 |
| SSO3095 | 0.294 | -0.199 | -0.017 | -0.196 |
| SSO1901 | -0.599 | 0.028 | -0.032 | -0.194 |
| SSO6175 | -0.009 | -0.048 | -0.135 | -0.194 |
| SSO0281 | 0.119 | -0.074 | -0.113 | -0.194 |
| SSO2582 | -0.047 | -0.065 | -0.098 | -0.193 |
| SSO0635 | 0.156 | -0.247 | -0.042 | -0.193 |
| SSO2174 | 0.053 | -0.218 | 0.075 | -0.193 |
| SSO1404 | 0.006 | -0.33 | -0.213 | -0.192 |
| SSO2599 | 0.037 | -0.241 | -0.026 | -0.192 |
| SSO2186 | 0.204 | -0.075 | -0.228 | -0.192 |
| SSO2652 | 0.284 | -0.037 | -0.236 | -0.192 |
| SSO3003 | -0.559 | -0.128 | -0.155 | -0.191 |
| SSO0999 | -0.269 | -0.553 | 0.117 | -0.191 |
| SSO0585 | 0.023 | 0.174 | -0.219 | -0.191 |
| SSO2574 | -0.247 | 0.13 | -0.277 | -0.19 |
| SSO2453 | -0.068 | 0.031 | -0.318 | -0.19 |
| SSO0724 | 0.207 | 0.01 | -0.075 | -0.19 |
| SSO0147 | -0.246 | -0.236 | -0.236 | -0.189 |
| SSO0176 | 0.189 | -0.336 | 0.333 | -0.188 |
| SSO1258 | -0.149 | -0.218 | -0.275 | -0.186 |
| SSO1403 | 0.11 | -0.152 | -0.237 | -0.186 |
| SSO2988 | 0.311 | -0.182 | -0.089 | -0.186 |
| SSO0396 | 0.14 | -0.189 | -0.284 | -0.185 |
| SSO3115 | -0.211 | -0.184 | 0.091 | -0.185 |
| SSO0777 | 0.198 | NA | -0.166 | -0.185 |
| SSO11972 | -0.287 | -0.17 | -0.346 | -0.184 |
| SSO1393 | -0.208 | -0.227 | -0.067 | -0.184 |
| SSO0640 | -0.015 | -0.197 | 0.297 | -0.184 |
| SSO0348 | 0.002 | -0.581 | -0.138 | -0.183 |
| SSO1785 | -0.357 | -0.092 | -0.081 | -0.183 |
| SSO1105 | 0.306 | -0.008 | -0.122 | -0.181 |
| SSO2354 | -0.069 | -0.223 | -0.247 | -0.179 |
| SSO8549 | 0.054 | -0.162 | -0.014 | -0.179 |
| SSO0086 | 0.344 | -0.085 | -0.134 | -0.179 |
| SSO0454 | 0.002 | -0.236 | -0.074 | -0.178 |
| SSO0499 | 0.243 | -0.061 | -0.171 | -0.178 |
| SSO3007 | -0.254 | -0.255 | 0.062 | -0.177 |
| SSO0221 | -0.112 | -0.134 | -0.195 | -0.177 |
| SSO0569 | 0.088 | -0.126 | -0.264 | -0.177 |
| SSO1651 | -0.157 | -0.138 | 0.188 | -0.177 |
| SSO2377 | -0.069 | -0.39 | -0.115 | -0.176 |
| SSO3112 | 0.177 | -0.091 | -0.052 | -0.176 |
| SSO2890 | -0.123 | -0.259 | -0.116 | -0.175 |
| SSO3206 | -0.086 | -0.176 | -0.059 | -0.175 |
| SSO2244 | -0.539 | -0.215 | -0.117 | -0.174 |
| SSO0195 | -0.316 | -0.385 | -0.038 | -0.174 |
| SSO2288 | 0.268 | -0.316 | -0.394 | -0.173 |
| SSO2870 | -0.07 | -0.233 | -0.092 | -0.173 |
| SSO2863 | -0.144 | -0.39 | 0.183 | -0.173 |
| SSO2215 | -0.137 | -0.377 | -0.064 | -0.172 |
| SSO2510 | -0.104 | -0.365 | 0.144 | -0.172 |
| SSO6454 | 0.117 | -0.08 | -0.333 | -0.172 |
| SSO0723 | -0.153 | 0.079 | 0.067 | -0.172 |
| SSO1215 | NA | NA | NA | -0.171 |
| SSO0318 | -0.185 | -0.252 | 0.127 | -0.171 |
| SSO3120 | -0.082 | -0.085 | -0.158 | -0.17 |
| SSO2509 | 0.083 | 0.133 | -0.059 | -0.17 |
| SSO0441 | -0.033 | -0.224 | -0.252 | -0.167 |
| SSO0475 | 0.077 | -0.315 | -0.02 | -0.167 |
| SSO2387 | 0.021 | -0.284 | 0.114 | -0.167 |
| SSO2606 | 0.2 | -0.215 | -0.034 | -0.167 |
| SSO3241 | -0.026 | -0.363 | -0.215 | -0.166 |
| SSO2171 | -0.097 | -0.067 | -0.135 | -0.166 |
| SSO2044 | -0.105 | -0.278 | -0.059 | -0.165 |
| SSO2987 | 0.133 | 0.064 | -0.43 | -0.163 |
| SSO1790 | -0.28 | -0.289 | 0.099 | -0.162 |
| SSO2693 | -0.069 | -0.447 | 0.084 | -0.162 |
| SSO0427 | 0.199 | -0.105 | -0.122 | -0.162 |
| SSO2735 | -0.627 | -0.353 | -0.287 | -0.161 |
| SSO2118 | -0.054 | -0.16 | -0.073 | -0.161 |
| SSO2170 | 0.09 | 0.015 | -0.359 | -0.161 |
| SSO1075 | 0.104 | -0.117 | -0.231 | -0.161 |
| SSO2005 | NA | NA | NA | -0.16 |
| SSO2684 | 0.066 | -0.264 | 0.026 | -0.159 |
| SSO2768 | 0.071 | -0.047 | 0.074 | -0.159 |
| SSO2741 | 0.188 | 0.003 | -0.039 | -0.159 |
| SSO1945 | -0.211 | -0.087 | -0.247 | -0.158 |
| SSO1868 | 0.018 | -0.072 | -0.219 | -0.157 |
| SSO0808 | 0.047 | 0.233 | -0.209 | -0.157 |
| SSO0085 | 0.176 | -0.082 | 0.007 | -0.157 |
| SSO2243 | -0.591 | -0.233 | -0.173 | -0.156 |
| SSO2066 | -0.035 | -0.176 | -0.048 | -0.155 |
| SSO2936 | -0.142 | -0.38 | -0.071 | -0.154 |
| SSO2347 | -0.117 | -0.222 | 0.092 | -0.154 |
| SSO1106 | -0.016 | -0.07 | -0.137 | -0.154 |
| SSO2899 | -0.107 | 0.019 | -0.207 | -0.153 |
| SSO3205 | 0.262 | -0.2 | -0.235 | -0.153 |
| SSO0330 | -0.601 | -0.352 | -0.162 | -0.152 |
| SSO1860 | -0.035 | -0.356 | -0.209 | -0.152 |
| SSO2517 | -0.179 | -0.36 | 0.146 | -0.152 |
| SSO1997 | -0.303 | -0.448 | 0.473 | -0.152 |
| SSO1623 | -0.209 | -0.268 | -0.003 | -0.151 |
| SSO3008 | -0.115 | -0.04 | -0.11 | -0.151 |
| SSO10784 | -0.18 | NA | -0.164 | -0.15 |
| SSO1470 | -0.152 | 0.005 | -0.043 | -0.15 |
| SSO3099 | -0.085 | -0.144 | 0.069 | -0.149 |
| SSO1568 | 0.02 | 0.046 | -0.02 | -0.149 |
| SSO1356 | -0.231 | -0.322 | -0.018 | -0.148 |
| SSO0282 | -0.064 | -0.728 | -0.063 | -0.147 |
| SSO11020 | 0.128 | 0.092 | -0.318 | -0.147 |
| SSO2549 | -0.39 | -0.175 | -0.043 | -0.146 |
| SSO2211 | 0.013 | -0.211 | 0.235 | -0.146 |
| SSO0545 | 0.273 | -0.126 | -0.041 | -0.146 |
| SSO0049 | 0.317 | 0.085 | -0.264 | -0.146 |
| SSO3055 | -0.343 | -0.402 | -0.039 | -0.145 |
| SSO0314 | 0.142 | -0.133 | 0.089 | -0.145 |
| SSO2370 | -0.646 | -0.558 | -0.623 | -0.144 |
| SSO1494 | -0.087 | -0.189 | -0.169 | -0.144 |
| SSO1974 | -0.155 | 0.061 | -0.251 | -0.143 |
| SSO0583 | 0.07 | -0.023 | -0.18 | -0.143 |
| SSO0382 | 0.073 | -0.188 | 0.105 | -0.143 |
| SSO1028 | -0.271 | -0.132 | 0.018 | -0.142 |
| SSO3209 | -0.141 | -0.167 | 0.046 | -0.142 |
| SSO1858 | 0.057 | -0.247 | 0.036 | -0.142 |
| SSO0526 | 0.264 | -0.007 | -0.207 | -0.142 |
| SSO1092 | -0.611 | -0.462 | -0.053 | -0.141 |
| SSO2810 | 0.042 | -0.107 | -0.006 | -0.141 |
| SSO2094 | NA | -0.326 | 0.248 | -0.14 |
| SSO2224 | 0.073 | -0.097 | 0.077 | -0.14 |
| SSO3254 | 0.137 | -0.13 | 0.069 | -0.14 |
| SSO0730 | -0.273 | -0.057 | -0.244 | -0.139 |
| SSO3068 | -0.124 | -0.344 | 0.098 | -0.138 |
| SSO2848 | -0.218 | 0.016 | 0.007 | -0.138 |
| SSO1514 | -1.24 | -0.357 | 0.073 | -0.137 |
| SSO3052 | 0.058 | -0.429 | -0.351 | -0.137 |
| SSO1839 | -0.034 | -0.352 | 0 | -0.137 |
| SSO0940 | -0.002 | -0.19 | -0.14 | -0.137 |
| SSO5844 | 0.079 | 0.104 | -0.218 | -0.137 |
| SSO0602 | 0.057 | -0.148 | 0.104 | -0.137 |
| SSO1611 | -0.112 | -0.025 | -0.148 | -0.136 |
| SSO3178 | -0.303 | -0.024 | -0.235 | -0.135 |
| SSO1537 | -0.586 | -0.273 | -0.034 | -0.134 |
| SSO1636 | 0.119 | -0.272 | -0.091 | -0.134 |
| SSO3105 | 0.051 | -0.095 | 0.042 | -0.134 |
| SSO1262 | 0.255 | -0.174 | NA | -0.134 |
| SSO2990 | -0.034 | -0.026 | 0.028 | -0.133 |
| SSO2029 | 0.074 | -0.305 | -0.374 | -0.131 |
| SSO2336 | 0.271 | -0.008 | -0.211 | -0.131 |
| SSO2301 | 0.031 | -0.136 | 0.102 | -0.13 |
| SSO2718 | 0.095 | 0.022 | -0.006 | -0.13 |
| SSO2431 | -0.114 | 0.044 | -0.116 | -0.129 |
| SSO0629 | -0.037 | -0.409 | 0.06 | -0.128 |
| SSO8910 | -0.812 | -0.156 | -0.181 | -0.127 |
| SSO2937 | 0.122 | -0.103 | NA | -0.126 |
| SSO0438 | 0.011 | -0.068 | 0.049 | -0.126 |
| SSO0324 | -0.169 | -0.413 | 0.15 | -0.125 |
| SSO1689 | NA | NA | -0.087 | -0.125 |
| SSO2218 | 0.194 | -0.032 | -0.062 | -0.125 |
| SSO1424 | -0.642 | -0.397 | -0.063 | -0.124 |
| SSO1667 | -0.111 | -0.45 | 0.06 | -0.124 |
| SSO1439 | -0.278 | -0.286 | 0.142 | -0.124 |
| SSO1137 | -0.077 | -0.201 | -0.042 | -0.124 |
| SSO2808 | 0.097 | -0.372 | 0.155 | -0.124 |
| SSO1665 | -0.749 | -0.68 | -0.346 | -0.121 |
| SSO1542 | -0.239 | -0.59 | -0.227 | -0.121 |
| SSO2131 | -0.343 | -0.182 | -0.221 | -0.121 |
| SSO0757 | -0.131 | -0.004 | 0.005 | -0.121 |
| SSO0447 | 0.111 | 0.137 | -0.242 | -0.119 |
| SSO2172 | 0.145 | -0.326 | 0.082 | -0.117 |
| SSO6453 | -0.108 | 0.065 | 0.04 | -0.117 |
| SSO2190 | 0.161 | -0.104 | -0.151 | -0.115 |
| SSO2672 | 0.074 | -0.08 | -0.064 | -0.115 |
| SSO2805 | 0.096 | -0.442 | -0.059 | -0.113 |
| SSO2515 | -0.345 | 0.389 | -0.223 | -0.113 |
| SSO1865 | -0.249 | -0.129 | -0.466 | -0.112 |
| SSO0821 | -0.41 | -0.122 | -0.029 | -0.112 |
| SSO3107 | -0.086 | -0.17 | 0.18 | -0.112 |
| SSO2960 | 0.108 | -0.081 | -0.092 | -0.112 |
| SSO0198 | -0.441 | -0.581 | 0.229 | -0.111 |
| SSO1513 | -0.277 | -0.225 | -0.047 | -0.111 |
| SSO3015 | 0.036 | -0.329 | -0.081 | -0.111 |
| SSO2819 | -0.033 | -0.397 | 0.069 | -0.11 |
| SSO1740 | -1.068 | -0.364 | -0.079 | -0.109 |
| SSO2067 | -0.042 | -0.402 | -0.005 | -0.109 |
| SSO0732 | -0.013 | -0.269 | 0.216 | -0.109 |
| SSO1066 | 0.176 | -0.051 | -0.096 | -0.109 |
| SSO2337 | -0.083 | -0.256 | -0.258 | -0.108 |
| SSO0655 | -0.015 | 0.071 | -0.027 | -0.108 |
| SSO1471 | -0.63 | -0.236 | 0.031 | -0.107 |
| SSO1294 | -0.085 | -0.263 | -0.189 | -0.107 |
| SSO0669 | -0.066 | -0.314 | 0.076 | -0.107 |
| SSO1405 | -0.134 | -0.741 | -0.245 | -0.106 |
| SSO2573 | -0.198 | -0.06 | -0.575 | -0.105 |
| SSO0326 | -0.278 | -0.095 | -0.001 | -0.105 |
| SSO0278 | -0.11 | -0.104 | 0.137 | -0.105 |
| SSO1345 | 0.081 | -0.047 | -0.088 | -0.105 |
| SSO3138 | -0.102 | -0.291 | -0.096 | -0.104 |
| SSO2615 | -0.548 | -0.286 | 0.043 | -0.102 |
| SSO2635 | -0.163 | -0.294 | 0.051 | -0.102 |
| SSO0632 | -0.082 | -0.598 | -0.047 | -0.1 |
| SSO2424 | -0.17 | -0.018 | -0.257 | -0.1 |
| SSO2641 | -0.265 | -0.158 | 0.095 | -0.1 |
| SSO2101 | -0.541 | NA | 0.138 | -0.098 |
| SSO1914 | -0.089 | -0.022 | -0.039 | -0.098 |
| SSO0887 | -0.165 | 0.04 | -0.148 | -0.097 |
| SSO2334 | -0.081 | -0.146 | 0.067 | -0.097 |
| SSO1527 | -0.065 | -0.418 | 0.107 | -0.096 |
| SSO2640 | -0.273 | -0.039 | 0.108 | -0.096 |
| SSO0873 | -0.047 | NA | 0.074 | -0.096 |
| SSO2535 | -0.125 | -0.169 | 0.093 | -0.095 |
| SSO2521 | -0.335 | -0.329 | -0.189 | -0.094 |
| SSO0506 | 0.195 | -0.471 | -0.055 | -0.094 |
| SSO2705 | -0.147 | 0.046 | -0.109 | -0.094 |
| SSO2790 | 0.061 | -0.257 | -0.007 | -0.093 |
| SSO2800 | -0.1 | -0.202 | 0.173 | -0.093 |
| SSO5672 | 0.124 | -0.452 | -0.132 | -0.092 |
| SSO1898 | 0.102 | 0.015 | -0.103 | -0.092 |
| SSO1330 | -0.146 | 0.211 | -0.195 | -0.091 |
| SSO2732 | -0.149 | 0.194 | -0.171 | -0.089 |
| SSO0804 | -0.017 | -0.135 | -0.257 | -0.087 |
| SSO2970 | -0.289 | -0.209 | 0.449 | -0.087 |
| SSO0267 | 0.16 | -0.166 | -0.038 | -0.087 |
| SSO1395 | -0.321 | -0.128 | 0.068 | -0.085 |
| SSO2537 | -0.404 | -0.101 | 0.179 | -0.085 |
| SSO1932 | -0.467 | -0.319 | 0.297 | -0.084 |
| SSO1655 | -0.096 | -0.262 | -0.071 | -0.084 |
| SSO0920 | 0.171 | -0.223 | -0.126 | -0.084 |
| SSO2891 | 0.002 | 0.185 | -0.299 | -0.084 |
| SSO1626 | -0.934 | -0.196 | -0.022 | -0.082 |
| SSO1426 | -0.372 | -0.44 | -0.031 | -0.082 |
| SSO0408 | -0.218 | -0.05 | -0.207 | -0.082 |
| SSO1344 | -0.635 | -0.283 | 0.003 | -0.081 |
| SSO2668 | -0.033 | -0.403 | -0.176 | -0.08 |
| SSO2117 | 0.026 | 0.044 | -0.059 | -0.08 |
| SSO0032 | -0.577 | -0.232 | 0.08 | -0.079 |
| SSO3186 | -0.203 | -0.072 | -0.209 | -0.078 |
| SSO0959 | NA | NA | NA | -0.078 |
| SSO1786 | -0.486 | -0.437 | -0.191 | -0.077 |
| SSO1185 | -0.196 | -0.019 | -0.233 | -0.077 |
| SSO0676 | 0.026 | -0.319 | 0.087 | -0.076 |
| SSO0450 | -0.378 | -0.039 | -0.116 | -0.074 |
| SSO10237 | -0.156 | NA | NA | -0.073 |
| SSO0951 | -0.139 | 0.196 | -0.055 | -0.073 |
| SSO1635 | 0.097 | -0.204 | 0.043 | -0.072 |
| SSO0962 | -0.199 | 0.145 | -0.446 | -0.071 |
| SSO0455 | 0.01 | -0.094 | -0.04 | -0.071 |
| SSO0871 | 0.071 | 0.115 | -0.184 | -0.07 |
| SSO1441 | -0.402 | -0.299 | 0.194 | -0.069 |
| SSO1859 | -0.061 | 0.066 | -0.164 | -0.068 |
| SSO2664 | -0.007 | -0.011 | -0.185 | -0.067 |
| SSO1768 | NA | NA | NA | -0.067 |
| SSO1229 | NA | -0.019 | NA | -0.067 |
| SSO3228 | 0.091 | -0.017 | -0.044 | -0.067 |
| SSO2324 | NA | -0.328 | NA | -0.066 |
| SSO2236 | -0.198 | -0.143 | -0.041 | -0.064 |
| SSO0922 | -0.019 | -0.249 | -0.036 | -0.064 |
| SSO3067 | -0.053 | -0.461 | 0.102 | -0.063 |
| SSO0883 | -0.12 | -0.112 | -0.041 | -0.063 |
| SSO1148 | 0.182 | -0.046 | -0.33 | -0.063 |
| SSO2807 | -0.108 | -0.184 | -0.373 | -0.062 |
| SSO3043 | -0.085 | -0.481 | -0.041 | -0.062 |
| SSO0746 | -0.207 | -0.111 | -0.161 | -0.062 |
| SSO2969 | -0.14 | -0.128 | 0.197 | -0.062 |
| SSO5663 | 0.345 | 0.005 | -0.419 | -0.062 |
| SSO1469 | -0.49 | 0.069 | 0.121 | -0.061 |
| SSO0847 | -0.068 | -0.045 | 0.026 | -0.061 |
| SSO2406 | -0.176 | -0.069 | 0.102 | -0.06 |
| SSO1861 | -0.253 | -0.262 | -0.064 | -0.059 |
| SSO2225 | -0.05 | -0.198 | -0.014 | -0.059 |
| SSO1980 | 0.242 | -0.203 | -0.067 | -0.059 |
| SSO2278 | -0.239 | 0.012 | -0.261 | -0.058 |
| SSO2518 | -0.253 | -0.279 | 0.05 | -0.058 |
| SSO2555 | 0.04 | -0.064 | -0.065 | -0.058 |
| SSO0633 | -0.348 | -0.672 | 0.06 | -0.057 |
| SSO2680 | -0.046 | -0.246 | -0.179 | -0.057 |
| SSO0019 | 0.089 | -0.144 | -0.059 | -0.057 |
| SSO0128 | 0.07 | -0.265 | 0.109 | -0.057 |
| SSO2669 | 0.108 | -0.402 | 0.351 | -0.057 |
| SSO2954 | -0.097 | -0.233 | NA | -0.056 |
| SSO6418 | -0.261 | 0.143 | -0.141 | -0.056 |
| SSO3232 | -0.339 | -0.062 | 0.111 | -0.055 |
| SSO2022 | -0.246 | -0.227 | -0.03 | -0.053 |
| SSO0251 | 0.05 | 0.002 | -0.047 | -0.053 |
| SSO2238 | -0.345 | -0.543 | -0.39 | -0.052 |
| SSO2393 | -0.211 | -0.525 | -0.073 | -0.051 |
| SSO2245 | 0.064 | -0.11 | -0.013 | -0.051 |
| SSO3250 | 0.135 | -0.053 | -0.058 | -0.05 |
| SSO2896 | -0.101 | -0.285 | -0.018 | -0.049 |
| SSO2740 | -0.107 | -0.141 | -0.092 | -0.049 |
| SSO0025 | 0.1 | -0.133 | -0.046 | -0.049 |
| SSO0921 | -0.051 | -0.227 | 0.187 | -0.048 |
| SSO6687 | 0.041 | -0.269 | -0.886 | -0.047 |
| SSO0329 | -0.316 | -0.163 | -0.055 | -0.046 |
| SSO0327 | -0.266 | -0.012 | -0.043 | -0.045 |
| SSO1329 | -0.037 | -0.272 | 0.203 | -0.045 |
| SSO2830 | -0.004 | -0.144 | -0.027 | -0.043 |
| SSO2847 | -0.267 | -0.272 | -0.12 | -0.042 |
| SSO12142 | -0.066 | 0.087 | -0.085 | -0.041 |
| SSO2061 | -0.375 | -0.215 | 0.346 | -0.04 |
| SSO2134 | -0.031 | -0.066 | 0.08 | -0.04 |
| SSO1432 | -0.282 | -0.603 | 0.227 | -0.039 |
| SSO2485 | -0.056 | -0.03 | -0.217 | -0.039 |
| SSO2372 | 0.076 | -0.015 | -0.138 | -0.039 |
| SSO2088 | -0.044 | -0.714 | 0.285 | -0.038 |
| SSO1409 | NA | NA | NA | -0.038 |
| SSO0114 | 0.068 | 0.035 | -0.091 | -0.038 |
| SSO2249 | -0.39 | -0.343 | 0.165 | -0.037 |
| SSO9500 | -0.273 | 0.131 | -0.22 | -0.037 |
| SSO2246 | -0.08 | -0.244 | 0.05 | -0.037 |
| SSO1412 | 0.057 | -0.04 | -0.158 | -0.037 |
| SSO3141 | 0.112 | 0.052 | -0.138 | -0.037 |
| SSO0591 | 0.148 | -0.072 | -0.089 | -0.036 |
| SSO3245 | 0.044 | 0.009 | -0.262 | -0.035 |
| SSO0892 | 0.099 | -0.327 | 0.235 | -0.035 |
| SSO1083 | -0.485 | -0.12 | -0.127 | -0.034 |
| SSO3058 | -0.524 | -0.388 | 0.189 | -0.033 |
| SSO11133 | -0.239 | -0.325 | -0.001 | -0.033 |
| SSO2658 | -0.041 | -0.149 | 0.202 | -0.031 |
| SSO0654 | -0.362 | 0.022 | -0.02 | -0.03 |
| SSO1360 | -0.016 | -0.075 | -0.223 | -0.028 |
| SSO0154 | 0.034 | -0.303 | -0.043 | -0.028 |
| SSO2481 | 0.289 | -0.078 | -0.28 | -0.027 |
| SSO1085 | 0.002 | 0.071 | -0.093 | -0.027 |
| SSO0779 | -0.159 | -0.021 | 0.175 | -0.027 |
| SSO2116 | -0.155 | -0.24 | 0.052 | -0.026 |
| SSO2257 | -0.047 | -0.356 | 0.218 | -0.025 |
| SSO0187 | -0.106 | -0.413 | -0.095 | -0.024 |
| SSO2401 | -0.164 | -0.104 | -0.004 | -0.024 |
| SSO2786 | -0.174 | 0.332 | -0.306 | -0.024 |
| SSO0552 | 0.058 | -0.488 | 0.147 | -0.021 |
| SSO3132 | -0.061 | -0.069 | -0.108 | -0.021 |
| SSO2520 | -0.661 | -0.328 | 0.116 | -0.02 |
| SSO2097 | -0.209 | 0.048 | -0.173 | -0.02 |
| SSO0758 | -0.334 | -0.017 | -0.164 | -0.019 |
| SSO0626 | 0.136 | -0.294 | 0.155 | -0.019 |
| SSO0529 | 0.027 | -0.589 | 0.003 | -0.016 |
| SSO1445 | -0.157 | -0.413 | 0.055 | -0.016 |
| SSO2237 | -0.014 | -0.099 | -0.094 | -0.013 |
| SSO1540 | -0.666 | -0.963 | 0.079 | -0.011 |
| SSO2738 | NA | NA | NA | -0.011 |
| SSO1026 | -0.853 | -0.181 | -0.09 | -0.01 |
| SSO2691 | 0.176 | -0.08 | -0.197 | -0.01 |
| SSO1437 | 0.046 | -0.421 | 0.241 | -0.008 |
| SSO0718 | -0.042 | -0.043 | -0.03 | -0.008 |
| SSO2119 | -0.049 | -0.079 | 0.125 | -0.008 |
| SSO0203 | -0.577 | -0.256 | 0.038 | -0.007 |
| SSO1522 | -0.448 | -0.112 | -0.168 | -0.005 |
| SSO1780 | -0.111 | -0.026 | NA | -0.005 |
| SSO0162 | -0.094 | -0.174 | 0.271 | -0.005 |
| SSO1996 | 0.035 | -0.43 | 0.105 | -0.003 |
| SSO2777 | 0.022 | -0.015 | -0.084 | -0.002 |
| SSO3054 | -0.32 | -0.43 | -0.118 | -0.001 |
| SSO2612 | -0.045 | 0.222 | -0.303 | 0 |
| SSO5561 | NA | NA | -0.01 | 0 |
| SSO0874 | -0.458 | -0.141 | -0.043 | 0.001 |
| SSO1526 | -0.417 | -0.411 | 0.336 | 0.003 |
| SSO2656 | -0.05 | -0.283 | -0.026 | 0.005 |
| SSO2183 | 0.273 | -0.237 | -0.246 | 0.005 |
| SSO3170 | 0.001 | -0.1 | -0.042 | 0.008 |
| SSO0216 | -0.054 | -0.032 | -0.014 | 0.009 |
| SSO2795 | 0.092 | -0.214 | 0.025 | 0.009 |
| SSO2426 | 0.035 | -0.13 | -0.085 | 0.01 |
| SSO1321 | -0.068 | -0.281 | -0.025 | 0.011 |
| SSO12083 | -0.111 | 0.003 | -0.054 | 0.011 |
| SSO2507 | -0.531 | 0.301 | -0.016 | 0.013 |
| SSO1931 | -0.433 | -0.233 | -0.121 | 0.014 |
| SSO1589 | -0.04 | -0.042 | -0.114 | 0.015 |
| SSO2749 | -0.762 | 0.022 | 0.139 | 0.016 |
| SSO0072 | -0.233 | 0.039 | 0.025 | 0.019 |
| SSO3042 | 0.092 | -0.198 | -0.035 | 0.019 |
| SSO12256 | 0.039 | -0.157 | -0.005 | 0.021 |
| SSO1025 | -0.35 | NA | -0.142 | 0.023 |
| SSO1077 | -0.153 | 0.111 | -0.08 | 0.023 |
| SSO1545 | -0.464 | -0.223 | 0.192 | 0.024 |
| SSO2878 | -0.357 | -0.037 | 0.214 | 0.024 |
| SSO0867 | -0.439 | -0.342 | -0.17 | 0.026 |
| SSO2427 | -0.452 | 0.154 | -0.464 | 0.027 |
| SSO2764 | 0.002 | -0.177 | -0.002 | 0.027 |
| SSO1976 | -0.31 | -0.082 | -0.095 | 0.029 |
| SSO2544 | 0.129 | -0.1 | -0.06 | 0.029 |
| SSO2639 | -0.142 | -0.221 | 0.086 | 0.03 |
| SSO2386 | -0.173 | -0.511 | 0.319 | 0.032 |
| SSO1922 | -0.448 | 0.126 | -0.028 | 0.032 |
| SSO0842 | 0.034 | -0.384 | 0.172 | 0.032 |
| SSO2519 | -0.337 | -0.082 | 0.142 | 0.034 |
| SSO1299 | -0.081 | -0.341 | NA | 0.035 |
| SSO1562 | -0.183 | 0.1 | -0.126 | 0.037 |
| SSO2783 | -0.259 | -0.216 | 0.135 | 0.038 |
| SSO1468 | -0.426 | -0.321 | 0.315 | 0.039 |
| SSO2522 | -0.205 | -0.176 | 0.122 | 0.042 |
| SSO0079 | -0.323 | -0.357 | -0.034 | 0.043 |
| SSO0298 | -0.32 | -0.012 | 0.162 | 0.046 |
| SSO8620 | -0.206 | NA | NA | 0.05 |
| SSO0485 | -0.062 | -0.29 | -0.078 | 0.052 |
| SSO2112 | -0.169 | -0.112 | 0.174 | 0.053 |
| SSO1265 | 0.177 | -0.158 | -0.08 | 0.056 |
| SSO1711 | -0.122 | -0.058 | -0.116 | 0.057 |
| SSO3039 | 0.044 | -0.432 | 0.214 | 0.057 |
| SSO2570 | 0.022 | 0.023 | -0.129 | 0.058 |
| SSO5761 | -0.275 | 0.032 | -0.178 | 0.059 |
| SSO2273 | -0.249 | 0.006 | -0.185 | 0.064 |
| SSO0648 | -0.084 | 0.377 | -0.415 | 0.071 |
| SSO2028 | -0.355 | -0.05 | -0.144 | 0.072 |
| SSO1340 | -0.149 | -0.259 | 0.05 | 0.076 |
| SSO0728 | -0.235 | -0.14 | 0.181 | 0.076 |
| SSO1811 | -0.155 | -0.313 | 0.109 | 0.078 |
| SSO1421 | -0.845 | -0.365 | -0.013 | 0.079 |
| SSO2971 | -0.47 | 0.001 | 0.355 | 0.079 |
| SSO0970 | -0.233 | -0.028 | -0.058 | 0.089 |
| SSO3019 | -0.383 | -0.133 | 0.143 | 0.09 |
| SSO2618 | -0.296 | 0.084 | 0.108 | 0.091 |
| SSO2484 | 0.2 | -0.238 | -0.097 | 0.095 |
| SSO1539 | -0.172 | -0.129 | -0.03 | 0.106 |
| SSO0645 | -0.231 | -0.102 | 0.152 | 0.107 |
| SSO2141 | -0.075 | -0.586 | 0.5 | 0.113 |
| SSO1505 | -0.017 | -0.116 | 0.018 | 0.113 |
| SSO1789 | -0.473 | -0.242 | 0.089 | 0.116 |
| SSO0761 | -0.526 | -0.42 | 0.798 | 0.116 |
| SSO1739 | -0.495 | -0.246 | -0.039 | 0.117 |
| SSO1465 | -0.262 | -0.2 | -0.015 | 0.12 |
| SSO0833 | -0.738 | 0.068 | -0.157 | 0.124 |
| SSO2468 | 0.012 | -0.431 | 0.154 | 0.125 |
| SSO2827 | -0.325 | -0.299 | 0.017 | 0.126 |
| SSO3111 | -0.45 | 0.332 | -0.047 | 0.133 |
| SSO1668 | 0.103 | -0.487 | 0.004 | 0.134 |
| SSO0388 | 0.205 | -0.14 | -0.361 | 0.134 |
| SSO1838 | -1.285 | -1.085 | 0.278 | 0.136 |
| SSO1422 | -0.385 | NA | -0.027 | 0.136 |
| SSO0604 | -0.128 | -0.222 | 0.179 | 0.136 |
| SSO2208 | -0.22 | -0.397 | -0.083 | 0.14 |
| SSO1532 | -0.866 | -0.188 | 0.128 | 0.145 |
| SSO2071 | -0.629 | -0.195 | -0.134 | 0.153 |
| SSO3041 | -0.021 | -0.157 | NA | 0.155 |
| SSO1310 | -0.184 | NA | NA | 0.16 |
| SSO1057 | -0.406 | -0.148 | -0.026 | 0.168 |
| SSO0057 | NA | -0.09 | -0.219 | 0.168 |
| SSO2613 | -0.223 | 0.083 | -0.359 | 0.169 |
| SSO2867 | -0.668 | 0.183 | 0.128 | 0.171 |
| SSO2603 | -0.393 | 0.095 | -0.095 | 0.173 |
| SSO1495 | -1.095 | 0.18 | -0.033 | 0.176 |
| SSO1752 | -0.649 | -0.035 | 0.131 | 0.18 |
| SSO0099 | -0.178 | -0.033 | 0.008 | 0.188 |
| SSO2442 | -0.606 | -0.213 | 0.163 | 0.202 |
| SSO0507 | -0.143 | -0.092 | -0.053 | 0.205 |
| SSO1438 | -0.48 | -0.248 | 0.249 | 0.21 |
| SSO2877 | -0.64 | 0.027 | 0.169 | 0.221 |
| SSO1433 | -0.663 | 0.139 | 0.101 | 0.241 |
| SSO2788 | -0.034 | NA | -0.47 | 0.243 |
| SSO1213 | -0.288 | -0.105 | -0.039 | 0.243 |
| SSO1039 | -0.078 | -0.303 | 0.114 | 0.244 |
| SSO2560 | -0.306 | -0.025 | 0.079 | 0.248 |
| SSO0814 | -0.633 | -0.08 | -0.126 | 0.265 |
| SSO1086 | -0.451 | 0.006 | -0.15 | 0.267 |
| SSO2126 | -0.657 | -0.129 | 0.159 | 0.293 |
| SSO6817 | -0.464 | -0.169 | 0.201 | 0.301 |
| SSO3180 | -0.467 | -0.93 | 0.232 | 0.309 |
| SSO2318 | NA | NA | -0.766 | 0.312 |
| SSO2609 | -0.054 | -0.296 | 0.005 | 0.335 |
| SSO0618 | -0.646 | -0.942 | 0.504 | 0.349 |
| SSO1533 | -0.811 | -0.334 | 0.127 | 0.351 |
| SSO2857 | -0.491 | NA | -0.477 | 0.415 |
| SSO1378 | NA | NA | -2.372 | NA |
| SSO6024 | NA | NA | -0.975 | NA |
| SSO0601 | -1.303 | NA | -0.581 | NA |
| SSO3059 | NA | -0.865 | NA | NA |
| SSO3257 | -0.83 | NA | NA | NA |
| SSO2914 | -0.171 | -0.735 | NA | NA |
| SSO0608 | 0.003 | NA | -0.817 | NA |
| SSO3049 | 0.059 | -0.653 | NA | NA |
| SSO0679 | -0.032 | -0.174 | -0.677 | NA |
| SSO1690 | -0.28 | NA | NA | NA |
| SSO1196 | -0.243 | NA | NA | NA |
| SSO10342 | 0.15 | NA | -0.534 | NA |
| SSO1666 | NA | -0.161 | NA | NA |
| SSO1116 | -0.143 | NA | NA | NA |
| SSO0109 | -0.13 | NA | NA | NA |
| SSO3230 | -0.073 | NA | -0.166 | NA |
| SSO2389 | -0.112 | -0.114 | NA | NA |
| SSO10224 | -0.095 | NA | NA | NA |
| SSO10269 | NA | -0.081 | NA | NA |
| SSO3097 | NA | -0.076 | NA | NA |
| SSO3108 | NA | NA | -0.075 | NA |
| SSO0004 | NA | NA | -0.072 | NA |
| SSO2085 | NA | NA | -0.072 | NA |
| SSO1234 | -0.071 | NA | NA | NA |
| SSO1777 | NA | NA | -0.054 | NA |
| SSO1765 | 0 | -0.092 | NA | NA |
| SSO2096 | NA | -0.031 | NA | NA |
| SSO0493 | NA | NA | -0.029 | NA |
| SSO7111 | -0.015 | -0.217 | 0.169 | NA |
| SSO1622 | 0.122 | -0.156 | NA | NA |
| SSO1305 | -0.01 | NA | NA | NA |
| SSO1949 | 0.175 | 0.32 | -0.518 | NA |
| SSO1569 | -0.006 | NA | NA | NA |
| SSO0828 | -0.086 | 0.263 | -0.193 | NA |
| SSO3034 | -0.283 | NA | 0.276 | NA |
