# Supplementary material for: Responses of hyperthermophilic crenarchaea to UV irradiation
Source: Genome Biol. 2007 Oct 11;8(10):R220. doi: 10.1186/gb-2007-8-10-r220 (PMC2246294; doi:10.1186/gb-2007-8-10-r220)
Supplement: Additional data file 6 — S. acidocaldarius genes repressed by UV irradiation. [file gb-2007-8-10-r220-S6.doc]

Table S6. Ratio of expression of all *S. acidocaldarius* genes repressed by UV irradiation, expressed as log_2_ (UV/ control) and ranked by degree of repression at 120 min.

| Gene | 30 min | 60 min | 90 min | 120 min |
| --- | --- | --- | --- | --- |
| Saci_0840 | -0.125 | -0.197 | -0.931 | -1.571 |
| Saci_0969 | 0.125 | -0.695 | -1.474 | -1.527 |
| Saci_1959 | -0.629 | -0.44 | -0.351 | -1.499 |
| Saci_1229 | 0.231 | -0.736 | -1.534 | -1.476 |
| Saci_0204 | 0.02 | -0.824 | -1.194 | -1.468 |
| Saci_1012 | -0.411 | -0.946 | -1.377 | -1.459 |
| Saci_1228 | -0.247 | -0.827 | -1.415 | -1.456 |
| Saci_0085 | -0.271 | -0.35 | -0.934 | -1.446 |
| Saci_0203 | -0.316 | -1.087 | -1.206 | -1.393 |
| Saci_0692 | -0.602 | -0.428 | -1.134 | -1.377 |
| Saci_0175 | -0.007 | -0.327 | -0.901 | -1.359 |
| Saci_0083 | -0.265 | -0.317 | -0.751 | -1.35 |
| Saci_1214 | 0.057 | -0.409 | -0.775 | -1.329 |
| Saci_1341 | 0.053 | -0.267 | -1.325 | -1.325 |
| Saci_0858 | 0.031 | -0.259 | -0.952 | -1.299 |
| Saci_1310 | 0.103 | -0.291 | -0.893 | -1.271 |
| Saci_1469 | -0.064 | 0.125 | -0.712 | -1.263 |
| Saci_2317 | -0.169 | -0.378 | -0.619 | -1.259 |
| Saci_1189 | -0.263 | -1.618 | -1.797 | -1.258 |
| Saci_0750 | 0.338 | -0.404 | -0.925 | -1.247 |
| Saci_0075 | -0.334 | 0.036 | -0.919 | -1.172 |
| Saci_1483 | 0.221 | 0.091 | -0.609 | -1.163 |
| Saci_0122 | -0.101 | -0.549 | -0.879 | -1.147 |
| Saci_0925 | -0.155 | -0.403 | -0.874 | -1.118 |
| Saci_0832 | -0.114 | -0.089 | -0.587 | -1.116 |
| Saci_1373 | 0.194 | -0.367 | -0.902 | -1.079 |
| Saci_1648 | 0.147 | -0.181 | -0.529 | -1.066 |
| Saci_0280 | -0.114 | -0.121 | -0.539 | -1.064 |
| Saci_0646 | -0.313 | 0.034 | -0.714 | -1.063 |
| Saci_0231 | -0.168 | -0.341 | -0.436 | -1.047 |
| Saci_1211 | 0.115 | -0.31 | -0.901 | -1.025 |
| Saci_1790 | 0.413 | -0.327 | -0.934 | -1.018 |
| Saci_1309 | 0.086 | -0.146 | -0.814 | -0.992 |
| Saci_1109 | 0.02 | -0.229 | -0.09 | -0.982 |
| Saci_1362 | -1.148 | -0.535 | -0.724 | -0.981 |
| Saci_0596 | -0.064 | 0.106 | -0.4 | -0.958 |
| Saci_0615 | -0.097 | -0.241 | -0.668 | -0.954 |
| Saci_1653 | 0.025 | -0.067 | -0.452 | -0.953 |
| Saci_0584 | -0.122 | -0.107 | -0.374 | -0.948 |
| Saci_0576 | -0.043 | -0.46 | -0.926 | -0.941 |
| Saci_0046 | 0.188 | -0.184 | -1.695 | -0.939 |
| Saci_1288 | -0.06 | -0.211 | -0.625 | -0.931 |
| Saci_2347 | -0.129 | -0.469 | -0.821 | -0.926 |
| Saci_0743 | -0.133 | -0.774 | -1.128 | -0.925 |
| Saci_2338 | -0.279 | -0.599 | -0.434 | -0.92 |
| Saci_1315 | 0.03 | -0.472 | -0.634 | -0.92 |
| Saci_0881 | -0.12 | -0.398 | -0.814 | -0.916 |
| Saci_1023 | -0.074 | -0.159 | -0.45 | -0.916 |
| Saci_1549 | -0.251 | -0.081 | -0.269 | -0.911 |
| Saci_1193 | -0.126 | -0.572 | -0.749 | -0.906 |
| Saci_1078 | -0.195 | -0.219 | -0.993 | -0.903 |
| Saci_0599 | 0.158 | -0.02 | -0.644 | -0.903 |
| Saci_0257 | -0.001 | -0.33 | -0.765 | -0.898 |
| Saci_1203 | -0.285 | 0.095 | -1.001 | -0.896 |
| Saci_0689 | -0.048 | -0.073 | -0.837 | -0.896 |
| Saci_0089 | -0.116 | -0.187 | -0.6 | -0.895 |
| Saci_1594 | 0.013 | -0.536 | -0.682 | -0.894 |
| Saci_1595 | -0.107 | -0.331 | -0.826 | -0.888 |
| Saci_0585 | -0.069 | -0.174 | -0.734 | -0.887 |
| Saci_0611 | -0.034 | 0.156 | -0.429 | -0.878 |
| Saci_0998 | 0.007 | -0.209 | -0.755 | -0.877 |
| Saci_0997 | 0.395 | -0.38 | -0.607 | -0.877 |
| Saci_1359 | -0.222 | -0.296 | -0.799 | -0.876 |
| Saci_0595 | -0.05 | -0.094 | -0.589 | -0.876 |
| Saci_1922 | -0.308 | -0.661 | -0.712 | -0.875 |
| Saci_1921 | -0.275 | -0.337 | -0.65 | -0.874 |
| Saci_1100 | -0.513 | -0.463 | -0.384 | -0.873 |
| Saci_1396 | -0.006 | -0.13 | -0.741 | -0.868 |
| Saci_0839 | -0.009 | -0.131 | -0.599 | -0.868 |
| Saci_0846 | -0.312 | -0.224 | -0.604 | -0.867 |
| Saci_0935 | -0.033 | -0.507 | -0.633 | -0.866 |
| Saci_2268 | -0.103 | -0.247 | -0.588 | -0.865 |
| Saci_1274 | 0.006 | -0.186 | -0.151 | -0.865 |
| Saci_1620 | -0.279 | -0.298 | -0.395 | -0.858 |
| Saci_0136 | 0.522 | -0.007 | -0.588 | -0.857 |
| Saci_0372 | -0.049 | -0.343 | -0.685 | -0.853 |
| Saci_1355 | -0.154 | -0.161 | -0.667 | -0.847 |
| Saci_0072 | 0.17 | -0.118 | -0.944 | -0.842 |
| Saci_1372 | -0.118 | -0.276 | -0.801 | -0.838 |
| Saci_0582 | 0.083 | -0.192 | -0.632 | -0.837 |
| Saci_0210 | -0.26 | -0.424 | -0.528 | -0.83 |
| Saci_0071 | -0.12 | -0.257 | -0.659 | -0.826 |
| Saci_0212 | -0.214 | -0.287 | -0.951 | -0.825 |
| Saci_1039 | -0.168 | -0.528 | -0.849 | -0.822 |
| Saci_0246 | -0.072 | 0.069 | -0.542 | -0.822 |
| Saci_0594 | -0.296 | -0.375 | -1.004 | -0.82 |
| Saci_1633 | -0.025 | -0.235 | -0.451 | -0.816 |
| Saci_1471 | -0.147 | -0.234 | -0.982 | -0.809 |
| Saci_0603 | 0.157 | 0.077 | -0.186 | -0.806 |
| Saci_1265 | 0.366 | -0.095 | -0.338 | -0.805 |
| Saci_1466 | -0.03 | 0.149 | -0.744 | -0.803 |
| Saci_1580 | 0.137 | -0.362 | -0.584 | -0.802 |
| Saci_0147 | -0.103 | -0.217 | -0.392 | -0.796 |
| Saci_1519 | -0.011 | -0.219 | -0.321 | -0.796 |
| Saci_1570 | -0.038 | -0.254 | -0.368 | -0.794 |
| Saci_0827 | -0.365 | 0.05 | -0.479 | -0.79 |
| Saci_1280 | -0.157 | -0.755 | -0.907 | -0.788 |
| Saci_0783 | -0.208 | -0.599 | -0.505 | -0.787 |
| Saci_1330 | -0.101 | -0.07 | -0.681 | -0.787 |
| Saci_0067 | -0.019 | -0.042 | -0.615 | -0.786 |
| Saci_0752 | 0.151 | 0.033 | -0.628 | -0.777 |
| Saci_0707 | -0.305 | -0.1 | -0.625 | -0.774 |
| Saci_1457 | -0.213 | -0.105 | -0.779 | -0.77 |
| Saci_0001 | -0.423 | 0.127 | -0.275 | -0.766 |
| Saci_1381 | -0.089 | -0.16 | -0.451 | -0.761 |
| Saci_2288 | 0.244 | -0.322 | -0.281 | -0.758 |
| Saci_0288 | 0.143 | -0.444 | -0.877 | -0.756 |
| Saci_0079 | 0.326 | -0.125 | -0.241 | -0.756 |
| Saci_1390 | 0.222 | 0.072 | -0.272 | -0.755 |
| Saci_1240 | 0.32 | 0.086 | -0.258 | -0.752 |
| Saci_1209 | -0.029 | -0.201 | -0.582 | -0.748 |
| Saci_0390 | 0.01 | -0.251 | -0.293 | -0.747 |
| Saci_0719 | 0.145 | 0.037 | -0.512 | -0.747 |
| Saci_0263 | 0.476 | 0.004 | -0.437 | -0.747 |
| Saci_0299 | 0.134 | -0.05 | -0.393 | -0.743 |
| Saci_2291 | 0.014 | -0.258 | -0.408 | -0.741 |
| Saci_1727 | -0.01 | -0.275 | -0.474 | -0.74 |
| Saci_1387 | 0.023 | 0.102 | -0.716 | -0.74 |
| Saci_2339 | -0.647 | -0.486 | -0.453 | -0.739 |
| Saci_0211 | -0.433 | -0.142 | -0.333 | -0.738 |
| Saci_0575 | -0.548 | -0.328 | -0.577 | -0.736 |
| Saci_0135 | -0.118 | -0.519 | -0.561 | -0.733 |
| Saci_1683 | -0.205 | -0.136 | -0.211 | -0.73 |
| Saci_0690 | -0.01 | 0.134 | -0.5 | -0.728 |
| Saci_2322 | 0.023 | -0.415 | -0.623 | -0.726 |
| Saci_1015 | 0.148 | -0.201 | -0.746 | -0.726 |
| Saci_0981 | 0.054 | -0.106 | -0.318 | -0.725 |
| Saci_0614 | -0.108 | -0.24 | -0.631 | -0.724 |
| Saci_1350 | -0.267 | -0.122 | -0.648 | -0.721 |
| Saci_0672 | 0.14 | 0.131 | -0.375 | -0.717 |
| Saci_0799 | -0.137 | 0.049 | -0.696 | -0.715 |
| Saci_0775 | -0.122 | -0.2 | -0.679 | -0.711 |
| Saci_2218 | 0.629 | -0.095 | -0.292 | -0.71 |
| Saci_1961 | -0.456 | -0.523 | -0.323 | -0.707 |
| Saci_2000 | -0.248 | -0.551 | -0.424 | -0.705 |
| Saci_1729 | -0.442 | -0.445 | -0.359 | -0.704 |
| Saci_0980 | -0.132 | 0.063 | -0.23 | -0.703 |
| Saci_0849 | 0.284 | -0.204 | -0.578 | -0.7 |
| Saci_0602 | 0.037 | -0.246 | -0.285 | -0.697 |
| Saci_1356 | -0.175 | -0.152 | -0.643 | -0.695 |
| Saci_0616 | 0.003 | -0.001 | -0.757 | -0.695 |
| Saci_1020 | 0.164 | -0.266 | -0.495 | -0.695 |
| Saci_1765 | -0.662 | -0.501 | -0.438 | -0.692 |
| Saci_1678 | -0.444 | -0.338 | -0.63 | -0.692 |
| Saci_1346 | -0.147 | -0.088 | -0.402 | -0.692 |
| Saci_0778 | -0.066 | -0.084 | -0.27 | -0.692 |
| Saci_1331 | 0.454 | 0.145 | -0.533 | -0.692 |
| Saci_2042 | -0.115 | -0.274 | -0.234 | -0.689 |
| Saci_1311 | 0.099 | -0.206 | -0.717 | -0.686 |
| Saci_0751 | 0.33 | 0.1 | -0.828 | -0.686 |
| Saci_0686 | 0.231 | 0.264 | -0.16 | -0.685 |
| Saci_0129 | 0.198 | -0.37 | -0.582 | -0.684 |
| Saci_2261 | 0.178 | 0.011 | -0.256 | -0.68 |
| Saci_0024 | 0.149 | -0.091 | -0.481 | -0.679 |
| Saci_1116 | -0.016 | -0.41 | -0.51 | -0.675 |
| Saci_2154 | 0.5 | -0.172 | -0.508 | -0.674 |
| Saci_0706 | -0.127 | 0.02 | -0.488 | -0.671 |
| Saci_1658 | -0.085 | -0.155 | -0.381 | -0.669 |
| Saci_0016 | 0.098 | -0.154 | -0.595 | -0.665 |
| Saci_0400 | 0.29 | -0.133 | -0.406 | -0.664 |
| Saci_1537 | 0.02 | -0.324 | -0.66 | -0.659 |
| Saci_0831 | -0.016 | 0.171 | -0.489 | -0.657 |
| Saci_0157 | -0.105 | -0.339 | -0.519 | -0.656 |
| Saci_0186 | 0.027 | -0.271 | -0.302 | -0.654 |
| Saci_1364 | -0.086 | -0.275 | -0.429 | -0.653 |
| Saci_2365 | 0.131 | -0.203 | -0.758 | -0.652 |
| Saci_0281 | 0.18 | -0.346 | -0.529 | -0.652 |
| Saci_0492 | -0.013 | -0.448 | -0.538 | -0.651 |
| Saci_1329 | -0.071 | -0.049 | -0.784 | -0.651 |
| Saci_1637 | 0.31 | 0.06 | -0.389 | -0.651 |
| Saci_1672 | -0.026 | -0.552 | -0.836 | -0.649 |
| Saci_1536 | 0.236 | -0.016 | -0.705 | -0.649 |
| Saci_1070 | -0.126 | -0.024 | -0.309 | -0.647 |
| Saci_1009 | 0.337 | -0.05 | -0.261 | -0.646 |
| Saci_1368 | 0.38 | 0.047 | -0.675 | -0.645 |
| Saci_1764 | -0.82 | -0.55 | -0.524 | -0.643 |
| Saci_0091 | 0.009 | -0.004 | -0.278 | -0.643 |
| Saci_1295 | 0.439 | -0.27 | -0.737 | -0.642 |
| Saci_0691 | -0.239 | -0.1 | -0.742 | -0.64 |
| Saci_1278 | -0.05 | -0.099 | -0.515 | -0.638 |
| Saci_1279 | -0.133 | -0.405 | -0.288 | -0.637 |
| Saci_2282 | -0.033 | -0.038 | -0.616 | -0.637 |
| Saci_0295 | -0.168 | -0.291 | -0.608 | -0.636 |
| Saci_1374 | -0.132 | -0.729 | -0.836 | -0.631 |
| Saci_2153 | 0.003 | -0.211 | -0.598 | -0.625 |
| Saci_1287 | 0.113 | -0.182 | -0.501 | -0.622 |
| Saci_0618 | 0.045 | -0.042 | -0.399 | -0.622 |
| Saci_0940 | 0.321 | 0.099 | -0.212 | -0.618 |
| Saci_0861 | -0.108 | -0.086 | -0.732 | -0.617 |
| Saci_0722 | 0.147 | -0.426 | -0.528 | -0.614 |
| Saci_0084 | -0.011 | -0.145 | -0.353 | -0.613 |
| Saci_1411 | 0.062 | 0.127 | -0.188 | -0.613 |
| Saci_1607 | -0.637 | -0.649 | -0.747 | -0.611 |
| Saci_1191 | 0.005 | -0.07 | -0.487 | -0.605 |
| Saci_0970 | -0.017 | -0.158 | -0.746 | -0.602 |
| Saci_1963 | 0.164 | -0.071 | -0.592 | -0.601 |
| Saci_0610 | 0.025 | -0.108 | -0.261 | -0.601 |
| Saci_1465 | 0.146 | 0.072 | -0.483 | -0.601 |
| Saci_0666 | 0.104 | 0.104 | -0.612 | -0.598 |
| Saci_1539 | -0.021 | 0.265 | -0.566 | -0.597 |
| Saci_1490 | -0.162 | -0.299 | -0.948 | -0.595 |
| Saci_0779 | 0.152 | -0.262 | -0.514 | -0.59 |
| Saci_1430 | 0.108 | 0.148 | -0.281 | -0.584 |
| Saci_2337 | -0.203 | -0.337 | -0.343 | -0.583 |
| Saci_0232 | -0.303 | -0.026 | -0.57 | -0.58 |
| Saci_0015 | 0.015 | -0.134 | -0.31 | -0.58 |
| Saci_0020 | 0.116 | -0.392 | -0.649 | -0.578 |
| Saci_2119 | -0.017 | 0.173 | -0.823 | -0.578 |
| Saci_1208 | -0.216 | -0.149 | -0.495 | -0.576 |
| Saci_0774 | -0.013 | -0.188 | -0.288 | -0.576 |
| Saci_0977 | 0.379 | 0.132 | -0.135 | -0.575 |
| Saci_0962 | -0.168 | -0.146 | -0.511 | -0.574 |
| Saci_0811 | -0.078 | -0.093 | -0.412 | -0.574 |
| Saci_1037 | -0.171 | -0.179 | 0.043 | -0.573 |
| Saci_0278 | -0.182 | 0.003 | -0.588 | -0.572 |
| Saci_1201 | 0.24 | -0.078 | -0.356 | -0.571 |
| Saci_0960 | 0.023 | 0.079 | -0.5 | -0.57 |
| Saci_0226 | -0.283 | -0.244 | -0.558 | -0.569 |
| Saci_0225 | -0.166 | -0.092 | -0.157 | -0.569 |
| Saci_0961 | 0.029 | 0.1 | -0.457 | -0.568 |
| Saci_0577 | -0.532 | -0.234 | -0.716 | -0.567 |
| Saci_1915 | -0.539 | -0.45 | -0.286 | -0.567 |
| Saci_1188 | -0.149 | -0.15 | -0.302 | -0.565 |
| Saci_1369 | 0.014 | -0.091 | -0.465 | -0.565 |
| Saci_0300 | 0.148 | -0.059 | -0.442 | -0.565 |
| Saci_1588 | 0.102 | -0.134 | -0.553 | -0.561 |
| Saci_0399 | -0.04 | -0.318 | -0.515 | -0.559 |
| Saci_0243 | -0.331 | -0.055 | -0.458 | -0.558 |
| Saci_2236 | 0.164 | -0.14 | -0.326 | -0.557 |
| Saci_1763 | -0.775 | -0.154 | -0.025 | -0.554 |
| Saci_1888 | -0.102 | -0.249 | -0.409 | -0.554 |
| Saci_0552 | -0.203 | -0.159 | -0.471 | -0.553 |
| Saci_0252 | 0.165 | -0.098 | -0.376 | -0.546 |
| Saci_1960 | -0.442 | -0.393 | -0.22 | -0.545 |
| Saci_0081 | -0.072 | -0.119 | -0.912 | -0.544 |
| Saci_0959 | 0.071 | 0.076 | -0.358 | -0.542 |
| Saci_0744 | 0.031 | -0.294 | -0.627 | -0.541 |
| Saci_0999 | -0.021 | 0.065 | -0.336 | -0.541 |
| Saci_0309 | 0.263 | 0.095 | -0.224 | -0.536 |
| Saci_0154 | 0.106 | -0.098 | -0.426 | -0.535 |
| Saci_1547 | 0.29 | 0.148 | -0.456 | -0.534 |
| Saci_0545 | 0.119 | -0.281 | -0.653 | -0.531 |
| Saci_1518 | 0.244 | 0.102 | -0.164 | -0.528 |
| Saci_2239 | 0.345 | 0.004 | -0.507 | -0.526 |
| Saci_0082 | -0.361 | -0.257 | -0.408 | -0.525 |
| Saci_0315 | 0.318 | -0.115 | -0.202 | -0.522 |
| Saci_2176 | 0.374 | 0.256 | -0.295 | -0.522 |
| Saci_0102 | -0.194 | -0.505 | -0.751 | -0.516 |
| Saci_2375 | -0.346 | -0.131 | -0.673 | -0.516 |
| Saci_1979 | 0.049 | -0.131 | -0.246 | -0.515 |
| Saci_0086 | -0.104 | -0.055 | -0.258 | -0.514 |
| Saci_1449 | -0.067 | 0.117 | -0.383 | -0.513 |
| Saci_0696 | -0.032 | -0.106 | -0.745 | -0.512 |
| Saci_0900 | 0.015 | -0.353 | -0.289 | -0.511 |
| Saci_1561 | 0.188 | -0.114 | -0.259 | -0.507 |
| Saci_1792 | 0.386 | 0.016 | -0.415 | -0.502 |
| Saci_0983 | -0.264 | -0.45 | -0.361 | -0.501 |
| Saci_0292 | -0.059 | -0.259 | -0.256 | -0.501 |
| Saci_1520 | -0.761 | -0.235 | -0.468 | -0.5 |
| Saci_0700 | 0.047 | 0.012 | -0.437 | -0.499 |
| Saci_0398 | -0.065 | -0.121 | 0.045 | -0.499 |
| Saci_1582 | -0.26 | -0.198 | -0.364 | -0.498 |
| Saci_0443 | 0.259 | 0.071 | -0.421 | -0.497 |
| Saci_0249 | 0.544 | 0.056 | -0.258 | -0.497 |
| Saci_1799 | 0.256 | -0.232 | -0.442 | -0.492 |
| Saci_0200 | -0.302 | -0.779 | -0.623 | -0.491 |
| Saci_0637 | -0.291 | -0.137 | -0.244 | -0.491 |
| Saci_0828 | -0.079 | -0.046 | -0.459 | -0.49 |
| Saci_0770 | 0.338 | 0.113 | -0.39 | -0.49 |
| Saci_0053 | 0.078 | 0.15 | -0.157 | -0.489 |
| Saci_0757 | -0.05 | -0.371 | -0.582 | -0.487 |
| Saci_0009 | 0.042 | -0.033 | -0.61 | -0.487 |
| Saci_1564 | -0.079 | -0.085 | -0.28 | -0.487 |
| Saci_0948 | 0.29 | -0.053 | -0.496 | -0.486 |
| Saci_0837 | 0.078 | -0.056 | -0.146 | -0.485 |
| Saci_1544 | -0.128 | -0.165 | -0.293 | -0.484 |
| Saci_2165 | 0.496 | -0.047 | -0.521 | -0.484 |
| Saci_2126 | 0.321 | 0.045 | -0.062 | -0.484 |
| Saci_1622 | 0.237 | -0.109 | -0.523 | -0.483 |
| Saci_1377 | 0.05 | 0.077 | -0.331 | -0.481 |
| Saci_0414 | -0.121 | -0.128 | -0.348 | -0.479 |
| Saci_0214 | -0.322 | -0.532 | -0.658 | -0.478 |
| Saci_1200 | 0.08 | -0.078 | -0.288 | -0.476 |
| Saci_1864 | 0.221 | -0.177 | -0.311 | -0.474 |
| Saci_0894 | -0.094 | 0.035 | -0.317 | -0.473 |
| Saci_1597 | 0.247 | 0.132 | -0.19 | -0.473 |
| Saci_0806 | 0.208 | 0.099 | -0.205 | -0.472 |
| Saci_0755 | 0.037 | -0.128 | -0.4 | -0.47 |
| Saci_1691 | 0.225 | -0.27 | -0.312 | -0.466 |
| Saci_1600 | 0.034 | -0.034 | -0.431 | -0.465 |
| Saci_0206 | 0.206 | -0.005 | -0.12 | -0.464 |
| Saci_2364 | 0.082 | -0.068 | -0.031 | -0.463 |
| Saci_1034 | -0.516 | -0.178 | 0.055 | -0.462 |
| Saci_2285 | -0.033 | -0.253 | -0.436 | -0.461 |
| Saci_1940 | -0.979 | -0.599 | -0.473 | -0.46 |
| Saci_2286 | -0.087 | 0.015 | -0.04 | -0.46 |
| Saci_2341 | -0.539 | -0.296 | 0.124 | -0.459 |
| Saci_0493 | 0.058 | -0.052 | -0.358 | -0.458 |
| Saci_0138 | -0.23 | -0.393 | -0.329 | -0.456 |
| Saci_0737 | -0.209 | -0.266 | -0.293 | -0.455 |
| Saci_1728 | -0.373 | -0.234 | -0.305 | -0.454 |
| Saci_1199 | 0.408 | 0.183 | -0.292 | -0.454 |
| Saci_1027 | 0.126 | 0.066 | -0.416 | -0.448 |
| Saci_0539 | 0.025 | -0.056 | -0.325 | -0.447 |
| Saci_0853 | -0.091 | -0.102 | -0.568 | -0.443 |
| Saci_0586 | 0.077 | 0.021 | -0.325 | -0.443 |
| Saci_1307 | -0.285 | -0.509 | -0.993 | -0.441 |
| Saci_1586 | -0.109 | 0.272 | -0.117 | -0.441 |
| Saci_1370 | -0.131 | -0.15 | -0.388 | -0.44 |
| Saci_2263 | -0.162 | -0.204 | -0.149 | -0.439 |
| Saci_1705 | -0.39 | -0.239 | -0.15 | -0.438 |
| Saci_1581 | 0.04 | -0.26 | -0.17 | -0.437 |
| Saci_1699 | 0.211 | -0.148 | -0.372 | -0.437 |
| Saci_0930 | 0.109 | 0.064 | -0.417 | -0.437 |
| Saci_2362 | -0.13 | -0.249 | -0.431 | -0.436 |
| Saci_1985 | -0.379 | -0.244 | -0.268 | -0.435 |
| Saci_1848 | -0.322 | -0.693 | -0.183 | -0.434 |
| Saci_0600 | -0.047 | -0.179 | -0.097 | -0.434 |
| Saci_0964 | 0.211 | 0.078 | -0.453 | -0.432 |
| Saci_1749 | -0.135 | -0.119 | -0.49 | -0.431 |
| Saci_0936 | 0.128 | -0.127 | -0.509 | -0.431 |
| Saci_1791 | 0.606 | -0.144 | -0.506 | -0.429 |
| Saci_0860 | -0.178 | 0.003 | -0.59 | -0.428 |
| Saci_0581 | -0.139 | -0.095 | -0.199 | -0.426 |
| Saci_1968 | -0.309 | -0.408 | -0.457 | -0.425 |
| Saci_0092 | 0.147 | 0.015 | -0.432 | -0.425 |
| Saci_0270 | 0.045 | 0.089 | -0.328 | -0.425 |
| Saci_1470 | 0.079 | 0.066 | -0.175 | -0.425 |
| Saci_1393 | 0.034 | 0.299 | 0.082 | -0.424 |
| Saci_1316 | 0.091 | -0.027 | -0.25 | -0.423 |
| Saci_1038 | -0.649 | -0.53 | -0.313 | -0.421 |
| Saci_1601 | 0.185 | -0.21 | -0.491 | -0.421 |
| Saci_1803 | 0.357 | 0.191 | -0.414 | -0.421 |
| Saci_2041 | -0.045 | 0.025 | 0 | -0.418 |
| Saci_1317 | 0.176 | 0.155 | 0.033 | -0.418 |
| Saci_0902 | -0.06 | -0.338 | -0.52 | -0.417 |
| Saci_1958 | -0.154 | -0.157 | -0.385 | -0.417 |
| Saci_1538 | -0.005 | 0.205 | -0.566 | -0.417 |
| Saci_1507 | -0.002 | 0.131 | -0.25 | -0.417 |
| Saci_1325 | -0.106 | -0.181 | -0.215 | -0.414 |
| Saci_1576 | 0.113 | -0.155 | -0.235 | -0.414 |
| Saci_1871 | 0.055 | 0.06 | 0.001 | -0.414 |
| Saci_2367 | 0.053 | -0.297 | -0.609 | -0.413 |
| Saci_2290 | -0.037 | -0.328 | -0.355 | -0.412 |
| Saci_1841 | -0.228 | -0.356 | -0.568 | -0.411 |
| Saci_1040 | 0.14 | -0.027 | -0.418 | -0.411 |
| Saci_1473 | 0.146 | -0.051 | -0.244 | -0.411 |
| Saci_0802 | -0.016 | -0.22 | -0.374 | -0.409 |
| Saci_1224 | -0.172 | -0.08 | -0.214 | -0.407 |
| Saci_0790 | 0.097 | -0.081 | -0.185 | -0.407 |
| Saci_0450 | -0.048 | -0.082 | -0.295 | -0.405 |
| Saci_0995 | 0.271 | 0.02 | -0.569 | -0.402 |
| Saci_0141 | -0.183 | -0.204 | -0.274 | -0.399 |
| Saci_1379 | 0.137 | -0.172 | -0.496 | -0.398 |
| Saci_1649 | 0.484 | -0.117 | -0.565 | -0.397 |
| Saci_0765 | 0.363 | 0.123 | -0.254 | -0.397 |
| Saci_1521 | 0.212 | -0.056 | -0.141 | -0.395 |
| Saci_0695 | 0.018 | -0.035 | -0.601 | -0.392 |
| Saci_1591 | 0.334 | -0.098 | -0.492 | -0.391 |
| Saci_1121 | -0.114 | -0.156 | 0.071 | -0.391 |
| Saci_1702 | -0.035 | -0.032 | -0.156 | -0.389 |
| Saci_2277 | -0.012 | -0.171 | -0.039 | -0.388 |
| Saci_1742 | 0.122 | -0.048 | -0.147 | -0.385 |
| Saci_2143 | 0.436 | -0.052 | -0.219 | -0.385 |
| Saci_2343 | -0.527 | -0.321 | 0.019 | -0.384 |
| Saci_0150 | 0.075 | -0.02 | -0.271 | -0.384 |
| Saci_0758 | -0.053 | 0.031 | -0.488 | -0.383 |
| Saci_1463 | -0.028 | 0.175 | -0.749 | -0.382 |
| Saci_1357 | -0.39 | -0.056 | -0.03 | -0.382 |
| Saci_1053 | 0.382 | 0.054 | -0.357 | -0.382 |
| Saci_1386 | -0.015 | -0.041 | -0.012 | -0.381 |
| Saci_1939 | 0.237 | 0.089 | -0.177 | -0.381 |
| Saci_0435 | 0.048 | 0.094 | 0.03 | -0.381 |
| Saci_0971 | -0.054 | -0.274 | -0.372 | -0.38 |
| Saci_0165 | 0.021 | -0.09 | -0.352 | -0.38 |
| Saci_0432 | -0.524 | -0.432 | -0.551 | -0.379 |
| Saci_1642 | -0.149 | -0.076 | -0.265 | -0.379 |
| Saci_1328 | 0.239 | 0.096 | -0.205 | -0.378 |
| Saci_1573 | -0.03 | -0.087 | -0.315 | -0.375 |
| Saci_0168 | -0.094 | 0.161 | -0.376 | -0.375 |
| Saci_1929 | 0.165 | -0.071 | -0.354 | -0.373 |
| Saci_1198 | 0.178 | -0.061 | -0.009 | -0.373 |
| Saci_0144 | 0.116 | -0.711 | -0.509 | -0.371 |
| Saci_0276 | -0.588 | -0.106 | -0.751 | -0.37 |
| Saci_0909 | -0.156 | -0.221 | -0.442 | -0.37 |
| Saci_1304 | -0.328 | -0.096 | -0.372 | -0.37 |
| Saci_0318 | 0.094 | 0.12 | -0.189 | -0.369 |
| Saci_0378 | 0.344 | -0.043 | -0.011 | -0.367 |
| Saci_0059 | 0.335 | -0.072 | -0.449 | -0.365 |
| Saci_2139 | 0.536 | 0.006 | -0.638 | -0.365 |
| Saci_1904 | -0.483 | -0.195 | -0.701 | -0.363 |
| Saci_0580 | -0.186 | 0.206 | -0.385 | -0.363 |
| Saci_0454 | 0.142 | -0.049 | -0.25 | -0.362 |
| Saci_1085 | 0.129 | -0.306 | -0.653 | -0.359 |
| Saci_1428 | -0.052 | -0.006 | -0.054 | -0.358 |
| Saci_0901 | 0.109 | -0.347 | -0.496 | -0.357 |
| Saci_2177 | -0.042 | 0.053 | -0.174 | -0.355 |
| Saci_1218 | -0.152 | -0.306 | -0.739 | -0.354 |
| Saci_1215 | 0.111 | 0.039 | -0.662 | -0.353 |
| Saci_1652 | 0.047 | 0.007 | -0.24 | -0.352 |
| Saci_0845 | -0.028 | 0.131 | -0.236 | -0.352 |
| Saci_0365 | 0.085 | -0.29 | -0.183 | -0.35 |
| Saci_1828 | -0.002 | -0.264 | -0.163 | -0.347 |
| Saci_0982 | 0.224 | 0.092 | -0.146 | -0.347 |
| Saci_0833 | 0.105 | 0.033 | -0.533 | -0.345 |
| Saci_1152 | -0.009 | 0.103 | -0.523 | -0.343 |
| Saci_0140 | 0.347 | -0.107 | -0.163 | -0.341 |
| Saci_0426 | 0.316 | 0.121 | -0.278 | -0.341 |
| Saci_1578 | 0.403 | 0.019 | -0.157 | -0.338 |
| Saci_1130 | -0.241 | -0.121 | -0.692 | -0.337 |
| Saci_1314 | -0.143 | -0.011 | -0.174 | -0.337 |
| Saci_0369 | 0.168 | 0.051 | -0.268 | -0.336 |
| Saci_1916 | -0.379 | -0.369 | -0.247 | -0.335 |
| Saci_1636 | 0.059 | -0.068 | -0.36 | -0.335 |
| Saci_0931 | 0.143 | 0.267 | -0.37 | -0.334 |
| Saci_1560 | -0.174 | -0.211 | -0.446 | -0.332 |
| Saci_1385 | 0 | -0.024 | -0.238 | -0.332 |
| Saci_2134 | -0.254 | -0.034 | -0.236 | -0.33 |
| Saci_0542 | 0.092 | 0.122 | -0.244 | -0.328 |
| Saci_2203 | -0.03 | -0.192 | -0.111 | -0.327 |
| Saci_0979 | -0.074 | 0.124 | 0.001 | -0.323 |
| Saci_0069 | 0.175 | -0.167 | -0.254 | -0.322 |
| Saci_0124 | -0.102 | -0.252 | -0.976 | -0.32 |
| Saci_0776 | -0.071 | -0.22 | -0.177 | -0.32 |
| Saci_0219 | -0.303 | -0.073 | 0.136 | -0.32 |
| Saci_1657 | 0.101 | -0.033 | -0.073 | -0.32 |
| Saci_1910 | -0.31 | -0.081 | -0.058 | -0.319 |
| Saci_1472 | -0.048 | 0.148 | -0.272 | -0.317 |
| Saci_0788 | 0.244 | 0.092 | -0.236 | -0.316 |
| Saci_0245 | 0.025 | 0.029 | -0.26 | -0.314 |
| Saci_0247 | -0.126 | -0.197 | -0.255 | -0.313 |
| Saci_2301 | -0.229 | -0.157 | -0.088 | -0.313 |
| Saci_1965 | -0.053 | -0.196 | -0.194 | -0.313 |
| Saci_1627 | 0.185 | -0.019 | -0.323 | -0.313 |
| Saci_0617 | -0.189 | -0.23 | -0.523 | -0.312 |
| Saci_1380 | -0.226 | -0.204 | -0.281 | -0.312 |
| Saci_0592 | -0.269 | -0.045 | -0.221 | -0.312 |
| Saci_1569 | 0.333 | -0.066 | -0.52 | -0.312 |
| Saci_1246 | 0.314 | -0.032 | -0.105 | -0.308 |
| Saci_0708 | -0.243 | -0.013 | -0.415 | -0.307 |
| Saci_0859 | 0.008 | 0.124 | -0.532 | -0.307 |
| Saci_1545 | -0.304 | -0.136 | -0.332 | -0.306 |
| Saci_0172 | -0.23 | -0.002 | -0.021 | -0.306 |
| Saci_1906 | -0.027 | -0.089 | 0.093 | -0.305 |
| Saci_0423 | 0.071 | 0.045 | -0.016 | -0.304 |
| Saci_0907 | 0.204 | 0.077 | -0.378 | -0.303 |
| Saci_0862 | -0.232 | -0.072 | -0.246 | -0.301 |
| Saci_1308 | -0.073 | -0.111 | -0.249 | -0.301 |
| Saci_2265 | 0.038 | 0.007 | -0.306 | -0.301 |
| Saci_0391 | -0.161 | 0.086 | -0.178 | -0.3 |
| Saci_1376 | 0.086 | -0.236 | -0.862 | -0.299 |
| Saci_1244 | 0.135 | -0.045 | -0.313 | -0.299 |
| Saci_0553 | -0.214 | -0.117 | -0.244 | -0.297 |
| Saci_0633 | 0.161 | -0.209 | -0.209 | -0.297 |
| Saci_2318 | -0.054 | 0.186 | 0.009 | -0.295 |
| Saci_1596 | 0.07 | -0.029 | -0.217 | -0.293 |
| Saci_0363 | 0.08 | -0.144 | -0.024 | -0.293 |
| Saci_1571 | 0.2 | 0.022 | -0.251 | -0.292 |
| Saci_0963 | 0.093 | -0.162 | -0.164 | -0.291 |
| Saci_0992 | -0.028 | -0.046 | -0.352 | -0.289 |
| Saci_1621 | -0.164 | 0.044 | -0.196 | -0.289 |
| Saci_1261 | 0.233 | -0.177 | -0.286 | -0.289 |
| Saci_0003 | -0.217 | 0.218 | -0.278 | -0.288 |
| Saci_0375 | 0.075 | 0.235 | -0.03 | -0.288 |
| Saci_1659 | -0.023 | -0.105 | -0.114 | -0.286 |
| Saci_1459 | -0.18 | -0.169 | -1.292 | -0.285 |
| Saci_0871 | -0.085 | 0.017 | -0.227 | -0.285 |
| Saci_1611 | -0.066 | -0.15 | -0.33 | -0.284 |
| Saci_2260 | -0.054 | -0.086 | -0.246 | -0.284 |
| Saci_1807 | 0.193 | 0.225 | -0.156 | -0.284 |
| Saci_1035 | -0.321 | -0.003 | -0.088 | -0.283 |
| Saci_2254 | 0.139 | -0.066 | -0.016 | -0.282 |
| Saci_1187 | 0.078 | -0.145 | -0.262 | -0.28 |
| Saci_2363 | -0.193 | -0.465 | -0.45 | -0.279 |
| Saci_0005 | -0.032 | 0.051 | -0.052 | -0.279 |
| Saci_0635 | -0.048 | 0.055 | -0.284 | -0.278 |
| Saci_1872 | 0.08 | -0.175 | -0.025 | -0.278 |
| Saci_0039 | 0.131 | -0.07 | -0.116 | -0.278 |
| Saci_1610 | 0.156 | -0.097 | -0.063 | -0.278 |
| Saci_0060 | -0.226 | 0.03 | 0.016 | -0.277 |
| Saci_1693 | -0.193 | -0.223 | 0.023 | -0.275 |
| Saci_0671 | -0.048 | -0.047 | -0.157 | -0.274 |
| Saci_1694 | -0.143 | -0.094 | -0.447 | -0.272 |
| Saci_0768 | -0.385 | -0.055 | -0.197 | -0.272 |
| Saci_1192 | -0.09 | -0.172 | -0.266 | -0.272 |
| Saci_2262 | 0.02 | -0.12 | -0.184 | -0.272 |
| Saci_1010 | -0.089 | -0.069 | -0.511 | -0.27 |
| Saci_0911 | 0.198 | 0.119 | -0.23 | -0.27 |
| Saci_0829 | -0.009 | -0.395 | -0.659 | -0.269 |
| Saci_1612 | 0.094 | -0.039 | 0.064 | -0.268 |
| Saci_0266 | 0.196 | -0.221 | -0.244 | -0.267 |
| Saci_0127 | 0.125 | 0.02 | -0.336 | -0.267 |
| Saci_0609 | -0.133 | 0.062 | -0.143 | -0.266 |
| Saci_1548 | -0.207 | -0.059 | 0.326 | -0.266 |
| Saci_1618 | 0.333 | 0.073 | -0.332 | -0.265 |
| Saci_0756 | 0.205 | -0.05 | -0.371 | -0.264 |
| Saci_1865 | 0.22 | -0.022 | -0.202 | -0.264 |
| Saci_0670 | -0.141 | 0.204 | -0.397 | -0.263 |
| Saci_0353 | 0.17 | 0.035 | -0.157 | -0.263 |
| Saci_2073 | 0.069 | -0.018 | -0.215 | -0.262 |
| Saci_0093 | 0.089 | -0.011 | -0.142 | -0.261 |
| Saci_1668 | -0.091 | -0.148 | 0.147 | -0.259 |
| Saci_0256 | 0.141 | -0.071 | -0.073 | -0.257 |
| Saci_2298 | 0.114 | 0.017 | 0.034 | -0.255 |
| Saci_0265 | -0.321 | -0.304 | -0.396 | -0.254 |
| Saci_1918 | -0.125 | -0.038 | -0.209 | -0.254 |
| Saci_2036 | -0.085 | -0.241 | -0.27 | -0.253 |
| Saci_1464 | 0.21 | -0.042 | -0.685 | -0.252 |
| Saci_2091 | -0.215 | -0.191 | -0.008 | -0.251 |
| Saci_0117 | 0.053 | 0.123 | -0.079 | -0.251 |
| Saci_1535 | 0.195 | -0.013 | 0.042 | -0.251 |
| Saci_1682 | 0.013 | -0.107 | -0.342 | -0.25 |
| Saci_1119 | 0 | -0.254 | -0.122 | -0.25 |
| Saci_2271 | -0.233 | -0.056 | 0.198 | -0.25 |
| Saci_0424 | 0.147 | 0.005 | -0.091 | -0.25 |
| Saci_1045 | -0.378 | -0.14 | 0.094 | -0.248 |
| Saci_0312 | 0.087 | -0.198 | 0.048 | -0.247 |
| Saci_0929 | 0.347 | 0.019 | -0.144 | -0.247 |
| Saci_0954 | 0.01 | 0.16 | -0.132 | -0.246 |
| Saci_0777 | 0.135 | 0.007 | -0.291 | -0.244 |
| Saci_1909 | -0.212 | -0.123 | -0.361 | -0.243 |
| Saci_0843 | -0.15 | -0.214 | -0.303 | -0.243 |
| Saci_0787 | -0.355 | -0.068 | -0.01 | -0.243 |
| Saci_0070 | 0.16 | 0.099 | -0.296 | -0.241 |
| Saci_0720 | 0.007 | 0.159 | -0.172 | -0.24 |
| Saci_0405 | 0.045 | 0.316 | -0.292 | -0.24 |
| Saci_0258 | -0.291 | -0.074 | -0.166 | -0.239 |
| Saci_1255 | -0.118 | 0.2 | -0.172 | -0.239 |
| Saci_0641 | -0.588 | -0.356 | -0.735 | -0.238 |
| Saci_1584 | -0.158 | 0.042 | -0.057 | -0.238 |
| Saci_0728 | 0.425 | -0.157 | -0.322 | -0.238 |
| Saci_0990 | 0.023 | -0.054 | 0.014 | -0.238 |
| Saci_2349 | -0.132 | -0.249 | -0.368 | -0.237 |
| Saci_1624 | -0.078 | -0.002 | -0.482 | -0.237 |
| Saci_1967 | -0.036 | -0.21 | -0.168 | -0.237 |
| Saci_1231 | -0.328 | -0.115 | -0.131 | -0.236 |
| Saci_0465 | -0.047 | -0.12 | -0.166 | -0.236 |
| Saci_1557 | -0.276 | 0.153 | 0.181 | -0.235 |
| Saci_0446 | -0.189 | 0.089 | -0.265 | -0.234 |
| Saci_0663 | 0.137 | -0.024 | -0.19 | -0.232 |
| Saci_0952 | 0.182 | -0.022 | -0.113 | -0.232 |
| Saci_1631 | -0.179 | -0.357 | -0.316 | -0.231 |
| Saci_0807 | 0 | -0.139 | -0.326 | -0.231 |
| Saci_0684 | 0.12 | 0.155 | -0.131 | -0.23 |
| Saci_0863 | -0.26 | -0.192 | -0.579 | -0.229 |
| Saci_1003 | -0.013 | 0.159 | -0.107 | -0.229 |
| Saci_1706 | -0.653 | -0.417 | -0.028 | -0.227 |
| Saci_0262 | 0.225 | -0.097 | -0.057 | -0.226 |
| Saci_1623 | 0.03 | -0.134 | -0.313 | -0.225 |
| Saci_0088 | -0.173 | 0.086 | 0.023 | -0.225 |
| Saci_1767 | 0.025 | 0.118 | 0.001 | -0.225 |
| Saci_0422 | 0.201 | 0.127 | -0.136 | -0.225 |
| Saci_1873 | -0.635 | -0.721 | 0.163 | -0.224 |
| Saci_2371 | -0.145 | 0.172 | -0.325 | -0.224 |
| Saci_0250 | -0.338 | -0.103 | -0.125 | -0.223 |
| Saci_0002 | -0.227 | 0.149 | -0.15 | -0.223 |
| Saci_2328 | 0.133 | -0.016 | -0.251 | -0.223 |
| Saci_1289 | -0.071 | -0.215 | -0.167 | -0.222 |
| Saci_2018 | 0.013 | -0.241 | -0.136 | -0.222 |
| Saci_0946 | 0.137 | -0.132 | -0.11 | -0.222 |
| Saci_0043 | -0.177 | -0.182 | -0.122 | -0.213 |
| Saci_0019 | -0.185 | -0.03 | -0.182 | -0.212 |
| Saci_0261 | 0.35 | -0.152 | -0.027 | -0.212 |
| Saci_2162 | -0.263 | -0.23 | 0.012 | -0.211 |
| Saci_1798 | 0.284 | 0.115 | -0.315 | -0.211 |
| Saci_0703 | 0.028 | 0.018 | -0.855 | -0.209 |
| Saci_0199 | 0.004 | 0.052 | -0.128 | -0.209 |
| Saci_1067 | -0.225 | -0.514 | -0.221 | -0.208 |
| Saci_1598 | 0.002 | -0.078 | 0.09 | -0.208 |
| Saci_1266 | -0.045 | -0.099 | -0.138 | -0.206 |
| Saci_1541 | 0.036 | -0.188 | -0.158 | -0.205 |
| Saci_0183 | -0.141 | -0.135 | 0.094 | -0.202 |
| Saci_0972 | 0.148 | -0.072 | -0.167 | -0.202 |
| Saci_1562 | 0.179 | -0.146 | -0.467 | -0.2 |
| Saci_1606 | -0.239 | 0.028 | -0.291 | -0.198 |
| Saci_0991 | -0.034 | 0.149 | -0.085 | -0.198 |
| Saci_1468 | -0.091 | 0.248 | -0.463 | -0.197 |
| Saci_2305 | -0.064 | -0.341 | -0.275 | -0.193 |
| Saci_0244 | -0.078 | 0.073 | -0.121 | -0.191 |
| Saci_2293 | -0.232 | 0.047 | 0.162 | -0.19 |
| Saci_1036 | 0.097 | 0.039 | 0.018 | -0.189 |
| Saci_0260 | 0.2 | 0.046 | -0.077 | -0.189 |
| Saci_1619 | -0.052 | 0.055 | 0.161 | -0.188 |
| Saci_0985 | 0.047 | 0.055 | -0.061 | -0.186 |
| Saci_0888 | -0.06 | 0.085 | 0.052 | -0.185 |
| Saci_1425 | 0.165 | -0.047 | -0.126 | -0.184 |
| Saci_0397 | -0.168 | -0.292 | 0.005 | -0.182 |
| Saci_0052 | -0.057 | -0.117 | -0.049 | -0.182 |
| Saci_0460 | -0.046 | -0.046 | -0.139 | -0.18 |
| Saci_1895 | -0.505 | -0.302 | -0.003 | -0.178 |
| Saci_1550 | -0.332 | -0.055 | -0.043 | -0.177 |
| Saci_1966 | -0.256 | -0.042 | -0.072 | -0.177 |
| Saci_1842 | 0.024 | -0.154 | -0.32 | -0.176 |
| Saci_0958 | 0.054 | -0.021 | 0.003 | -0.175 |
| Saci_1883 | -0.572 | -0.503 | -0.397 | -0.171 |
| Saci_0283 | -0.165 | 0.088 | -0.059 | -0.17 |
| Saci_2087 | -0.517 | -0.029 | 0.286 | -0.169 |
| Saci_1455 | 0.035 | -0.009 | -0.068 | -0.169 |
| Saci_0630 | -0.306 | -0.24 | -0.036 | -0.164 |
| Saci_0159 | 0.117 | -0.421 | -0.191 | -0.164 |
| Saci_1937 | 0.051 | 0.033 | -0.144 | -0.164 |
| Saci_1981 | -0.813 | -0.25 | -0.357 | -0.161 |
| Saci_1283 | 0.077 | 0.011 | -0.226 | -0.161 |
| Saci_0554 | 0.009 | 0.112 | -0.189 | -0.161 |
| Saci_0463 | -0.03 | 0.046 | 0.006 | -0.161 |
| Saci_0146 | -0.005 | -0.08 | -0.254 | -0.16 |
| Saci_0027 | 0.231 | -0.044 | -0.181 | -0.159 |
| Saci_0745 | -0.04 | -0.002 | -0.166 | -0.157 |
| Saci_0187 | -0.043 | -0.058 | -0.009 | -0.157 |
| Saci_0763 | -0.343 | -0.022 | -0.751 | -0.154 |
| Saci_0023 | -0.062 | 0.018 | -0.091 | -0.154 |
| Saci_0967 | 0.144 | -0.034 | -0.394 | -0.152 |
| Saci_0699 | -0.09 | 0.078 | -0.137 | -0.152 |
| Saci_0018 | -0.336 | -0.125 | -0.3 | -0.149 |
| Saci_0953 | 0.086 | -0.064 | -0.085 | -0.149 |
| Saci_0282 | 0.037 | -0.071 | -0.173 | -0.148 |
| Saci_0332 | 0.126 | -0.162 | -0.286 | -0.147 |
| Saci_1676 | 0.004 | -0.023 | -0.039 | -0.144 |
| Saci_2019 | 0.087 | -0.04 | 0.057 | -0.143 |
| Saci_0734 | 0.05 | -0.005 | -0.038 | -0.141 |
| Saci_0727 | 0.005 | -0.228 | -0.676 | -0.14 |
| Saci_2201 | 0.238 | -0.07 | -0.046 | -0.14 |
| Saci_1613 | 0.067 | 0.013 | -0.107 | -0.138 |
| Saci_0061 | -0.07 | 0.092 | 0.064 | -0.137 |
| Saci_0413 | 0.094 | 0.066 | -0.19 | -0.133 |
| Saci_0080 | 0.14 | -0.023 | -0.483 | -0.132 |
| Saci_0620 | 0.058 | 0.067 | -0.317 | -0.131 |
| Saci_0410 | -0.058 | -0.146 | 0.154 | -0.131 |
| Saci_1360 | -0.172 | -0.129 | -0.212 | -0.129 |
| Saci_0164 | -0.084 | -0.012 | -0.082 | -0.129 |
| Saci_2227 | 0.042 | -0.131 | -0.119 | -0.128 |
| Saci_1802 | 0.007 | -0.125 | -0.1 | -0.127 |
| Saci_2294 | -0.194 | 0.022 | 0.271 | -0.127 |
| Saci_2090 | -0.091 | 0.021 | -0.04 | -0.126 |
| Saci_1059 | -0.046 | 0.018 | -0.13 | -0.125 |
| Saci_1698 | -0.367 | -0.209 | -0.002 | -0.124 |
| Saci_1874 | -0.382 | -0.429 | -0.141 | -0.122 |
| Saci_1349 | -0.049 | 0.05 | -0.087 | -0.122 |
| Saci_0042 | 0.017 | -0.021 | -0.074 | -0.122 |
| Saci_1525 | 0.085 | 0.075 | -0.136 | -0.121 |
| Saci_0128 | 0.021 | 0.074 | -0.042 | -0.12 |
| Saci_1014 | -0.217 | 0.061 | -0.04 | -0.118 |
| Saci_2020 | -0.005 | -0.144 | 0.019 | -0.118 |
| Saci_2296 | -0.167 | 0.098 | -0.233 | -0.114 |
| Saci_1645 | -0.028 | -0.12 | -0.006 | -0.113 |
| Saci_2164 | 0.149 | 0.052 | -0.342 | -0.111 |
| Saci_0798 | -0.219 | 0.092 | -0.613 | -0.11 |
| Saci_1510 | -0.043 | 0.04 | 0.075 | -0.11 |
| Saci_0192 | -0.026 | -0.454 | -0.078 | -0.109 |
| Saci_0321 | -0.011 | 0.302 | -0.248 | -0.109 |
| Saci_1087 | 0.071 | -0.201 | -0.233 | -0.108 |
| Saci_1197 | -0.002 | 0.074 | -0.277 | -0.108 |
| Saci_0277 | -0.502 | -0.237 | -0.307 | -0.105 |
| Saci_1860 | -0.028 | 0.062 | -0.239 | -0.105 |
| Saci_2340 | -0.413 | -0.128 | -0.006 | -0.104 |
| Saci_0556 | 0.022 | -0.198 | -0.106 | -0.104 |
| Saci_0230 | 0.029 | 0.06 | -0.198 | -0.104 |
| Saci_1949 | 0.154 | -0.005 | -0.116 | -0.104 |
| Saci_2353 | -0.133 | -0.092 | -0.085 | -0.103 |
| Saci_0792 | 0.096 | -0.009 | -0.191 | -0.103 |
| Saci_1334 | 0.084 | -0.046 | -0.268 | -0.101 |
| Saci_2197 | -0.037 | 0.17 | -0.049 | -0.101 |
| Saci_1781 | -0.278 | -0.255 | -0.174 | -0.1 |
| Saci_1408 | 0.158 | -0.099 | -0.255 | -0.1 |
| Saci_0373 | -0.065 | -0.169 | 0.123 | -0.1 |
| Saci_2246 | -0.539 | -0.353 | -0.047 | -0.098 |
| Saci_2342 | -0.325 | -0.231 | -0.18 | -0.098 |
| Saci_0216 | -0.101 | 0.021 | -0.157 | -0.098 |
| Saci_0761 | 0.22 | 0.007 | -0.205 | -0.098 |
| Saci_1867 | -0.003 | 0.025 | 0.064 | -0.098 |
| Saci_1969 | -0.168 | -0.153 | -0.147 | -0.097 |
| Saci_0896 | 0.204 | -0.026 | -0.2 | -0.097 |
| Saci_1993 | -0.329 | -0.165 | -0.112 | -0.096 |
| Saci_1006 | 0.1 | 0.007 | -0.013 | -0.096 |
| Saci_2172 | 0.032 | -0.143 | -0.168 | -0.093 |
| Saci_1474 | -0.189 | 0.014 | -0.024 | -0.093 |
| Saci_2171 | -0.361 | -0.278 | -0.301 | -0.092 |
| Saci_0994 | -0.596 | -0.059 | 0.055 | -0.091 |
| Saci_1737 | -0.387 | -0.31 | 0.209 | -0.091 |
| Saci_0464 | -0.033 | -0.121 | -0.227 | -0.091 |
| Saci_2354 | -0.413 | 0.2 | 0.103 | -0.091 |
| Saci_0753 | 0.063 | -0.078 | -0.255 | -0.089 |
| Saci_1808 | -0.097 | 0.034 | -0.103 | -0.089 |
| Saci_1724 | 0.188 | 0.016 | -0.141 | -0.089 |
| Saci_0389 | -0.013 | 0.046 | -0.096 | -0.086 |
| Saci_1285 | -0.193 | -0.048 | -0.077 | -0.081 |
| Saci_2135 | 0.07 | -0.093 | -0.604 | -0.08 |
| Saci_0716 | -0.113 | 0.067 | -0.273 | -0.08 |
| Saci_0933 | -0.058 | 0.021 | -0.037 | -0.078 |
| Saci_1927 | 0.039 | -0.135 | -0.305 | -0.077 |
| Saci_0664 | 0.068 | -0.103 | -0.122 | -0.077 |
| Saci_1711 | -0.274 | -0.04 | 0.127 | -0.073 |
| Saci_1928 | 0.074 | 0.019 | -0.182 | -0.073 |
| Saci_0910 | -0.406 | -0.054 | 0.037 | -0.07 |
| Saci_1120 | -0.166 | -0.215 | 0.176 | -0.069 |
| Saci_2373 | 0.01 | 0.05 | -0.108 | -0.069 |
| Saci_1401 | -0.292 | -0.21 | -0.128 | -0.064 |
| Saci_0625 | 0.028 | 0.051 | -0.098 | -0.061 |
| Saci_1254 | -0.108 | 0.018 | 0.103 | -0.061 |
| Saci_0458 | -0.055 | 0.047 | 0.024 | -0.055 |
| Saci_2017 | -0.322 | -0.343 | -0.305 | -0.054 |
| Saci_1948 | -0.041 | 0.022 | -0.145 | -0.052 |
| Saci_0428 | -0.049 | -0.13 | -0.025 | -0.051 |
| Saci_1608 | -0.408 | -0.426 | -0.325 | -0.049 |
| Saci_1414 | -0.119 | 0.058 | -0.087 | -0.049 |
| Saci_1094 | -0.066 | 0.057 | -0.02 | -0.048 |
| Saci_1934 | 0.048 | -0.177 | 0.005 | -0.046 |
| Saci_0031 | -0.276 | -0.068 | 0.253 | -0.046 |
| Saci_0987 | 0.001 | -0.009 | -0.054 | -0.045 |
| Saci_1388 | -0.138 | 0.115 | -0.333 | -0.044 |
| Saci_0057 | -0.062 | -0.044 | 0.001 | -0.044 |
| Saci_1524 | 0.003 | -0.016 | 0 | -0.043 |
| Saci_0173 | -0.128 | -0.092 | -0.293 | -0.042 |
| Saci_0188 | -0.11 | 0.069 | -0.127 | -0.042 |
| Saci_0048 | -0.227 | 0.033 | -0.254 | -0.041 |
| Saci_1760 | -0.4 | -0.112 | 0.29 | -0.039 |
| Saci_1897 | -0.305 | -0.283 | -0.279 | -0.038 |
| Saci_0926 | 0.024 | -0.025 | -0.016 | -0.036 |
| Saci_2372 | -0.154 | -0.04 | -0.093 | -0.032 |
| Saci_0388 | -0.169 | 0.03 | -0.032 | -0.032 |
| Saci_0228 | 0.031 | -0.059 | -0.026 | -0.032 |
| Saci_1641 | -0.007 | -0.235 | -0.049 | -0.03 |
| Saci_0877 | 0.038 | 0.01 | -0.026 | -0.03 |
| Saci_1080 | -0.044 | -0.141 | -0.11 | -0.028 |
| Saci_2368 | 0.23 | -0.039 | -0.248 | -0.026 |
| Saci_1919 | -0.103 | 0.024 | 0.013 | -0.025 |
| Saci_1590 | 0.143 | -0.056 | -0.347 | -0.024 |
| Saci_0415 | -0.095 | 0.248 | -0.284 | -0.023 |
| Saci_0844 | 0.11 | -0.053 | -0.181 | -0.023 |
| Saci_2060 | -0.003 | 0.013 | -0.009 | -0.023 |
| Saci_1716 | -0.17 | -0.088 | -0.09 | -0.022 |
| Saci_0208 | -0.073 | -0.059 | 0.005 | -0.022 |
| Saci_2352 | -0.241 | -0.015 | 0.005 | -0.02 |
| Saci_0643 | -0.019 | -0.013 | -0.007 | -0.02 |
| Saci_0374 | 0.041 | -0.093 | 0.07 | -0.02 |
| Saci_1277 | -0.061 | -0.132 | -0.296 | -0.019 |
| Saci_1976 | 0.012 | -0.141 | -0.011 | -0.019 |
| Saci_0221 | 0 | -0.151 | -0.212 | -0.018 |
| Saci_0218 | -0.005 | -0.027 | -0.237 | -0.018 |
| Saci_2115 | -0.057 | -0.082 | -0.186 | -0.017 |
| Saci_2037 | -0.076 | -0.149 | -0.095 | -0.017 |
| Saci_0543 | 0.117 | -0.15 | -0.194 | -0.017 |
| Saci_2250 | -0.013 | 0.01 | 0.001 | -0.017 |
| Saci_1272 | -0.572 | -0.361 | -0.161 | -0.016 |
| Saci_0174 | 0.017 | -0.02 | -0.004 | -0.016 |
| Saci_0110 | 0.003 | -0.011 | 0.004 | -0.016 |
| Saci_1568 | -0.021 | 0 | -0.087 | -0.014 |
| Saci_0893 | -0.145 | -0.254 | 0.024 | -0.013 |
| Saci_0368 | -0.097 | 0.043 | -0.133 | -0.008 |
| Saci_1454 | -0.09 | -0.161 | 0.022 | -0.007 |
| Saci_0272 | -0.024 | -0.018 | -0.039 | -0.007 |
| Saci_2033 | -0.008 | -0.016 | -0.023 | -0.007 |
| Saci_2169 | -0.167 | -0.024 | -0.216 | -0.006 |
| Saci_2008 | -0.333 | 0.077 | 0.135 | -0.006 |
| Saci_1195 | -0.034 | 0.024 | -0.046 | -0.006 |
| Saci_1007 | -0.187 | -0.111 | -0.009 | -0.005 |
| Saci_0038 | -0.048 | 0.149 | -0.145 | -0.005 |
| Saci_0803 | -0.516 | 0.219 | -0.23 | -0.004 |
| Saci_1247 | -0.108 | -0.265 | -0.371 | -0.003 |
| Saci_0749 | -0.081 | 0.001 | -0.015 | -0.003 |
| Saci_1566 | 0.011 | 0.019 | -0.042 | -0.003 |
| Saci_1321 | -0.142 | -0.059 | -0.093 | -0.001 |
| Saci_0494 | -0.263 | -0.165 | -0.275 | 0 |
| Saci_2061 | -0.022 | 0.016 | -0.033 | 0 |
| Saci_1122 | -0.21 | -0.044 | 0.116 | 0.001 |
| Saci_1382 | -0.063 | 0.021 | -0.096 | 0.003 |
| Saci_0854 | -0.155 | -0.022 | 0.081 | 0.004 |
| Saci_0447 | -0.104 | -0.067 | -0.162 | 0.007 |
| Saci_1673 | 0.07 | -0.059 | -0.155 | 0.007 |
| Saci_1178 | -0.13 | -0.091 | 0.182 | 0.007 |
| Saci_2289 | -0.054 | -0.183 | -0.038 | 0.008 |
| Saci_1447 | 0.03 | -0.146 | 0.034 | 0.008 |
| Saci_1901 | -0.239 | -0.251 | -0.208 | 0.01 |
| Saci_1351 | -0.349 | -0.211 | -0.074 | 0.011 |
| Saci_0133 | -0.067 | -0.063 | -0.213 | 0.011 |
| Saci_1599 | -0.161 | -0.019 | -0.057 | 0.011 |
| Saci_2170 | -0.161 | -0.057 | 0.048 | 0.011 |
| Saci_0612 | -0.032 | -0.099 | 0.058 | 0.011 |
| Saci_0607 | -0.087 | -0.054 | -0.061 | 0.012 |
| Saci_1345 | -0.043 | -0.054 | -0.006 | 0.012 |
| Saci_0011 | -0.165 | -0.046 | 0.119 | 0.012 |
| Saci_2229 | -0.523 | -0.111 | -0.041 | 0.015 |
| Saci_0938 | 0.057 | -0.099 | -0.298 | 0.015 |
| Saci_0004 | -0.204 | 0.036 | -0.051 | 0.016 |
| Saci_2323 | -0.023 | -0.005 | -0.081 | 0.016 |
| Saci_2359 | 0.077 | -0.109 | -0.061 | 0.016 |
| Saci_0814 | -0.132 | -0.22 | 0.177 | 0.017 |
| Saci_0286 | -0.082 | -0.009 | 0.049 | 0.017 |
| Saci_1869 | -0.311 | -0.175 | -0.005 | 0.02 |
| Saci_1243 | -0.086 | -0.07 | 0.042 | 0.021 |
| Saci_0160 | -0.051 | -0.001 | -0.01 | 0.022 |
| Saci_0279 | 0.097 | -0.026 | -0.35 | 0.023 |
| Saci_1245 | -0.122 | 0.098 | -0.076 | 0.023 |
| Saci_1882 | -0.607 | -0.301 | -0.283 | 0.026 |
| Saci_1602 | 0.083 | -0.15 | -0.211 | 0.027 |
| Saci_1722 | -0.142 | -0.162 | 0.03 | 0.027 |
| Saci_0264 | -0.486 | -0.203 | -0.188 | 0.029 |
| Saci_1115 | -0.058 | -0.158 | 0.082 | 0.03 |
| Saci_0139 | -0.416 | -0.457 | -0.017 | 0.032 |
| Saci_0487 | -0.024 | -0.027 | -0.013 | 0.037 |
| Saci_0123 | 0.058 | -0.114 | -0.167 | 0.04 |
| Saci_2045 | -0.192 | -0.005 | -0.045 | 0.042 |
| Saci_0241 | -0.002 | -0.003 | -0.095 | 0.042 |
| Saci_0193 | -0.006 | -0.024 | -0.174 | 0.043 |
| Saci_1962 | -0.357 | -0.02 | -0.187 | 0.044 |
| Saci_0191 | -0.022 | -0.001 | -0.13 | 0.046 |
| Saci_0796 | -0.018 | -0.065 | -0.019 | 0.047 |
| Saci_0759 | -0.119 | -0.057 | 0.029 | 0.049 |
| Saci_1772 | -0.458 | 0.136 | 0.222 | 0.051 |
| Saci_0073 | -0.051 | -0.218 | -0.122 | 0.055 |
| Saci_0593 | -0.085 | -0.085 | 0.04 | 0.055 |
| Saci_0437 | -0.09 | -0.2 | 0.032 | 0.056 |
| Saci_0022 | -0.161 | -0.125 | -0.05 | 0.058 |
| Saci_2142 | -0.23 | -0.029 | 0.013 | 0.058 |
| Saci_0800 | -0.182 | -0.436 | -0.235 | 0.061 |
| Saci_1911 | -0.095 | -0.063 | -0.003 | 0.062 |
| Saci_1982 | -0.706 | -0.066 | 0.166 | 0.066 |
| Saci_0120 | -0.087 | -0.212 | -0.065 | 0.066 |
| Saci_0869 | -0.118 | -0.116 | 0.13 | 0.066 |
| Saci_0984 | -0.275 | -0.221 | -0.021 | 0.069 |
| Saci_1004 | -0.307 | 0.025 | -0.146 | 0.075 |
| Saci_1762 | -0.341 | -0.039 | 0.044 | 0.077 |
| Saci_0884 | -0.034 | -0.024 | -0.12 | 0.077 |
| Saci_0402 | -0.126 | -0.219 | 0.047 | 0.079 |
| Saci_1984 | -0.2 | -0.1 | -0.106 | 0.08 |
| Saci_0495 | -0.208 | 0.073 | 0.018 | 0.08 |
| Saci_2228 | -0.258 | -0.094 | 0.027 | 0.081 |
| Saci_0152 | -0.121 | -0.048 | -0.074 | 0.089 |
| Saci_0485 | -0.203 | 0.059 | -0.152 | 0.096 |
| Saci_1513 | -0.259 | -0.071 | 0.178 | 0.096 |
| Saci_1800 | -0.025 | -0.082 | -0.036 | 0.103 |
| Saci_0605 | -0.062 | -0.026 | -0.324 | 0.104 |
| Saci_1639 | -0.172 | -0.082 | 0.078 | 0.107 |
| Saci_1717 | -0.131 | -0.06 | 0.067 | 0.111 |
| Saci_2011 | -0.462 | -0.176 | 0.212 | 0.115 |
| Saci_1885 | -0.36 | -0.052 | 0.111 | 0.115 |
| Saci_1262 | -0.086 | -0.141 | 0.019 | 0.116 |
| Saci_0370 | -0.213 | 0.02 | 0 | 0.118 |
| Saci_1476 | -0.228 | 0.013 | -0.152 | 0.119 |
| Saci_2028 | -0.53 | -0.201 | 0.312 | 0.121 |
| Saci_1854 | 0.021 | -0.189 | -0.027 | 0.136 |
| Saci_1998 | -0.496 | -0.081 | 0.178 | 0.14 |
| Saci_1319 | -0.222 | -0.067 | -0.072 | 0.141 |
| Saci_2319 | 0.204 | 0.16 | -0.687 | 0.146 |
| Saci_1529 | -0.315 | -0.036 | -0.013 | 0.154 |
| Saci_0156 | -0.296 | -0.047 | 0.068 | 0.154 |
| Saci_1850 | -0.459 | -0.25 | 0.252 | 0.17 |
| Saci_0955 | -0.178 | -0.177 | -0.816 | 0.171 |
| Saci_0685 | -0.61 | -0.034 | -0.209 | 0.171 |
| Saci_0248 | -0.219 | -0.224 | 0.198 | 0.174 |
| Saci_1952 | -0.228 | -0.1 | 0.083 | 0.183 |
| Saci_0462 | 0.015 | -0.321 | 0.093 | 0.196 |
| Saci_1907 | -0.202 | -0.198 | 0.162 | 0.214 |
| Saci_1896 | -0.357 | -0.459 | 0.187 | 0.223 |
| Saci_1049 | -0.106 | -0.263 | -0.057 | 0.231 |
| Saci_0222 | -0.387 | -0.228 | 0.02 | 0.232 |
| Saci_1753 | -0.561 | -0.73 | -0.236 | 0.255 |
| Saci_0109 | -0.255 | -0.269 | 0.117 | 0.26 |
| Saci_0166 | -0.272 | -0.096 | -0.192 | 0.264 |
| Saci_0813 | -0.002 | 0 | -0.477 | 0.272 |
| Saci_1298 | -0.456 | -0.102 | 0.215 | 0.284 |
| Saci_2237 | -0.526 | -0.144 | 0.105 | 0.3 |
| Saci_1604 | -0.446 | -0.179 | 0.028 | 0.379 |
| Saci_1851 | -0.496 | -0.323 | 0.303 | 0.399 |
| Saci_1223 | -0.222 | -0.406 | -0.545 | 0.426 |
| Saci_0724 | -0.601 | -0.337 | 0.454 | 0.476 |
